# Data science enabled discovery of a highly soluble 2,2′-bipyrimidine anolyte for application in a flow battery†

**DOI:** 10.1039/d3sc04084d

**Published:** 2023-11-02

**Authors:** Adam R. Pancoast, Sara L. McCormack, Shelby Galinat, Ryan Walser-Kuntz, Brianna M. Jett, Melanie S. Sanford, Matthew S. Sigman

**Affiliations:** a Department of Chemistry, University of Utah 315 South 1400 East Salt Lake City Utah 84112 USA sigman@chem.utah.edu; b Department of Chemistry, University of Michigan, 930 North University Avenue Ann Arbor Michigan 48109 USA; c Joint Center for Energy Storage Research 9700 S. Cass Avenue Argonne Illinois 60439 USA

## Abstract

Development of non-aqueous redox flow batteries as a viable energy storage solution relies upon the identification of soluble charge carriers capable of storing large amounts of energy over extended time periods. A combination of metrics including number of electrons stored per molecule, redox potential, stability, and solubility of the charge carrier impact performance. In this context, we recently reported a 2,2′-bipyrimidine charge carrier that stores two electrons per molecule with reduction near −2.0 V *vs.* Fc/Fc^+^ and high stability. However, these first-generation derivatives showed a modest solubility of 0.17 M (0.34 M e^−^). Seeking to improve solubility without sacrificing stability, we harnessed the synthetic modularity of this scaffold to design a library of sixteen candidates. Using computed molecular descriptors and a single node decision tree, we found that minimization of the solvent accessible surface area (SASA) can be used to predict derivatives with enhanced solubility. This parameter was used in combination with a heatmap describing stability to de-risk a virtual screen that ultimately identified a 2,2′-bipyrimidine with significantly increased solubility and good stability metrics in the reduced states. This molecule was paired with a cyclopropenium catholyte in a prototype all-organic redox flow battery, achieving a cell potential up to 3 V.

## Abstract 2

Non-linear threshold analysis enabled discovery of a highly soluble (1.3 M) bipyrimidine anolyte. Stable cycling in a full flow battery cell for 75 cycles.

## Graphical abstract

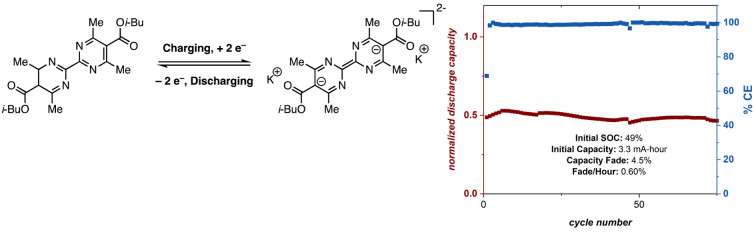

## Introduction

Renewable energy sources, such as solar or wind power, have seen a steep decrease in cost over the last 10 years and consequently are envisioned to play an important role in global energy generation. Indeed, the International Renewable Energy Agency (IRENA) has projected that 90% of electricity will be derived from renewable sources by 2050.^1^ Critical to realizing this goal is further development of effective energy storage technologies, such as redox flow batteries.^2^ Redox flow batteries, which store energy in the form of flowable solutions of oxidized or reduced charge carriers (catholytes and anolytes, respectively), benefit from the separation of the electrolyte reservoirs from the cell stack.^3^ This allows energy to be scaled independently from power, creating a high level of flexibility for various applications.^4^

The current state of the art in redox flow battery technology utilizes aqueous solutions of vanadium-based charge carriers. Despite success on a commercial level, vanadium redox flow batteries face several hurdles to broader utilization as energy storage devices, including modest energy densities due to the relatively narrow electrochemical window of water.^4,5^ Conversely, non-aqueous solvents like acetonitrile present an attractive route to produce high energy density flow batteries due to the enhanced thermodynamic electrochemical window relative to that of water (6.1 *versus* 1.23 V, respectively).^6^ As shown in eqn (1) (where *C*_ap_ is the battery limiting-capacity, *V* is the cell voltage, and *μ* is the volume factor), increasing cell voltage should lead to improved energy densities, with all other factors being equal.^3^

1 Theoretical energy density (W hr L^−1^) = *C*_ap_*V*/*μ*_V_

The key to harnessing the electrochemical window of non-aqueous solvents is increasing the cell voltage through the development of charge carriers with electron transfer events that occur at extreme potentials.^3^ This is difficult due to the propensity of highly energetic oxidized or reduced species to decompose by one or more pathways.^7^ This challenge notwithstanding, there has been significant progress towards utilizing the expanded solvent window with highly reducing (negative) potentials for anolytes^8^ and strongly oxidizing (positive) potentials for catholytes.^9,10^

Beyond stability considerations, another challenge lies in achieving high solubility.^11^ Since conventional redox flow batteries store energy in the form of dissolved redox active molecules, energy density is directly correlated to the charge carrier concentration.^3^ The relationship between capacity and solubility has prompted efforts to understand the molecular features dictating solubility for flow battery materials,^12,13^ as well as the introduction of an alternative configurations based on an insoluble charge storing species.^14^ There are practical limitations to solubility, as the tradeoff between energy density, solution viscosity, and conductivity can become unfavorable at higher concentrations of active electrolyte in non-aqueous systems.^15^ Increasing solution viscosity can decrease mass transfer of both active and supporting electrolytes, ultimately causing decreased conductivity, increased overpotentials, and impeded battery performance.^16^

2 Theoretical capacity (A hr L^−1^) = *nCF*/3600

Considering the tradeoff of solubility and viscosity, a general target of ∼0.5 M for charge carriers was proposed by Zhang and coworkers in 2018 as an upper limit for solubility due to negative impacts of mass transfer above this concentration.^15^ Shown by eqn (2), where *n* is the number of electrons transferred, *C* is the concentration of the anolyte/catholyte, *F* is Faraday's constant, and 3600 is a conversion factor between coulombs and amp-hours, battery capacity is not solely dependent upon solubility.^3^ As capacity is a measure of a batteries ability to store charge, anolytes/catholytes capable of storing multiple electrons per molecule will also increase battery capacity through an increased effective electron concentration while limiting mass-transfer issues present in systems with high molecular concentration.

**Fig. 1:**
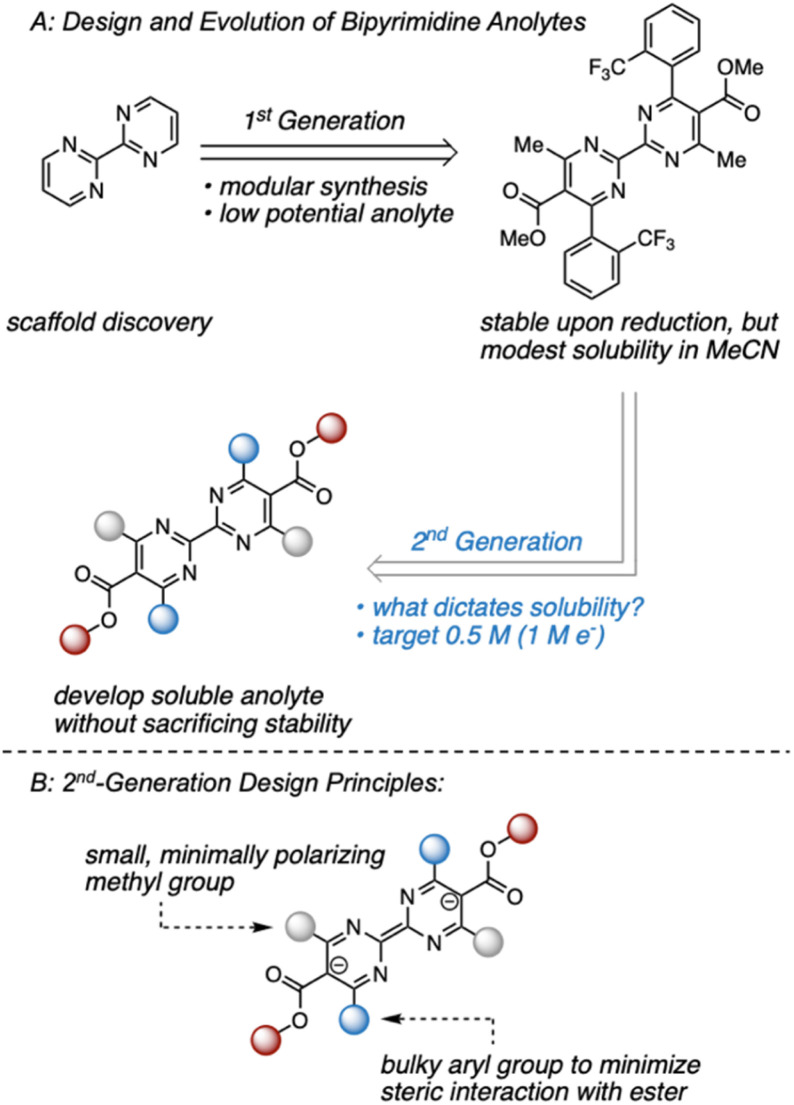
(A) Evolution of bipyrimidine anolytes. (B) Design principles of 2nd-generation candidates aimed at maintaining a stable reduced state.

Within this framework, we recently disclosed a promising class of anolytes based on the 2,2′-bipyrimidine scaffold.^17^ These molecules exhibit two reduction events near −2.0 V *vs.* Fc/Fc^+^ and are accessed through a modular synthetic route that enables the analysis of structure–function relationships. The original report utilized a combination of statistical modeling and mechanistic studies to identify a derivative with promising stability (Fig. 1). Despite this, early studies on anolyte solubility revealed that the neutral form exhibited a modest solubility (<0.2 M) in acetonitrile. This prompted us to investigate the structure–solubility relationship for 2,2′-bipyrimidines.^12,17,18^ Herein, we report the use of a single node decision tree and a heatmap to facilitate the design of an analogue with improved solubility while maintaining good stability. The optimized molecule demonstrates stable cycling in a prototype organic redox flow battery with a cell potential up to 3 V.

## Results and discussion

**Fig. 2:**
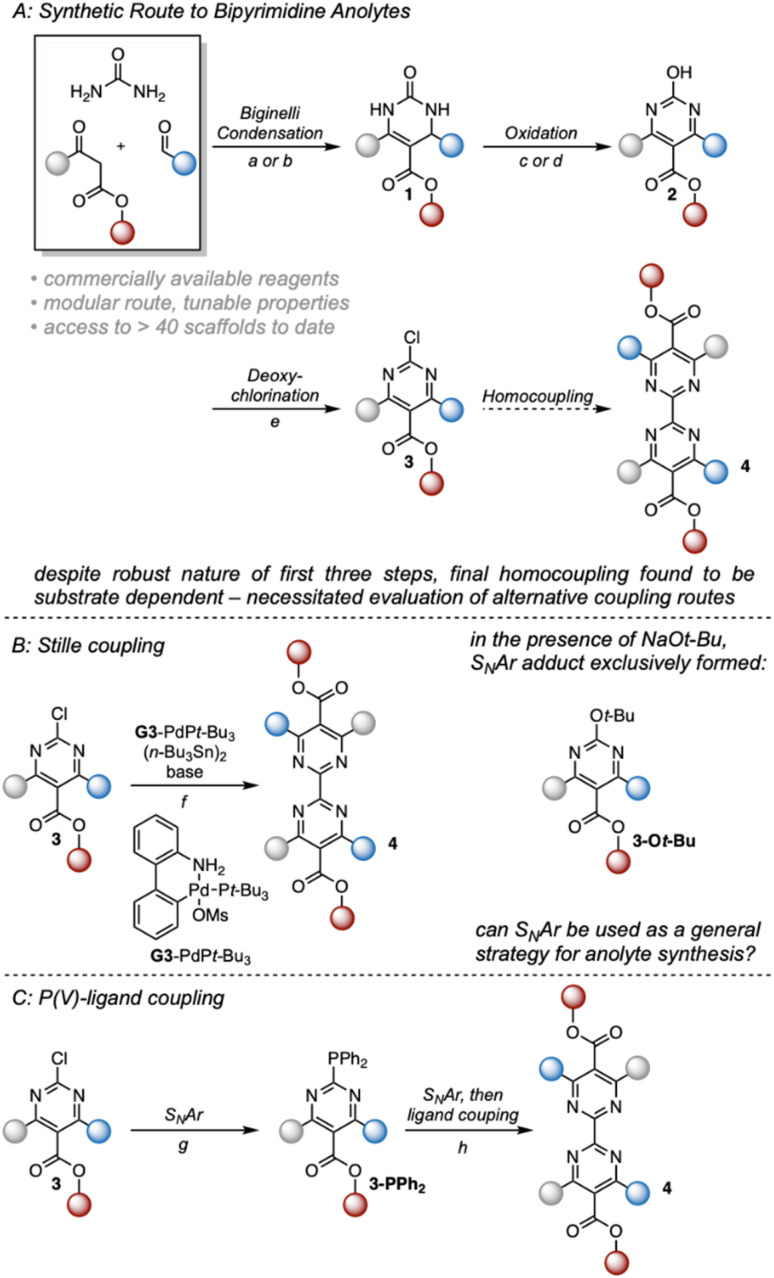
Synthetic route to 2,2′-bipyrimidines. (A) Synthetic strategy used to access 2,2′-bipyrimidines. For (a) and (b), solvent and ester identity were matched to avoid transesterification. (a) 1 mol% CuCl, 20 mol% H_2_SO_4_, ROH. (b) 0.6 eq. FeCl_3_·6H_2_O, cat. HCl, ROH. (c) 1.2 eq. K_2_S_2_O_8_, 3 : 2 MeCN : H_2_O, Δ. (d) 1 mol% CuCl_2_, 20 mol% K_2_CO_3_, 2.5 eq. *t*-BuOOH, DCM, Δ. (e) 0.5 eq. *N*,*N*-dimethylaniline, 8.5 eq. POCl_3_, 70 °C. (f) 1.2 mol% Buchwald G3-PdP*t*-Bu_3_, 0.5 eq. (*n*-Bu_3_Sn)_2_, 1.4 eq. base, PhMe, Δ. When using K_3_PO_4_ as base, no reaction was observed. (g) 1.2 eq. HPPh_2_, 2,2,2-trifluroethanol, 75 °C. (h) 1.2 eqn (2)-chloropyrimidine, 1.2 eq. HOTf, 2.2 eq. NaOTf, 10 eq. H_2_O, 2,2,2-trifluoroethanol, 75 °C. (B) Evaluation of a Stille coupling strategy. (C) P(v)-Ligand coupling strategy used to access 2nd-generation anolytes.

Our previous studies focused on understanding the structural features imparting stability to bipyrimidine anolytes.^17^ This was achieved by incorporating sterically small, minimally polarizing methyl groups at the 4- and 4′ positions (grey in Fig. 1 and 2). In concert, *ortho* and *meta* substituted aryl groups were placed at the 6- and 6′-positions (blue in Fig. 1 and 2). These substituted arenes can rotate out of plane and thus minimize steric interactions with the ester. Based on this 1st generation scaffold, we focused on modifying two sites focused to enhance solubility: (1) the aryl groups (blue) and (2) the substituents on the esters (red).

Our previous synthesis commenced with a Biginelli condensation of commercial building blocks, followed by oxidative dehydrogenation and deoxychlorination to form a 2-chloropyrimidine (Fig. 2).^17^ These first three steps can be readily performed on ≥100 mmol scale. However, the next step, a homocoupling of these 2-chloropyrimidine building blocks, was found to give poor yields that are highly dependent on the nature of the substrate. Therefore, to enable the synthesis of a wider range of new compounds for structure–solubility relationship studies, we investigated alternative synthetic approaches to form the bipyrimidine core. Through several model studies, we identified a nucleophilic aromatic substitution strategy based upon recent reports by the McNally group using P(v)-ligand coupling.^19,20^ This process operates *via* sequential nucleophilic aromatic substitution and apicophilic ligand coupling,^20^ necessitating the use of inherently electrophilic substrates like 2-chloropyrimidines. Gratifyingly, a slightly modified protocol proved to be a general method for the synthesis of the desired bipyrimidine products.

**Fig. 3:**
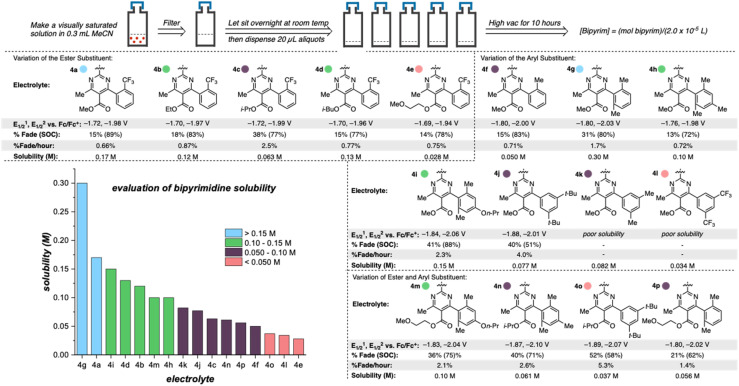
Library of 2,2′-bipyrimidines. Solubilities were measured according to the method outlined at the top of the figure and reduction potentials internally referenced *vs.* Fc/Fc^+^. A reported H-cell cycling assay was used to measure the percent capacity fade through the course of 50 cycles, state of charge (SOC; reported value is the highest measured), and percent fade per hour (total capacity fade per total experiment time).

Once library synthesis was completed, each 2,2′-bipyrimidine was characterized with respect to reduction potential, stability (measured by the temporal rate of fade during H-cell cycling; see ESI† for details), and solubility. Solubility measurements were performed according to a previously reported procedure.^9,12^ The values reported in Fig. 3 represent the mean of ≥3 trials conducted on each anolyte. Anolyte 4a was the most stable analog identified in the first generation but exhibited only modest solublity (0.17 M in neat acetonitrile) in the neutral form. However, in the presence of tetra-*n*-butylammonium (TBA) counterions, the doubly reduced state was found to be a viscous oil that was miscible with acetonitrile (see ESI†). These results indicate that the neutral bipyrimidine is the solubility-limiting form; as such, all solubility measurements going forward were performed on the neutral structures. Cyclic voltammetry (CV, see ESI† for details) showed that each novel candidate (except for 4k and 4l due to their poor solubility in supporting electrolyte solution) undergoes reversible electron transfer in a diffusion-controlled manner.

**Fig. 4:**
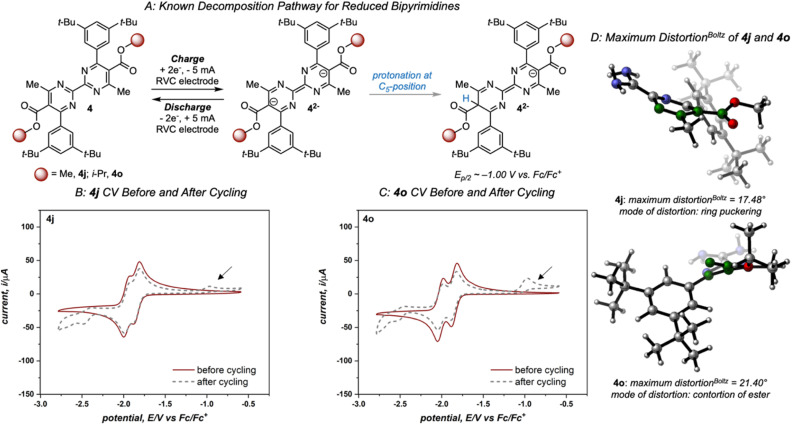
(A) Known decomposition pathway of reduced bipyrimidine anolytes. (B) and (C) Cyclic voltammograms of 2nd-generation candidates 4j and 4o, respectively, recorded before and after cycling at 100 mV s^−1^ and an initial negative scan direction. The arrow indicates the diagnostic oxidation peak of the protonated bipyrimidine. (D) DFT computed structures of 4j and 4o with the maximum mode of distortion for each molecule (relevant atoms highlighted in green). While each mode serves to alleviate steric strain present in the reduced state, 4j exhibited a maximum distortion *via* ring puckering while 4o demonstrated torsion of the ester out of the plane of the pyrimidine core.

Variation of the ester led to minimal change in the electrochemistry of 4a–4e (Fig. 3), while variation of the aryl group had a more pronounced impact on both reduction potential and stability as observed in the first-generation library.^17^ The range of anolyte stability (0.66% fade per hour to 5.3% fade per hour) was relatively narrow, consistent with the 2nd generation library design principles. For the candidates with the highest rates of fade (4j: 4.0% fade per hour and 4o: 5.3% fade per hour, respectively), an irreversible peak corresponding to the oxidation of the protonated bipyrimidine (Fig. 4) was observed. This is consistent with the previously reported decomposition pathway, which is promoted by distortion of the reduced anolyte, either through ring puckering or rotation of the ester from the bipyrimidine plane.^17^ Comparing 4o and 4j, 4o has a greater distortion relative to 4j by ∼4°, leading to a greater amount of the protonated decomposition product (Fig. 4D). Accordingly, we hypothesized that the stability profiles of the 1st and 2nd generation anolyte libraries could be analyzed simultaneously.

**Fig. 5:**
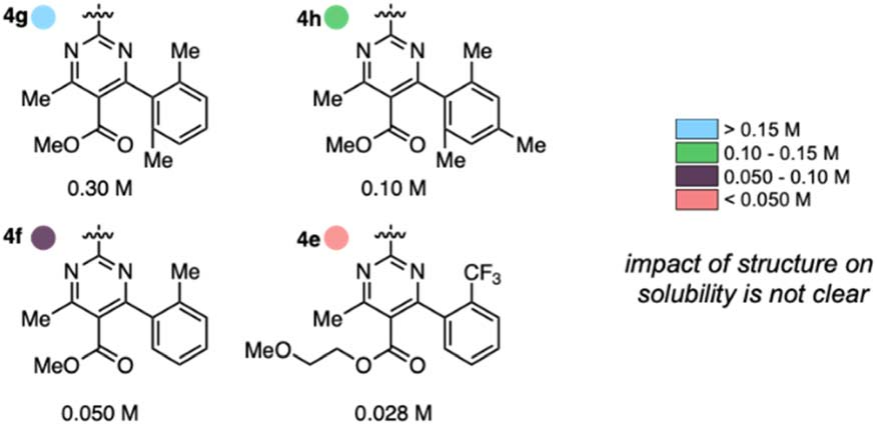
Impact of anolyte structure on solubility with representative structures of each solubility class.

Anolyte solubility was markedly impacted by ester identity, with an approximately six-fold *decrease* in solubility as the ester is changed from methyl (4a in Figure 3) to 2-methoxyethyl (4e in Fig. 3). This stands in stark contrast to the reported ability of alkoxyether substituents to enhance solubility in other systems.^21^ The impact of the aryl substituent was less clear: changing from an *ortho*-methyl substituted phenyl (4f) ring to a 2,6-dimethyl substituted phenyl ring (4g) led to a six-fold increase in solubility (Fig. 5). However, no clear trend was evident, and the most soluble candidate, 4g at 0.3 M (0.6 M e^−^), fell short of the desired target of 0.5 M (1 M e^−^). Notably, only 3 out of 16 2nd generation candidates had solubilities of ≥0.15 M. This provided the impetus to employ statistical modeling techniques capable of deconvoluting the impact of structure on solubility (*vide infra*).

Previously we found that computational analysis of these systems was expedited by using a truncated monoanionic surrogate of the bipyrimidine scaffold, as this strategy greatly reduced the number of accessible conformers.^17^ Similarly, we opted to compute a truncated version of the neutral (for solubility modeling) and reduced (monoanionic form for stability modeling) bipyrimidines for statistical modeling efforts (see ESI† for details). Conformational searches were performed with a 5 kcal mol^−1^ energetic window and the OPLS4 force field.^22^ The ensuing conformational ensembles were then subjected to geometry optimization with DFT using the M06-2x/6-31+G(d,p) level of theory and the implicit conductor-like polarizable continuum model (CPCM) in acetonitrile.^23^ Following single point energy calculations with M06-2x/def-TZVP, molecular descriptors such as Sterimol values, partial charges (assessed by natural bond orbital (NBO) calculations), and surface area measurements, were collected for each conformer. In addition to extracting the descriptor values for the lowest energy conformer, the Boltzmann-weighted average and minimum/maximum values of each descriptor were collected to account for anolyte flexibility. In addition to these DFT descriptors, descriptors from Schrödinger's QikProp library were also collected.

Following descriptor calculation, we next evaluated the data distribution to determine which statistical modeling technique was best suited for solubility data analysis. Traditionally, linear regression has been used to correlate structure to function, but these methods failed in this study, likely due to poor data distribution.^24^ This could be due to a poor representation of anolytes with solubility >0.15 M. To expand the distribution of data, multiple 1st-generation bipyrimidines were included in solubility statistical modeling efforts, even though their stability profile may have been undesirable. Additionally, this type of data distribution prompted the use of a classification algorithm whereby “good” *versus* “poor” measured solubilities can be binned using an algorithm-identified molecular descriptor and a user-defined threshold. This process, initially reported within the context of catalyst speciation in cross-coupling reactions,^25^ has been integrated as a standard part of our statistical modeling workflow.^26^

**Fig. 6:**
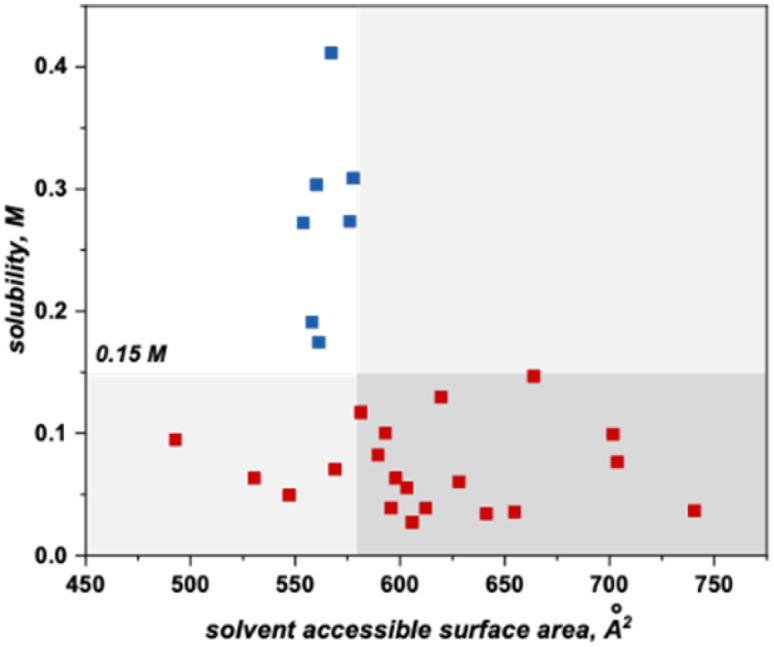
Threshold of 579 Å^2^ SASA based on a user-defined cutoff of 0.15 M.

This algorithm identified a solvent accessible surface area (SASA) descriptor to classify bipyrimidine solubility. Applying a user defined cutoff of 0.15 M, the single node decision tree (Fig. 6) revealed that anolytes with average solvent accessible surface area SASA, derived by moving a 1.4 Å probe over the enclosed molecular surface^27^ values of <579 Å^2^ exhibit reasonable solubilities (≥0.15 M; blue squares in upper left quadrant), while anolytes with a SASA >579 Å^2^ have poor solubilities (≤0.15 M, red squares in bottom right quadrant).

Within this classification, there are four false positives that represent molecules that the model identifies as soluble but have measured solubilities below the 0.15 M cut-off. Although the exact molecular feature(s) that contribute to the decreased solubilities of these four candidates is not clear, the model directs us to a specific property region to target for improved solubility.

Since anolyte stability is also a crucial metric, we sought to combine both the solubility and stability profiles of the 1st generation and 2nd generation libraries in a virtual screening workflow. Previously, linear regression was used to correlate the experimental rate of fade to two molecular descriptors: the Boltzmann averaged maximum distortion (either ring puckering or contortion of the ester out of the bipyrimidine plane) and the Boltzmann-averaged NBO charge at both C_4_/C_6_ (corresponding to the polarization of the C=N double bonds in the bipyrimidine). A heatmap that plots these two descriptors for all 1st and 2nd-generation anolytes was constructed, with points colored according to their rates of fade. Similar to the solubility threshold, the stability heatmap identifies a region of chemical space to target for design of stable candidates: bipyrimidines with maximum distortion values less than approximately 15° or NBO values less than 0.30 are envisioned to possess favorable stability.

**Fig. 7:**
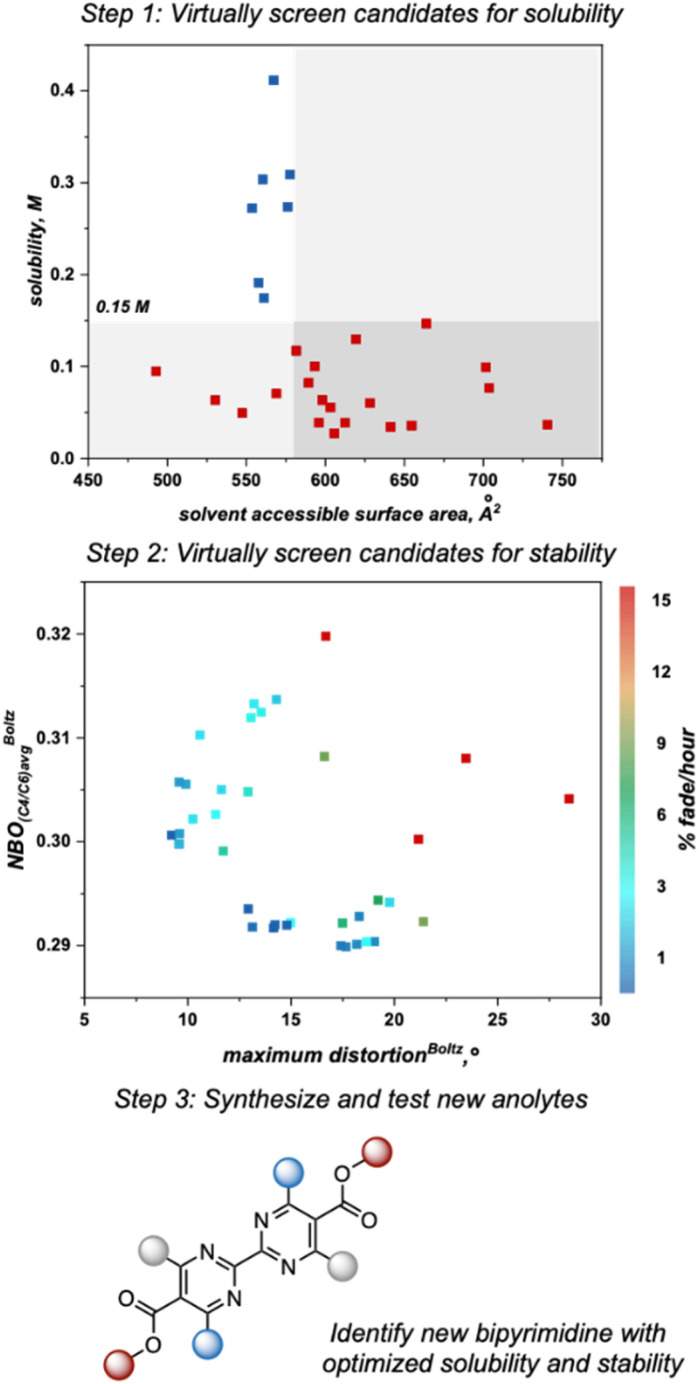
Workflow utilized to virtually screen new anolytes with respect to solubility and stability.

**Fig. 8:**
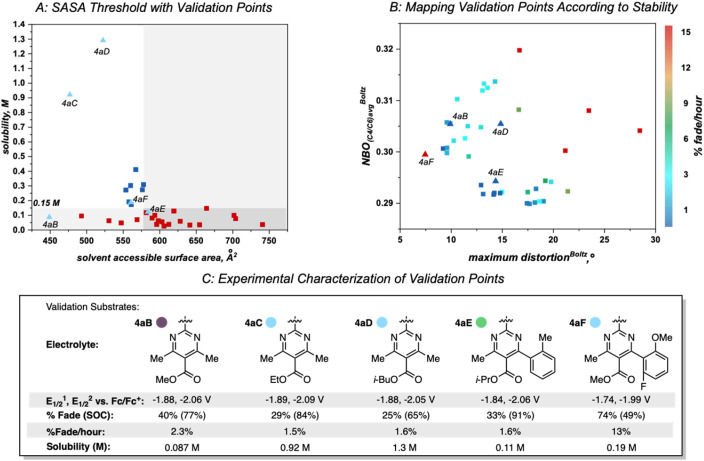
(A). Threshold at 579 Å^2^ SASA with validation points (labeled and shown by light blue triangles) plotted by their SASA value. (B) Heat map of the molecular descriptors dictating stability. Blue points indicate highly stable anolytes that have low rates of fade while red points indicate unstable molecules that have high rates of fade. Note that 4aC was characterized with respect to stability in the first paper and not re-evaluated in the heatmap describing anolyte stability. (C) Characterization of validation substrates. Reduction potentials internally referenced *vs.* Fc/Fc^+^. A reported H-cell cycling assay was used to measure the percent capacity fade through the course of 50 cycles, state of charge (SOC; reported value is the highest measured), and percent fade per hour (total capacity fade per total experiment time).

As the next step, we utilized the solubility threshold and stability heatmap for a data-guided design of bipyrimidine candidates that meet or surpass the target solubility and de-risk future anolyte synthesis. Anolyte candidates were virtually screened according to a two-step workflow utilizing the SASA threshold for solubility and the heatmap describing anolyte stability as a function of the previously reported molecular descriptors (Fig. 7). Advantageously, the SASA term dictating solubility and the maximum distortion term describing stability are complementary to one another, in that smaller anolytes could potentially be soluble while also minimizing steric interactions that induce decomposition. However, since structural variations designed to minimize the maximum distortion descriptor could also impact the average NBO charge at C_4_/C_6_, it can be difficult to design candidates that optimize both features simultaneously. Thus, candidates could be selected to optimize one of these features or to balance the two descriptors (Fig. 8).

A set of possible bipyrimidines was designed based on the computed SASA values (for solubility) and their positions in the heatmap describing anolyte stability. Notably, three candidates within the virtual screening process contain a tetramethyl substitution pattern. Despite the fact that this could lead to increased NBO charges at the C_4_/C_6_ position (and potentially a slightly less stable anolyte),^17^ this substitution pattern was pursued based on initial investigation of the SASA of 4aC, along with two other analogs bearing tetramethyl substitution, 4aB and 4aD (Fig. 8A). Candidates with desirable SASA values along with minimized distortion (4aB, 4aD, 4aE, 4aF), minimized NBO charge (4aE, 4aF), or minimization of both stability descriptors (4aE, 4aF) were selected for synthesis (Fig. 8B).

As before, experimental results demonstrated that ester identity had a substantial impact on solubility, represented by an order of magnitude increase in solubility when the methyl ester in 4aB is changed to an ethyl ester in 4aC. Furthermore, 4aD was isolated as a viscous, miscible liquid that slowly solidified over a matter of weeks and was later found to have a solubility of ∼1.3 M (Fig. 8C). This constitutes an ∼50-fold increase in solubility of relative to 4e. Accompanying this increase in solubility was a slight decrease in stability (1.6% fade per hour relative to the most stable anolyte 4a with 0.66% fade per hour). This was not unexpected due the increased average NBO charge induced by the tetra-methyl substitution pattern. Interestingly, 4aF was highly unstable (13% fade per hour) despite the minimal distortion and NBO charge. This could be indicative of a yet unidentified mechanism of decomposition for bipyrimidines possessing a fluorinated aryl substituent. With the identification of 4aD as a highly soluble candidate with good stability, we next sought to evaluate its performance in a flow battery.

To fully utilize the two highly energetic reduction events of 4aD it was necessary to identify a compatible catholyte that undergoes multiple oxidation events at high potentials. Preliminary compatibility tests of catholytes (see ESI† for details) identified diaminocyclopropenium-phenothiazine 4-DMPP as a suitable species, with two high-energy oxidation events (0.64 V and 1.00 V *vs.* Fc/Fc^+^)^10*e*^ and reasonable compatibility (Fig. S3†).

**Fig. 9:**
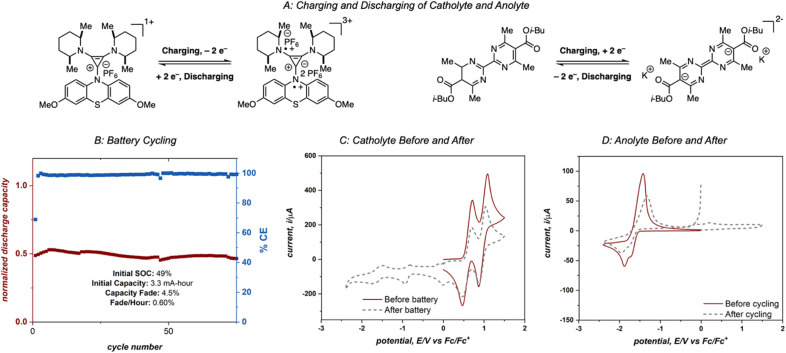
(A) Electrolyte charging and discharging processes. (B) Flow cell cycling of 25 mM 4aD and 4-DMPP. (C) Cyclic voltammograms of catholyte recorded before and after cycling at 100 mV s^−1^ and an initial positive scanning direction. (D) Cyclic voltammograms of anolyte recorded before and after cycling at 100 mV s^−1^ and an initial negative canning direction.

Electrolytes 4aD and 4-DMPP were cycled in an asymmetric redox flow battery using a Fumasep FAP-375-PP separator and 25 mM solutions of 4aD and 4-DMPP in 0.5 M potassium hexafluorophosphate/acetonitrile (Fig. 9A). The solutions were flowed through the cell at 10 mL min^−1^ and were electrochemically cycled at 15 mA cm^−2^ with a cell potential up to 3 V, one of the highest cell potentials demonstrated in a non-aqueous two-electron redox flow battery.^28^ This system achieved 49% material utilization on the first cycle and showed >95% capacity retention over 75 cycles with an average power density of 0.74 W h L^−1^ power density (1.55 W h L^−1^ theoretical power density).^29^ However, catastrophic failure of the system was observed after these initial 75 cycles (see ESI†). Cyclic voltammetry analysis of the anolyte and catholyte solutions after 180 cycles revealed that crossover of the anolyte as well as molecular decomposition of both compounds contributed to the loss of capacity and system failure (Fig. 9C and D), ultimately disqualifying this system from operating in a high-concentration battery. However, this experiment demonstrates the cycling capability of 4aD in a stable, high energy flow battery.

## Conclusions

In summary, we report herein the design of a library of 16 new 2,2′-bipyrimidine anolytes with the intent of analyzing the impact of structure on solubility. Additionally, the design of this 2nd-generation library was carried out with respect for the structural features responsible for stable bipyrimidines. Aiming for a target concentration of 0.5 M (1 M e^−^ since the bipyrimidine can store two-electrons per molecule) to avoid mass transfer limitations, a narrow range of solubilities was observed within the initial library. This prompted the inclusion of multiple 1st generation bipyrimidines (independent of the corresponding stability) to improve dataset diversity. A non-linear threshold relationship between the solvent accessible surface area and solubility was identified, and when paired with a heat map describing anolyte stability with previously identified molecular descriptors, five new anolyte candidates were designed in a virtual screening campaign and experimentally tested. One candidate was identified to have a 46-fold increase in solubility (relative to the least soluble anolyte) of 1.3 M while minimally sacrificing stability. Pairing this compound with a diaminocyclopropenium-phenothiazine derivative accessed a proof-of-principle organic redox flow battery with a cell potential up to 3 V. Although relatively stable cycling was achieved for the first 75 cycles, drastic battery failure was observed beyond 75 cycles. Cyclic voltammograms after cycling suggest that electrolyte decomposition, potentially due to anolyte crossover, was a primary mechanism of failure. Going forward, evolution of the bipyrimidine scaffold will focus on maintaining the gains made in stability and solubility while also optimizing other battery relevant properties, such as crossover.

## Data availability

The ESI† is freely available at https://doi.org/10.1039/d3sc04084d and contains the following for this article: experimental procedures for all reactions and electrochemical measurements; spectroscopic characterization of new compounds; detailed computational methods and model evaluation; copes of ^1^H, ^13^C, and ^19^F NMR spectra.

## Author contributions

A. R. P. and M. S. S. conceptualized the project. A. R. P. and S. L. M. performed experimental investigations. A. R. P., S. L. M, and S. L. G. carried out modeling studies. R. W. K. and B. J. prepared and operated the flow battery. All authors contributed to writing and editing the manuscript. M. S. S. and M. S. S. acquired funding for the project.

## Conflicts of interest

There are no conflicts to declare.

## Supplementary Material

SC-014-D3SC04084D-s001

SC-014-D3SC04084D-s002

## References

[cit1] IRENA , World Energy Transitions Outlook 2022: 1.5 °C Pathway, International Renewable Energy Agency, Abu Dhabi, 2022

[cit2] Yang Z., Zhang J., Kintner-Meyer M. C. W., Lu X., Choi D., Lemmon J. P., Liu J. (2011). Chem. Rev..

[cit3] Luo J., Hu B., Hu M., Zhao Y., Liu T. L. (2019). ACS Energy Lett..

[cit4] Kwabi D. G., Ji Y., Aziz M. J. (2020). Chem. Rev..

[cit5] Winsberg J., Hagemann T., Janoschka T., Hager M. D., Schubert U. S. (2017). Angew Chem. Int. Ed. Engl..

[cit6] Gong K., Fang Q., Gu S., Li S. F. Y., Yan Y. (2015). Energy Environ. Sci..

[cit7] Bard A. J. and Faulkner L. R., Electrochemical Methods: Fundamentals and Applications, John Wiley & Sons, Inc., Hoboken, New Jersey, United States of America, 2nd edn, 2001

[cit8] Hendriks K. H., Sevov C. S., Cook M. E., Sanford M. S. (2017). ACS Energy Lett..

[cit9] Sevov C. S., Samaroo S. K., Sanford M. S. (2017). Adv. Energy Mater..

[cit10] Zhang J., Yang Z., Shkrob I. A., Assary R. S., Tung S. O., Silcox B., Duan W., Zhang J., Su C. C., Hu B., Pan B., Liao C., Zhang Z., Wang W., Curtiss L. A., Thompson L. T., Wei X., Zhang L. (2017). Adv. Energy Mater..

[cit11] Wang X., Gautam R. K., Jiang J. J. (2022). Batteries Supercaps.

[cit12] Robinson S. G., Yan Y., Hendriks K. H., Sanford M. S., Sigman M. S. (2019). J. Am. Chem. Soc..

[cit13] Vermeire F. H., Chung Y., Green W. H. (2022). J. Am. Chem. Soc..

[cit14] Wong C. M., Sevov C. S. (2021). ACS Energy Lett..

[cit15] Zhang J., Corman R. E., Schuh J. K., Ewoldt R. H., Shkrob I. A., Zhang L. (2018). J. Phys. Chem. C.

[cit16] Zhang L., Feng R., Wang W., Yu G. (2022). Nat. Rev. Chem.

[cit17] Griffin J. D., Pancoast A. R., Sigman M. S. (2021). J. Am. Chem. Soc..

[cit18] Sevov C. S., Hickey D. P., Cook M. E., Robinson S. G., Barnett S., Minteer S. D., Sigman M. S., Sanford M. S. (2017). J. Am. Chem. Soc..

[cit19] Boyle B. T., Hilton M. C., McNally A. (2019). J. Am. Chem. Soc..

[cit20] Hilton M. C., Zhang X., Boyle B. T., Alegre-Requena J. V., Paton R. S., McNally A. (2018). Science.

[cit21] Milshtein J. D., Kaur A. P., Casselman M. D., Kowalski J. A., Modekrutti S., Zhang P. L., Attanayake N. H., Elliott C. F., Parkin S. R., Risko C., Brushett F. R., Odom S. A. (2016). Energy Environ. Sci..

[cit22] Lu C., Wu C., Ghoreishi D., Chen W., Wang L., Damm W., Ross G. A., Dahlgren M. K., Russell E., Von Bargen C. D., Abel R., Friesner R. A., Harder E. D. (2021). J. Chem. Theory Comput..

[cit23] Cossi M., Rega N., Scalmani G., Barone V. (2003). J. Comput. Chem..

[cit24] Lustosa D. M., Milo A. (2022). ACS Catal..

[cit25] Newman-Stonebraker S. H., Smith S. R., Borowski J. E., Peters E., Gensch T., Johnson H. C., Sigman M. S., Doyle A. G. (2021). Science.

[cit26] Liles J. P., Rouget-Virbel C., Wahlman J. L. H., Rahimoff R., Crawford J. M., Medlin A., O'Connor V. S., Li J., Roytman V. A., Toste F. D., Sigman M. S. (2023). Chem.

[cit27] Jorgensen W. L., Duffy E. M. (2002). Adv. Drug Delivery Rev..

[a] Yan Y., Robinson S. G., Sigman M. S., Sanford M. S. (2019). J. Am. Chem. Soc..

[cit29] Pahari S. K., Gokoglan T. C., Visayas B. R. B., Woehl J., Golen J. A., Howland R., Mayes M. L., Agar E., Cappillino P. J. (2021). RSC Adv..

