# Supplementary material for: Data science enabled discovery of a highly soluble 2,2′-bipyrimidine anolyte for application in a flow battery
Source: Chem Sci. 2023 Nov 2;14(47):13734–42. doi: 10.1039/d3sc04084d (PMC10699568; doi:10.1039/d3sc04084d)
Supplement: SC-014-D3SC04084D-s001 [file SC-014-D3SC04084D-s001.pdf]

Electronic Supplementary Material (ESI) for Chemical Science.
This journal is © The Royal Society of Chemistry 2023

Supporting Information For:

Data Science Enabled Discovery of a Highly Soluble 2,2´-Bipyrimidine Anolyte for Application in a Flow Battery

Adam R. Pancoast, Sara L. McCormack, Shelby L. Galinat, Ryan Walser-Kuntz,
*Brianna M. Jett, Melanie S. Sanford, and Matthew S. Sigman**
Department of Chemistry, University of Utah, 315 South 1400 East, Salt Lake City, UT 84112, USA
Joint Center for Energy Storage Research (JCESR) 9700 S Cass Avenue, Argonne, IL 60439, USA

Table of Contents:

i. General Considerations: 2

Ii. Synthesis Of Anolytes: 3

Iii. Electrochemical Experiments And Characterization: 6

Iv. Solubility Measurements: 25

V. Computational Methods: 27

Vi. Characterization Of Compounds: 33

Vii. References: 80

Viii. ^{1}H, ^{13}C, and ^{19}FNmr Spectra: 82

I. General Considerations:

All reactions were performed using oven-dried or flame-dried glassware equipped with a magnetic stir bar, under an atmosphere of nitrogen unless otherwise noted. All reagents were purchased from commercial suppliers and used without further purification unless otherwise noted. *N,N*-dimethyl formamide (DMF), dichloromethane (DCM), and tetrahydrofuran (THF) were dried by passing through a column of activated alumina immediately prior to use. ^{1}H NMR spectra were obtained in $CDCl_3$ or DMSO-d_6 at 500 MHz. Chemical shifts are reported in ppm and referenced to either the $CHCl_3$ singlet at 7.26 ppm or the DMSO-d_6 triplet at 2.50 ppm. ^{13}C NMR spectra were obtained in $CDCl_3$ or DMSO-d_6 at125 MHz and referenced to the center peak of the $CDCl_3$ triplet at 77.16 ppm or the center peak of the DMSO-d_6 septet at 39.52 ppm. ^{19}F NMR [^{1}H-decoupled] spectra were obtained in $CDCl_3$ or DMSO-d_6 at 282 MHz. The abbreviations s, d, t, quint, h, hept, dd, ddd, dt, tt, and m stand for the resonance multiplicities singlet, doublet, triplet, quintet, hextet, heptet, doublet of doublets, doublet of doublet of doublets, doublet of triplets, triplet of triplets, and multiplet, respectively. Thin-layer chromatography (TLC) was performed with Silicycle glass backed (extra hard layer) 60A F_{254} silica gel plates eluting with solvents indicated, visualized by a 254 nm UV lamp or by staining with basic $KMnO_4$ or Vanillin. Flash chromatography was performed using Silicycle SiliaFlash® F60 (230-400 mesh) silica. IR spectra were recorded using a Thermo Scientific Nicolet is50 FT-IR. HRMS data were obtained on a Waters LCP Premier XE instrument by ESI/TOF.

II. Synthesis of Anolytes:

Multi-Component Biginelli Coupling (General Procedure A):

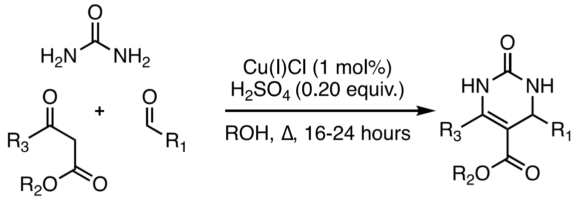

1

All compounds were synthesized via an adapted literature procedure.[1] In the case of aliphatic aldehydes, these were distilled before use. A typical procedure is described below:

To a round bottom flask equipped with a stir bar was added urea (1.75 equiv.) and alcoholic solvent (1 M). The ensuing solution (or in some cases, a suspension) was added aldehyde (1.0 equiv.), ketoester (1.0 equiv.), Cu(I)Cl (0.010 equiv.), and concentrated sulfuric acid (18.4 M, 0.20 equiv.). The mixture was heated to reflux (see note about higher boiling solvents below) and stirred vigorously for 16-24 hours. Once the allotted time had passed, the flask was removed from heat and allowed to cool to room temperature while maintaining stirring of the flasks' contents. The product often precipitated out of solution during the reaction or cooling process (for certain products, no precipitation was observed – these will be specifically addressed below). The solid was filtered over a fine to coarse glass frit and washed once with a minimal amount of MeOH and again with a minimal amount of H_2O. After drying the product on the frit, the dihydropyrimidinone was carried forward to the next step without further purification, even in the case of products contaminated with minor impurities (explicit cases will be noted below).

***Note:** as previously reported, the alcoholic solvents were chosen based on the identity of the ester to avoid transesterification. When using higher boiling solvents, e.g., 2-methoxyethanol, the reaction was heated to 90°C.*

Oxidative Aromatization (General Procedure B):

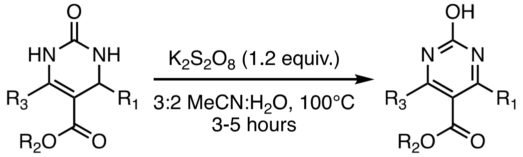

1 **2**

All compounds (except **1aB**, **1aC**, and **1aD**) were synthesized according to a modified literature procedure for sterically hindered substrates.[2] Below, a general protocol is described:

A round bottom flask was charged with a stir bar, dihydropyrimidinone (1.0 equiv.), followed by the addition of 3:2 MeCN:H_2O (0.2 M). The suspension was stirred gently while adding $K_2S_2O_8$ (1.2 equiv.) in one portion at room-temperature. The heterogeneous mixture was stirred vigorously and heated to reflux (100°C) for 3-5 hours; during the heating process, it was observed that the white heterogeneous mixture typically underwent multiple color changes until settling on a deep red or brown color that persisted throughout the rest of the reaction. After 3-5 hours, 0.5 M $Na_2S_2O_3$ was added to quench any peroxides

(evaluated with peroxide test strips). The MeCN was removed under reduced pressure to yield an aqueous suspension; this was extracted three times with DCM. The organic layers were combined, dried over Na_2SO_4, filtered, and concentrated under reduced pressure. In most cases, a sticky deep red residue remained in the flask after concentration – this could be triturated with EtOAc:Hexanes to yield the desired 2-hydroxypyrimidine. The triturated material was filtered on a fine to coarse glass frit and dried for several hours to remove solvent. In some cases, certain 2-hydroxypyrimidines proved resistant to trituration and/or were carried forward with minor impurities; this resulted in lower yields of the deoxychlorinated product and will be described when appropriate.

Oxidative Aromatization (General Procedure C):

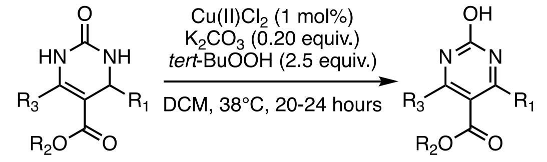

1 **2**

For **1aB**, **1aC**, and **1aD**, a modified literature procedure[1] was used and is described as follows:

A multi-neck round bottom flask containing a stir bar was equipped with a pressure equalizing addition funnel, condenser, and glass stoppers. Dihydropyrimidinone (1.0 equiv.), Cu(II)Cl_2 (0.010 equiv.), and K_2CO_3 (0.20 equiv.) were added to the flask. Stabilizer free DCM (0.34 M) was added to the flask to yield a white to pale yellow suspension. While heating, *tert*-butyl hydroperoxide (2.5 equiv., 70% aqueous solution) was added dropwise through the addition funnel over 10-20 minutes. After the peroxide had been fully added, the biphasic mixture was stirred forcefully while refluxing for 20-24 hours. The reaction was cooled to room temperature and quenched by adding an aqueous solution containing 0.5 M $Na_2S_2O_3$ and 25% (*w*/*w*) NH_4Cl via the addition funnel. The flask contents were stirred for 1 hour at room temperature and checked to ensure no peroxides remained. The contents were poured into a separatory funnel and extracted three to five times with DCM until the extracts were colorless. The organic layers were dried over Na_2SO_4, filtered, and concentrated under reduced pressure until most of the DCM had been removed. At this point, heptane was added to the flask and immediate precipitation was observed. This solid was filtered and dried on a fine to coarse glass frit for several hours to remove excess solvent. No further purification was needed.

Deoxychlorination of 2-hydroxypyrimidines (General Procedure D):

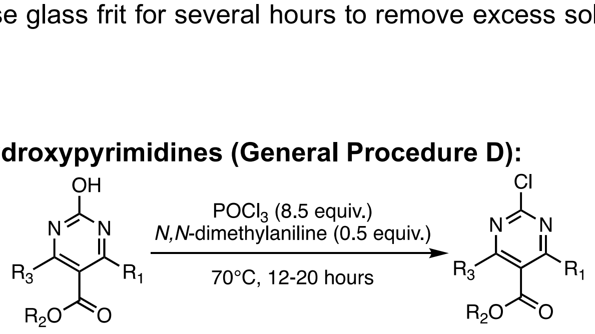

2 **3**

All compounds were synthesized via an adapted literature procedure;

In this work, short path distillation of the $POCl_3$ (Alfa Aesar 98%) immediately prior to use was essential to

obtaining significant amounts of product. A typical protocol is defined below:

A round bottom flask containing a stir bar was charged with 2-hydroxypyrimidine (1.0 equiv.), freshly distilled $POCl_3$ (8.5 equiv.), and then *N,N*-dimethylaniline (0.50 equiv.). The reaction mixture was stirred vigorously while heating to 70 °C to yield a deep red/brown homogeneous solution in most cases. After 12-20 hours, the reaction was checked for conversion by ^{1}H-NMR. Once complete, the reaction was cooled to room temperature and slowly poured into a flask containing a bed of crushed ice. *Quenching $POCl_3$ is a highly exothermic process – care should be taken to swirl the flask and ensure a thorough quench with ice before adding water or proceeding with the workup.* After quenching excess $POCl_3$, the contents were poured into a separatory funnel. Three to five extractions with DCM were performed (till extracts were clear) and the organic layers were combined, dried over Na_2SO_4, filtered, and concentrated to yield a viscous dark colored oil in most cases. The oil was purified via flash column chromatography to yield the 2-chloropyrimidine.

P(V)-Ligand Coupling of 2-chloropyrimidines (General Procedure E):

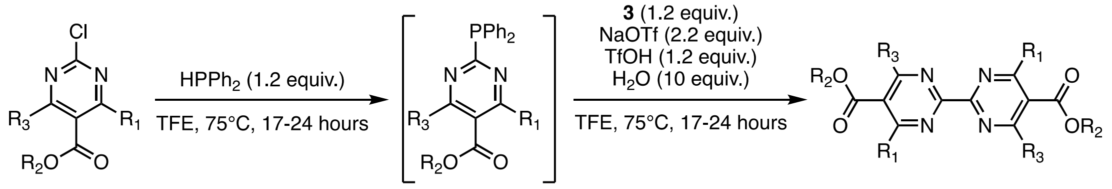

All 2,2'-bipyrimidines (except **4q – 4aA**) were synthesized via a modified literature procedure for P(V) ligand coupling of chloroazines.[3] Diphenylphosphine ($HPPh_2$, Alfa Aesar 99%) was stored in a nitrogen filled glovebox. For each reaction, aliquots were removed from the box, with care taken to leave N_2 headspace in the syringe, and immediately charged into the reaction vessel. A typical procedure for a 4 mmol (with respect to the 2-chloropyrimidine) scale reaction is described below:

Step 1: A 4-dram vial containing a stir bar was charged with 2-chloropyrimidine (1.0 equiv.) while hot and then subjected to three evacuation/backfill (N_2) cycles. After the third cycle, 2,2,2-trifluoroethanol (TFE, 0.4 M with respect to 2-chloropyrimidine) was added to form a heterogeneous solution that was vigorously stirred. $HPPh_2$ (1.2 equiv.) was added in one portion, following which the vial was capped and sealed with electrical tape. The vial was placed in an aluminum heating block set to 75°C and stirred for 17-24 hours. Upon reaching the set temperature, the reaction mixture often became homogeneous and brightly colored.

After verifying completion via TLC or ^{1}H-NMR, the reaction was cooled to room temperature and the vial contents were poured into a separatory funnel containing saturated aqueous sodium carbonate solution. Three extractions with DCM were performed and then the organic layers were combined and extracted twice with saturated brine solution. The organic layers were dried with Na_2SO_4, filtered, and the crude mixture was concentrated under reduced pressure. Post concentration, the viscous oil was immediately placed under an atmosphere of N_2 and an aliquot removed for ^{1}H-NMR analysis; in most cases,

high levels of conversion of the 2-chloropyrimidine to the 2-phosphinopyrimidine was observed with varying amounts of starting material and hydrodehalogenated starting material observed.

Step 2: To a hot 4-dram vial equipped with a stir bar was added 2-chloropyrimidine (1.2 equiv.) and NaOTf (2.2 equiv.). The vial was subjected to three evacuation/backfill (N_2) cycles. Meanwhile, TFE (8 mL total over three portions) was added to the flask containing the crude product from Step 1; following the addition of each portion of TFE, the flask was swirled to ensure dissolution and the contents were removed via syringe. The syringe was then used to charge the 4-dram vial with the 2-phosphinopyrimidine. After the third iteration, H_2O (10 equiv.) and TfOH (1.2 equiv.) were added to yield a brightly colored heterogeneous mixture. At this point, the vial was capped and sealed with electrical tape, heated to 75°C, and stirred vigorously for 17-24 hours. Reaction progress was checked via TLC or ^{1}H-NMR. These remained heterogeneous throughout the course of the reaction.

After cooling to room temperature, the reaction was worked up in the same manner as described in Step 1. The crude material was purified via flash column chromatography and recrystallized as described below before solubility measurements or electrochemical characterization.

***Note:** for larger scale reactions (> 4 mmol), a round bottom flask under an atmosphere of N_2 was used instead of a sealed 4-dram vial. Additionally, it was found that isolation and storage of the intermediate 2-phosphinopyrimidines under ambient conditions was accompanied by rapid formation of degradation products; therefore, these intermediates were carried forward immediately after the workup without further purification.*

III. Electrochemical Experiments and Characterization:

General: Anhydrous MeCN (99.8%), DMF (99.8%), and DMA (99.8%) were purchased from Sigma-Aldrich and stored inside a N_2 filled glovebox. Electrochemical grade (≥99.0%) tetrabutylammonium hexafluorophosphate ($TBAPF_6$) was purchased from Sigma-Aldrich. Each batch of $TBAPF_6$ was dried for three days at 80 ˚C under high vacuum prior to use. Generally, the electrolyte salts were free flowing after drying. If any larger clumps were observed, they were broken apart to help aid the drying process. Bipyrimidines **4a**, **4f**, **4h**, **4q** – **4aA**, and **4aC** were electrochemically characterized in a previous report.[2]

Cyclic Voltammetry:

Cyclic voltammetry (CV) for potential referencing against ferrocene and scan rate studies was performed in a N_2-filled glovebox and recorded with a CH Instruments 600E potentiostat using a three-electrode electrochemical cell. Cyclic voltammograms (CVs) recorded before/after H-cell cycling were recorded on a Pine Wavenow potentiostat/galvanostat (*vide infra*). A glassy carbon disk (0.071 cm^2, BASi®), Pt wire (Sigma-Aldrich), and $Ag/AgNO_3$ (10 mM with respect to $Ag/AgNO_3$ in 0.10 M $TBAPF_6$ in acetonitrile, BASi®) were used as the working, counter, and reference electrodes respectively. All CVs recorded for scan rate studies were conducted with 0.1 M $TBAPF_6$/acetonitrile stock solution while CVs taken during H-cell cycling

experiments were conducted with 0.5 M $TBAPF_6$/acetonitrile stock solution. CVs were referenced internally against Fc/Fc^+ (5 mM Fc in 0.1 M $TBAPF_6$/acetonitrile solution) to yield the reported redox potentials. Each anolyte was evaluated with respect to their diffusional properties by recording CVs at varying scan rates (10-500 mV/s). A plot of the cathodic and anodic peak currents against the square root of the scan rate yielded a linear relationship, indicative of a diffusion limited redox event.[4] The slope of each line was measured and combined with the Randles-Sevcik equation (eq. 1) was used to determine the diffusion coefficient.[5]

$$i_p = 0.4463(F^3/RT)^{0.5}\, n^{1.5} A D_0^{0.5} C_0^* v^{0.5} \qquad \text{Eq. 1}$$

Cyclic Voltammograms Referenced versus Ferrocene:

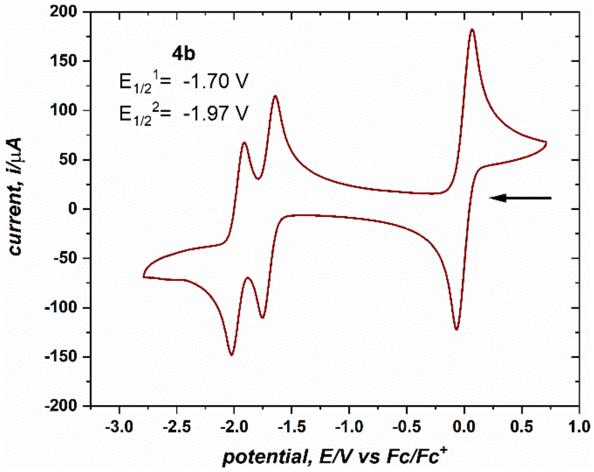

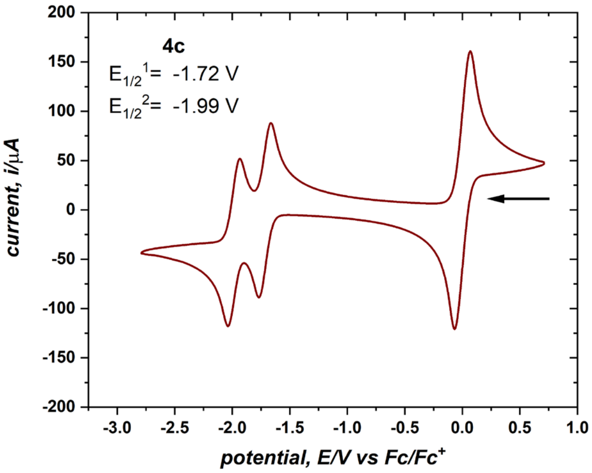

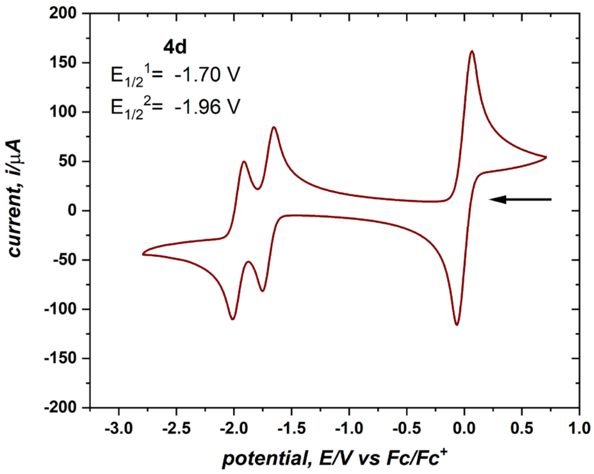

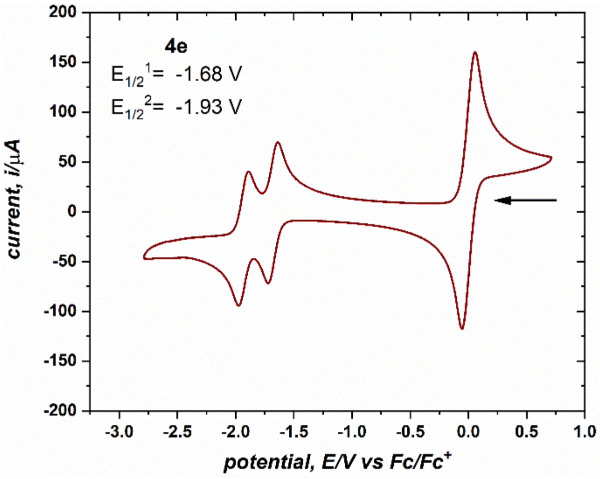

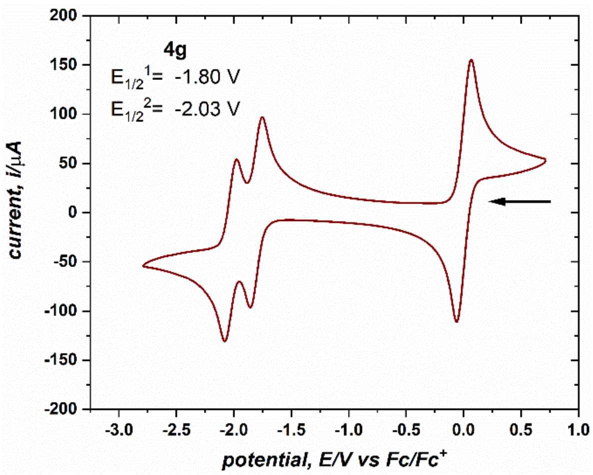

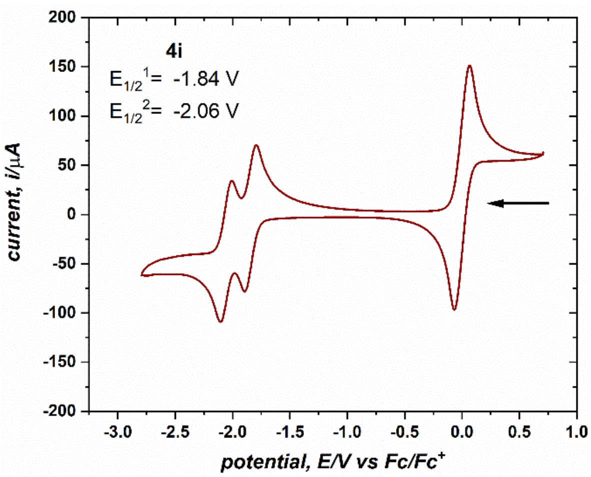

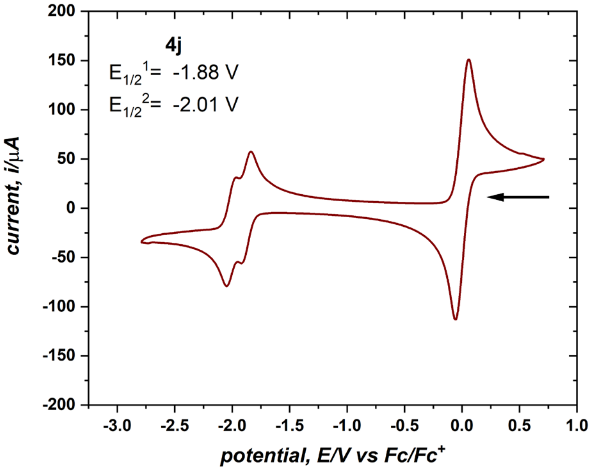
4j
$E_{1/2}^1$= -1.88 V
$E_{1/2}^2$= -2.01 V
current, i/μA
potential, E/V vs Fc/Fc+

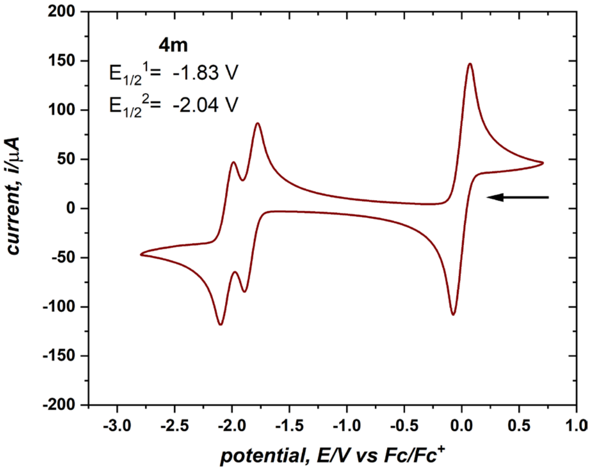
4m
$E_{1/2}^1$= -1.83 V
$E_{1/2}^2$= -2.04 V
current, i/μA
potential, E/V vs Fc/Fc+

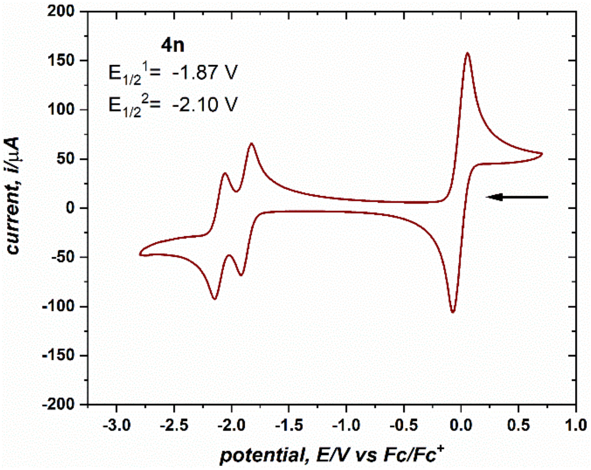
4n
$E_{1/2}^1$= -1.87 V
$E_{1/2}^2$= -2.10 V
current, i/μA
potential, E/V vs Fc/Fc+

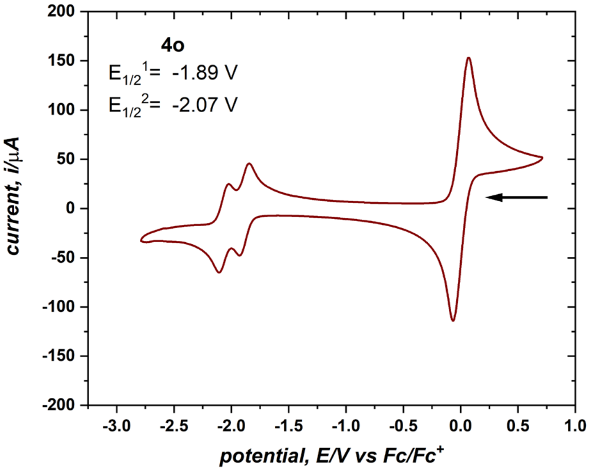
4o
$E_{1/2}^1$= -1.89 V
$E_{1/2}^2$= -2.07 V
current, i/μA
potential, E/V vs Fc/Fc+

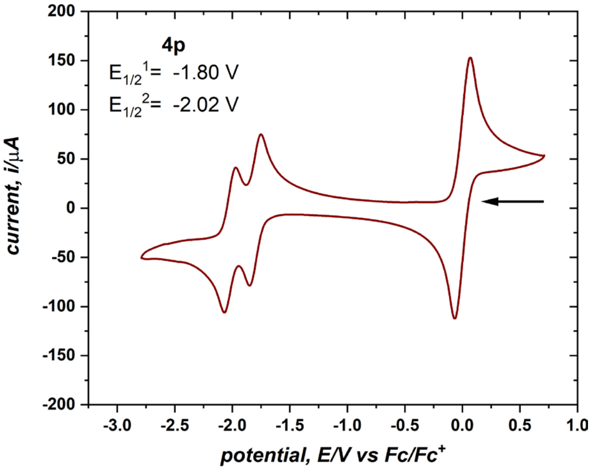
4p
$E_{1/2}^1$= -1.80 V
$E_{1/2}^2$= -2.02 V
current, i/μA
potential, E/V vs Fc/Fc+

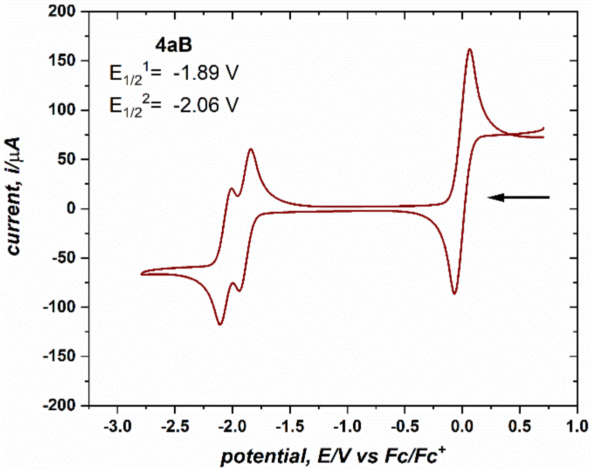
4aB
$E_{1/2}^1$= -1.89 V
$E_{1/2}^2$= -2.06 V
current, i/μA
potential, E/V vs Fc/Fc+

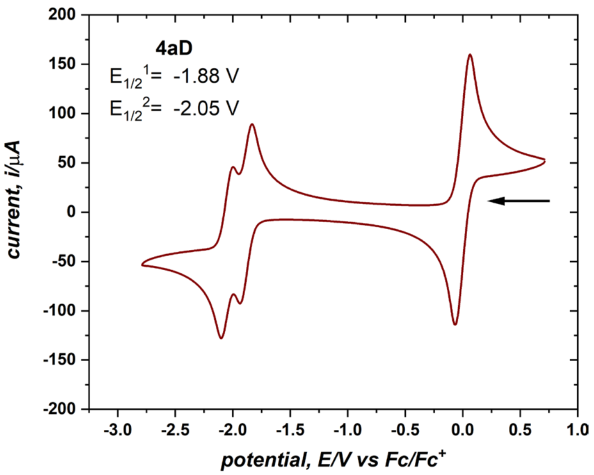
4aD
$E_{1/2}^{1}$= -1.88 V
$E_{1/2}^{2}$= -2.05 V
current, i/μA
potential, E/V vs Fc/Fc+

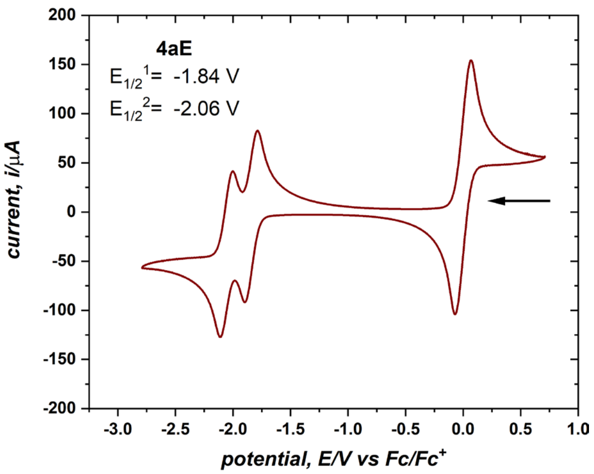
4aE
$E_{1/2}^{1}$= -1.84 V
$E_{1/2}^{2}$= -2.06 V
current, i/μA
potential, E/V vs Fc/Fc+

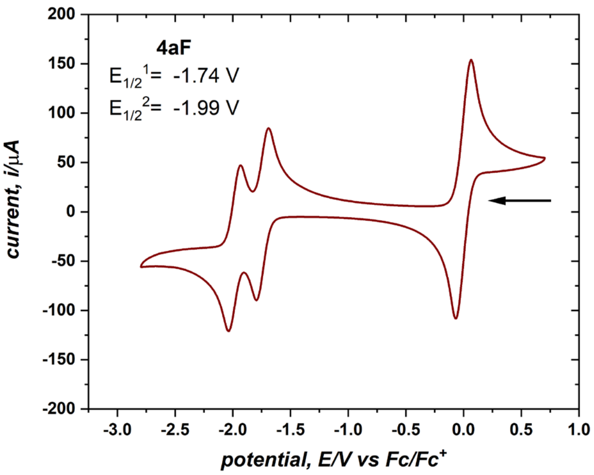
4aF
$E_{1/2}^{1}$= -1.74 V
$E_{1/2}^{2}$= -1.99 V
current, i/μA
potential, E/V vs Fc/Fc+

Scan Rate Dependence:

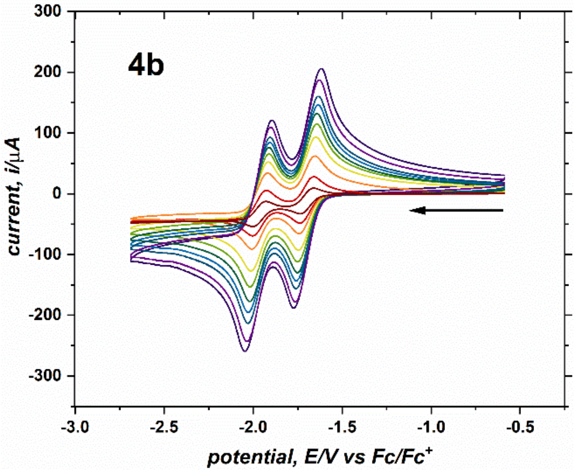

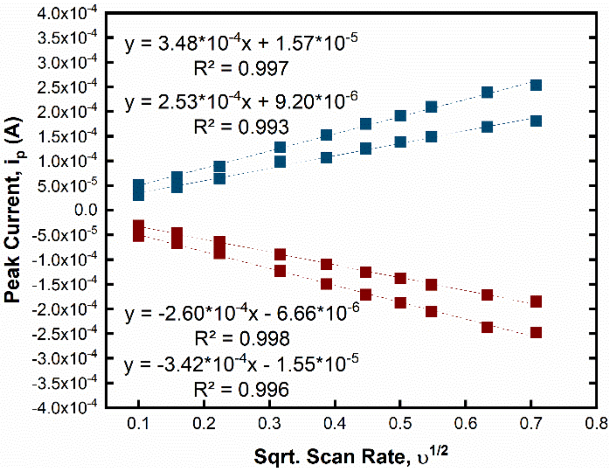

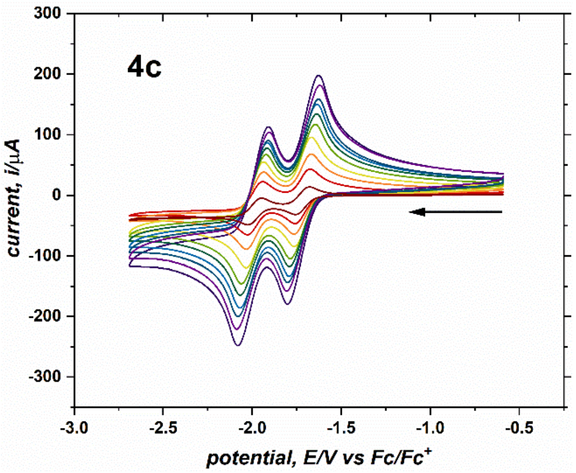

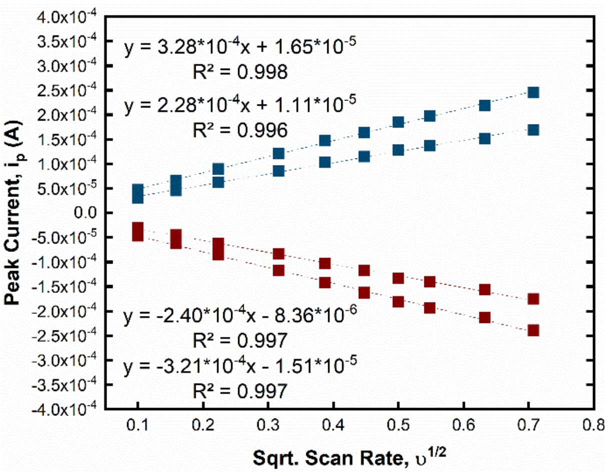

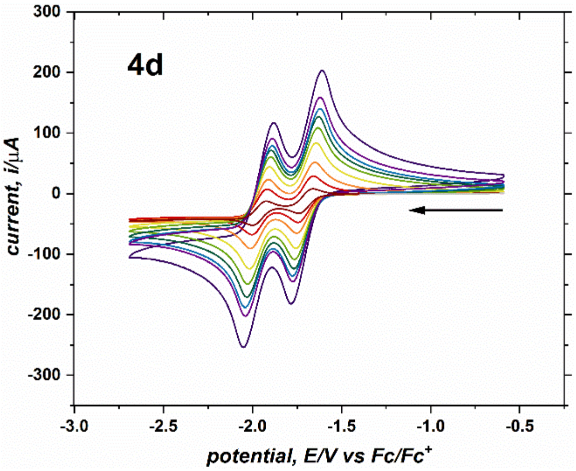

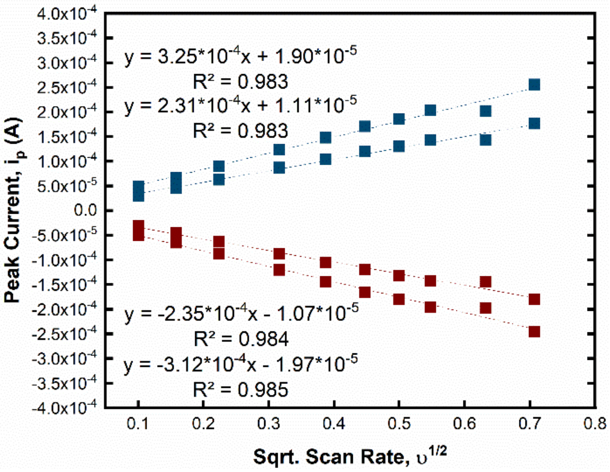

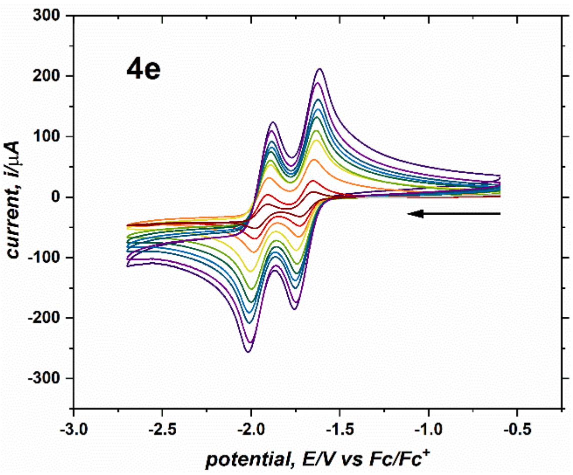

4e
current, i/μA
potential, E/V vs Fc/Fc+

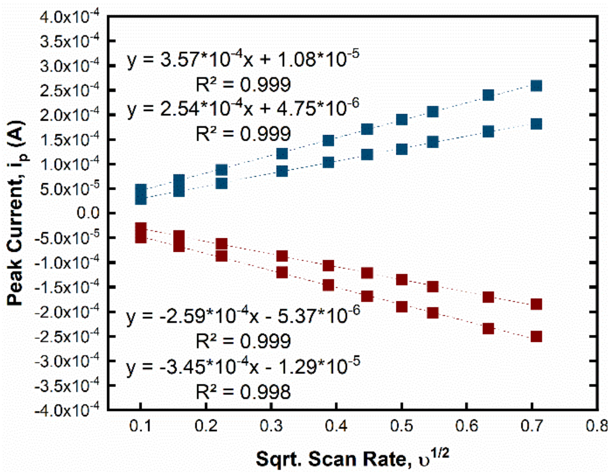

y = 3.57*10^-4x + 1.08*10^-5
R² = 0.999
y = 2.54*10^-4x + 4.75*10^-6
R² = 0.999
y = -2.59*10^-4x - 5.37*10^-6
R² = 0.999
y = -3.45*10^-4x - 1.29*10^-5
R² = 0.998
Peak Current, i_p (A)
Sqrt. Scan Rate, υ^1/2

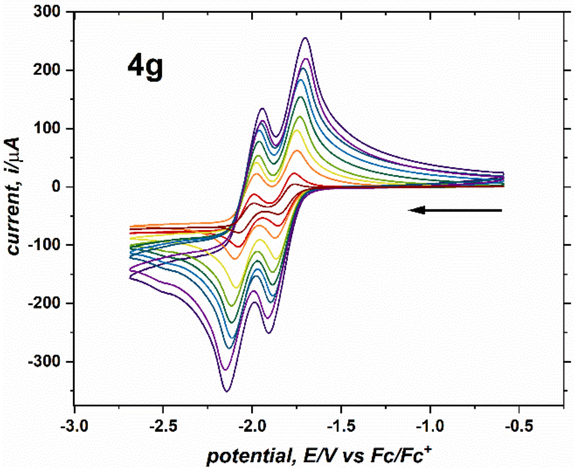

4g
current, i/μA
potential, E/V vs Fc/Fc+

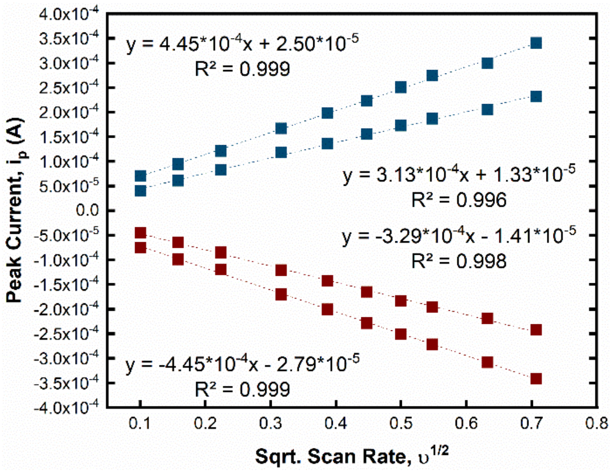

y = 4.45*10^-4x + 2.50*10^-5
R² = 0.999
y = 3.13*10^-4x + 1.33*10^-5
R² = 0.996
y = -3.29*10^-4x - 1.41*10^-5
R² = 0.998
y = -4.45*10^-4x - 2.79*10^-5
R² = 0.999
Peak Current, i_p (A)
Sqrt. Scan Rate, υ^1/2

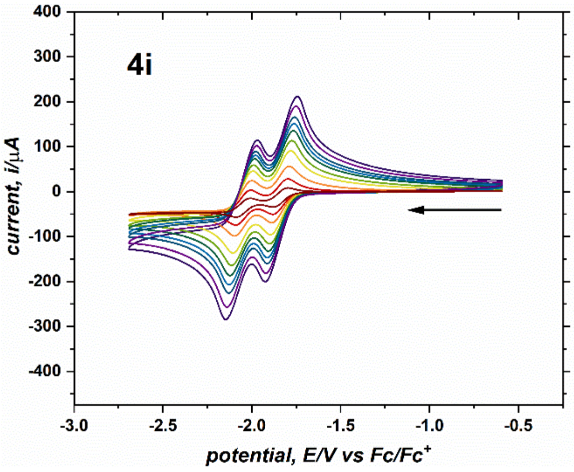

4i
current, i/μA
potential, E/V vs Fc/Fc+

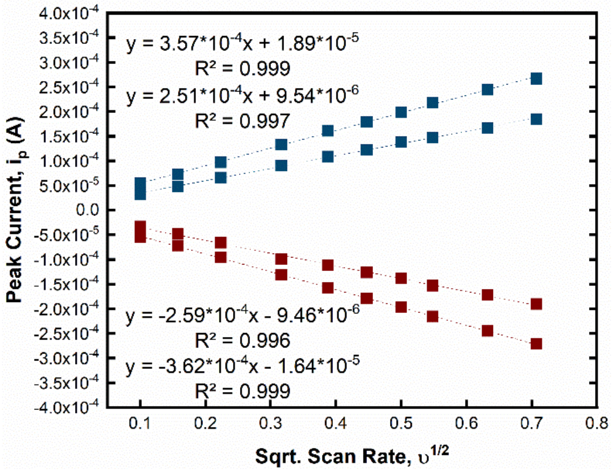

y = 3.57*10^-4x + 1.89*10^-5
R² = 0.999
y = 2.51*10^-4x + 9.54*10^-6
R² = 0.997
y = -2.59*10^-4x - 9.46*10^-6
R² = 0.996
y = -3.62*10^-4x - 1.64*10^-5
R² = 0.999
Peak Current, i_p (A)
Sqrt. Scan Rate, υ^1/2

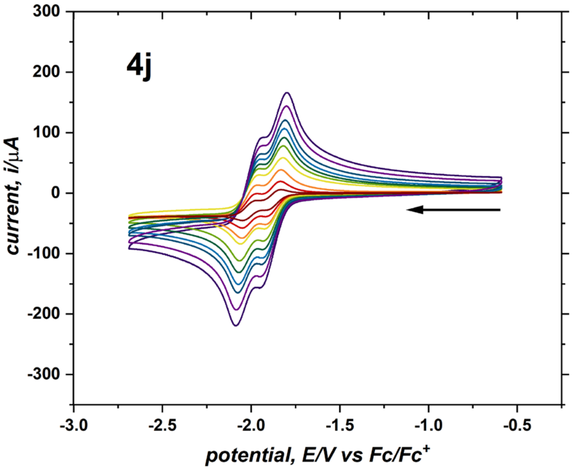
4j
current, i/μA
potential, E/V vs Fc/Fc+
300
200
100
0
-100
-200
-300
-3.0
-2.5
-2.0
-1.5
-1.0
-0.5

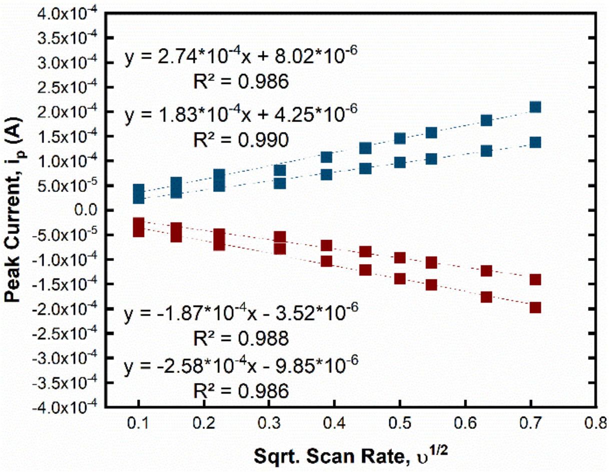
y = 2.74*10-4x + 8.02*10-6
R² = 0.986
y = 1.83*10-4x + 4.25*10-6
R² = 0.990
y = -1.87*10-4x - 3.52*10-6
R² = 0.988
y = -2.58*10-4x - 9.85*10-6
R² = 0.986
Peak Current, ip (A)
Sqrt. Scan Rate, υ1/2

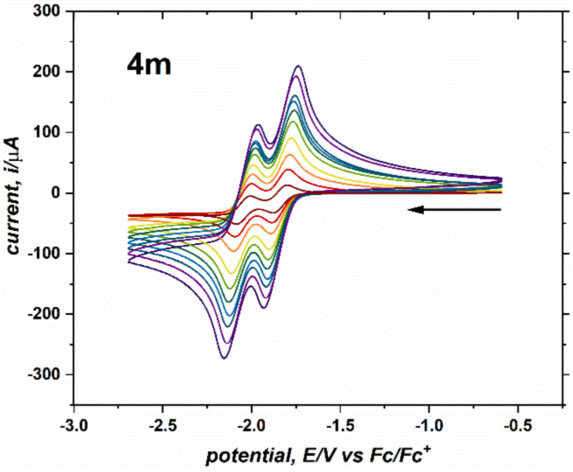
4m
current, i/μA
potential, E/V vs Fc/Fc+

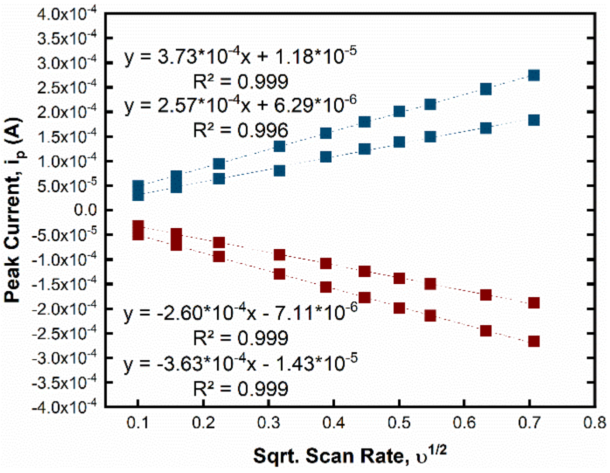
y = 3.73*10-4x + 1.18*10-5
R² = 0.999
y = 2.57*10-4x + 6.29*10-6
R² = 0.996
y = -2.60*10-4x - 7.11*10-6
R² = 0.999
y = -3.63*10-4x - 1.43*10-5
R² = 0.999
Peak Current, ip (A)
Sqrt. Scan Rate, υ1/2

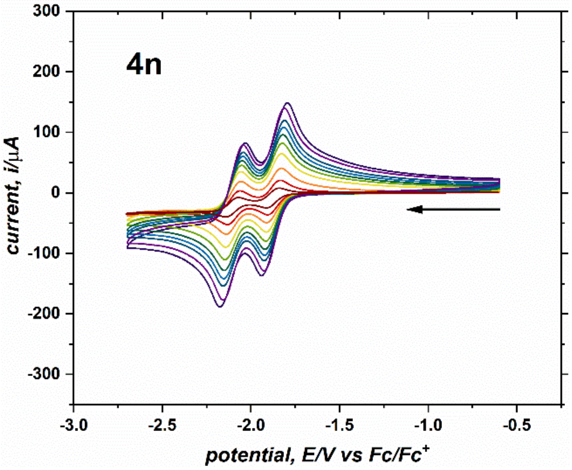
4n
current, i/μA
potential, E/V vs Fc/Fc+

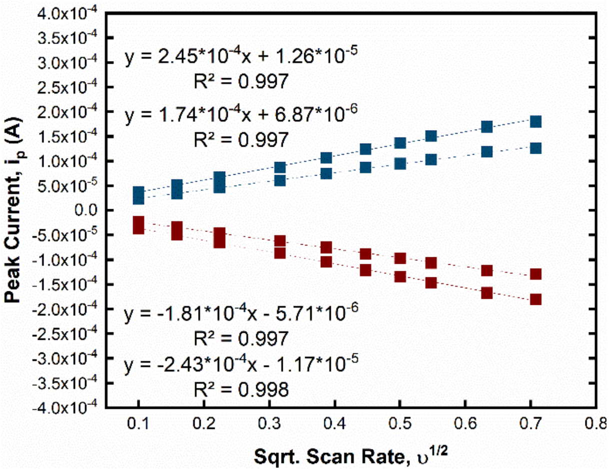
y = 2.45*10-4x + 1.26*10-5
R² = 0.997
y = 1.74*10-4x + 6.87*10-6
R² = 0.997
y = -1.81*10-4x - 5.71*10-6
R² = 0.997
y = -2.43*10-4x - 1.17*10-5
R² = 0.998
Peak Current, ip (A)
Sqrt. Scan Rate, υ1/2

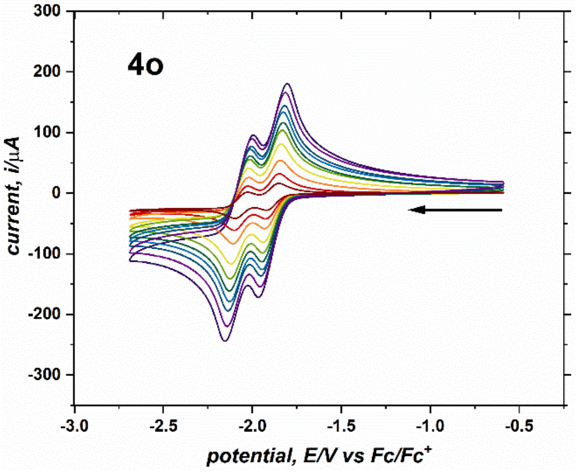
4o
current, i/μA
potential, E/V vs Fc/Fc+
300
200
100
0
-100
-200
-300
-3.0
-2.5
-2.0
-1.5
-1.0
-0.5

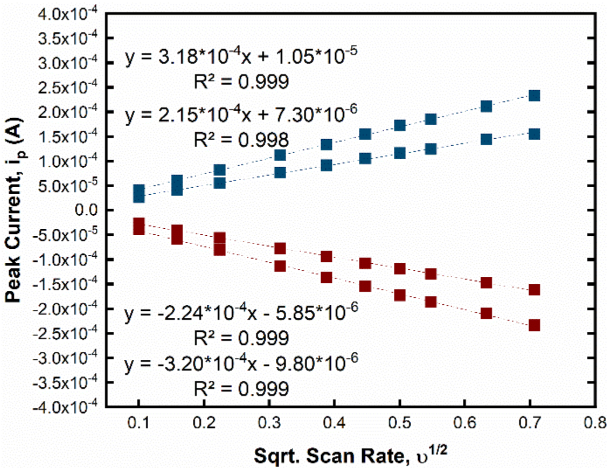
y = 3.18*10-4x + 1.05*10-5
R² = 0.999
y = 2.15*10-4x + 7.30*10-6
R² = 0.998
y = -2.24*10-4x - 5.85*10-6
R² = 0.999
y = -3.20*10-4x - 9.80*10-6
R² = 0.999
Peak Current, ip (A)
Sqrt. Scan Rate, υ1/2

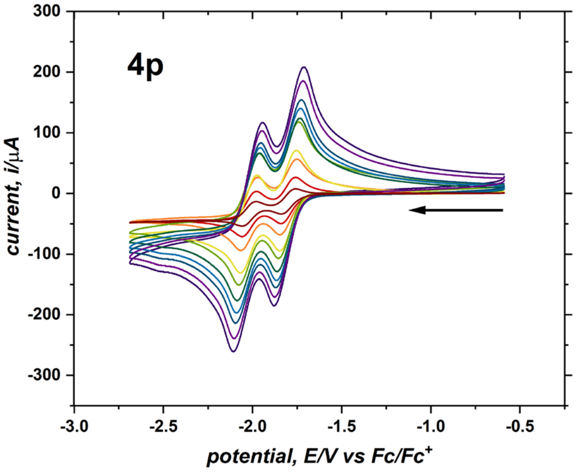
4p
current, i/μA
potential, E/V vs Fc/Fc+
300
200
100
0
-100
-200
-300
-3.0
-2.5
-2.0
-1.5
-1.0
-0.5

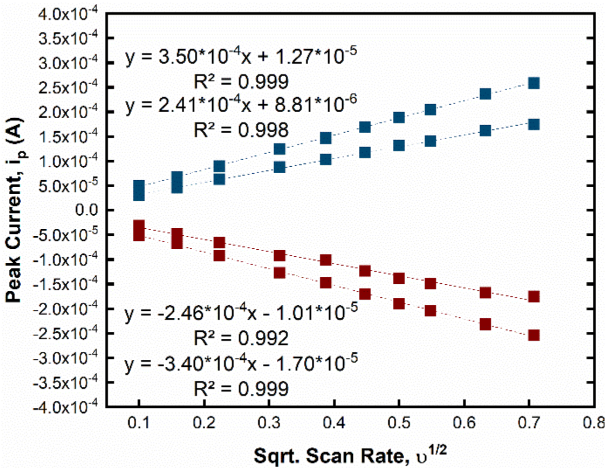
y = 3.50*10-4x + 1.27*10-5
R² = 0.999
y = 2.41*10-4x + 8.81*10-6
R² = 0.998
y = -2.46*10-4x - 1.01*10-5
R² = 0.992
y = -3.40*10-4x - 1.70*10-5
R² = 0.999
Peak Current, ip (A)
Sqrt. Scan Rate, υ1/2

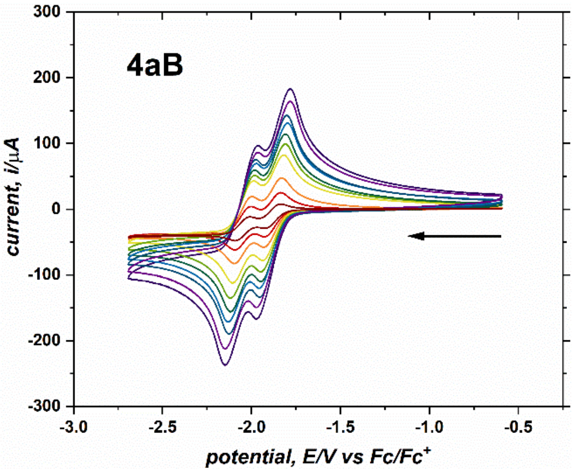
4aB
current, i/μA
potential, E/V vs Fc/Fc+
300
200
100
0
-100
-200
-300
-3.0
-2.5
-2.0
-1.5
-1.0
-0.5

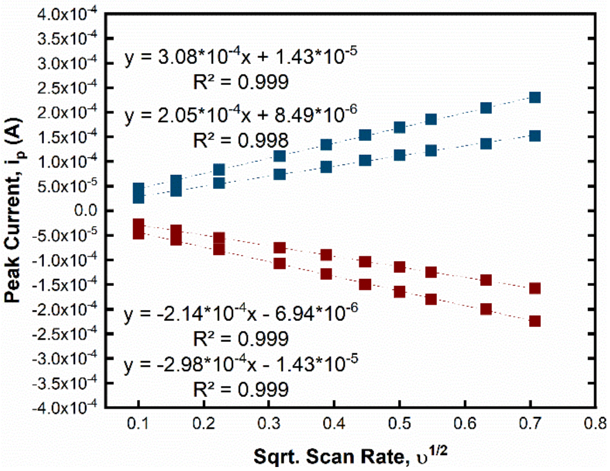
y = 3.08*10-4x + 1.43*10-5
R² = 0.999
y = 2.05*10-4x + 8.49*10-6
R² = 0.998
y = -2.14*10-4x - 6.94*10-6
R² = 0.999
y = -2.98*10-4x - 1.43*10-5
R² = 0.999
Peak Current, ip (A)
Sqrt. Scan Rate, υ1/2

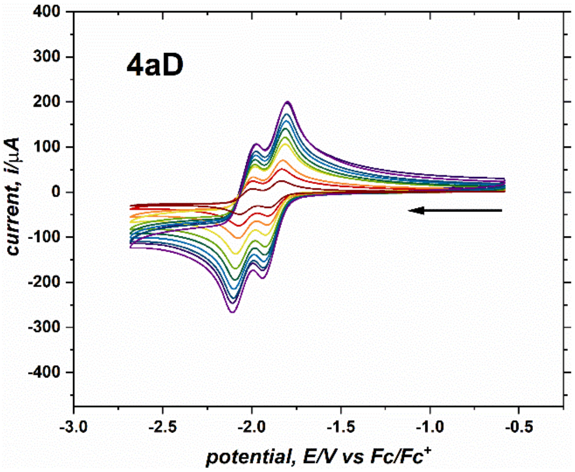
4aD
current, i/μA
potential, E/V vs Fc/Fc+

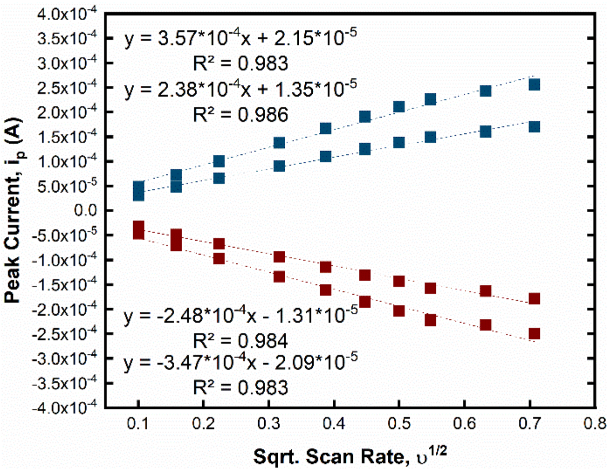
y = 3.57*10-4x + 2.15*10-5
R² = 0.983
y = 2.38*10-4x + 1.35*10-5
R² = 0.986
y = -2.48*10-4x - 1.31*10-5
R² = 0.984
y = -3.47*10-4x - 2.09*10-5
R² = 0.983
Peak Current, ip (A)
Sqrt. Scan Rate, υ1/2

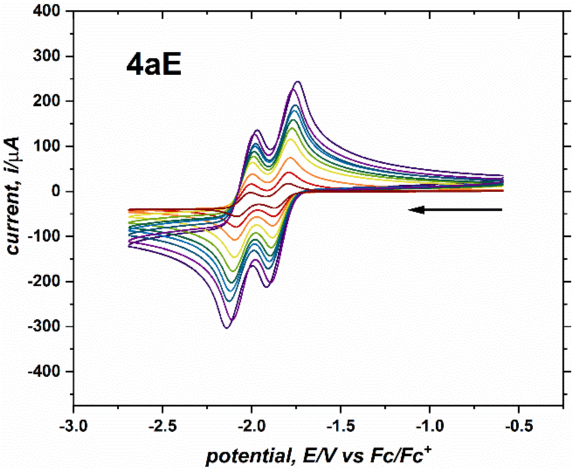
4aE
current, i/μA
potential, E/V vs Fc/Fc+

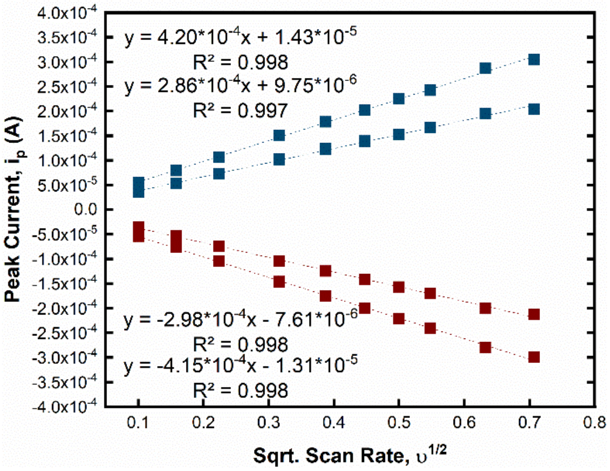
y = 4.20*10-4x + 1.43*10-5
R² = 0.998
y = 2.86*10-4x + 9.75*10-6
R² = 0.997
y = -2.98*10-4x - 7.61*10-6
R² = 0.998
y = -4.15*10-4x - 1.31*10-5
R² = 0.998
Peak Current, ip (A)
Sqrt. Scan Rate, υ1/2

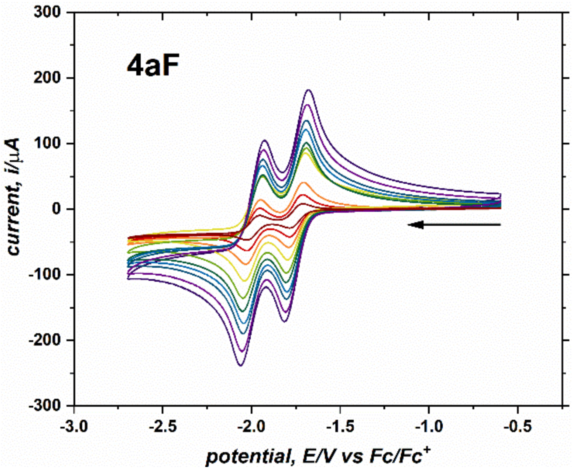
4aF
current, i/μA
potential, E/V vs Fc/Fc+

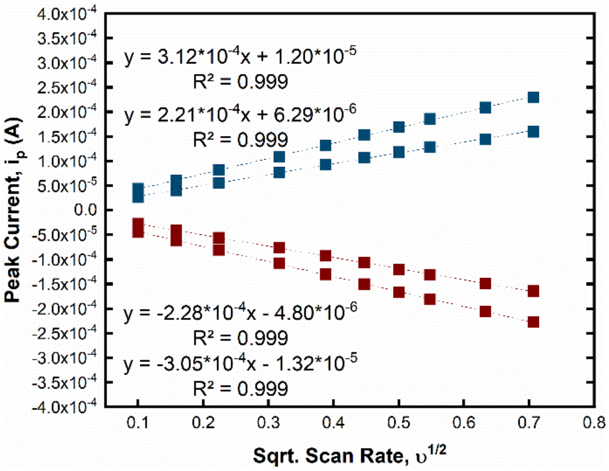
y = 3.12*10-4x + 1.20*10-5
R² = 0.999
y = 2.21*10-4x + 6.29*10-6
R² = 0.999
y = -2.28*10-4x - 4.80*10-6
R² = 0.999
y = -3.05*10-4x - 1.32*10-5
R² = 0.999
Peak Current, ip (A)
Sqrt. Scan Rate, υ1/2

H-Cell Cycling Procedure:

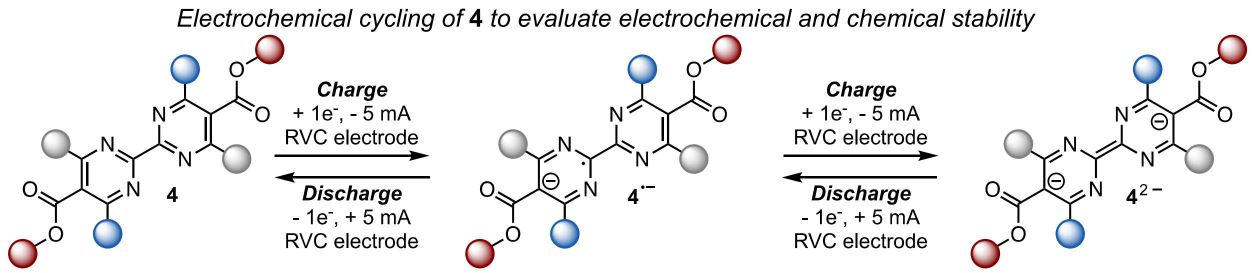

Figure S1: H-cell cycling of 2,2'-bipyrimidines

Charge/discharge cycling measurements were carried out via cyclic step chronopotentiometry in a N_2-filled glovebox with a Pine WaveNow potentiostat/galvanostat in a custom glass H-cell with an ultra-fine fritted glass separator (P5, Adams and Chittenden). The working and counter electrodes were reticulated vitreous carbon (RVC, 100 ppi, ~ 8.5 cm^2 surface area, ERG Aerospace) attached to nickel wire. A Ag/$AgNO_3$ (10 mM with respect to Ag/$AgNO_3$ in 0.10 M $TBAPF_6$ in acetonitrile) electrode from BASi® was used as the reference electrode. The working side of the H-cell was loaded with active anolyte and 5 mL of supporting electrolyte (0.5 M $TBAPF_6$ in acetonitrile) while the counter side consisted of 5 mL of the supporting electrolyte solution. A pressure equalizing bridge was utilized to minimize crossover between the sides of the H-cell. While cycling at a constant current of ± 5 mA, both sides of the H-cell were stirred continuously at 940 rpm to ensure consistent and effective mass transport to the electrodes. After the first cycle, the counter side solution and electrode were replaced. Voltage cutoffs of –2.3 V and –0.9 V versus the Ag/$AgNO_3$ reference were used as the potential cutoffs for the charging and discharging processes, respectively. Figure S2 shows three separate trials for a 1st-generation anolyte and averaged metrics for the three trials; importantly, the rates of fade are nearly identical in all three cases, highlighting the reproducibility of this assay.

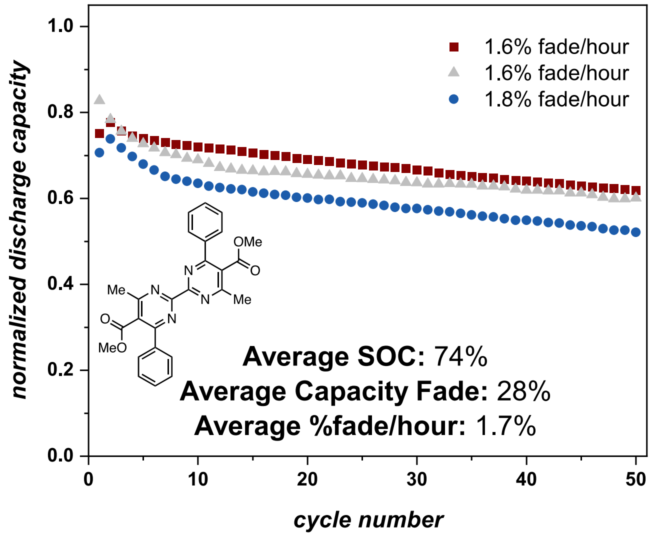

Figure S2. Reproducibility of H-Cell cycling assay.

H-Cell Cycling Data:

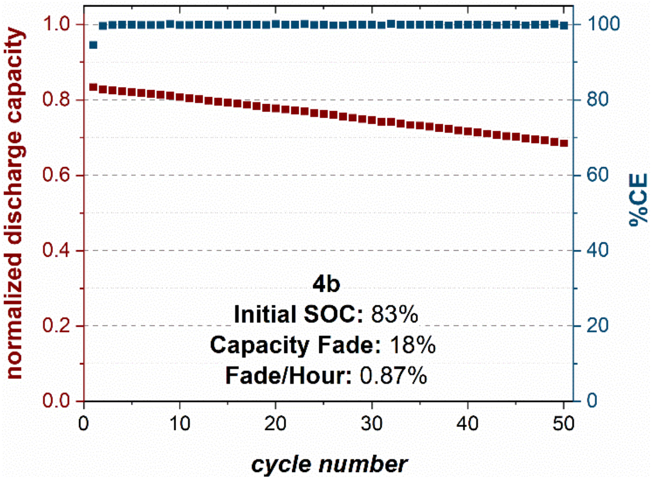

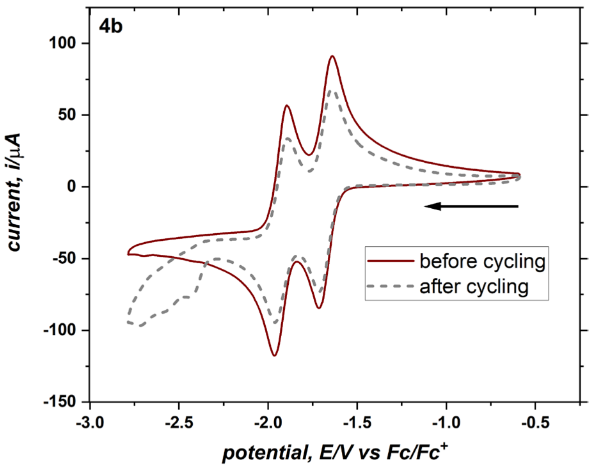

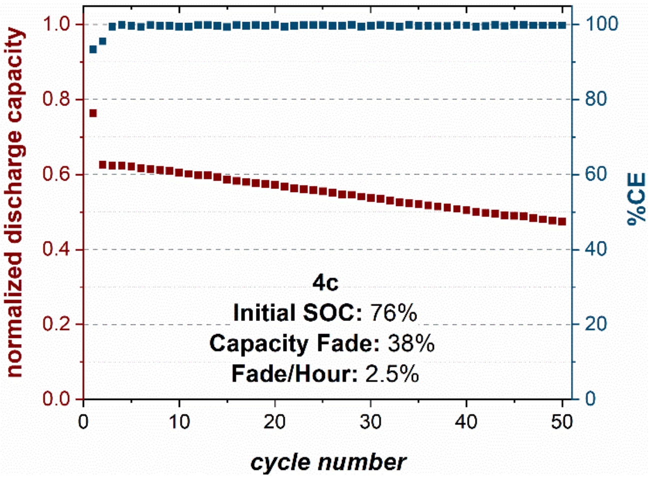

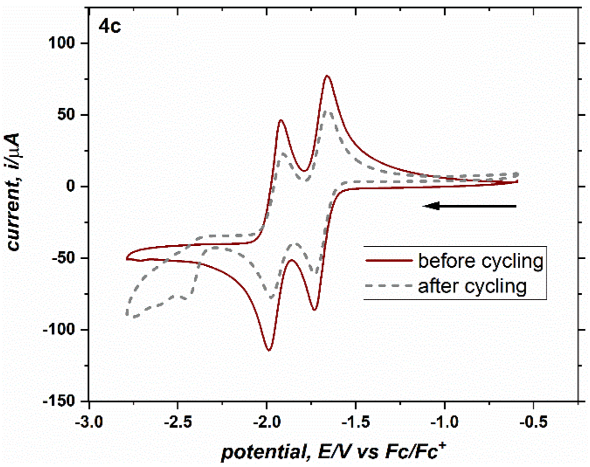

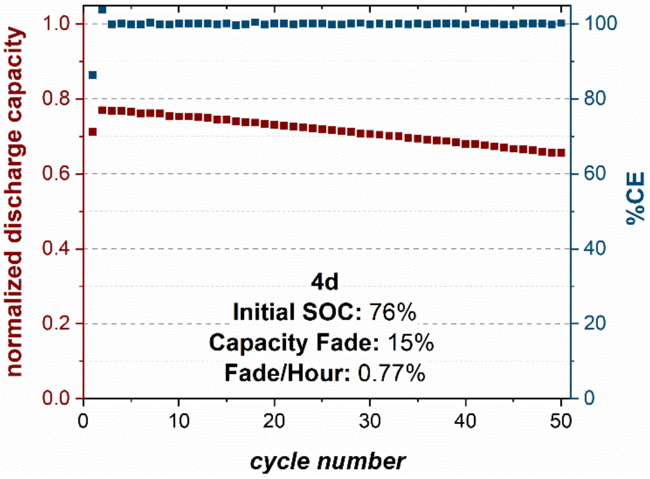

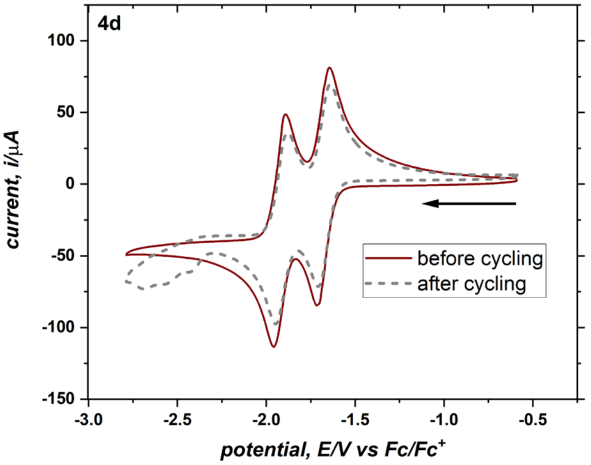

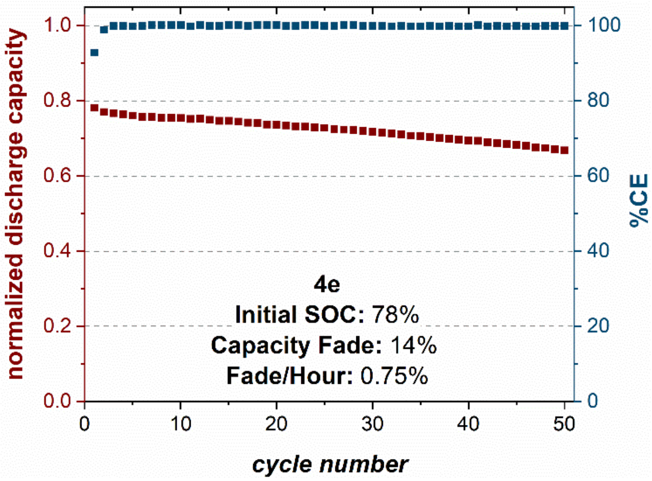
normalized discharge capacity
%CE
cycle number
4e
Initial SOC: 78%
Capacity Fade: 14%
Fade/Hour: 0.75%

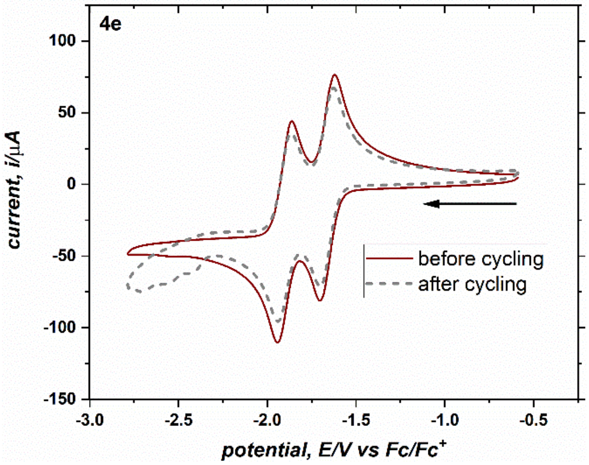
4e
current, i/μA
potential, E/V vs Fc/Fc+
before cycling
after cycling

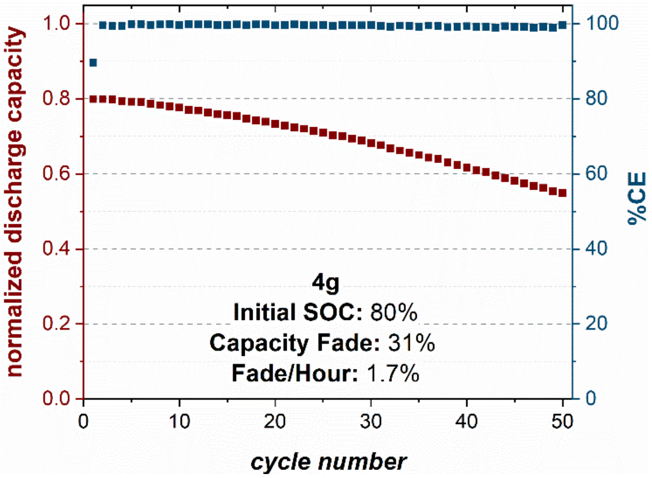
normalized discharge capacity
%CE
cycle number
4g
Initial SOC: 80%
Capacity Fade: 31%
Fade/Hour: 1.7%

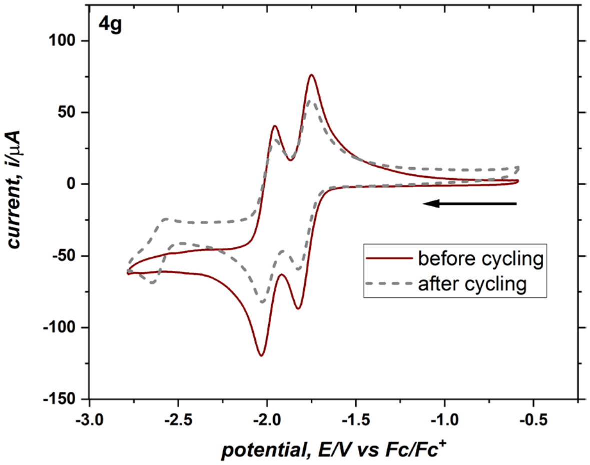
4g
current, i/μA
potential, E/V vs Fc/Fc+
before cycling
after cycling

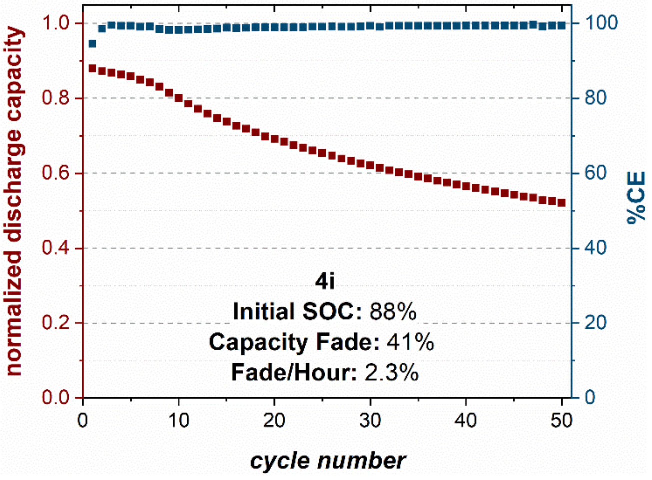
normalized discharge capacity
%CE
cycle number
4i
Initial SOC: 88%
Capacity Fade: 41%
Fade/Hour: 2.3%

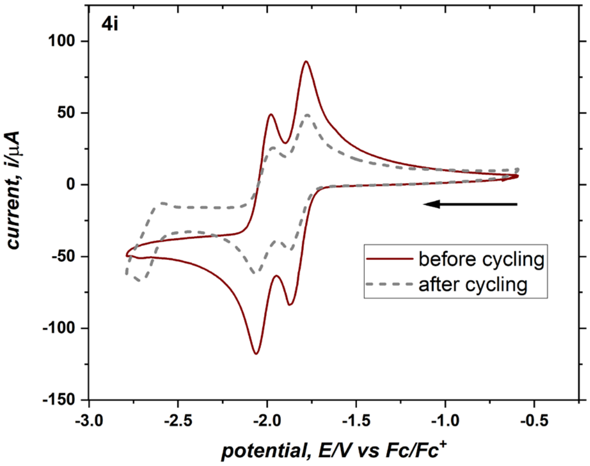
4i
current, i/μA
potential, E/V vs Fc/Fc+
before cycling
after cycling

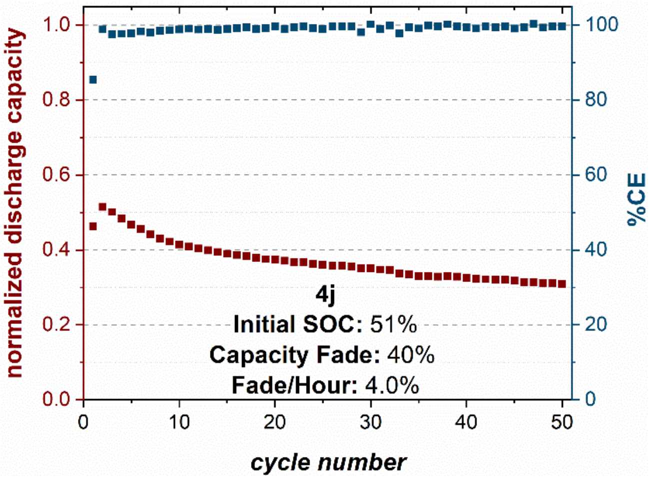

4j
Initial SOC: 51%
Capacity Fade: 40%
Fade/Hour: 4.0%
normalized discharge capacity
%CE
cycle number

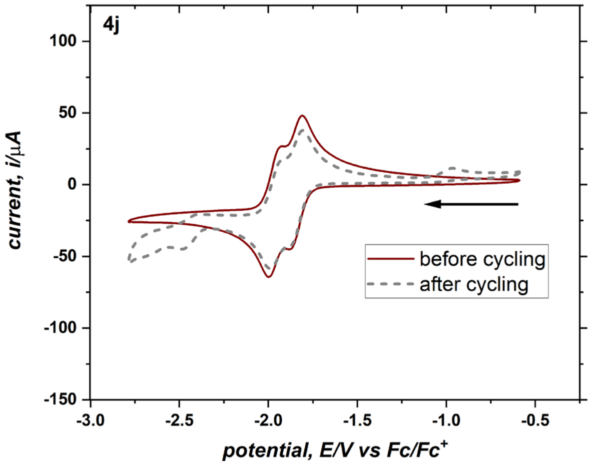

4j
before cycling
after cycling
current, i/μA
potential, E/V vs Fc/Fc+

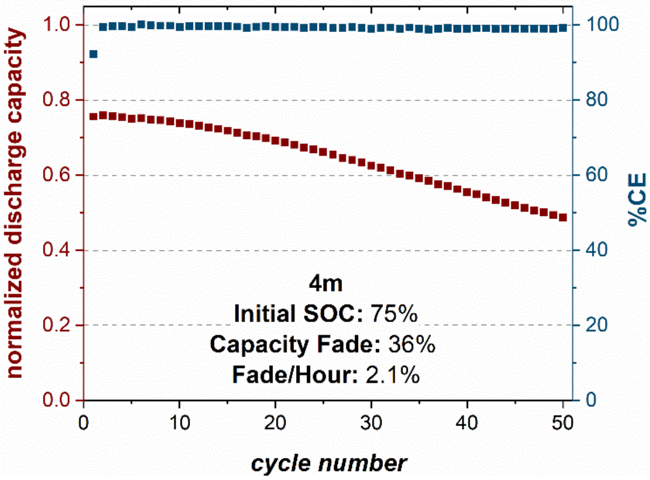

4m
Initial SOC: 75%
Capacity Fade: 36%
Fade/Hour: 2.1%
normalized discharge capacity
%CE
cycle number

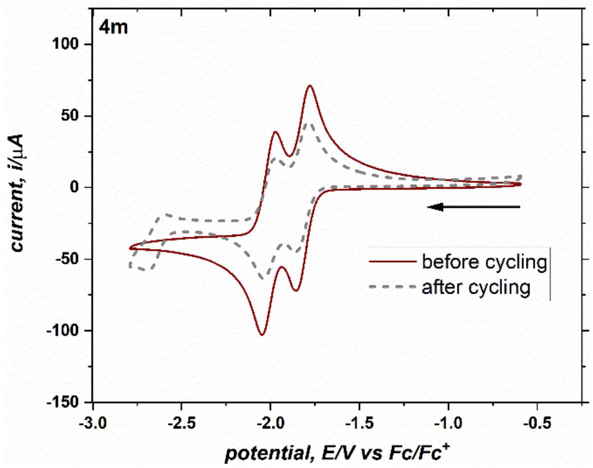

4m
before cycling
after cycling
current, i/μA
potential, E/V vs Fc/Fc+

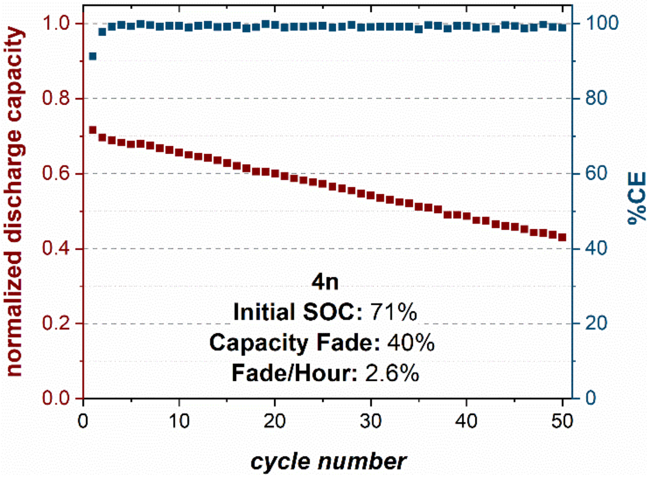

4n
Initial SOC: 71%
Capacity Fade: 40%
Fade/Hour: 2.6%
normalized discharge capacity
%CE
cycle number

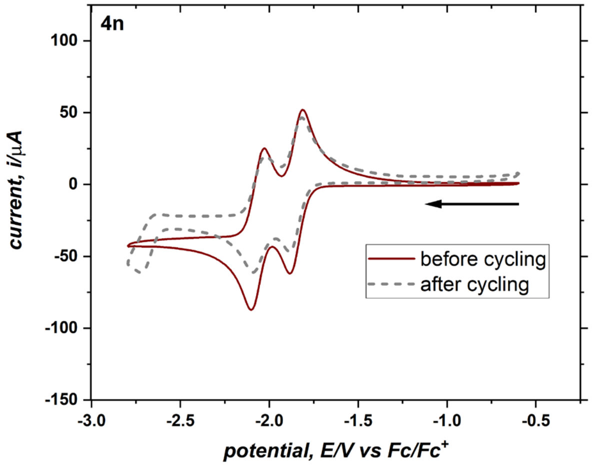

4n
before cycling
after cycling
current, i/μA
potential, E/V vs Fc/Fc+

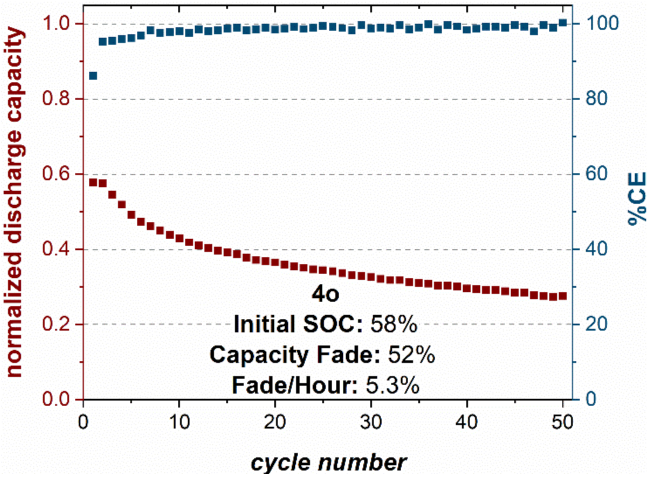
normalized discharge capacity
%CE
cycle number
4o
Initial SOC: 58%
Capacity Fade: 52%
Fade/Hour: 5.3%

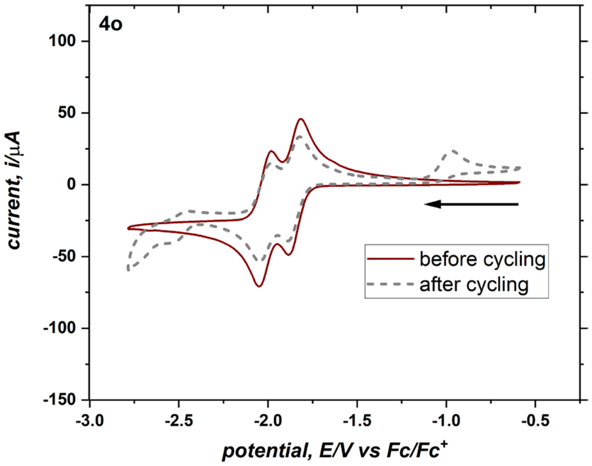
4o
current, i/μA
potential, E/V vs Fc/Fc^+
before cycling
after cycling

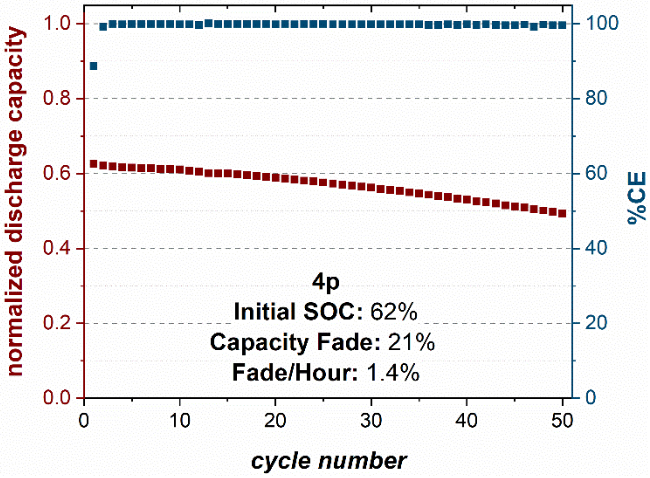
normalized discharge capacity
%CE
cycle number
4p
Initial SOC: 62%
Capacity Fade: 21%
Fade/Hour: 1.4%

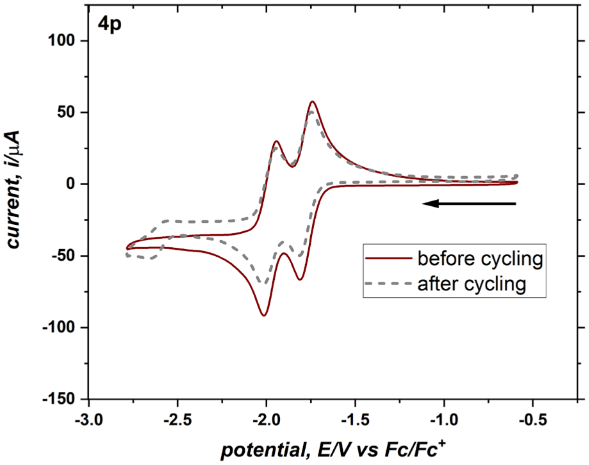
4p
current, i/μA
potential, E/V vs Fc/Fc^+
before cycling
after cycling

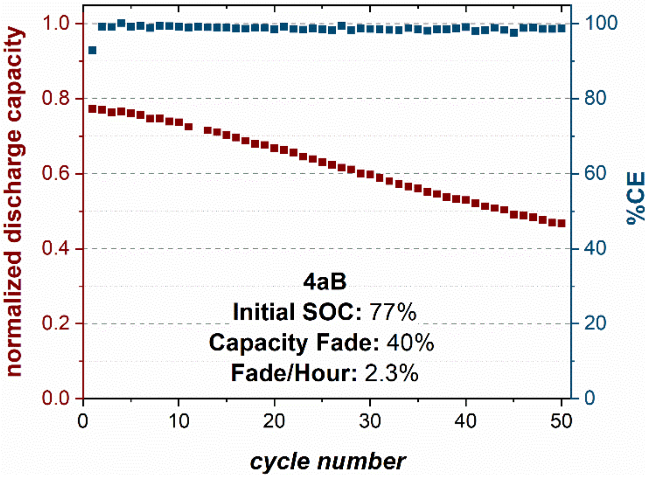
normalized discharge capacity
%CE
cycle number
4aB
Initial SOC: 77%
Capacity Fade: 40%
Fade/Hour: 2.3%

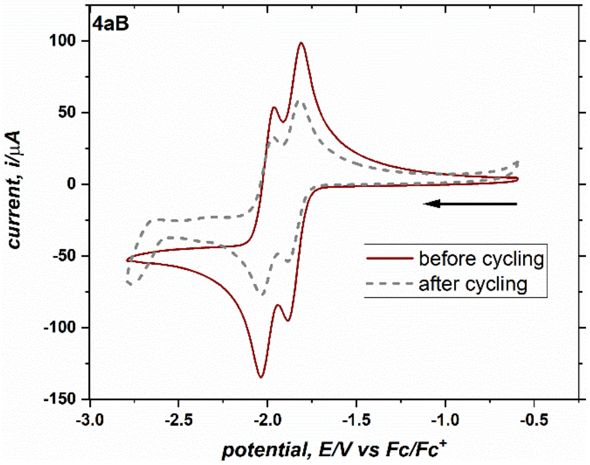
4aB
current, i/μA
potential, E/V vs Fc/Fc^+
before cycling
after cycling

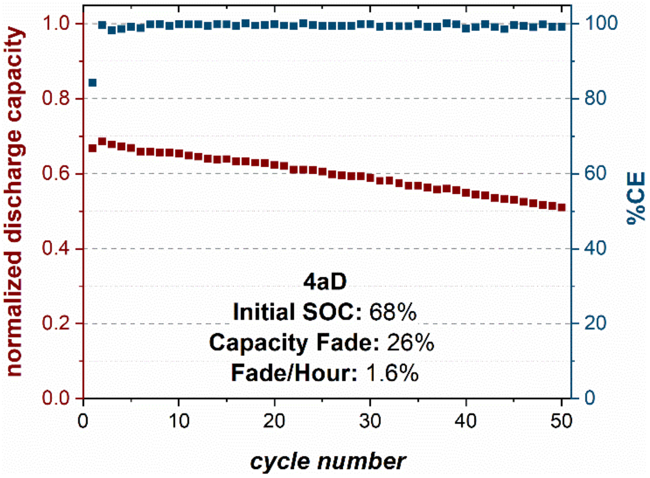
4aD
Initial SOC: 68%
Capacity Fade: 26%
Fade/Hour: 1.6%
normalized discharge capacity
%CE
cycle number

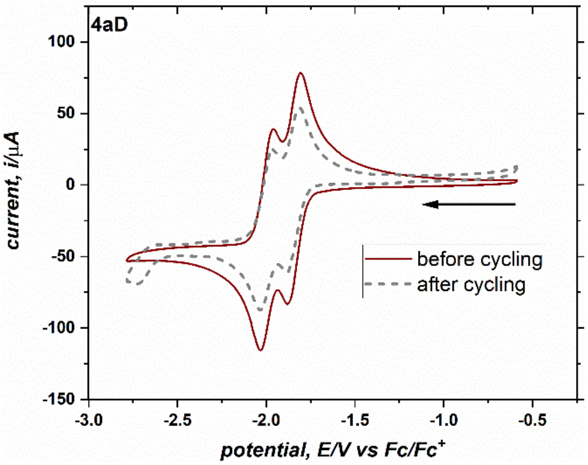
4aD
before cycling
after cycling
current, i/μA
potential, E/V vs Fc/Fc+

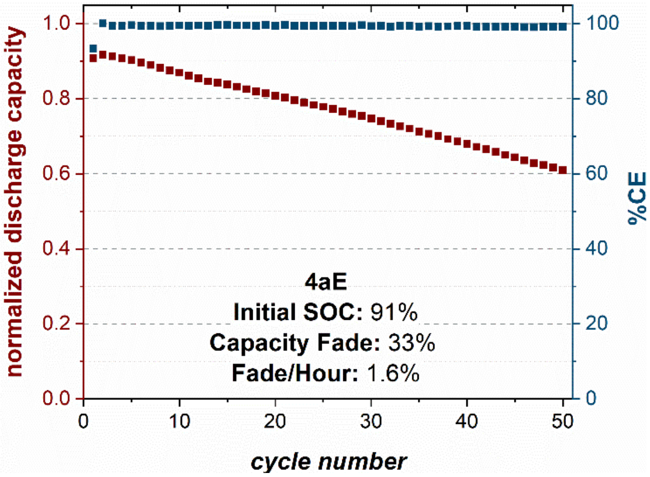
4aE
Initial SOC: 91%
Capacity Fade: 33%
Fade/Hour: 1.6%
normalized discharge capacity
%CE
cycle number

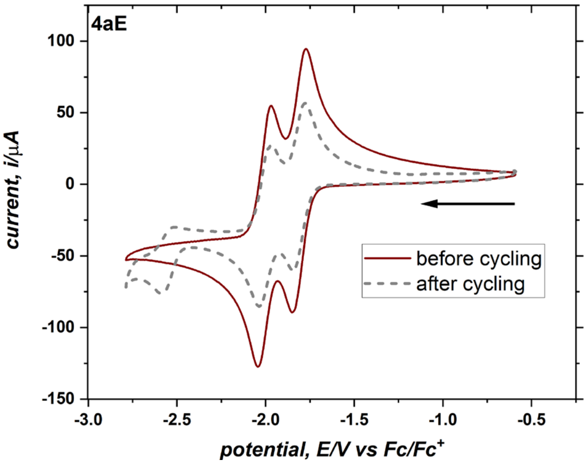
4aE
before cycling
after cycling
current, i/μA
potential, E/V vs Fc/Fc+

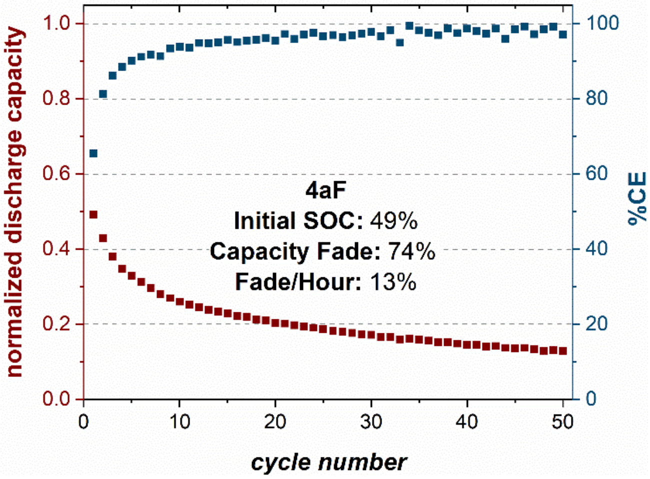
4aF
Initial SOC: 49%
Capacity Fade: 74%
Fade/Hour: 13%
normalized discharge capacity
%CE
cycle number

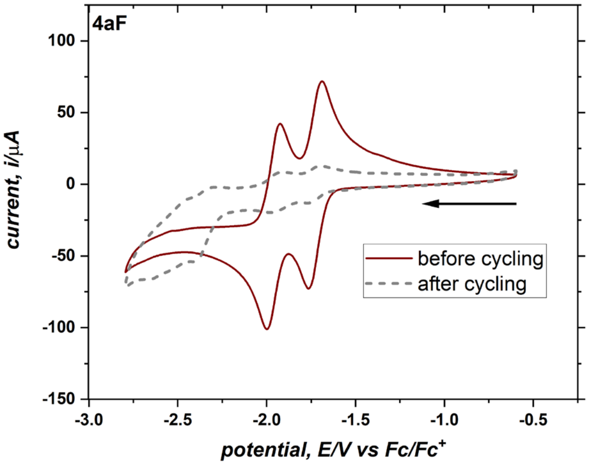
4aF
before cycling
after cycling
current, i/μA
potential, E/V vs Fc/Fc+

Battery Cycling:

General: All compatibility testing and battery cycling experiments were conducted in a nitrogen-filled glovebox with a Biologic VSP multichannel potentiostat/galvanostat. 99.9 % extra dry acetonitrile was obtained from Acros Organics and was dried over activated 3 Å molecular sieves for 24 hours prior to use. Tetra-*n*-butylammonium hexafluorophosphate (electrochemical grade) was obtained from Sigma-Aldrich and dried under vacuum at 50 °C for 24 hours prior to use. Potassium hexafluorophosphate (99.8 %) was obtained from Sigma-Aldrich and was recrystallized from an aqueous KOH solution and dried under vacuum at 50 °C for 48 hours prior to use. **CP** and **4-DMPP** were synthesized according to previous reports.[6b,c]

Compatibility Studies via Cyclic Voltammetry

A glassy carbon electrode (0.07 cm^2, BASi) was used as the working electrode, and a coiled platinum wire was used as the counter electrode. A Ag/Ag^+ quasi-reference electrode (BASi) containing 0.01 M $AgBF_4$ (Sigma Aldrich) in 0.5 M KPF_6/MeCN was used. All potentials are referenced to Ag/Ag^+. The CVs were performed with a scan rate of 100 mV/s, using 2 mL of a mixed 1 mM solution of **4aD** with either **CP** or **4-DMPP** in 0.5 M KPF_6 in MeCN as supporting electrolyte.

Compatibility Studies via Galvanostatic Cycling

The working and counter electrodes were comprised of reticulated vitreous carbons (100 ppi, ~70 cm2 surface area, Duocell). A Ag/Ag^+ quasi-reference electrode containing 0.01 M $AgBF_4$ in 0.5 M $TBAPF_6$/MeCN was used to reference the potential at the working electrode. A porous glass frit (P5, Adams and Chittenden) was used as the separator. Each side was charged with 5 mL of a mixture of 5 mM **4aD** and **4-DMPP** in 0.5 M $TBAPF_6$/MeCN. Cycling experiments were conducted at ±5 mA charging/discharging rates between 1.5 V and 0.2 V. Both chambers were stirred continuously during cycling.

Asymmetric Flow Cell Battery Cycling

Flow cell cycling was performed with a zero-gap flow cell comprised of graphite charge collecting plates containing an interdigitated flow field in combination with two layers of non-woven carbon felt electrodes (Sicracet 29AA) on each side. ePTFE gaskets were used to achieve ~20% compression of the felt. A Fumasep® FAP-375-PP anion-exchange membrane separated the two half cells, and the exposed area of the membrane in the gasket window was used as the active area (2.55 cm^2). After assembly, each side of the cell was charged with a mixture of 25 mM of either **4aD** or **4-DMPP** in 0.5 M KPF_6/MeCN. The cell was pretreated by continuously flowing the solution above at 10 mL/min for 2 h without any charging process using a peristaltic pump (Cole-Parmer) with Solveflex and PFA tubing. Electrochemical impedance spectroscopy (EIS) was conducted when the battery initially started flowing, after the 2-hour pretreatment, and after cycling. After this step, using the same flow rate, galvanostatic charge/discharge cycling was performed at a charging current of 10 mA/cm^2 and a discharging current of -15 mA/cm^2 with 4.0 V and 1.4 V voltage limits. The battery was cycled for 186 cycles.

Cyclic Voltammetry:

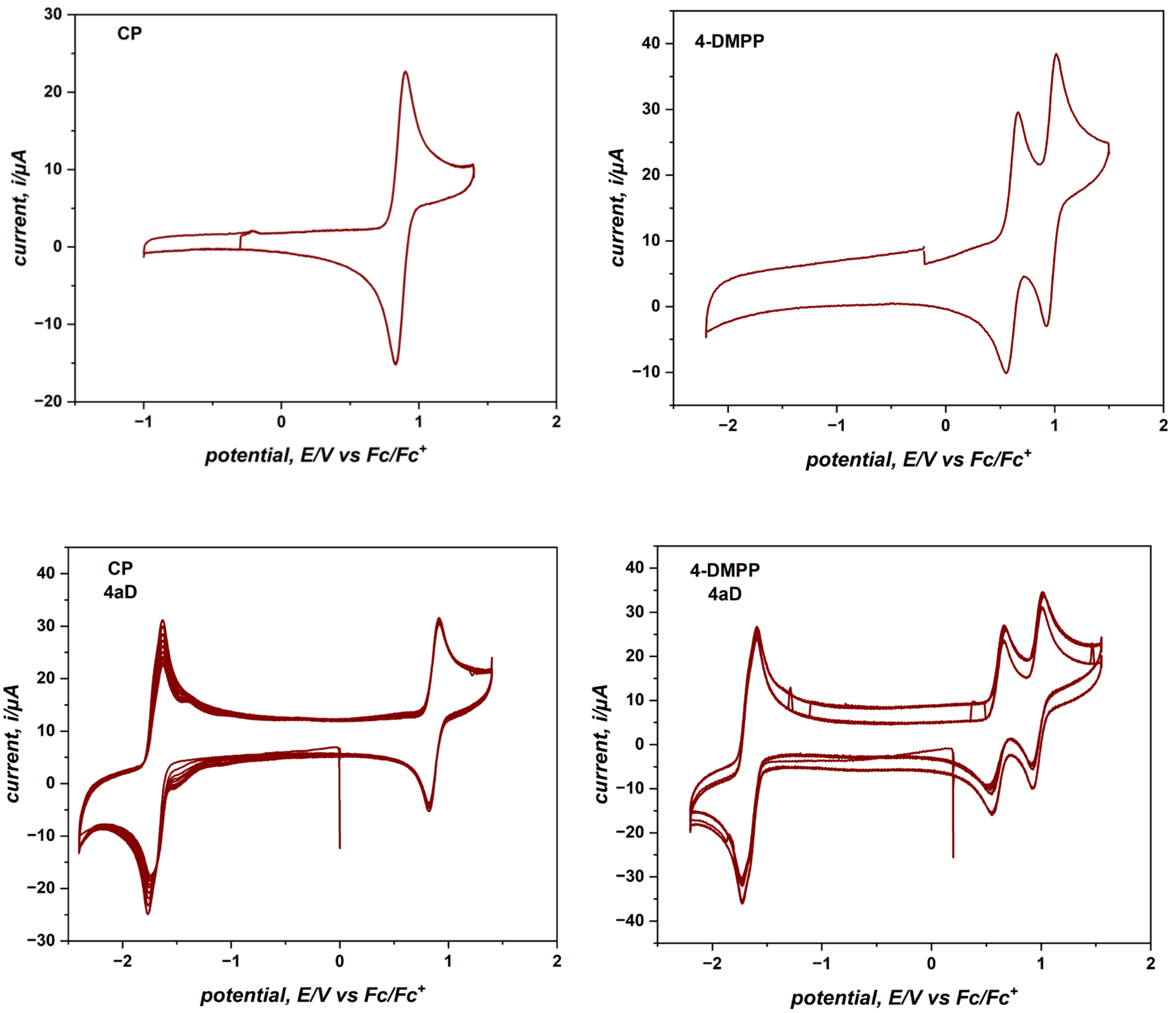

Figure S2. Cyclic voltammetry compatibility studies between **4aD** and cyclopropenium catholytes.

Mixed Oxidative Galvanostatic Cycling:

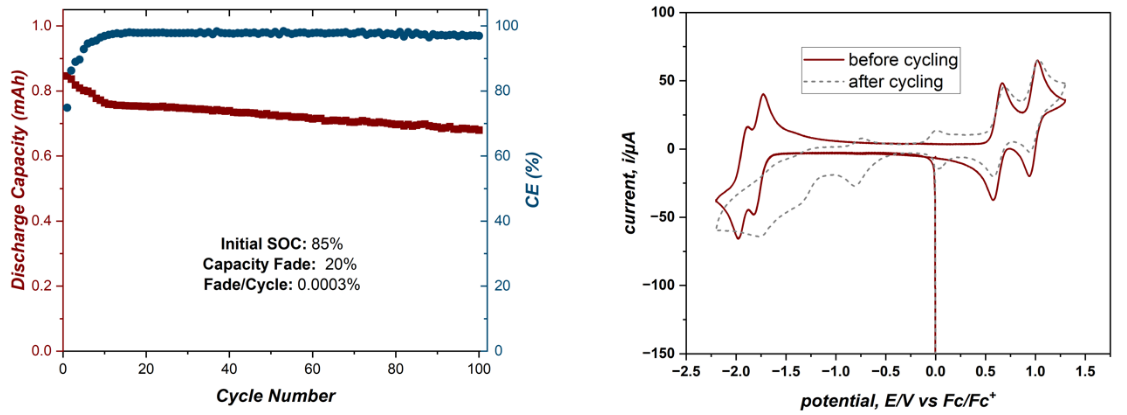

Figure S3. Cycling results from a mixed oxidative cycling between **4aD** and **4-DMPP**.

Asymmetric Flow Battery Cycling:

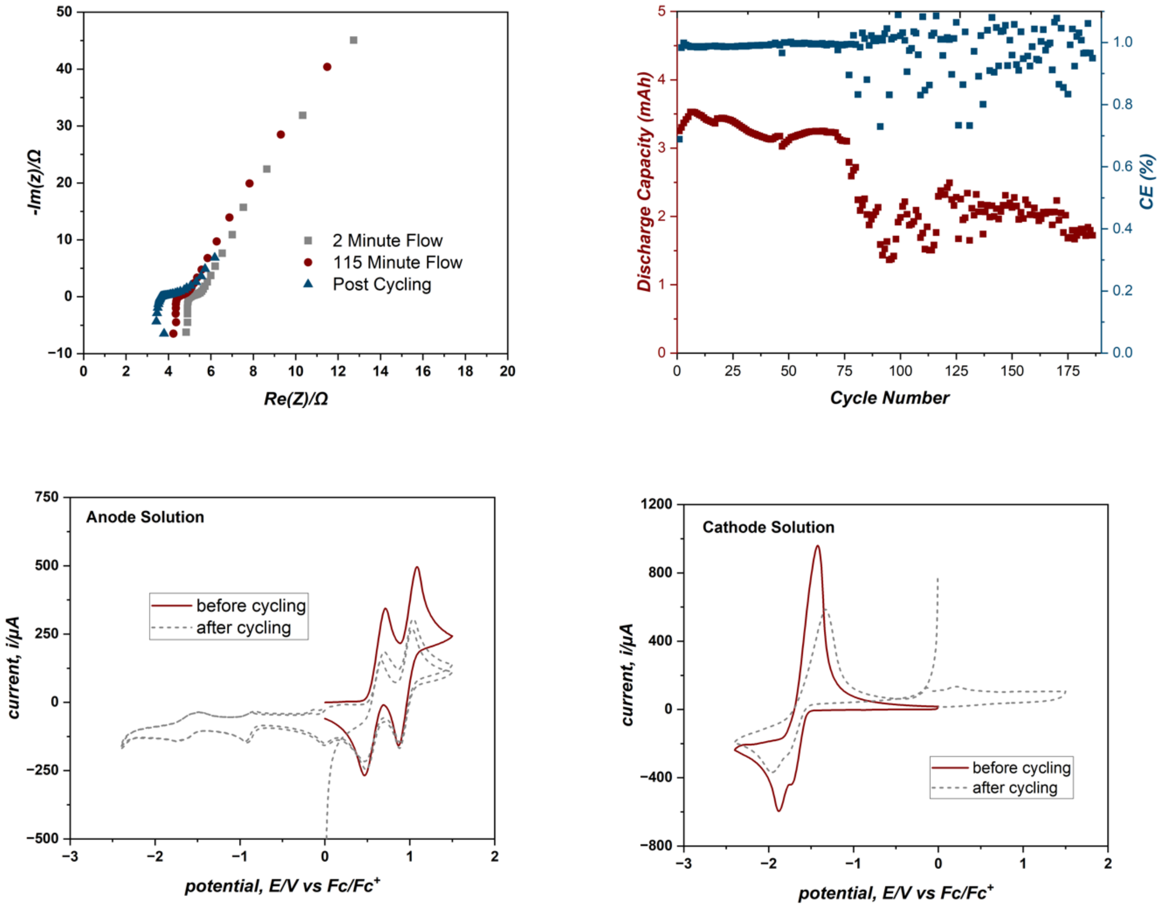

Figure S4A. Electrochemical impedance spectra recorded before and after cycling. **S4B**. Full battery cycling. **S4C**. Cyclic voltammogram of the anode solution after cycling. **S4C**. Cyclic voltammogram of the cathode solution after cycling.

IV. Solubility Measurements:

Solubility measurements were performed according to a previously published method.[6]. Concerned about the multiple charge states accessible to the bipyrimidine scaffold, studies were conducted to identify the solubility (capacity) limiting state. Shown in Figure S5, reduction of **4a** with potassium napthalenide (approximately -3.0 V reducing power) in THF gave the potassium salt of the dianion.[7] This was filtered on a frit and washed with minimal anhydrous benzene to remove naphthalene. Dissolution in MeCN and salt exchange with 2.0 equiv. of tetra-*n*-butylammonium chloride (TBACl) yielded the TBA salt and insoluble KCl. After removal of the KCl via a 10 μM syringe filter, concentration of the solution under vacuum gave the dianionic bipyrimidine in the form of a viscous oil in the presence of TBA cations. Attempts to crash out this salt via trituration were unsuccessful despite multiple efforts Therefore, the neutral charge state was deemed to be the solubility limiting state under these conditions and solubility measurements were conducted on the neutral bipyrimidine. A full list of solubility data is given in Figure S6.

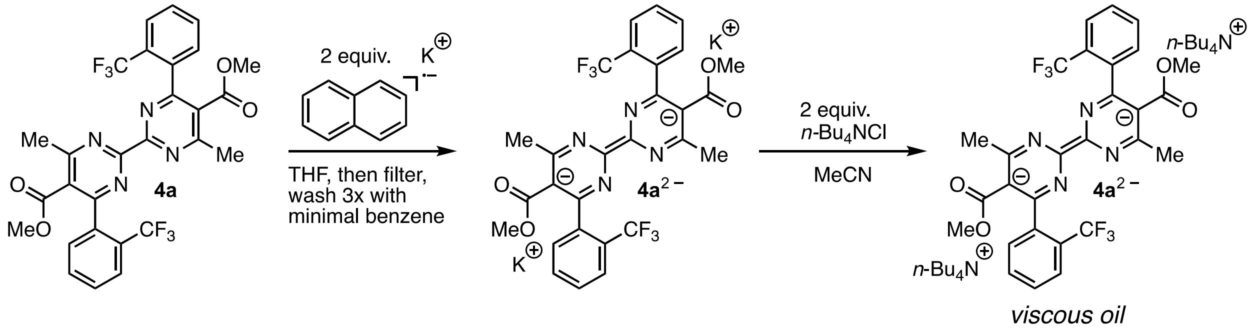

Figure S5. Evaluating solubility of the dianionic scaffold with the TBA counterion.

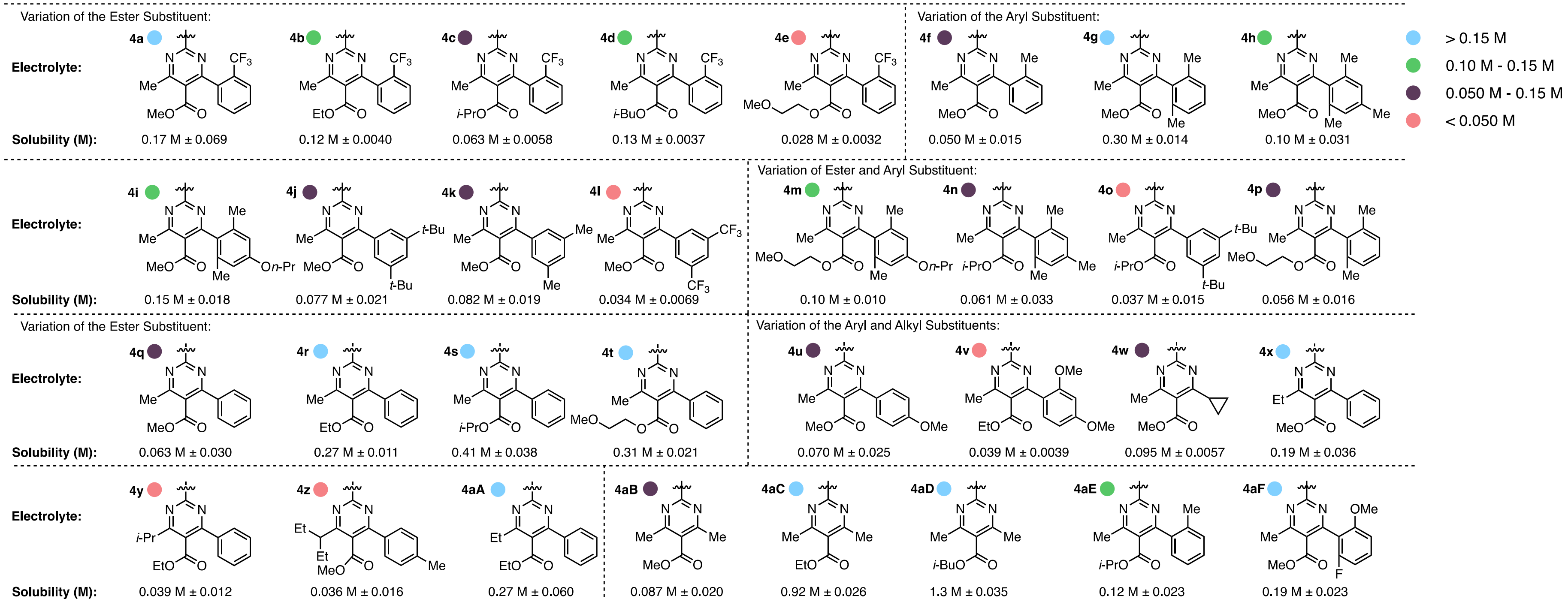

Figure S6. Solubility data of all bipyrmidine analytes. **4a**-**4p** represent the initial 2nd-generation library, **4q**-**4aA** are the 1st-generation candidates, and **4aB**-**4aF** represent the compounds chosen to evaluate the SASA threshold.

V. Computational Methods:

General

Conformational searching was performed using Macromodel by Schrodinger, release 2022-1, and the OPLS4 force field with an energetic window of 2.4 kcal/mol or 5 kcal/mol (for stability and solubility modeling, respectively) and a constant di-electric corresponding to MeCN.[8] All DFT calculations were performed using Gaussian 16, Revision C.01;[9] the level of theory was chosen based on literature precedent.[2] As previously described, the truncated bipyrimidine was found to be a robust mimic while minimizing the number of conformers/isomers; therefore, the neutral truncate shown in Figure S7 was used for the solubility modeling while the anionic truncate in Figure S7 was used for modeling stability data. DFT was used for geometry optimization at the M06-2X/6-31+G(d,p) level of theory with conductor like polarizable continuum model (CPCM) solvation in acetonitrile.[10] From the optimized geometries, single point energies were collected with M06-2X/def2-TZVP level of theory. At minimum, molecular descriptors from the following classes of conformers were gathered: Boltzmann-weighted, minimum, maximum, and lowest energy. For certain descriptors like molecular surface area or dihedral angles, range terms were also calculated to capture flexibility. NBO analysis was performed with NBO 7.0.[11] For descriptors from the Qikprop library in Schrodinger,[12] mathematical averages were used instead of Boltzmann-weighted values. The algorithms used to for modeling are available on the Sigman Group Github.[14] Select descriptors used for both solubility and stability modeling efforts are included in Tables S1 and S2 while the .xyz coordinates of the output files are included as compressed folders.

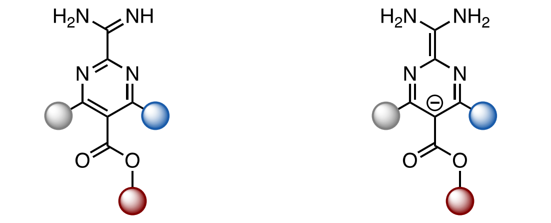

Figure S7. Structural mimics of the neutral and reduced bipyrimidines for the computational workflow.

Non-linear threshold models:

Threshold analysis was conducted according to a previously reported procedure and the code is available at https://zenodo.org/record/5227162#.ZCc6aeLMIqs or on the Sigman Group Github.[13-15] Plots were remade in Origin prior to publication. These thresholds can be divided into four quadrants: true positives, true negatives, false positives, and false negatives (Figure S8). Note that the directionality of the threshold can change the location of each quadrant, but not the relationship between quadrants. Statistical measures are included below, but caution should be used when using these statistics to describe thresholds since they are designed to penalize the presence of both false positives (points expected to be "positive" based

on the descriptor value but are experimentally determined to be "negative") and false negatives (points expected to be "negative" based on the descriptor value but are experimentally measured to be "positive"). The same can be said for the F1 score. Nonetheless, these metrics can be useful when comparing the thresholds derived from different descriptors. Equations 2 and 3 show the mathematics behind these measures and TP, TN, FP, and FN indicate true positives/negatives and false positives/negatives, respectively.

$$\text{Accuracy: } TP + TN/(TP + TN + FP + FN) \quad (2)$$

$$\text{F1 Score: } 2TP/(2TP + FP + FN) \quad (3)$$

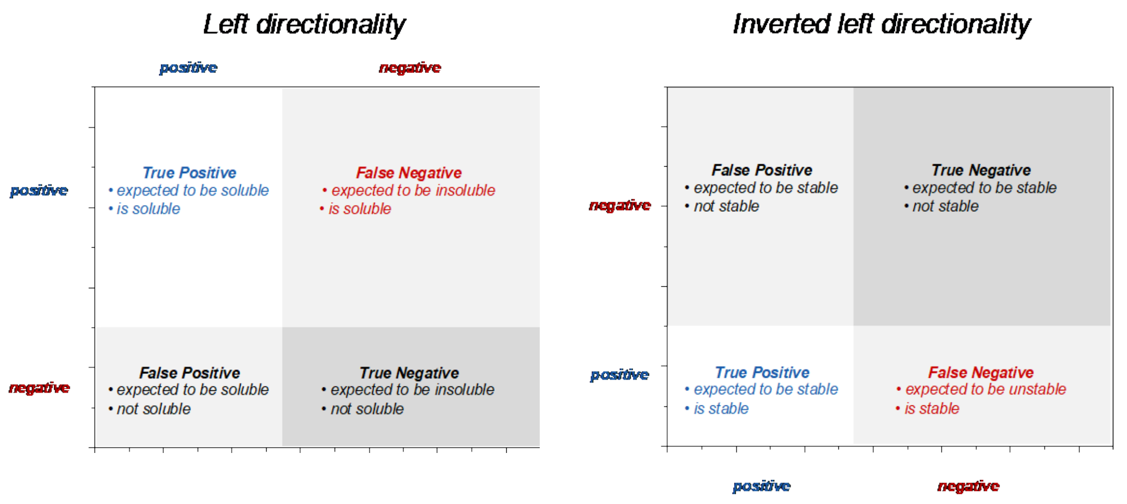

Figure S8. Four quadrants of a threshold.

Solubility modeling:

For solubility modeling efforts, a user-defined cutoff of 0.15 M was employed. This cutoff was chosen after visual inspection revealed that the solubility data could be split into two regimes: molecules with solubilities above 0.15 M and those with solubilities equal to/below 0.15 M. A class-weight descriptor of {0:1, 1:20} was used when identifying the solubility thresholds. Descriptors evaluated by the algorithm were compiled from DFT optimized structures and the Qikprop descriptor library.[12] It was found that regardless of the energy cutoff (2.4 or 5 kcal/mol) or the use of conformer clustering, the solvent accessible surface area values between the truncates and whole structures exhibited a high degree of collinearity (Figure S9). Additionally, the thresholds developed identically partition the full dataset in each case (Figure S10). Therefore, modeling efforts were carried out on the neutral, truncated structure shown in Figure S7.

Solubility Modeling:

SASA descriptor used for solubility modeling (Table S1). SASA was mathematically averaged and calculated on conformational ensembles generated with a 5 kcal/mol cutoff.

Table S1. SASA descriptors used for stability modeling.

Compound Name	Solubility (M)	SASA (Å^2)
**4A**	0.17	561.0559
**4B**	0.12	581.2619
**4C**	0.063	597.9573
**4D**	0.13	619.4454
**4E**	0.028	605.6827
**4F**	0.049	547.1034
**4G**	0.30	560.2090
**4H**	0.10	593.0260
**4I**	0.15	663.7855
**4J**	0.077	703.6844
**4K**	0.082	589.3921
**4L**	0.034	641.1562
**4M**	0.10	701.5701
**4N**	0.061	628.1067
**4O**	0.037	740.4917
**4P**	0.056	603.1652
**4Q**	0.063	530.1875
**4R**	0.27	553.6592
**4S**	0.41	567.1432
**4T**	0.31	577.5485
**4U**	0.071	568.8684
**4V**	0.039	612.3343
**4W**	0.095	492.822
**4X**	0.19	557.8481
**4Y**	0.039	595.6957
**4Z**	0.036	654.4797
**4AA**	0.27	576.0039
**4AB**	0.087	448.9345
**4AC**	0.92	476.9763
**4AD**	1.3	522.3629
**4AE**	0.12	583.8811
**4AF**	0.19	560.6652

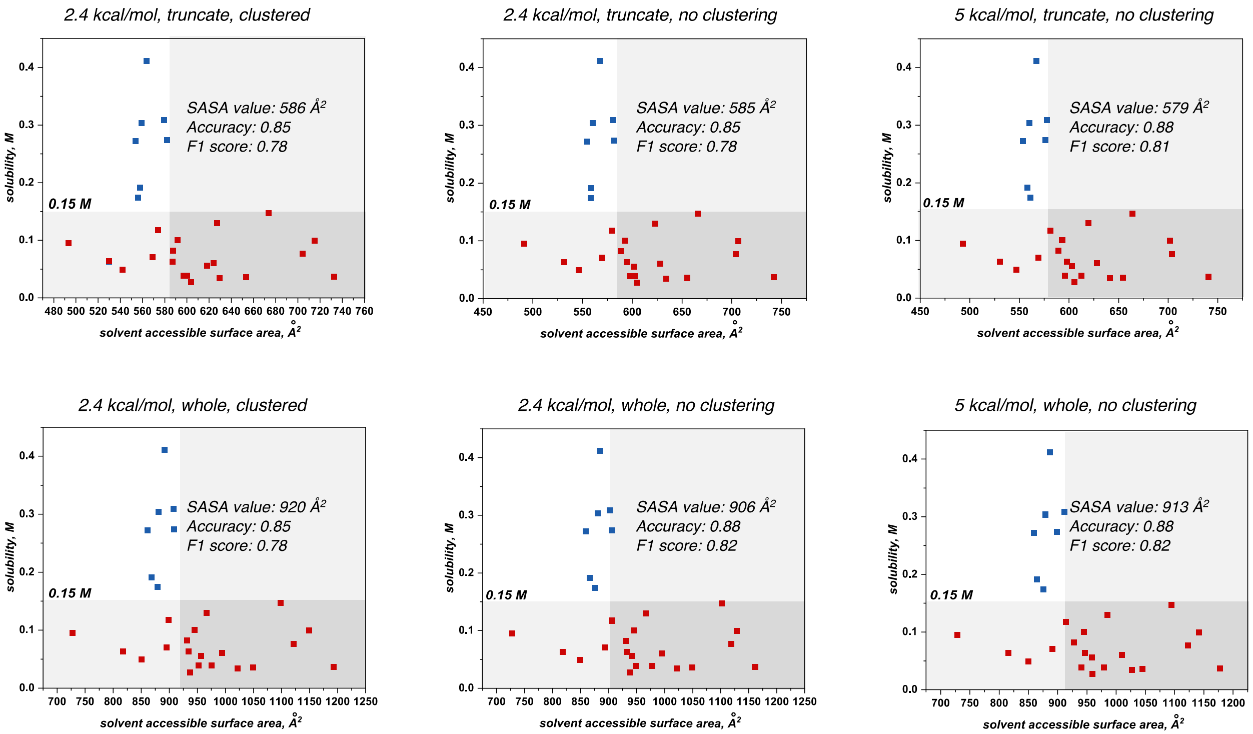

Figure S9. Impact of energetic window of the conformational search, whole or truncated structure, and conformer clustering on SASA threshold.

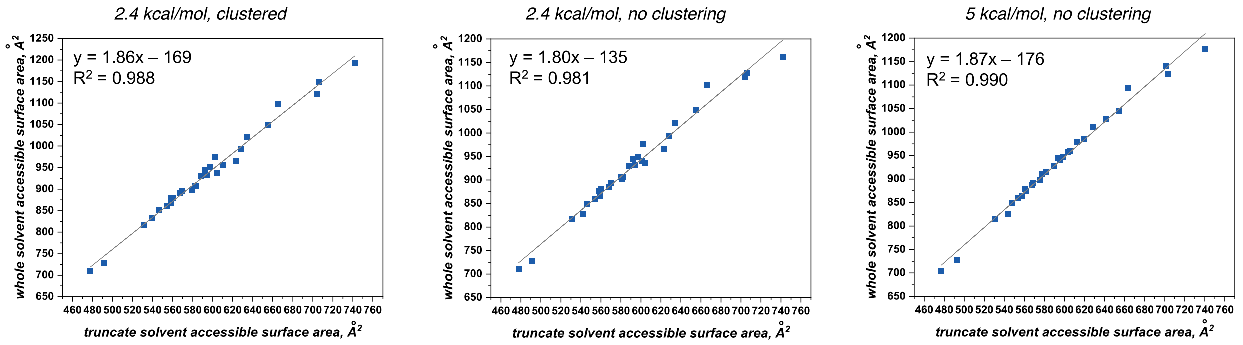

Figure S10. Comparison of solvent accessible surface area values for the truncated and whole structures with/without clustering at two different energetic cutoffs.

Stability Modeling:

Initially, stability modeling was evaluated with the described algorithm and each of the two terms previously identified to describe stability: the Boltzmann-averaged NBO charge at C_4 and C_6 and the Boltzmann-averaged maximum mode of distortion. These are shown in Figure S11A and B, respectively. As shown in Figure S11A and B, both non-linear models have multiple points within the False Negative quadrant of the graph (lower right quadrant), indicating that the algorithm is incapable of describing anolyte stability using a single parameter. We elected to plot the two parameters against each other to generate a heat map describing anolyte stability. Illustrated in Figure S11C, this heat map can direct researchers to an area of desirable chemical space to target when designing new anolytes.

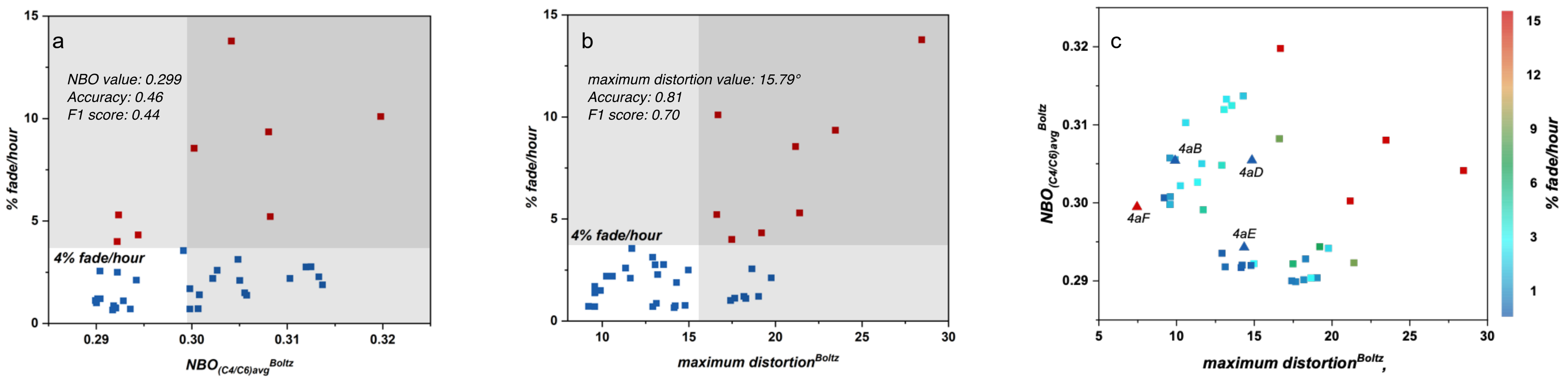

Figure S11A. Threshold generated using the Boltzmann-averaged NBO charge at C_4 and C_6. **S11B**. Threshold generated using the Boltzmann-averaged maximum distortion value. **S11C**. Heatmap comprised of both descriptors. The validation points are also included. Stable anolytes, indicated by a dark blue square or triangle, can be found by targeting the lower left corner of the map; this corresponds to minimizing both the NBO term and the maximum distortion term.

Modeling Stability of 1st and 2nd-Generation Anolytes

Tabulated selected computed parameters from the truncated reduced scaffold (Table S2). All values Boltzmann averaged unless noted as a minimum or maximum. DIST. = distortion from 180°. Θ_3 is ring puckering dihedral angle. Θ_2 is ester dihedral angle. 1st generation molecules denoted by their naming scheme from previously reported publication.[6] 4k and 4l excluded due to inability to obtain stability data (insoluble).

Table S2. Computed parameters for describing anolyte stability.

COMPOUND NAME	%FADE/ HOUR	AVG NBO$_{C4/C6}$	DISTANCE$_{C5/C7}$(Å)	DIST. (Θ_3)	DIST. (Θ_3)$_{MIN}$	DIST. (Θ_3)$_{MAX}$	DIST. (Θ_2)	DIST. (Θ_2)$_{MIN}$	DIST. (Θ_2)$_{MAX}$	TOTAL DIST.	MAX DIST.
4A$_{TRUNC}^{2-}$	0.66	0.292	1.4296	14.15	12.02	16.40	9.64	6.79	10.50	23.79	14.15
4B $_{TRUNC}^{2-}$	0.87	0.292	1.4299	13.12	11.43	16.66	9.73	6.57	14.55	22.85	13.12
4C $_{TRUNC}^{2-}$	2.5	0.292	1.4309	14.97	12.58	16.43	9.11	5.72	15.39	24.08	14.97
4D $_{TRUNC}^{2-}$	0.77	0.292	1.4299	14.78	11.09	19.13	9.05	0.40	13.69	23.83	14.78
4E $_{TRUNC}^{2-}$	0.75	0.292	1.4284	14.21	11.59	18.06	11.63	1.42	14.31	25.84	14.21
4F $_{TRUNC}^{2-}$	0.71	0.294	1.4297	12.92	11.44	15.88	9.46	1.7	13.51	22.37	12.92
4G $_{TRUNC}^{2-}$	1.7	0.300	1.4306	9.56	8.71	11.08	3.92	0.060	6.11	13.49	9.56
4H $_{TRUNC}^{2-}$	0.72	0.301	1.4311	9.20	8.24	11.68	5.43	1.18	7.07	14.63	9.20
4I $_{TRUNC}^{2-}$	2.3	0.302	1.4311	10.24	7.78	12.22	4.49	0.012	8.89	14.73	10.24
4J $_{TRUNC}^{2-}$	4.0	0.292	1.4296	17.48	15.43	20.70	16.39	13.23	18.65	33.87	17.48
4M $_{TRUNC}^{2-}$	2.1	0.305	1.4306	11.62	8.13	13.24	4.61	2.93	14.82	16.23	11.62
4N $_{TRUNC}^{2-}$	2.6	0.303	1.4314	11.34	9.10	11.34	2.78	0.39	13.50	14.13	11.34
4O $_{TRUNC}^{2-}$	5.3	0.292	1.4330	16.52	13.79	20.97	21.40	11.35	25.64	37.92	21.40
4P $_{TRUNC}^{2-}$	1.4	0.301	1.4292	9.59	6.44	14.30	8.07	0.092	13.04	17.65	9.59
4Q $_{TRUNC}^{2-}$	1.1	0.290	1.4285	17.65	17.07	20.77	15.74	12.20	16.40	33.39	17.65
4R $_{TRUNC}^{2-}$	1.0	0.290	1.4294	17.41	15.14	20.78	16.54	11.90	19.46	33.95	17.41
4S $_{TRUNC}^{2-}$	1.2	0.290	1.4308	19.03	16.81	20.48	15.07	11.96	19.31	34.10	19.03
4T $_{TRUNC}^{2-}$	1.2	0.290	1.4282	16.38	15.50	21.82	18.18	3.22	25.19	34.56	18.18
4U $_{TRUNC}^{2-}$	1.1	0.293	1.4291	18.29	17.44	22.37	16.35	12.10	17.74	34.64	18.29
4V $_{TRUNC}^{2-}$	3.6	0.299	1.4325	10.23	7.25	15.05	11.70	4.34	16.96	21.93	11.70
4W $_{TRUNC}^{2-}$	5.2	0.308	1.4297	16.61	16.16	16.93	8.26	6.28	11.63	24.87	16.61
4X $_{TRUNC}^{2-}$	2.1	0.294	1.4278	19.76	16.19	24.25	14.67	8.51	17.71	34.43	19.76
4Y $_{TRUNC}^{2-}$	8.6	0.300	1.4278	21.16	15.24	28.00	14.99	5.85	20.74	36.15	21.16
4Z $_{TRUNC}^{2-}$	9.4	0.308	1.4275	23.46	15.62	30.37	11.96	3.76	18.87	35.42	23.46
4AA $_{TRUNC}^{2-}$	4.3	0.294	1.4287	19.20	13.36	24.46	15.70	6.03	20.72	34.90	19.20
4AB $_{TRUNC}^{2-}$	2.3	0.306	1.4270	10.70	9.22	10.95	3.36	1.86	9.25	14.05	10.70
4AC $_{TRUNC}^{2-}$	1.5	0.305	1.4314	9.89	9.11	10.19	5.71	4.85	10.03	15.61	9.90
4AD $_{TRUNC}^{2-}$	1.6	0.305	1.4314	10.48	9.42	12.14	4.61	1.868	9.64	15.10	10.48
4AE $_{TRUNC}^{2-}$	1.6	0.294	1.4319	14.34	11.20	15.97	11.19	3.263	18.48	25.54	14.35
4AF $_{TRUNC}^{2-}$	13.0	0.299	1.4318	7.45	4.15	9.45	4.79	3.333	10.50	12.25	7.45
1ST GEN 4D $_{TRUNC}^{2-}$	2.6	0.290	1.4344	18.64	18.02	19.47	16.60	15.318	18.32	35.24	18.64
1ST GEN 4N $_{TRUNC}^{2-}$	13.8	0.304	1.4249	28.45	27.77	36.95	16.59	1.822	17.78	45.04	28.45
1ST GEN 4P $_{TRUNC}^{2-}$	1.4	0.306	1.4324	9.56	8.33	9.96	8.29	6.548	11.76	17.85	9.56
1ST GEN 4Q $_{TRUNC}^{2-}$	3.1	0.305	1.4241	12.91	7.37	29.88	3.48	0.149	12.46	16.39	12.91
1ST GEN 4R $_{TRUNC}^{2-}$	2.2	0.310	1.4307	10.58	10.21	18.12	9.99	4.625	10.96	20.57	10.58
1ST GEN 4S $_{TRUNC}^{2-}$	2.8	0.312	1.4299	13.54	6.17	19.16	6.44	0.411	14.77	19.98	13.54
1ST GEN 4T $_{TRUNC}^{2-}$	2.8	0.312	1.4309	13.05	3.90	18.21	7.70	0.019	22.95	20.75	13.05
1ST GEN 4U $_{TRUNC}^{2-}$	2.3	0.313	1.4302	13.20	7.98	18.45	9.17	0.734	14.32	22.37	13.20
1ST GEN 4V $_{TRUNC}^{2-}$	1.9	0.314	1.4312	14.28	0.29	19.18	5.26	0.152	25.85	19.54	14.28
1ST GEN 4W $_{TRUNC}^{2-}$	10.1	0.320	1.4282	16.68	9.18	19.86	6.48	0.942	17.90	23.16	16.68

VI. Characterization of Compounds:

2,6-dimethyl-4-propoxybenzaldehyde (1)

1

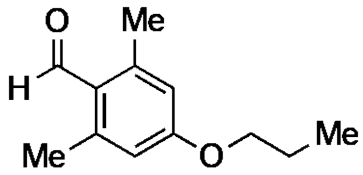

Aldehyde **1** was prepared by adding 4-hydroxy-2,6-dimethylbenzaldehyde (1.0 equiv.) to a round bottom flask containing a stir bar and DMF (0.4 M). K_2CO_3 (1.2 equiv.) was added to the flask, followed by *n*-propyl bromide (1.0 equiv.). The reaction was heated to 75°C for four hours at which point it was cooled to room temperature. DMF was removed by azeotroping with heptane until a brown solid was left in the flask. At this point, the solid was dissolved with EtOAc and added to a separatory funnel containing deionized water. The organic layer was washed three times with deionized water to remove residual DMF, followed by three back extractions of the aqueous layer with EtOAc. The organic layers were dried with $MgSO_4$, filtered on a glass frit, and concentrated. ^{1}H-NMR analysis revealed the presence of a small amount of residual DMF; further azeotropes with heptane removed the rest of the DMF. The desired product was isolated as a brown semi-crystalline solid (18 g, 95% yield).

Analytical data for **1**:

^{1}H NMR (500 MHz, Chloroform-*d$_1$*) δ 10.46 (s, 1H, ***H***CO), 6.58 (s, 2H, *m-**ArH***), 3.96 (t, *J* = 6.5 Hz, 2H, *p*-ArO***CH$_2$***CH$_2$CH$_3$), 2.59 (s, 6H, *o-**ArCH$_3$***), 1.81 (h, *J* = 7.1 Hz, 2H, *p*-ArOCH$_2$***CH$_2$***CH$_3$), 1.04 (t, *J* = 7.4 Hz, 3H, *p*-ArOCH$_2$CH$_2$***CH$_3$***).

^{13}C NMR (126 MHz, Chloroform-*d$_1$*) δ 191.70, 162.54, 144.59, 125.91, 115.47, 69.61, 22.59, 21.20, 10.57.

IR (thin film, cm^{-1}): 2968, 2870, 1681, 1592, 1468, 1308, 1286, 1151, 1125, 1058, 841, 798, 637.

HRMS *m/z* **calculated for $C_{12}H_{17}O_2^+$:** 193.1223 **found:** 193.1229.

NMR Spectra (^{1}H, ^{13}C): S82

methyl 6-methyl-2-oxo-4-(2-(trifluoromethyl)phenyl)-1,2,3,4-tetrahydropyrimidine-5-carboxylate (1a)

1a

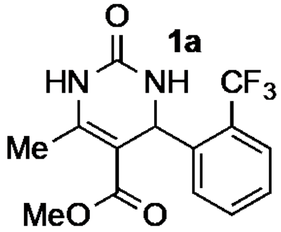

Prepared according to General Procedure A. Collected after filtration as a white solid (20 g, 63% yield). Characterization matched previously reported data.[2]

ethyl 6-methyl-2-oxo-4-(2-(trifluoromethyl)phenyl)-1,2,3,4-tetrahydropyrimidine-5-carboxylate (1b)

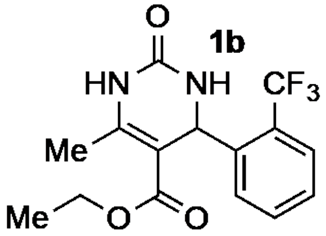

1b

Prepared according to General Procedure A. Collected after filtration as a white solid (15 g, 56% yield).

Analytical data for **1b**:

^{1}H NMR (500 MHz, Dimethylsulfoxide-*d_6*) δ 9.34 (bs, 1H, ***NH***), 7.70-7.65 (m, 2H, ***ArH***), 7.52-7.45 (m, 2H, ***ArH***), 7.30 (bs, 1H, ***NH***), 5.58 (bs, 1H, ***CH***), 3.85 (q, *J* = 7.1 Hz, 2H, $CO_2\boldsymbol{CH_2}CH_3$), 2.34 (s, 3H, $–\boldsymbol{CH_3}$), 0.88 (t, *J* = 7.1 Hz, 3H, $CO_2CH_2\boldsymbol{CH_3}$).

^{13}C NMR (126 MHz, Dimethylsulfoxide-*d_6*) δ 164.74, 150.99, 149.69, 143.11, 133.35, 128.50, 128.03, 125.70, 125.66, 123.17, 98.09, 58.95, 50.55, 17.70, 13.70.

^{19}F NMR (282 MHz, Dimethysulfoxide-*d_6*) -55.76.

IR (thin film, cm^{-1}): 3221, 3095, 1696, 1641, 1443, 1312, 1231, 1094, 1039, 766, 671.

HRMS ***m/z*** **calculated for $C_{15}H_{16}F_3N_2O_3^+$:** 329.1108 **found:** 329.1112.

NMR Spectra (^{1}H, ^{13}C, ^{19}F): S83-S84

isopropyl 6-methyl-2-oxo-4-(2-(trifluoromethyl)phenyl)-1,2,3,4-tetrahydropyrimidine-5-carboxylate (1c)

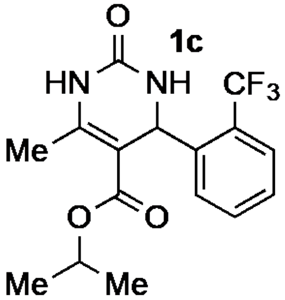

1c

Prepared according to General Procedure A. Product was collected after filtration as a white solid (14 g, 41% yield).

Analytical data for **1c**:

^{1}H NMR (500 MHz, Dimethylsulfoxide-*d_6*) δ 9.31 (bs, 1H, ***NH***), 7.72-7.64 (m, 2H, ***ArH***), 7.51-7.45 (m, 2H, ***ArH***), 7.19 (bs, 1H, ***NH***), 5.56 (bs, 1H, ***CH***), 4.74 (hept, *J* = 6.3 Hz, 1H, $CO_2\boldsymbol{CH}(CH_3)_2$), 2.34 (s, 3H, $–\boldsymbol{CH_3}$), 1.04 (d, *J* = 6.2 Hz, 3H, $CO_2CH(\boldsymbol{CH_3})_2$), 0.64 (d, *J* = 6.2 Hz, 3H, $CO_2CH(\boldsymbol{CH_3})_2$).

^{13}C NMR (126 MHz, Dimethylsulfoxide-*d_6*) δ 164.22, 150.85, 149.62, 142.98, 133.29, 128.42, 127.97, 125.74, 125.68, 125.63, 98.02, 65.87, 50.61, 21.40, 20.89, 17.67.

^{19}F NMR (282 MHz, Dimethysulfoxide-*d_6*) -55.61.

IR (thin film, cm^{-1}): 3220, 3102, 2980, 1697, 1640, 1453, 1312, 1233, 1088, 767, 660.

HRMS *m/z* **calculated for $C_{16}H_{18}F_3N_2O_3^+$:** 343.1264 **found:** 343.1272.

NMR Spectra (^{1}H, ^{13}C, ^{19}F): S85-S86

isobutyl 6-methyl-2-oxo-4-(2-(trifluoromethyl)phenyl)-1,2,3,4-tetrahydropyrimidine-5-carboxylate (1d)

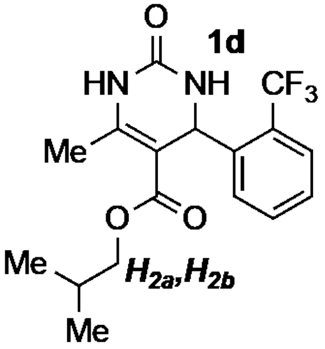

Prepared according to General Procedure A. The solvent was removed under reduced pressure to yield a yellow residue. This was triturated with EtOAc:Hexanes to yield, after filtering on a glass frit, an off-white powder (15 g, 42% yield).

Analytical data for **1d**:

^{1}H NMR (500 MHz, Dimethylsulfoxide-*d$_6$*) δ 9.37 (bs, 1H, ***NH***), 7.72-7.64 (m, 2H, ***ArH***), 7.52-7.45 (m, 2H, ***ArH***), 7.23 (bs, 1H, ***NH***), 5.57 (d, *J* = 3.0 Hz, 1H, ***CH***), 3.71 (dd, *J* = 10.7, 7.0 Hz, 1H, $CO_2\boldsymbol{CH_{2a}}CH_{2b}CH(CH_3)_2$), 3.56 (dd, *J* = 10.7, 6.4 Hz, 1H, $CO_2CH_{2a}\boldsymbol{CH_{2b}}CH(CH_3)_2$), 2.36 (s, 3H, $-\boldsymbol{CH_3}$), 1.61 (*apparent* hept, *J* = 6.7 Hz, 1H, $CO_2CH_{2a}CH_{2b}\boldsymbol{CH}(CH_3)_2$), 0.59 (d, *J* = 6.7 Hz, 3H, $CO_2CH_{2a}CH_{2b}CH(\boldsymbol{CH_3})_2$), 0.52 (d, *J* = 6.6 Hz, 3H, $CO_2CH_{2a}CH_{2b}CH(\boldsymbol{CH_3})_2$).

^{13}C NMR (126 MHz, Dimethylsulfoxide-*d$_6$*) δ 164.76, 151.02, 150.08, 142.90, 133.37, 128.36, 128.07, 125.90, 125.84, 123.19, 97.85, 69.22, 50.47, 26.97, 18.52, 18.48, 17.83.

^{19}F NMR (282 MHz, Dimethysulfoxide-*d$_6$*) -55.79.

IR (thin film, cm^{-1}): 3442, 2959, 1639, 1640, 1311, 1232, 1165, 1116, 770.

HRMS *m/z* **calculated for $C_{17}H_{20}F_3N_2O_3^+$:** 357.1421 **found:** 357.1423.

NMR Spectra (^{1}H, ^{13}C, ^{19}F): S87-S88

2-methoxyethyl 6-methyl-2-oxo-4-(2-(trifluoromethyl)phenyl)-1,2,3,4-tetrahydropyrimidine-5-carboxylate (1e)

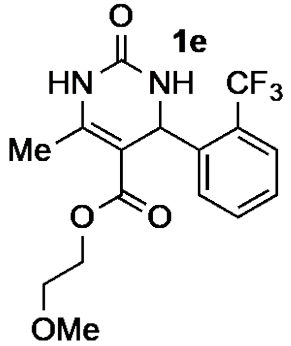

Prepared according to General Procedure A. No precipitate was observed after cooling the reaction. The reaction was concentrated under reduced pressure to remove most of the solvent. The crude mixture was dissolved in DCM and poured into a separatory funnel. Deionized water was added to the funnel and the biphasic mixture was extracted three times with DCM. The organic layers were dried over Na_2SO_4, filtered, and concentrated to give a viscous gold oil. This oil was triturated with EtOAc:Hexanes to yield a XX solid that was filtered on a glass frit to afford the product as an off-white powder (22 g, 63% yield).

Analytical data for **1e**:

^{1}H NMR (500 MHz, Dimethylsulfoxide-d_6) δ 9.37 (bs, 1H, ***NH***), 7.71-7.64 (m, 2H, ***ArH***), 7.52-7.45 (m, 2H, ***ArH***), 7.29 (bs, 1H, ***NH***), 5.57 (d, J = 2.9 Hz, 1H, ***CH***), 3.99-3.91 (m, 2H, $CO_2\boldsymbol{CH_2}CH_2OCH_3$), 3.27 (t, J = 4.8 Hz, 2H, $CO_2CH_2\boldsymbol{CH_2}OCH_3$), 3.05 (s, 3H, $CO_2CH_2CH_2\boldsymbol{OCH_3}$), 2.35 (s, 3H, $–\boldsymbol{CH_3}$).

^{13}C NMR (126 MHz, Dimethylsulfoxide-d_6) δ 164.74, 151.01, 150.07, 142.89, 133.33, 128.45, 128.02, 125.75, 125.71, 125.67, 97.92, 69.50, 62.16, 57.77, 50.45, 17.83.

^{19}F NMR (282 MHz, Dimethysulfoxide-d_6) -55.81.

IR (thin film, cm^{-1}): 3425, 2952, 1694, 1640, 1313, 1234, 1100, 1029, 763, 551.

HRMS *m/z* **calculated for $C_{16}H_{18}F_3N_2O_4^+$:** 359.1213 **found:** 359.1215.

NMR Spectra (^{1}H, ^{13}C, ^{19}F): S89-S90

methyl 6-methyl-2-oxo-4-(*o*-tolyl)-1,2,3,4-tetrahydropyrimidine-5-carboxylate (1f)

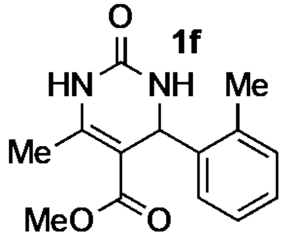

Prepared according to General Procedure A. After filtration, the product was collected as a white powder (15 g, 75% yield). Characterization matched previously reported data.[2]

methyl 4-(2,6-dimethylphenyl)-6-methyl-2-oxo-1,2,3,4-tetrahydropyrimidine-5-carboxylate (1g)

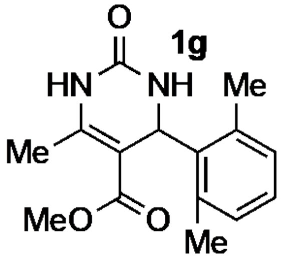

Prepared according to General Procedure A. Product was collected as a white powder (12 g, 55% yield).

Analytical data for **1g**:

^{1}H NMR (500 MHz, Dimethylsulfoxide-*d*$_6$) δ 9.10 (bs, 1H, ***NH***), 7.35 (bs, 1H, ***NH***), 7.03-6.97 (m, 1H, *p*-***ArH***), 6.97-6.91 (m, 2H, *m*-***ArH***), 5.82 (bs, 1H, ***CH***), 3.35 (s, 3H, CO_2***CH_3***), 2.34 (s, 6H, *o*-***ArCH_3***), 2.14 (s, 3H, –***CH_3***).

^{13}C NMR (126 MHz, Dimethylsulfoxide-*d*$_6$) δ 165.99, 150.68, 146.68, 139.79, 136.69, 129.10, 126.81, 96.45, 51.09, 50.29, 19.16, 17.58.

IR (thin film, cm^{-1}): 3350, 3109, 2968, 1690, 1636, 1432, 1228, 1097, 765, 655.

HRMS *m/z* **calculated for $C_{15}H_{19}N_2O_3^+$:** 275.1390 **found:** 275.1403.

NMR Spectra (^{1}H, ^{13}C): S91

methyl 4-mesityl-6-methyl-2-oxo-1,2,3,4-tetrahydropyrimidine-5-carboxylate (1h)

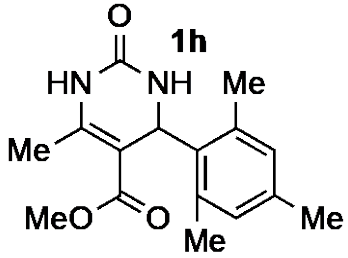

Prepared according to General Procedure A. Product was collected as a white solid after filtration (19 g, 66% yield). Characterization matched previously reported data.[2]

methyl 4-(2,6-dimethyl-4-propoxyphenyl)-6-methyl-2-oxo-1,2,3,4-tetrahydropyrimidine-5-carboxylate (1i)

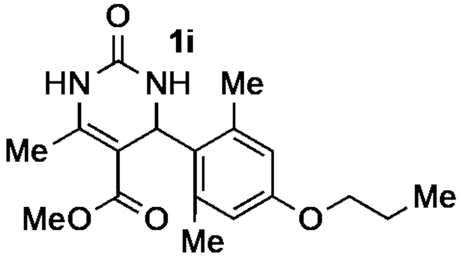

Prepared according to General Procedure A. Product collected after filtration as a light orange solid (7.5 g, 36% yield).

Analytical data for **1i**:

^{1}H NMR (500 MHz, Dimethylsulfoxide-*d$_6$*) δ 9.05 (bs, 1H, ***NH***), 7.26 (bs, 1H, ***NH***), 6.52 (s, 2H, *m*-***ArH***), 5.73 (s, 1H, ***CH***), 3.86 (t, *J* = 6.5 Hz, 2H, *p*-ArO***CH$_2$***CH$_2$CH$_3$), 3.37 (s, 3H, CO$_2$***CH$_3$***), 2.29 (s, 6H, *o*-***ArCH$_3$***), 2.12 (s, 3H, –***CH$_3$***), 1.68 (h, *J* = 7.1 Hz, 2H, *p*-ArOCH$_2$***CH$_2$***CH$_3$), 0.95 (t, *J* = 7.4 Hz, 3H, *p*-ArOCH$_2$CH$_2$***CH$_3$***).

^{13}C NMR (126 MHz, Dimethylsulfoxide-*d$_6$*) δ 166.08, 156.79, 150.69, 146.20, 138.20, 132.29, 96.85, 68.50, 50.57, 50.32, 22.09, 17.53, 10.40.

IR (thin film, cm^{-1}): 3227, 2945, 1634, 1644, 1307, 1232, 1155, 1092, 770, 656.

HRMS *m/z* **calculated for $C_{18}H_{25}N_2O_4^+$:** 333.1809 **found:** 333.1817.

NMR Spectra (^{1}H, ^{13}C): S92

methyl 4-(3,5-di-*tert*-butylphenyl)-6-methyl-2-oxo-1,2,3,4-tetrahydropyrimidine-5-carboxylate (1j)

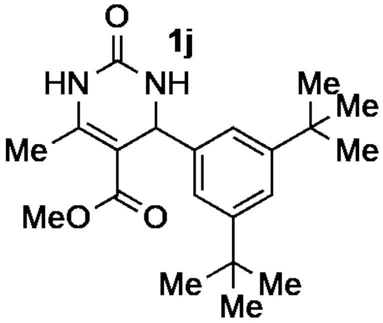

Prepared according to General Procedure A. Product collected after filtration as a white powder (21 g, 73% yield).

Analytical data for **1j**:

^{1}H NMR (500 MHz, Dimethylsulfoxide-*d$_6$*) δ 9.18 (bs, 1H, ***NH***), 7.71 (bs, 1H, ***NH***), 7.27 (t, *J* = 1.7 Hz, 1H, *p*-***ArH***), 7.10 (d, *J* = 1.8 Hz, 2H, *o*-***ArH***), 5.14 (d, *J* = 3.4 Hz, 1H, ***CH***), 3.56 (s, 3H, CO$_2$***CH$_3$***), 2.22 (s, 3H, –***CH$_3$***), 1.25 (s, 18H, *m*-**ArC(*CH$_3$*)$_3$**).

^{13}C NMR (126 MHz, Dimethylsulfoxide-*d$_6$*) δ 165.90, 152.49, 150.12, 148.14, 144.01, 120.73, 119.95, 99.50, 53.99, 50.71, 34.40, 31.21, 17.70.

IR (thin film, cm^{-1}): 3355, 2962, 1693, 1635, 1455, 1224, 1092, 790, 556.

HRMS *m/z* **calculated for $C_{21}H_{31}N_2O_3^+$:** 359.2329 **found:** 359.2336.

NMR Spectra (^1H, ^{13}C): S93

methyl 4-(3,5-dimethylphenyl)-6-methyl-2-oxo-1,2,3,4-tetrahydropyrimidine-5-carboxylate (1k)

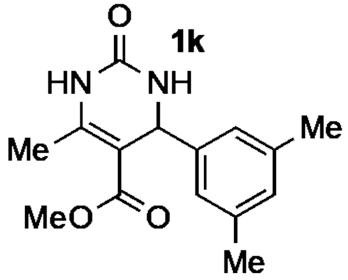

Prepared according to General Procedure A. Product collected after filtration as white powder (18 g, 87% yield).

Analytical data for **1k**:

^{1}H NMR (500 MHz, Dimethylsulfoxide-*d6*) δ 9.14 (bs, 1H, ***NH***), 7.65 (bs, 1H, ***NH***), 6.87 (s, 1H, *p-**ArH***), 6.82 (s, 2H, *o-**ArH***), 5.08 (d, *J* = 3.3 Hz, 1H, ***CH***), 3.53 (s, 3H, CO_2CH_3), 2.25 (s, 3H, –***CH3***), 2.23 (s, 6H, *m-**ArCH3***).

^{13}C NMR (126 MHz, Dimethylsulfoxide-*d6*) δ 165.87, 152.13, 148.44, 144.62, 137.30, 128.71, 123.90, 98.98, 53.78, 50.73, 21.04, 17.85.

IR (thin film, cm^{-1}): 3344, 3215, 3101, 2951, 1689, 1639, 1456,1326, 1230, 1098, 794, 631.

HRMS *m/z* **calculated for $C_{15}H_{19}N_2O_3^+$:** 275.1390 **found:** 275.1402.

NMR Spectra (^1H, ^{13}C): S94

methyl 4-(3,5-bis(trifluoromethyl)phenyl)-6-methyl-2-oxo-1,2,3,4-tetrahydropyrimidine-5-carboxylate (1l)

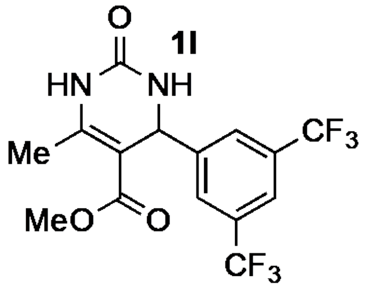

Prepared according to General Procedure A. No precipitate was observed after cooling to room temperature. The solvent was removed under reduced pressure and the resulting residue was dissolved in DCM. This was poured into a separatory funnel containing deionized water and three extractions with DCM were done. The organic layers were combined and dried over Na_2SO_4; post filtration and concentration, the residue was triturated with EtOAc:Hexanes to yield a solid that was filtered on a glass frit. The product was collected as an off-white powder with a blueish tint (31 g, 51% yield). *Minor impurities were present, but other characterization data was consistent with the structure and the material was carried forward.*

Analytical data for **1l**:

^{1}H NMR (500 MHz, Dimethylsulfoxide-*d$_6$*) δ 9.43 (bs, 1H, ***NH***), 8.03 (bs, 1H, ***NH***), 7.93 (s, 1H, *p*-***ArH***), 7.84 (s, 2H, *o*-***ArH***), 5.39 (d, *J* = 3.3 Hz, 1H, ***CH***), 3.54 (s, 3H, CO_2***CH$_3$***), 2.28 (s, 3H, –***CH$_3$***).

^{13}C NMR (126 MHz, Dimethylsulfoxide-*d$_6$*) δ 165.54, 151.65, 150.03, 147.92, 130.85, 130.59, 130.33, 130.07, 129.81, 126.95, 126.76, 126.48, 124.50, 124.31, 122.33, 122.14, 121.41, 121.38, 121.35, 119.98, 97.61, 53.38, 50.90, 17.93.

^{19}F NMR (282 MHz, Dimethylsulfoxide-*d$_6$*) δ -61.39.

IR (thin film, cm^{-1}): 3436, 3296, 1654, 1591, 1372, 1276, 1169, 1117, 1097, 896, 680.

HRMS ***m/z*** **calculated for $C_{15}H_{13}F_6N_2O_3^+$:** 383.0825 **found:** 383.0835.

NMR Spectra (^{1}H, ^{13}C, ^{19}F): S95-S96

2-methoxyethyl 4-(2,6-dimethyl-4-propoxyphenyl)-6-methyl-2-oxo-1,2,3,4-tetrahydropyrimidine-5-carboxylate (1m)

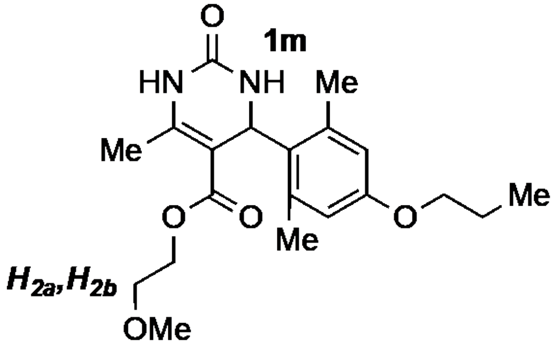

Prepared according to General Procedure A. The product did not precipitate after cooling the reaction and the reaction mixture was directly concentrated to remove most of the solvent. The remaining contents in the flask were dissolved in DCM and poured into a separatory funnel containing deionized water. The biphasic mixture was extracted three times with DCM. The organic layers were combined, dried over Na_2SO_4, filtered, and concentrated to yield an orange residue. This residue was triturated with hexanes to yield the product as a light orange powder (12 g, 44% yield).

Analytical data for **1m**:

^{1}H NMR (500 MHz, Dimethylsulfoxide-*d$_6$*) δ 9.05 (bs, 1H, ***NH***), 7.27 (bs, 1H, ***NH***), 6.52 (s, 2H, *m*-***ArH***), 5.72 (s, 1H, ***CH***), 3.91-3.87 (m, 2H, CO_2***CH$_2$***$CH_{2a}CH_{2b}CH_3$), 3.86 (t, *J* = 6.5 Hz, 2H, *p*-ArO***CH$_2$***CH_2CH_3), 3.29-3.24 (m, 1H, CO_2CH_2***CH$_{2a}$***$CH_{2b}OCH_3$), 3.25-3.20 (m, 1H, $CO_2CH_2CH_{2a}$***CH$_{2b}$***OCH_3), 3.14 (s, 3H, $CO_2CH_2CH_{2a}CH_{2b}$***OCH$_3$***), 2.30 (s, 6H, *o*-***ArCH$_3$***), 2.13 (s, 3H, –***CH$_3$***), 1.68 (h, *J* = 7.1 Hz, 2H, *p*-$ArOCH_2$***CH$_2$***CH_3), 0.95 (t, *J* = 7.0 Hz, 3H, *p*-$ArOCH_2CH_2$***CH$_3$***).

^{13}C NMR (126 MHz, Dimethylsulfoxide-*d$_6$*) δ 165.58, 156.83, 150.68, 146.63, 138.22, 132.34, 96.85, 69.67, 68.52, 62.02, 57.83, 50.53, 22.08, 17.60, 10.39.

IR (thin film, cm^{-1}): 3357, 3111, 2968, 1689, 1636, 1453, 1302, 1226, 1100, 795, 656.

HRMS ***m/z*** **calculated for $C_{20}H_{29}N_2O_5^+$:** 377.2071 **found:** 377.2076.

NMR Spectra (^{1}H, ^{13}C): S97

isopropyl 4-mesityl-6-methyl-2-oxo-1,2,3,4-tetrahydropyrimidine-5-carboxylate (1n)

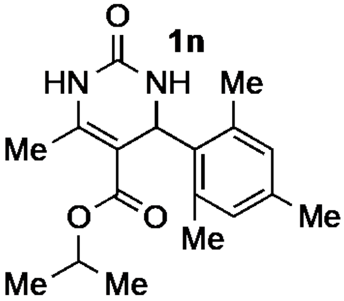

Prepared according to General Procedure A. Product was collected as a white solid (12 g, 46%)

Analytical data for **1n**:

^{1}H NMR (500 MHz, Dimethylsulfoxide-*d$_6$*) δ 8.99 (bs, 1H, ***NH***), 7.25 (bs, 1H, ***NH***), 6.76 (s, 2H, *m*-***ArH***), 5.73 (s, 1H, ***CH***), 4.67 (hept, *J* = 6.3 Hz, 1H, CO_2***CH***$(CH_3)_2$), 2.30 (s, 6H, *o*-***ArCH$_3$***), 2.17 (s, 3H, ***CH$_3$***), 2.11 (s, 3H, ***CH$_3$***), 1.05 (d, *J* = 6.2 Hz, 3H, CO_2CH(***CH$_3$***$)_2$), 0.62 (d, *J* = 6.2 Hz, 3H, CO_2CH(***CH$_3$***$)_2$).

^{13}C NMR (126 MHz, Dimethylsulfoxide-*d$_6$*) δ 165.05, 150.73, 146.17, 137.08, 136.45, 135.52, 97.34, 65.80, 50.87, 21.54, 20.66, 20.33, 17.43.

IR (thin film, cm^{-1}): 3357, 3109, 2968, 1688, 1639, 1454, 1292, 1230, 1087, 793, 654.

HRMS ***m/z*** **calculated for $C_{18}H_{25}N_2O_3^+$:** 317.1860 **found:** 317.1870.
NMR Spectra (^{1}H, ^{13}C): S98

isopropyl 4-(3,5-di-*tert*-butylphenyl)-6-methyl-2-oxo-1,2,3,4-tetrahydropyrimidine-5-carboxylate (1o)

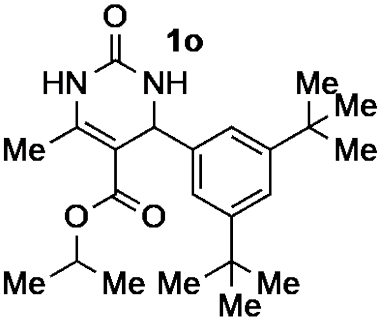

Prepared according to General Procedure A. Collected after filtration as a white powder (24 g, 62% yield)

Analytical data for **1o**:

^{1}H NMR (500 MHz, Dimethylsulfoxide-*d$_6$*) δ 9.12 (bs, 1H, ***NH***), 7.67 (s, 1H, ***NH***), 7.27 (s, 1H, *p*-***ArH***), 7.09 (s, 2H, *o*-***ArH***), 5.12 (d, *J* = 3.3 Hz, 1H, ***CH***), 4.83 (hept, *J* = 6.2 Hz, 1H, CO_2***CH***$(CH_3)_2$), 2.23 (s, 3H, –***CH$_3$***), 1.25 (s, 18H, *m*-**ArC(*CH$_3$*)$_3$**), 1.17 (d, *J* = 6.2 Hz, 3H, CO_2CH(***CH$_3$***$)_2$), 1.01 (d, *J* = 6.2 Hz, 3H, CO_2CH(***CH$_3$***$)_2$).

^{13}C NMR (126 MHz, Dimethylsulfoxide-*d$_6$*) δ 164.96, 152.40, 150.05, 147.78, 144.25, 120.73, 120.02, 99.97, 66.24, 54.16, 34.39, 31.22, 21.78, 21.49, 17.63.

IR (thin film, cm^{-1}): 3232, 2952, 1699, 1652, 1477, 1204, 1225, 1085, 793.

HRMS ***m/z*** **calculated for $C_{23}H_{35}N_2O_3^+$:** 387.2642 **found:** 387.2639.

NMR Spectra (^{1}H, ^{13}C): S99

2-methoxyethyl 4-(2,6-dimethylphenyl)-6-methyl-2-oxo-1,2,3,4-tetrahydropyrimidine-5-carboxylate (1p)

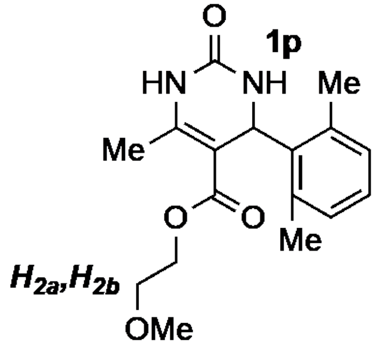

Prepared according to General Procedure A. Collected after filtration as a white powder (12 g, 46% yield).

Analytical data for **1p**:

^{1}H NMR (500 MHz, Dimethylsulfoxide-*d$_6$*) δ 9.10 (bs, 1H, ***NH***), 7.36 (bs, 1H, ***NH***), 7.04-6.97 (m, 1H, *p-**ArH***), 6.96-6.93 (m, 2H, *m-**ArH***), 5.81 (s, 1H, ***CH***), 3.93-3.82 (m, 2H, CO_2***CH_2***$CH_{2a}CH_{2b}OCH_3$), 3.28-3.21 (m, 1H, CO_2CH_2***CH_{2a}***$CH_{2b}OCH_3$), 3.21-3.15 (m, 1H, $CO_2CH_2CH_{2a}$***CH_{2b}***OCH_3), 3.13 (s, 3H, $CO_2CH_2CH_{2a}CH_{2b}$***OCH_3***), 2.34 (s, 6H, *o-**ArCH$_3$***), 2.15 (s, 3H, –***CH$_3$***).

^{13}C NMR (126 MHz, Dimethylsulfoxide-*d$_6$*) δ 165.48, 150.66, 147.11, 139.81, 136.72, 129.10, 126.78, 96.44, 69.59, 62.02, 57.82, 51.04, 19.25, 17.65.

IR (thin film, cm^{-1}): 3352, 3108, 2965, 1688, 1634, 1456, 1295, 1233, 1104, 952, 778, 655.

HRMS *m/z* **calculated for $C_{17}H_{23}N_2O_4^+$:** 319.1652 **found:** 319.1667.

NMR Spectra (^{1}H, ^{13}C): S100

methyl 4,6-dimethyl-2-oxo-1,2,3,4-tetrahydropyrimidine-5-carboxylate (1aB)

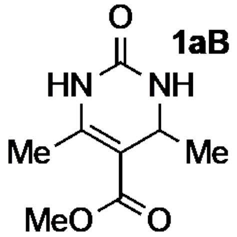

Prepared according to General Procedure A. Acetaldehyde was distilled directly before use and collected in a pear flask chilled in a dry ice:acetone bath. The product did not precipitate from solution after the reaction and the solvent was removed under vacuum. This gave a blue suspension that was dissolved in DCM and poured into a separatory funnel containing deionized water. After three extractions with DCM, the organic layers were dried over Na_2SO_4, filtered, and concentrated to yield a fluffy white solid. A suspension of this solid was made in EtOAc:Hexanes and filtered on a glass frit to give the desired product as a white powder (11 g, 57%).

Analytical data for **1aB**:

^{1}H NMR (500 MHz, Dimethylsulfoxide-*d$_6$*) δ 8.99 (bs, 1H, ***NH***), 7.20 (bs, 1H, ***NH***), 4.11 (qd, *J* = 6.2, 3.1 Hz, 1H, ***CH***), 3.60 (s, 3H, CO_2***CH_3***), 2.15 (s, 3H, –***CH$_3$***), 1.09 (d, *J* = 6.3 Hz, 3H, –***CH$_3$***). XX revisit splitting

^{13}C NMR (126 MHz, Dimethylsulfoxide-*d$_6$*) δ 165.81, 152.44, 147.95, 100.27, 50.72, 46.26, 23.41, 17.69.

IR (thin film, cm^{-1}): 3233, 3106, 2951, 1700, 1652, 1489, 1432, 1326, 1229, 1191, 1099, 800, 760, 642.

HRMS *m/z* **calculated for $C_8H_{13}N_2O_3^+$:** 185.0921 **found:** 185.0930.

NMR Spectra (^1H, ^{13}C): S101

ethyl 4,6-dimethyl-2-oxo-1,2,3,4-tetrahydropyrimidine-5-carboxylate (1aC)

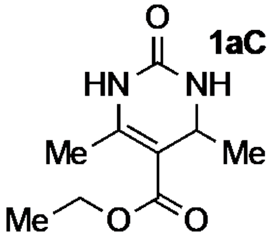

1aC

Prepared according to General Procedure A. Acetaldehyde was distilled directly before use and collected in a pear flask chilled in a dry ice:acetone bath. Product was collected after filtration as a white powder (12 g, 61% yield). Characterization matched previously reported data.[2]

isobutyl 4,6-dimethyl-2-oxo-1,2,3,4-tetrahydropyrimidine-5-carboxylate (1aD)

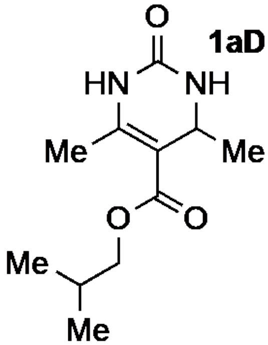

1aD

Prepared according to General Procedure A. Acetaldehyde was distilled directly before use and collected in a pear flask chilled in a dry ice:acetone bath. The product did not precipitate from solution after the reaction and the solvent was removed under vacuum. This gave a blue suspension that was dissolved in DCM and poured into a separatory funnel containing deionized water. After three extractions with DCM, the organic layers were dried over Na_2SO_4, filtered, and concentrated to yield an orange colored residue. The residue was triturated with EtOAc:Hexanes to give a solid that was filtered on a glass frit. Product was collected after filtration as a white powder. (3.6 g, 16% yield). *Minor impurities were present, but the rest of characterization was consistent with the structure and the material was carried forward.* ***Note****: as previously reported for certain aliphatic aldehydes,*[xx9] *switching conditions to 0.60 equiv. of $FeCl_3$•$6H_2O$ and 3 drops of 12 M HCl resulted in higher isolation of the product as an off-white solid (15 g, 66% yield).*

Analytical data for **1aD**:

^1H NMR (500 MHz, Dimethylsulfoxide-*d_6*) δ 8.99 (bs, 1H, ***NH***), 7.19 (bs, 1H, ***NH***), 4.13 (m, 1H, ***CH***), 3.82 (m, 2H, CO_2***CH_2***$CH(CH_3)_2$), 2.16 (s, 3H, –***CH_3***), 1.89 (*apparent* hept, *J* = 6.4 Hz, 1H, CO_2CH_2***CH***$(CH_3)_2$), 1.11 (d, *J* = 6.2 Hz, 3H, –***CH_3***), 0.90 (d, *J* = 6.7 Hz, 6H, CO_2CH_2CH(***CH_3***$)_2$). XX revisit splitting

^{13}C NMR (126 MHz, Dimethylsulfoxide-*d_6*) δ 165.33, 152.47, 147.91, 100.40, 69.18, 46.28, 27.31, 23.42, 19.04, 17.68.

IR (thin film, cm^{-1}): 3242, 2959, 1704, 1651, 1428, 1228, 1098, 1077, 772, 641.

HRMS *m/z* **calculated for $C_{11}H_{19}N_2O_3^+$:** 227.1390 **found:** 227.1398.

NMR Spectra (^1H, ^{13}C): S102

isopropyl 6-methyl-2-oxo-4-(*o*-tolyl)-1,2,3,4-tetrahydropyrimidine-5-carboxylate (1aE)

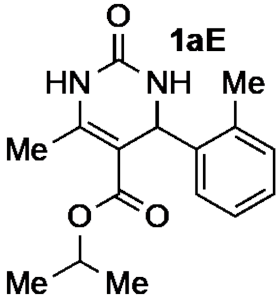

i

Prepared according to General Procedure A. The product did not crash out of solution and the solvent was removed under reduced pressure. This yielded a blue-white residue that was insoluble in DCM. Upon addition of deionized water to the heterogeneous mixture, the contents were swirled vigorously and filtered on a glass frit to yield the product as an off-white powder (16.2 g, 56% yield).

Analytical data for **1aE**:

^1H NMR (500 MHz, Dimethylsulfoxide-*d_6*) δ 9.09 (bs, 1H, ***NH***), 7.58 (bs, 1H, ***NH***), 7.18-7.09 (m, 4H, ***ArH***), 5.38 (d, *J* = 2.8 Hz, 1H, ***CH***), 4.72 (hept, *J* = 6.2 Hz, 1H, CO_2***CH***$(CH_3)_2$), 2.42 (s, 3H, *o*-***ArCH₃***), 2.29 (s, 3H, –***CH₃***), 1.10 (d, *J* = 6.3 Hz, 3H, CO_2CH(***CH₃***)$_2$), 0.77 (d, *J* = 6.2 Hz, 3H, CO_2CH(***CH₃***)$_2$).

^{13}C NMR (126 MHz, Dimethylsulfoxide-*d_6*) δ 164.69, 151.40, 148.22, 143.23, 134.64, 129.99, 127.07, 126.50, 126.43, 99.26, 65.96, 50.54, 21.69, 21.15, 18.65, 17.56.

IR (thin film, cm^{-1}): 3224, 3102, 2975, 1699, 1646, 1467, 1383, 1226, 1078, 760, 726, 669.

HRMS *m/z* **calculated for $C_{16}H_{21}N_2O_3^+$:** 289.1547 **found:** 289.1551.

NMR Spectra (^1H, ^{13}C): S103

methyl 4-(2-fluoro-6-methoxyphenyl)-6-methyl-2-oxo-1,2,3,4-tetrahydropyrimidine-5-carboxylate (1aF)

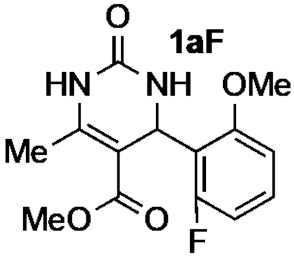

Prepared according to General Procedure A. Product was collected after filtration as a white solid (28 g, 95%).

Analytical data for **1aF**:

^1H NMR (500 MHz, Dimethylsulfoxide-*d_6*) δ 9.15 (bs, 1H, ***NH***), 7.27 (bs, 1H, ***NH***), 7.25-7.20 (m, 1H, ***ArH***), 6.83 (d, *J* = 8.3 Hz, 1H, ***ArH***), 6.73-6.69 (m, 1H, ***ArH***), 5.70 (s, 1H, ***CH***), 3.77 (s, 3H, *o*-***ArOCH₃***), 3.42 (s, 3H, CO_2***CH₃***), 2.18 (s, 3H, –***CH₃***).

^{13}**C NMR** (126 MHz, Dimethylsulfoxide-*d*$_6$) δ 165.81, 161.59, 159.64, 158.82, 151.65, 148.89, 128.96, 128.87, 120.04, 119.93, 107.65, 107.58, 107.56, 107.47, 94.99, 56.01, 50.33, 45.28, 17.84. XX revisit splitting

^{19}**F NMR** (282 MHz, Dimethylsulfoxide-*d*$_6$) δ -117.58.

IR (thin film, cm^{-1}): 3355, 3112, 2989, 1683, 1635, 1610, 1433, 1312, 1225, 1087, 782, 659.

HRMS ***m/z*** **calculated for** $C_{14}H_{16}FN_2O_4^+$**:** 295.1089 **found:** 295.1098.

NMR Spectra (^{1}H, ^{13}C, ^{19}F): S104-S105

methyl 2-hydroxy-4-methyl-6-(2-(trifluoromethyl)phenyl)pyrimidine-5-carboxylate (2a)

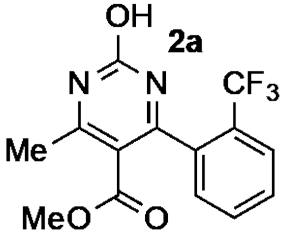

Prepared according to General Procedure B. Collected after filtration as a light red solid (18 g, 89% yield). Characterization matched previously reported data.[2]

ethyl 2-hydroxy-4-methyl-6-(2-(trifluoromethyl)phenyl)pyrimidine-5-carboxylate (2b)

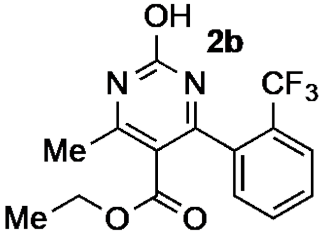

Prepared according to General Procedure B. Product was collected after filtration as a red brown solid (11 g, 88% yield).

Analytical data for **2b**:

^{1}H NMR (500 MHz, Chloroform-*d$_1$*) δ 13.70 (bs, 1H, –***OH***), 7.70 (d, *J* = 7.0 Hz, 1H, ***ArH***), 7.58 (t, *J* = 7.4 Hz, 1H, ***ArH***), 7.54 (t, *J* = 7.6 Hz, 1H, ***ArH***), 7.29 (d, *J* = 7.5 Hz, 1H, ***ArH***), 3.91 (q, *J* = 7.2 Hz, 2H, CO_2***CH_2***CH_3), 2.73 (s, 1H, –***CH_3***), 0.77 (t, *J* = 7.1 Hz, 3H, CO_2CH_2***CH_3***).

^{13}C NMR (126 MHz, Chloroform-*d$_1$*) δ 164.06, 157.44, 131.47, 129.10, 128.45, 126.37, 111.06, 61.24, 13.33.

^{19}F NMR (282 MHz, Chloroform-*d$_1$*) δ -57.92.

IR (thin film, cm^{-1}): 2992, 2801 (br), 1715, 1652, 1593, 1430, 1316, 1267, 1105, 1037, 768, 688, 666.

HRMS *m/z* calculated for $C_{15}H_{14}F_3N_2O_3^+$: 327.0951 **found:** 327.0962.

NMR Spectra (^{1}H, ^{13}C, ^{19}F): S106-S107

isopropyl 2-hydroxy-4-methyl-6-(2-(trifluoromethyl)phenyl)pyrimidine-5-carboxylate (2c)

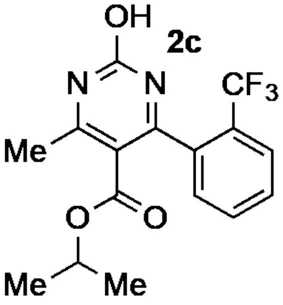

Prepared according to General Procedure B. Product was collected as a red brown solid after filtration (6.6 g, 83% yield).

Analytical data for **2c**:

^{1}H NMR (500 MHz, Chloroform-*d$_1$*) δ 7.70 (d, *J* = 7.6 Hz, 1H, ***ArH***), 7.60-7.51 (m, 2H, ***ArH***), 7.28 (d, *J* = 6.9 Hz, 1H, ***ArH***), 4.83 (hept, *J* = 5.6 Hz, 1H, CO_2***CH***$(CH_3)_2$), 2.71 (s, 3H, –***CH$_3$***), 0.90 (d, *J* = 6.3 Hz, 3H, CO_2CH(***CH$_3$***)$_2$), 0.75 (d, *J* = 6.3 Hz, 3H, CO_2CH(***CH$_3$***)$_2$).

^{13}C NMR (126 MHz, Chloroform-*d$_1$*) δ 163.54, 157.38, 131.43, 129.07, 128.52, 126.40, 124.94, 122.76, 111.45, 69.16, 21.22, 21.04.

^{19}F NMR (282 MHz, Chloroform-*d$_1$*) δ -57.85.

IR (thin film, cm^{-1}): 2984, 1656, 1592, 1425, 1314, 1275, 1167, 1098, 1036, 769, 665.

HRMS *m/z* **calculated for $C_{16}H_{16}F_3N_2O_3^+$:** 341.1108 **found:** 341.1115.

NMR Spectra (^{1}H, ^{13}C, ^{19}F): S108-S109

isobutyl 2-hydroxy-4-methyl-6-(2-(trifluoromethyl)phenyl)pyrimidine-5-carboxylate (2d)

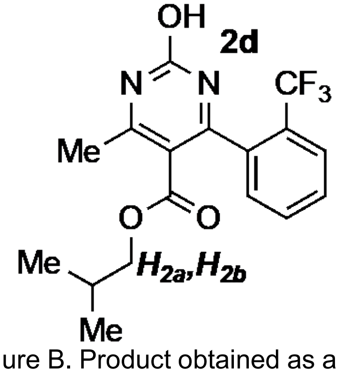

Prepared according to General Procedure B. Product obtained as a salmon colored solid (15 g, 42% yield).

Analytical data for **2d**:

^{1}H NMR (500 MHz, Chloroform-*d$_1$*) δ 13.72 (bs, 1H, –***OH***), 7.71 (d, *J* = 7.9 Hz, 1H, ***ArH***), 7.58 (t, *J* = 7.5 Hz, 1H, ***ArH***), 7.53 (d, *J* = 7.5 Hz, 1H, ***ArH***), 7.30 (d, *J* = 6.9 Hz, 1H, ***ArH***), 3.68 (d, *J* = 7.0 Hz, 1H, CO_2***CH$_{2a}$***$CH_{2b}CH(CH_3)_2$), 3.63 (d, *J* = 7.3 Hz, 1H, CO_2CH_{2a}***CH$_{2b}$***$CH(CH_3)_2$), 2.72 (s, 3H, –***CH$_3$***), 1.41 (*apparent* hept, *J* = 6.7 Hz, 1H, $CO_2CH_{2a}CH_{2b}$***CH***$(CH_3)_2$), 0.67 (s, 6H, $CO_2CH_{2a}CH_{2b}CH$(***CH$_3$***)$_2$). Xx adam check

^{13}C NMR (126 MHz, Chloroform-*d$_1$*) δ 164.39, 157.43, 131.55, 128.54, 126.51, 122.78, 111.23, 71.92, 27.33, 19.05.

^{19}F NMR (282 MHz, Chloroform-*d$_1$*) δ -57.81.

IR (thin film, cm^{-1}): 2969 (br), 1655, 1578, 1425, 1316, 1275, 1167, 1113, 1035, 971, 771, 692, 666.

HRMS *m/z* **calculated for $C_{17}H_{18}F_3N_2O_3^+$:** 355.1264 **found:** 355.1276.

NMR Spectra (^{1}H, ^{13}C, ^{19}F): S110-S111

2-methoxyethyl 2-hydroxy-4-methyl-6-(2-(trifluoromethyl)phenyl)pyrimidine-5-carboxylate (2e)

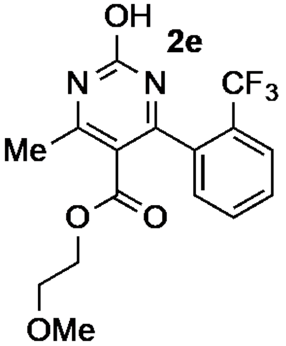

Prepared according to General Procedure B. Collected after filtration as a red brown solid (22 g, 63% yield).

Analytical data for **2e**:

^{1}H NMR (500 MHz, Chloroform-*d$_1$*) δ 7.71 (d, *J* = 7.8 Hz, 1H, ***ArH***), 7.63-7.52 (m, 2H, ***ArH***), 7.32 (d, *J* = 7.6 Hz, 1H, ***ArH***), 4.07-3.95 (m, 2H, CO_2***CH_2***CH_2OCH_3), 3.19 (s, 3H, $CO_2CH_2CH_2$***OCH_3***), 3.15-3.07 (m, 2H, CO_2CH_2***CH_2***OCH_3), 2.73 (s, 1H, –***CH_3***).

^{13}C NMR (126 MHz, Chloroform-*d$_1$*) δ 164.03, 157.37, 131.50, 129.08, 128.60, 126.34, 110.81, 69.58, 64.13, 58.81.

^{19}F NMR (282 MHz, Chloroform-*d$_1$*) δ -57.96.

IR (thin film, cm^{-1}): 2810, 1719, 1654, 1594, 1428, 1318, 1278, 1114, 1030, 873, 766, 689, 666.

HRMS *m/z* **calculated for $C_{16}H_{16}F_3N_2O_4^+$:** 357.1057 **found:** 357.1068.

NMR Spectra (^{1}H, ^{13}C, ^{19}F): S112-S113

methyl 2-hydroxy-4-methyl-6-(*o*-tolyl)pyrimidine-5-carboxylate (2f)

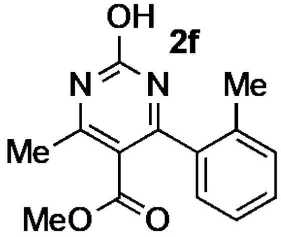

Prepared according to General Procedure B. Collected after filtration as a brown solid (14 g, 99% yield). Characterization matched previously reported data.[2]

methyl 4-(2,6-dimethylphenyl)-2-hydroxy-6-methylpyrimidine-5-carboxylate (2g)

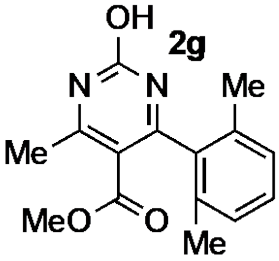

Prepared according to General Procedure B. Product collected after filtration as a light orange solid (6.7 g, 84% yield).

Analytical data for **2g**:

^{1}H NMR (500 MHz, Chloroform-d_1) δ 7.17 (t, J = 7.7 Hz, 1H, *p-**ArH***), 7.03 (d, J = 7.6 Hz, 2H, *m-**ArH***), 3.45 (s, 3H, CO_2***CH₃***), 2.61 (s, 3H, –***CH₃***), 2.12 (s, 6H, o-***ArCH₃***).

^{13}C NMR (126 MHz, Chloroform-d_1) δ 165.50, 158.06, 127.52, 112.41, 52.29, 31.73, 22.80, 19.73, 14.28.

IR (thin film, cm^{-1}): 2951 (br), 1721, 1648, 1594, 1425, 1274, 1106, 950, 775, 669.

HRMS *m/z* **calculated for $C_{15}H_{17}N_2O_3^+$:** 273.1234 **found:** 273.1242.

NMR Spectra (^{1}H, ^{13}C): S114

methyl 2-hydroxy-4-mesityl-6-methylpyrimidine-5-carboxylate (2h)

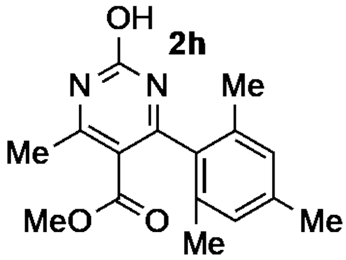

Prepared according to General Procedure B. The product was collected as a light orange solid after filtration (10 g, 57% yield). Characterization matched previously reported data.[2]

methyl 4-(2,6-dimethyl-4-propoxyphenyl)-2-hydroxy-6-methylpyrimidine-5-carboxylate (2i)

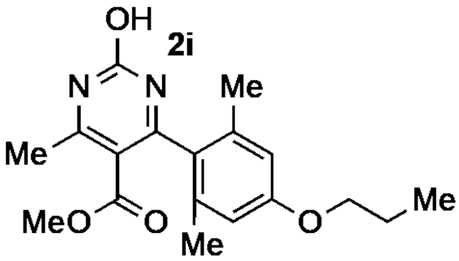

Prepared according to General Procedure B. Collected after filtration as an orange solid (6.5 g, 94% yield).

Analytical data for **2i**:

^{1}**H NMR** (500 MHz, Chloroform-d_1) δ 6.57 (s, 2H, *m-**ArH***), 3.89 (t, J = 6.6 Hz, 2H, *p*-ArO***CH₂***CH₂CH₃), 3.50 (s, 3H, CO_2***CH₃***), 2.59 (s, 3H, –***CH₃***), 2.10 (s, 6H, *o-**ArCH₃***), 1.79 (h, J = 7.1 Hz, 2H, *p*-ArOCH₂**CH₂**CH₃), 1.03 (t, J = 7.4 Hz, 3H, *p*-ArOCH₂CH₂**CH₃**).

^{13}**C NMR** (126 MHz, Chloroform-d_1) δ 165.84, 113.55, 113.04, 69.35, 52.38, 22.80, 20.03, 10.70.

IR (thin film, cm^{-1}): 2877 (br), 1721, 1651, 1601, 1434, 1319, 1272, 1208, 1164, 1127, 1103, 1057, 964, 677.

HRMS *m/z* **calculated for $C_{18}H_{23}N_2O_4^+$:** 331.1652 **found:** 331.1661.

NMR Spectra (^{1}H, ^{13}C): S115

methyl 4-(3,5-di-*tert*-butylphenyl)-2-hydroxy-6-methylpyrimidine-5-carboxylate (2j)

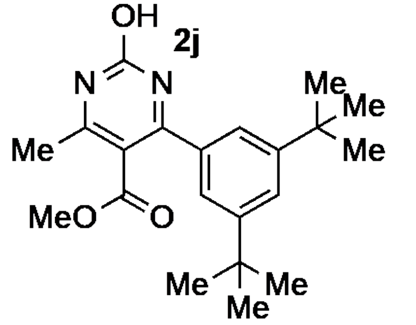

2j

Prepared according to General Procedure B. Product collected after filtration as an off-white solid (11 g, 76% yield)

Analytical data for **2j**:

^{1}**H NMR** (500 MHz, Chloroform-d_1) δ 7.54 (s, 1H, *p-**ArH***), 7.42 (d, J = 1.8 Hz, 2H, *o-**ArH***), 3.59 (s, 2H, CO_2***CH₃***), 2.59 (s, 2H, –***CH₃***), 1.34 (s, 9H, *m-**ArC(CH₃)₃***).

^{13}**C NMR** (126 MHz, Chloroform-d_1) δ 167.17, 158.49, 151.22, 125.39, 122.49, 111.70, 52.49, 35.14, 31.56.

IR (thin film, cm^{-1}): 2953 (br), 2868, 1722, 1658, 1593,1447, 1254, 1203, 1102, 944, 886, 799, 708, 676.

HRMS *m/z* **calculated for $C_{21}H_{29}N_2O_3^+$:** 357.2173 **found:** 357.2181.

NMR Spectra (^{1}H, ^{13}C): S116

methyl 4-(3,5-dimethylphenyl)-2-hydroxy-6-methylpyrimidine-5-carboxylate (2k)

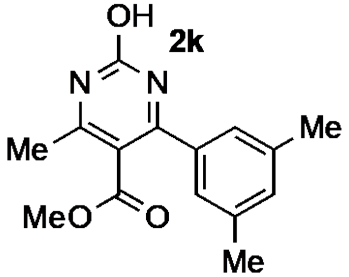

2k

Prepared according to General Procedure B. Product collected after filtration as a light brown solid (8.5 g, 86% yield).
Analytical data for **2k**:

^{1}**H NMR** (500 MHz, Chloroform-*d₁*) δ 7.23 (s, 2H, *m*-***ArH***), 7.11 (s, 1H, *p*-***ArH***), 3.60 (s, 1H, CO_2***CH₃***), 2.59 (s, 1H, –***CH₃***), 2.34 (s, 6H, *m*-***ArCH₃***).

^{13}**C NMR** (126 MHz, Chloroform-*d₁*) δ 167.04, 158.48, 138.20, 132.91, 125.99, 111.29, 52.41, 21.35.

IR (thin film, cm^{-1}): 2721 (br), 1736, 1636, 1595, 1432, 1303, 1246, 1102, 940, 865, 690, 671.

HRMS *m/z* **calculated for $C_{15}H_{17}N_2O_3^+$:** 273.1234 **found:** 273.1243.

NMR Spectra (^{1}H, ^{13}C): S117

methyl 4-(3,5-bis(trifluoromethyl)phenyl)-2-hydroxy-6-methylpyrimidine-5-carboxylate (2l)

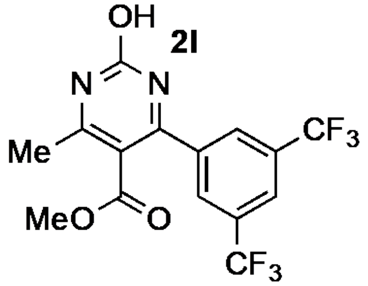

Prepared according to General Procedure B. Collected after filtration as a maroon solid (13 g, 73% yield). *Yield and characterization collected with minor impurities.*

Analytical data for **2l**:

^{1}**H NMR** (500 MHz, Chloroform-*d₁*) δ 8.05 (s, 2H, *m*-***ArH***), 8.00 (s, 1H, *p*-***ArH***), 3.63 (s, 1H, CO_2***CH₃***), 2.68 (s, 1H, –***CH₃***).

^{13}**C NMR** (126 MHz, Chloroform-*d₁*) δ 165.49, 139.69, 132.20, 131.93, 128.58, 128.37, 124.45, 124.14, 121.97, 110.96, 52.69, 19.10.

IR (thin film, cm^{-1}): 2958 (br), 1661, 1596, 1351, 1275, 1171, 1125, 902, 681.

^{19}**F NMR** (282 MHz, Chloroform-*d₁*) δ -62.90.

HRMS *m/z* **calculated for $C_{15}H_{11}F_6N_2O_3^+$:** 381.0668 **found:** 381.0671.

NMR Spectra (^{1}H, ^{13}C, ^{19}F): S118-S119

2-methoxyethyl 4-(2,6-dimethyl-4-propoxyphenyl)-2-hydroxy-6-methylpyrimidine-5-carboxylate (2m)

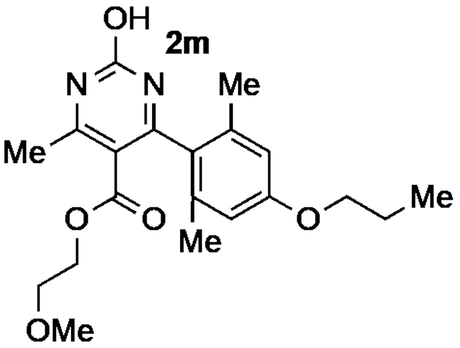

Prepared according to General Procedure B. Product collected after filtration as a pink solid (8.4 g, 73% yield). *Yield and characterization collected with minor impurities.*

Analytical data for **2m**:

^{1}H NMR (500 MHz, Chloroform-d_1) δ 6.57 (s, 2H, *m-**ArH***), 4.09-4.03 (m, 2H, CO_2***CH_2***CH_2OCH_3), 3.89 (t, *J* = 6.4 Hz, 2H, *p*-ArO***CH_2***CH_2CH_3), 3.24 (s, 3H, $CO_2CH_2CH_2$***OCH_3***), 3.19-3.14 (m, 2H, CO_2CH_2***CH_2***OCH_3), 2.61 (s, 3H, –***CH_3***), 2.11 (s, 6H, *o-**ArCH$_3$***), 1.83-1.74 (m, 2H, *p*-ArOCH_2***CH_2***CH_3), 1.03 (t, *J* = 7.0 Hz, 3H, *p*-ArOCH_2CH_2***CH_3***).

^{13}C NMR (126 MHz, Chloroform-d_1) δ 165.91, 165.26, 158.05, 157.96, 152.60, 145.23, 130.79, 114.06, 113.55, 112.82, 99.61, 70.38, 69.91, 69.41, 64.12, 62.84, 58.96, 58.80, 52.00, 24.15, 22.72, 22.19, 20.47, 20.00, 18.54, 10.66.

IR (thin film, cm^{-1}): 2925 (br), 1667, 1651, 1601, 1430, 1318, 1273, 1229, 1162, 1102, 1058, 673.

HRMS *m/z* **calculated for $C_{20}H_{27}N_2O_5^+$:** 375.1914 **found:** 375.1925.

NMR Spectra (^{1}H, ^{13}C): S120

isopropyl 2-hydroxy-4-mesityl-6-methylpyrimidine-5-carboxylate (2n)

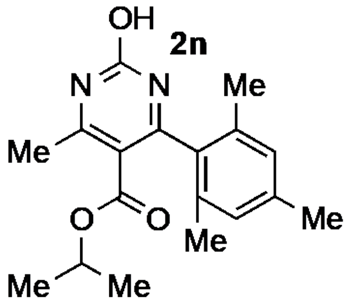

Prepared according to General Procedure B. Product was collected as a light orange solid after filtration (11 g, 71%).

Analytical data for **2n**:

^{1}H NMR (500 MHz, Chloroform-d_1) δ 6.85 (s, 2H, *m-**ArH***), 4.84 (hept, *J* = 6.2 Hz, 1H, CO_2***CH***$(CH_3)_3$), 2.62 (s, 3H, –***CH_3***), 2.27 (s, 3H, *p-**ArCH$_3$***), 2.09 (s, 6H, *o-**ArH***), 0.86 (d, *J* = 6.2 Hz, 6H, CO_2CH(***CH_3***)$_2$).

^{13}C NMR (126 MHz, Chloroform-d_1) δ 164.67, 158.03, 134.90, 128.31, 113.15, 69.10, 21.26, 21.03, 19.64.

IR (thin film, cm^{-1}): 2981, 2920 (br), 1716, 1652, 1593, 1429, 1274, 1104, 913, 803, 668.

HRMS *m/z* **calculated for $C_{18}H_{23}N_2O_3^+$:** 315.1703 **found:** 315.1716.

NMR Spectra (^{1}H, ^{13}C): S121

isopropyl 4-(3,5-di-*tert*-butylphenyl)-2-hydroxy-6-methylpyrimidine-5-carboxylate (2o)

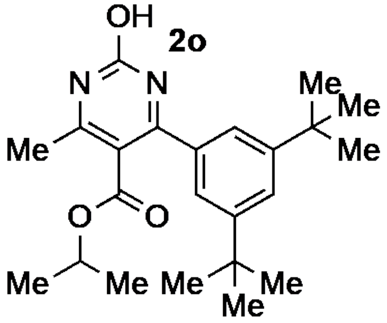

Prepared according to General Procedure B. The product was collected as an off-white solid after filtration (13 g, 66% yield).

Analytical data for **2o**:

^{1}H NMR (500 MHz, Chloroform-d_1) δ 13.76 (bs, 1H, –***OH***), 7.52 (s, 1H, *p*-***ArH***), 7.36 (s, 1H, *o*-***ArH***), 4.76 (hept, *J* = 6.3 Hz, 1H, CO_2***CH***$(CH_3)_2$), 2.59 (s, 3H, –***CH₃***), 1.32 (s, 18H, *m*-***ArC(CH₃)₃***), 0.88 (d, *J* = 6.8 Hz, 6H, CO_2CH(***CH₃***$)_2$).

^{13}C NMR (126 MHz, Chloroform-d_1) δ 166.01, 122.16, 112.46, 69.60, 35.11, 31.51, 21.34.

IR (thin film, cm^{-1}): 2962, 2733 (br), 1714, 1652, 1594, 1258, 1104, 918, 706, 684.

HRMS *m/z* **calculated for $C_{23}H_{33}N_2O_3^+$:** 385.2486 **found:** 385.2494.

NMR Spectra (^{1}H, ^{13}C): S122

2-methoxyethyl 4-(2,6-dimethylphenyl)-2-hydroxy-6-methylpyrimidine-5-carboxylate (2p)

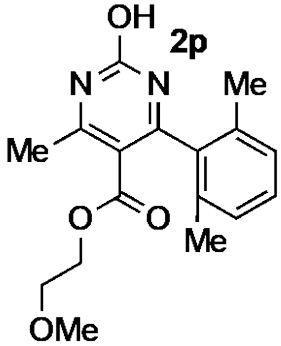

Prepared according to General Procedure B. Trituration in EtOAc:Hexanes failed to precipitate the product, but small quantity of light brown solid was present and the mixture subsequently filtered on a glass frit. The filtrate was dissolved in DCM and concentrated to yield the product as a low melting solid/viscous red oil (4.7 g, 53% yield).

Analytical data for **2p**:

¹H NMR (500 MHz, Chloroform-*d₁*) δ 7.18 (t, *J* = 7.6 Hz, 1H, *p-**ArH***), 7.02 (d, *J* = 7.7 Hz, 2H, *m-**ArH***), 4.04-3.98 (m, 2H, CO_2***CH_2***CH_2OCH_3), 3.22 (s, 3H, $CO_2CH_2CH_2$***OCH_3***), 3.09-3.06 (m, 2H, CO_2CH_2***CH_2***OCH_3), 2.64 (s, 3H, –***CH_3***), 2.13 (s, 6H, *o-**$ArCH_3$***).

¹³C NMR (126 MHz, Chloroform-*d₁*) δ 164.94, 158.09, 134.82, 129.16, 127.52, 112.28, 69.73, 64.10, 58.77, 19.72.

IR (thin film, cm^{-1}): 2949 (br), 1652, 1592, 1418, 1273, 1103, 1027, 775, 665.

HRMS *m/z* **calculated for $C_{17}H_{21}N_2O_4^+$:** 317.1496 **found:** 317.1502.

NMR Spectra (¹H, ¹³C): S123

methyl 2-hydroxy-4,6-dimethylpyrimidine-5-carboxylate (2aB)

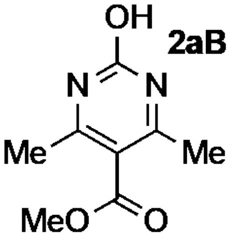

Prepared according to General Procedure C. Product collected after filtration as a pale-yellow solid (3.6 g, 68% yield).

Analytical data for **2aB**:

¹H NMR (500 MHz, Chloroform-*d₁*) δ 13.60 (bs, 1H, –***OH***), 3.89 (s, 1H, CO_2***CH_3***), 2.57 (s, 2H, –***CH_3***).

¹³C NMR (126 MHz, Chloroform-*d₁*) δ 165.91, 158.29, 111.13, 52.42.

IR (thin film, cm^{-1}): 2812 (br), 1652, 1592, 1408, 1266, 1117, 938, 797, 677.

HRMS *m/z* **calculated for $C_8H_{11}N_2O_3^+$:** 183.0764 **found:** 183.0772.

NMR Spectra (¹H, ¹³C): S124

ethyl 2-hydroxy-4,6-dimethylpyrimidine-5-carboxylate (2aC)

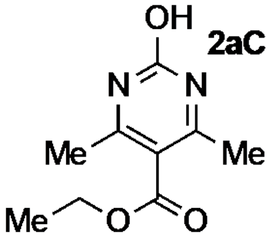

Prepared according to General Procedure B. Product was collected after filtration as a pale yellow solid (8.7 g, 76% yield). Characterization matched previously reported data.[2]

isobutyl 2-hydroxy-4,6-dimethylpyrimidine-5-carboxylate (2aD)

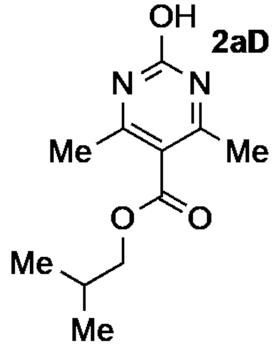

Prepared according to General Procedure C. Product collected as a pale yellow solid (8.1 g, 63% yield).

Analytical data for **2aD**:

^{1}H NMR (500 MHz, Chloroform-*d_1*) δ 13.55 (bs, 1H, –***OH***), 4.09 (d, *J* = 6.6 Hz, 2H, CO_2***CH_2***$CH(CH_3)_2$), 2.57 (s, 3H, –***CH_3***), 2.04 (*apparent* hept, *J* = 6.7 Hz, 1H, CO_2CH_2***CH***$(CH_3)_2$), 1.00 (d, *J* = 6.7 Hz, 1H, $CO_2CH_2CH($***CH_3***$)_2$). Xx adam check

^{13}C NMR (126 MHz, Chloroform-*d_1*) δ 165.66, 158.27, 72.09, 27.86, 19.47.

IR (thin film, cm^{-1}): 2961, 2878 (br), 1698, 1652, 1588, 1411, 1326, 1268, 1120, 942, 800, 680, 661, 575.

HRMS *m/z* **calculated for $C_{11}H_{17}N_2O_3^+$:** 225.1234 **found:** 225.1238.

NMR Spectra (^{1}H, ^{13}C): S125

isopropyl 2-hydroxy-4-methyl-6-(*o*-tolyl)pyrimidine-5-carboxylate (2aE)

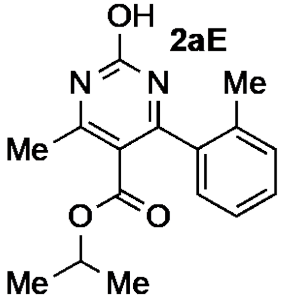

Prepared according to General Procedure B. Trituration in EtOAc:Hexanes or DCM:Hexanes failed. The crude viscous oil was placed under high vacuum overnight to yield a maroon solid. Product was collected after filtration as a maroon powder (12 g, 79% yield).

Analytical data for **2aE**:

^{1}H NMR (500 MHz, Chloroform-*d_1*) δ 7.31 (t, *J* = 7.6 Hz, 1H, ***ArH***), 7.25-7.16 (m, 2H, ***ArH***), 7.14-7.09 (m, 1H, ***ArH***), 4.81 (hept, *J* = 6.3 Hz, 1H, CO_2***CH***$(CH_3)_2$), 2.62 (s, 3H, –***CH_3***), 2.28 (s, 3H, *o*-***$ArCH_3$***), 0.84 (d, *J* = 6.0 Hz, 6H, $CO_2CH($***CH_3***$)_2$).

^{13}C NMR (126 MHz, Chloroform-*d_1*) δ 164.73, 157.85, 135.34, 130.48, 129.71, 127.33, 125.67, 112.79, 69.29, 21.09, 19.57.

IR (thin film, cm^{-1}): 2980, 2932 (br), 1651, 1593, 1417, 1271, 1100, 913, 732, 672.

HRMS *m/z* **calculated for $C_{16}H_{19}N_2O_3^+$:** 287.1390 **found:** 287.1404.

NMR Spectra (^{1}H, ^{13}C): S126

methyl 4-(2-fluoro-6-methoxyphenyl)-2-hydroxy-6-methylpyrimidine-5-carboxylate (2aF)

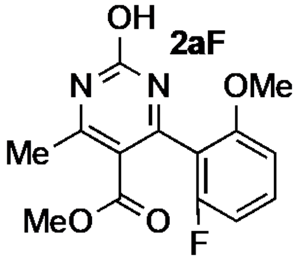

Prepared according to General Procedure B. Product was collected as a light brown solid after filtration (quantitative yield).

Analytical data for **2aF**:

^{1}H NMR (500 MHz, Chloroform-*d₁*) δ 7.33 (q, *J* = 7.9 Hz, 1H, ***ArH***), 6.79-6.70 (m, 2H, ***ArH***), 3.77 (s, 3H, *o*-***ArOCH₃***), 3.55 (s, 3H, CO_2***CH_3***), 2.67 (s, 3H, –***CH_3***).

^{13}C NMR (126 MHz, Chloroform-*d₁*) δ 165.16, 160.78, 158.80, 158.06, 157.41, 157.36, 131.69, 112.41, 108.44, 108.26, 106.58, 56.25, 52.00.

^{19}F NMR (282 MHz, Chloroform-*d₁*) δ -114.63.

IR (thin film, cm^{-1}): 2730 (br), 1724, 1652, 1592, 1476, 1425, 1270, 1208, 1131, 1080, 962, 935, 788, 749, 686, 647.

HRMS *m/z* **calculated for $C_{14}H_{14}FN_2O_4^+$:** 293.0932 **found:** 293.0940.

NMR Spectra (^{1}H, ^{13}C, ^{19}F): S127-S128

methyl 2-chloro-4-methyl-6-(2-(trifluoromethyl)phenyl)pyrimidine-5-carboxylate (3a)

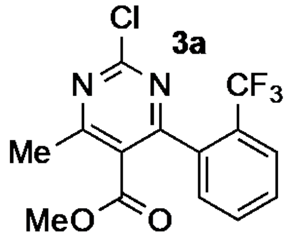

Prepared according to General Procedure D. Isolated via column chromatrography (silica gel, 5 to 10% EtOAc:Hexanes) as an off-white crystalline solid (11 g, 56% yield). Characterization matched previously reported data.[2]

ethyl 2-chloro-4-methyl-6-(2-(trifluoromethyl)phenyl)pyrimidine-5-carboxylate (3b)

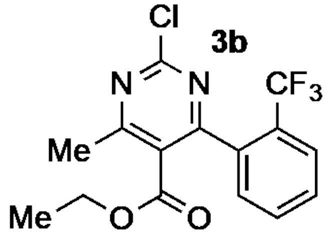

Prepared according to General Procedure D. Isolated via column chromatrography (silica gel, 10% EtOAc:Hexanes) as an off-white crystalline solid (7.2 g, 71% yield).

Analytical data for **3b**:

^{1}H NMR (500 MHz, Chloroform-*d_1*) δ 7.79-7.74 (m, 1H, ***ArH***), 7.63-7.56 (m, 2H, ***ArH***), 7.32-7.26 (m, 1H, ***ArH***), 4.03 (q, *J* = 7.1 Hz, 2H, CO_2***CH_2***CH_3), 2.72 (s, 3H, –***CH_3***), 0.87 (t, *J* = 7.1 Hz, 3H, CO_2CH_2***CH_3***).

^{13}C NMR (126 MHz, Chloroform-*d_1*) δ 170.01, 167.02, 165.27, 160.31, 135.47, 131.54, 129.75, 129.70, 128.56, 128.31, 126.86, 126.82, 124.87, 124.79, 122.62, 62.00, 23.48, 13.48.

^{19}F NMR (282 MHz, Chloroform-*d_1*) δ –57.71

IR (thin film, cm^{-1}): 1720, 1525, 1315, 1238, 1166,1124, 1107, 1057, 776.

HRMS *m/z* **calculated for $C_{15}H_{13}ClF_3N_2O_2^+$:** 345.0612 and 347.0583 **found:** 345.0623 and 347.0598.

NMR Spectra (^{1}H, ^{13}C, ^{19}F): S129-S130

isopropyl 2-chloro-4-methyl-6-(2-(trifluoromethyl)phenyl)pyrimidine-5-carboxylate (3c)

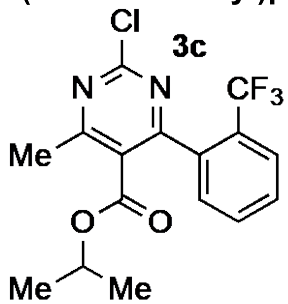

Prepared according to General Procedure D. Isolated via column chromatrography (silica gel, 10% to 20% EtOAc:Hexanes) as a white crystalline solid (4.8 g, 51%).

Analytical data for **3c**:

^{1}H NMR (500 MHz, Chloroform-*d$_1$*) δ 7.79-7.74 (m, 1H, ***ArH***), 7.62-7.56 (m, 2H, ***ArH***), 7.31-7.27 (m, 1H, ***ArH***), 4.92 (hept, *J* = 6.3 Hz, 1H, CO_2***CH***$(CH_3)_2$), 2.71 (s, 3H, –***CH_3***), 0.91 (s, 6H, CO_2CH(***CH_3***)$_2$).

^{13}C NMR (126 MHz, Chloroform-*d$_1$*) δ 169.80, 166.76, 164.77, 160.11, 135.43, 131.51, 129.83, 129.74, 128.93, 128.68, 128.43, 128.17, 126.90, 126.86, 126.82, 126.79, 125.23, 124.77, 122.59, 70.09, 23.36, 21.14.

^{19}F NMR (282 MHz, Chloroform-*d$_1$*) δ –57.68

IR (thin film, cm^{-1}): 2988, 1717, 1531, 1312, 1235, 1163, 1125, 1110, 1087, 1054, 1035, 942, 912, 782.

HRMS ***m/z*** **calculated for $C_{16}H_{15}ClF_3N_2O_2^+$:** 359.0769 and 361.0740 **found:** 359.0784 and 361.0757.

NMR Spectra (^{1}H, ^{13}C, ^{19}F): S131-S132

isobutyl 2-chloro-4-methyl-6-(2-(trifluoromethyl)phenyl)pyrimidine-5-carboxylate (3d)

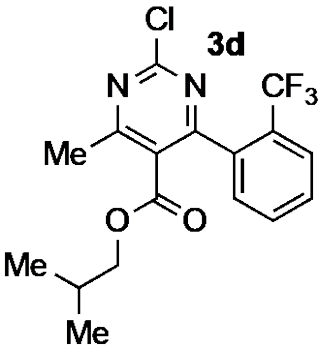

3d

Prepared according to General Procedure D. Isolated via column chromatrography (silica gel, 10% EtOAc:Hexanes) as a yellow-orange solid (9.2 g, 76% yield).

Analytical data for **3d**:

^{1}H NMR (500 MHz, Chloroform-*d$_1$*) δ 7.80-7.74 (m, 1H, ***ArH***), 7.62-7.55 (m, 2H, ***ArH***), 7.32-7.28 (m, 1H, ***ArH***), 3.76 (d, *J* = 6.5 Hz, 2H, CO_2***CH_2***$CH(CH_3)_2$), 2.71 (s, 3H, –***CH_3***), 1.56 (*apparent* hept, *J* = 6.7 Hz, 1H, CO_2CH_2***CH***$(CH_3)_2$), 0.69 (d, *J* = 6.8 Hz, 1H, CO_2CH_2CH(***CH_3***)$_2$). Xx adam check

^{13}C NMR (126 MHz, Chloroform-*d$_1$*) δ 169.85, 166.69, 165.65, 160.24, 135.43, 131.61, 129.84, 129.75, 128.90, 128.65, 128.40, 128.14, 127.10, 127.06, 127.02, 126.99, 125.05, 124.78, 122.60, 120.42, 72.47, 27.44, 23.52, 18.91.

^{19}F NMR (282 MHz, Chloroform-*d$_1$*) δ –57.59.

IR (thin film, cm^{-1}): 2960, 2877, 1727, 1530, 1314, 1217, 1166, 1126, 1112, 1081, 1058, 1035, 934, 772, 668.

HRMS ***m/z*** **calculated for $C_{17}H_{17}ClF_3N_2O_2^+$:** 373.0925 and 375.0896 **found:** 373.0938 and 375.0909.

NMR Spectra (^{1}H, ^{13}C, ^{19}F): S133-S134

2-methoxyethyl 2-chloro-4-methyl-6-(2-(trifluoromethyl)phenyl)pyrimidine-5-carboxylate (3e)

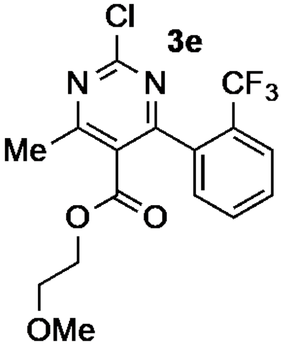

Prepared according to General Procedure D. Isolated via column chromatography (silica gel, 10 to 25% EtOAc:Hexanes) as a brown solid (7.8 g, 37% yield).

Analytical data for **3e**:

^{1}H NMR (500 MHz, Chloroform-*d$_1$*) δ 7.80-7.73 (m, 1H, ***ArH***), 7.63-7.57 (m, 2H, ***ArH***), 7.34-7.28 (m, 1H, ***ArH***), 4.15-4.10 (m, 2H, CO_2***CH_2***CH_2OCH_3), 3.25-3.22 (m, 2H, CO_2CH_2***CH_2***OCH_3), 3.21 (s, 3H, $CO_2CH_2CH_2$***OCH_3***), 2.72 (s, 3H, –***CH_3***).

^{13}C NMR (126 MHz, Chloroform-*d$_1$*) δ 170.13, 167.04, 165.29, 160.41, 135.40, 131.55, 129.72, 128.78, 128.53, 128.28, 128.03, 126.88, 126.85, 126.81, 126.77, 124.79, 124.64, 122.61, 69.66, 64.74, 58.85.

^{19}F NMR (282 MHz, Chloroform-*d$_1$*) δ –57.72.

IR (thin film, cm^{-1}): 2926, 1724, 1530, 1317, 1239, 1121, 1096, 1057, 1020, 943, 775, 669.

HRMS *m/z* **calculated for $C_{16}H_{15}ClF_3N_2O_3^+$:** 375.0718 and 377.0689 **found:** 375.0731 and 377.0703.

NMR Spectra (^{1}H, ^{13}C, ^{19}F): S135-S136

methyl 2-chloro-4-methyl-6-(*o*-tolyl)pyrimidine-5-carboxylate (3f)

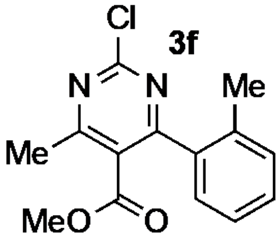

Prepared according to General Procedure D. Isolated via column chromatography (silica gel, 10% EtOAc:Hexanes). as a light orange solid (3.6 g, 28% yield). *$POCl_3$ was not distilled prior to use in this instance*. Characterization matched previously reported data.[2]

methyl 2-chloro-4-(2,6-dimethylphenyl)-6-methylpyrimidine-5-carboxylate (3g)

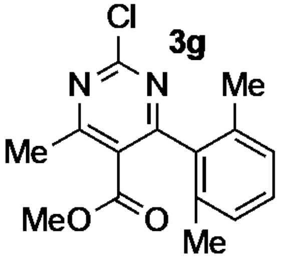

Prepared according to General Procedure D. Product was isolated via column chromatography (silica gel, 10% EtOAc:Hexanes) as an off-white solid. (3.2 g, 56% yield).

Analytical data for **3g**:

^{1}H NMR (500 MHz, Chloroform-*d$_1$*) δ 7.19 (t, *J* = 7.6 Hz, 1H, *p-**ArH***), 7.05 (d, *J* = 7.6 Hz, 2H, *m*-**ArH**), 3.55 (s, 3H, CO_2***CH$_3$***), 2.64 (s, 3H, –***CH$_3$***), 2.03 (s, 6H, *o-**ArCH$_3$***).

^{13}C NMR (126 MHz, Chloroform-*d$_1$*) δ 168.93, 168.81, 166.46, 161.30, 135.75, 135.21, 129.27, 127.53, 126.41, 52.73, 22.97, 19.85.

IR (thin film, cm^{-1}): 2957, 1724, 1545, 1524, 1226, 1084, 928, 859, 774.

HRMS *m/z* **calculated for $C_{15}H_{16}ClN_2O_2^+$:** 291.0895 and 293.0866 **found**: 291.0905 and 293.0877.

NMR Spectra (^{1}H, ^{13}C): S137

methyl 2-chloro-4-mesityl-6-methylpyrimidine-5-carboxylate (3h)

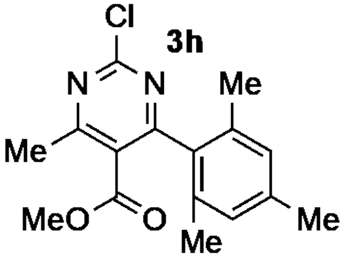

Prepared according to General Procedure D. Product was isolated via column chromatography (silica gel, 5% to 10% EtOAc:Hexanes) as an off-white solid. (3.5 g, 81% yield). Characterization matched previously reported data.[2]

methyl 2-chloro-4-(2,6-dimethyl-4-propoxyphenyl)-6-methylpyrimidine-5-carboxylate (3i)

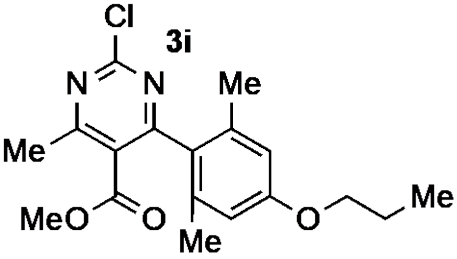

Prepared according to General Procedure D. Product was purified via column chromatography (silica gel, 10 EtOAc:hexanes) as an orange-red solid (4.8 g, 76% yield).

Analytical data for **3i**:

^{1}**H NMR** (500 MHz, Chloroform-*d*$_1$) δ 6.59 (s, 2H, *m*-***ArH***), 3.90 (t, *J* = 6.6 Hz, 2H, *p*-ArO$\boldsymbol{CH_2}$$CH_2CH_3$), 3.59 (s, 3H, $CO_2\boldsymbol{CH_3}$), 2.62 (s, 3H, –$\boldsymbol{CH_3}$), 1.99 (s, 6H, *o*-$\boldsymbol{ArCH_3}$), 1.79 (h, *J* = 7.1 Hz, 2H, *p*-$ArOCH_2\boldsymbol{CH_2}CH_3$), 1.03 (t, *J* = 7.4 Hz, 3H, *p*-$ArOCH_2CH_2\boldsymbol{CH_3}$).

^{13}**C NMR** (126 MHz, Chloroform-*d*$_1$) δ 168.99, 168.47, 166.66, 161.14, 159.53, 136.89, 128.34, 127.05, 113.56, 69.50, 52.79, 22.87, 22.72, 20.13, 10.63.

IR (thin film, cm^{-1}): 2963, 2877, 1732, 1527, 1219, 1170, 1118, 1084, 927, 845, 649.

HRMS ***m/z*** **calculated for** $\mathbf{C_{18}H_{22}ClN_2O_3^+}$**:** 349.1313 and 351.1284 **found** 349.1320 and 351.1295.

NMR Spectra (^{1}H, ^{13}C): S138

methyl 2-chloro-4-(3,5-di-*tert*-butylphenyl)-6-methylpyrimidine-5-carboxylate (3j)

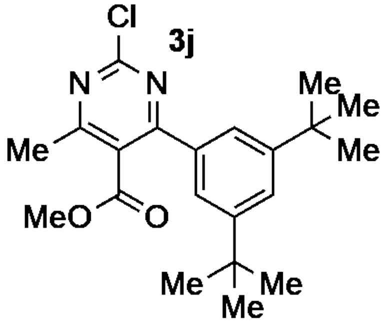

Prepared according to General Procedure D. Product was isolated via column chromatography (silica gel, 5% to 10% EtOAc:hexanes) as an off-white solid (7.7 g, 73% yield).

Analytical data for **3j**:

^{1}**H NMR** (500 MHz, Chloroform-*d*$_1$) δ 7.55 (m, 1H, *p*-***ArH***), 7.47 (m, 2H, *o*-***ArH***), 3.74 (s, 3H, $CO_2\boldsymbol{CH_3}$), 2.60 (s, 3H, –$\boldsymbol{CH_3}$), 1.34 (s, 18H, *m*-$\boldsymbol{ArC(CH_3)_3}$).

^{13}**C NMR** (126 MHz, Chloroform-*d*$_1$) δ 168.31, 168.10, 167.50, 160.73, 151.54, 135.68, 125.25, 124.50, 122.85, 53.00, 35.16, 31.55, 22.66.

IR (thin film, cm^{-1}): 2962, 2868, 1729, 1532, 1231, 1082, 910, 885, 712, 704.

HRMS ***m/z*** **calculated for** $\mathbf{C_{21}H_{28}ClN_2O_2^+}$**:** 375.1834 and 377.1805 **found**: 375.1841and 377.1817.

NMR Spectra (^{1}H, ^{13}C): S139

methyl 2-chloro-4-(3,5-dimethylphenyl)-6-methylpyrimidine-5-carboxylate (3k)

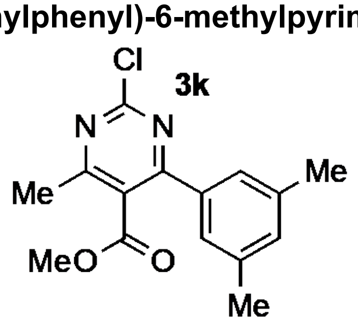

Prepared according to General Procedure D. Product was isolated by column chromatography (silica gel, 0% to 10% EtOAc:Hexanes) as a pale yellow solid (4.4 g, 55% yield).

Analytical data for **3k**:

^{1}H NMR (500 MHz, Chloroform-d_1) δ 7.26 (s, 2H, *o-**ArH*** under $CDCl_3$), 7.13 (s, 1H, *p-**ArH***), 3.74 (s, 3H, CO_2***CH₃***), 2.60 (s, 3H, –***CH₃***), 2.36 (s, 6H, *m-**ArCH₃***)

^{13}C NMR (126 MHz, Chloroform-d_1) δ 168.58, 167.99, 166.64, 160.75, 138.62, 136.21, 132.82, 126.22, 124.25, 52.95, 22.70, 21.42.

IR (thin film, cm^{-1}): 2953, 2915, 1737, 1526, 1438, 1219, 1179, 1132, 1080, 911, 860, 816, 726, 668.

HRMS *m/z* **calculated for $C_{15}H_{16}ClN_2O_2^+$:** 291.0895 and 293.0866 **found**: 291.0906 and 293.0880.

NMR Spectra (^{1}H, ^{13}C): S140

methyl 4-(3,5-bis(trifluoromethyl)phenyl)-2-chloro-6-methylpyrimidine-5-carboxylate (3l)

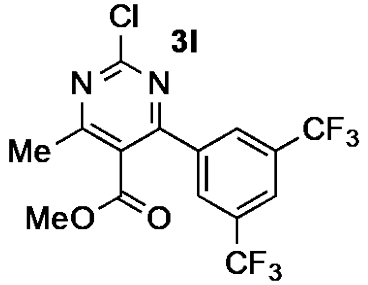

3l

Prepared according to General Procedure D. Product was isolated by column chromatography (silica gel, 5% EtOAc:Hexanes) as a rust colored solid (3.7 g, 28% yield).

Analytical data for **3l**:

^{1}H NMR (500 MHz, Chloroform-d_1) δ 8.13 (s, 2H, *o-**ArH***), 8.02 (s, 1H, *p-**ArH***), 3.80 (s, 3H, *CO_2**CH₃***), 2.67 (s, 3H, –***CH₃***).

^{13}C NMR (126 MHz, Chloroform-d_1) δ 169.96, 166.90, 162.95, 161.35, 138.22, 132.94, 132.67, 132.40, 132.13, 128.88, 126.27, 124.55, 124.52, 124.49, 124.46, 124.10, 121.93, 119.76, 53.29, 22.99.

^{19}F NMR (282 MHz, Chloroform-d_1) δ –62.93

IR (thin film, cm^{-1}): 2956, 1725, 1533, 1349, 1276, 1226, 1170, 1121, 897, 726, 682.

HRMS *m/z* **calculated for $C_{15}H_{10}ClF_6N_2O_2^+$:** 399.0330 and 401.0300 **found**: 399.0345 and 401.0325.

NMR Spectra (^{1}H, ^{13}C, ^{19}F): S141-S142

2-methoxyethyl 2-chloro-4-(2,6-dimethyl-4-propoxyphenyl)-6-methylpyrimidine-5-carboxylate (3m)

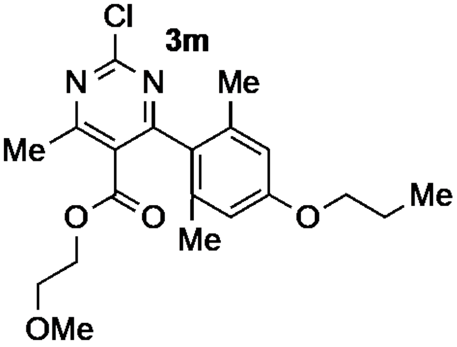

Prepared according to General Procedure D. Product was isolated by column chromatography (silica gel, 10% EtOAc:Hexanes) as a light orange solid (2.8 g, 38% yield).

Analytical data for **3m**:

^{1}H NMR (500 MHz, Chloroform-*d_1*) δ 6.59 (s, 2H, *m-**ArH***), 4.16 (t, *J* = 4.7 Hz, 2H, CO_2***CH_2***CH_2OCH_3), 3.90 (t, *J* = 6.6 Hz, 2H, *p*-ArO***CH_2***CH_2CH_3), 3.27 (m, 5H, CO_2CH_2***CH_2OCH_3***), 2.64 (s, 3H, –***CH_3***), 2.01 (s, 6H, *o-**$ArCH_3$***), 1.79 (h, *J* = 7.1 Hz, 2H, *p*-$ArOCH_2$***CH_2***CH_3), 1.03 (t, *J* = 7.4 Hz, 3H, *p*-$ArOCH_2CH_2$***CH_3***).

^{13}C NMR (126 MHz, Chloroform-*d_1*) δ 168.92, 168.76, 166.09, 161.11, 159.52, 137.05, 128.44, 126.91, 113.49, 69.93, 69.47, 64.60, 58.89, 22.90, 22.70, 20.15, 10.65.

IR (thin film, cm^{-1}): 2962, 2930, 1734, 1533, 1279, 1219, 1173, 1122, 1079, 864.

HRMS ***m/z*** **calculated for $C_{20}H_{26}ClN_2O_4^+$:** 393.1576 and 395.1547 **found**: 393.1585 and 395.1560.

NMR Spectra (^{1}H, ^{13}C): S143

isopropyl 2-chloro-4-mesityl-6-methylpyrimidine-5-carboxylate (3n)

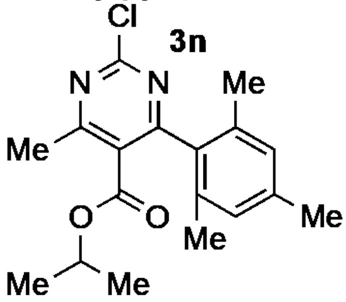

Prepared according to General Procedure D. Product was isolated by column chromatography (silica gel, 5% to 10% EtOAc:Hexanes) as an off white semi-crystalline solid (4.5 g, 56%).

Analytical data for **3n**:

^{1}H NMR (500 MHz, Chloroform-*d_1*) δ 6.86 (s, 2H, *m*-**ArH**), 4.93 (hept, *J* = 6.3 Hz, 1H, CO_2***CH***$(CH_3)_2$), 2.64 (s, 3H, –***CH_3***), 2.28 (s, 3H, *p-**$ArCH_3$***), 1.99 (s, 6H, *o-**$ArCH_3$***), 0.94 (d, *J* = 6.2 Hz, 6H, CO_2CH(***CH_3***)$_2$).

^{13}C NMR (126 MHz, Chloroform-*d_1*) δ 168.94, 168.52, 165.51, 160.92, 138.95, 135.23, 133.17, 128.20, 127.00, 69.93, 22.79, 21.26, 21.07, 19.80.

IR (thin film, cm^{-1}): 2986, 2920, 1716, 1526, 1230, 1086, 940, 857, 835, 626.

HRMS *m/z* **calculated for $C_{18}H_{22}ClN_2O_2^+$:** 333.1364 and 335.1335 **found**: 333.1367 and 335.1342.

NMR Spectra (^{1}H, ^{13}C): S144

isopropyl 2-chloro-4-(3,5-di-*tert*-butylphenyl)-6-methylpyrimidine-5-carboxylate (3o)

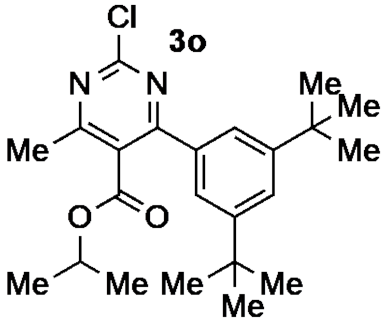

Prepared according to General Procedure D. Product was isolated by column chromatography (silica gel, 5% to 10% EtOAc:Hexanes) as an off-white solid in (8.3 g, 66% yield).

Analytical data for **3o**:

^{1}H NMR (500 MHz, Chloroform-*d₁*) δ 7.54 (t, *J* = 1.8 Hz, 1H, *p-**ArH***), 7.39 (d, *J* = 1.8 Hz, 2H, *o-**ArH***), 4.25 (hept, *J* = 6.3 Hz, 1H, CO_2***CH(***$CH_3)_2$), 2.61 (s, 3H, –***CH₃***), 1.33 (s, 18H, *m-**ArC(CH₃)₃***), 0.97 (d, *J* = 6.3 Hz, 6H, CO_2CH(***CH₃***$)_2$***)***.

^{13}C NMR (126 MHz, Chloroform-*d₁*) δ 168.22, 168.17, 166.82, 160.42, 151.46, 136.06, 125.19, 124.83, 122.65, 70.16, 35.12, 30.15, 22.52, 21.35.

IR (thin film, cm^{-1}): 2955, 2867, 1724, 1542, 1530, 1227, 1154, 1080, 908, 879, 714, 707.

HRMS *m/z* **calculated for $C_{23}H_{32}ClN_2O_2^+$:** 403.2147 and 405.2118 **found**: 403.2159 and 405.2135.

NMR Spectra (^{1}H, ^{13}C): S145

2-methoxyethyl 2-chloro-4-(2,6-dimethylphenyl)-6-methylpyrimidine-5-carboxylate (3p)

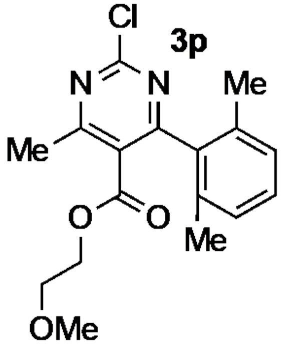

Prepared according to General Procedure D. isolated by column chromatography (5% to 10% to 50% EtOAc:Hexanes) as a brown solid (3.3 g, 77% yield).

Analytical data for **3p**:

^{1}H NMR (500 MHz, Chloroform-d_1) δ 7.20 (t, J = 7.6 Hz, 1H, *p*-***ArH***), 7.05 (d, J = 7.7 Hz, 2H, *m*-***ArH***), 4.14-4.10 (m, 2H, CO_2***CH_2***CH_2OCH_3), 3.25 (s, 3H, $CO_2CH_2CH_2$***OCH_3***), 3.21-3.18 (m, 2H, CO_2CH_2***CH_2***OCH_3), 2.67 (s, 3H, –***CH_3***), 2.04 (s, 6H, *o*-***$ArCH_3$***).

^{13}C NMR (126 MHz, Chloroform-d_1) δ 169.15, 168.86, 165.88, 161.27, 135.88, 135.38, 129.20, 127.52, 126.23, 69.78, 64.60, 58.86, 23.00, 19.87.

IR (thin film, cm^{-1}): 2925, 2829, 1734, 1547, 1527, 1300, 1218, 1200, 1078, 939, 862, 767.

HRMS *m/z* **calculated for $C_{17}H_{20}ClN_2O_3^+$:** 335.1157 and 337.1128 **found**: 335.1161 and 337.1135.

NMR Spectra (^{1}H, ^{13}C): S146

methyl 2-chloro-4,6-dimethylpyrimidine-5-carboxylate (3aB)

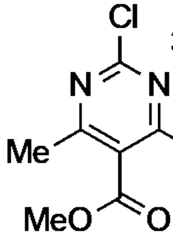

3aB

Prepared according to General Procedure D. Product was isolated by column chromatography (silica gel, 10% EtOAc:Hexanes) isolated as a flocculent white solid (3.6 g, 68% yield).

Analytical data for **3aB**:

^{1}H NMR (500 MHz, Chloroform-d_1) δ 3.97 (s, 3H, CO_2***CH_3***), 2.55 (s, 6H, –***CH_3***).

^{13}C NMR (126 MHz, Chloroform-d_1) δ 168.28, 167.07, 160.64, 125.11, 52.99, 23.06.

IR (thin film, cm^{-1}): 2961, 1721, 1537, 1419, 1305, 1227, 1090, 913, 786, 549.

HRMS *m/z* **calculated for $C_8H_{10}ClN_2O_2^+$:** 201.0425 and 203.0396 **found**: 201.0435 and 203.0407.

NMR Spectra (^{1}H, ^{13}C): S147

ethyl 2-chloro-4,6-dimethylpyrimidine-5-carboxylate (3aC)

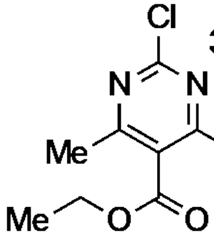

3aC

Prepared according to General Procedure D. Product was isolated via column chromatography (silica gel, 10% EtOAc:hexanes) as a white crystalline solid (7.9 g, 85% yield). Characterization matched previously reported data.[2]

isobutyl 2-chloro-4,6-dimethylpyrimidine-5-carboxylate (3aD)

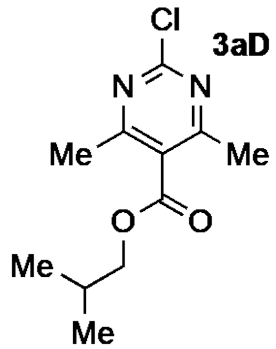

Prepared according to General Procedure D. Product was isolated via column chromatography (silica gel, 10% EtOAc:hexanes) as a yellow colored oil (5.6 g, 70% yield).

Analytical data for **3aD**:

^{1}H NMR (500 MHz, Chloroform-*d_1*) δ 4.16 (d, *J* = 6.6 Hz, 2H, CO_2***CH_2***CH(CH_3)$_2$), 2.55 (s, 6H, –***CH_3***), 2.06 (*apparent* hept, *J* = 6.7 Hz, 1H, CO_2CH_2***CH***(CH_3)$_2$), 1.01 (d, *J* = 6.7 Hz, 6H, CO_2CH_2CH(***CH_3***)$_2$). Xx adam check

^{13}C NMR (126 MHz, Chloroform-*d_1*) δ 168.04, 166.78, 160.44, 125.50, 72.54, 27.86, 23.06, 19.33.

IR (thin film, cm^{-1}): 2964, 2876, 1723, 1542, 1422, 1217, 1082, 916, 787.

HRMS *m/z* **calculated for $C_{11}H_{16}ClN_2O_2^+$:** 243.0895 and 245.0866 **found**: 243.0902 and 245.0848.

NMR Spectra (^{1}H, ^{13}C): S148

isopropyl 2-chloro-4-methyl-6-(*o*-tolyl)pyrimidine-5-carboxylate (3aE)

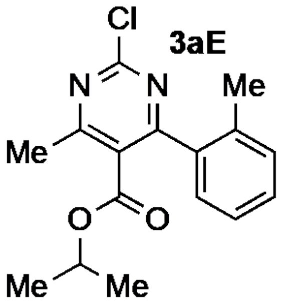

Prepared according to General Procedure D. Product was isolated via column chromatography (silica gel, 20% EtOAc:hexanes) as a viscous green oil (3.3 g, 28% yield).

Analytical data for **3aE**:

^{1}H NMR (500 MHz, Chloroform-*d_1*) δ 7.32 (t, *J* = 7.5 Hz, 1H, ***ArH***), 7.28-7.23 (m, 1H, ***ArH*** under $CDCl_3$), 7.20 (t, *J* = 7.5 Hz, 1H, ***ArH***), 7.12 (d, *J* = 7.6 Hz, 1H, ***ArH***), 4.93 (hept, *J* = 6.3 Hz, 1H, CO_2***CH***(CH_3)$_2$), 2.65 (s, 3H, –***CH_3***), 2.24 (s, 3H, *o*-***ArCH_3***), 0.93 (d, *J* = 6.2 Hz, 6H, CO_2CH(***CH_3***)$_2$).

^{13}C NMR (126 MHz, Chloroform-*d_1*) δ 168.80, 168.59, 165.68, 160.26, 136.38, 136.02, 130.61, 129.77, 128.23, 126.32, 125.70, 70.04, 22.80, 21.17, 19.68.

IR (thin film, cm^{-1}): 2981, 2934, 1722, 1528, 1225, 1080, 937, 867, 767, 751, 726.

HRMS *m/z* **calculated for $C_{16}H_{18}ClN_2O_2^+$:** 305.1051 and 307.1022 **found**: 305.1063 and 307.1035.

NMR Spectra (^{1}H, ^{13}C): S149

methyl 2-chloro-4-(2-fluoro-6-methoxyphenyl)-6-methylpyrimidine-5-carboxylate (3aF)

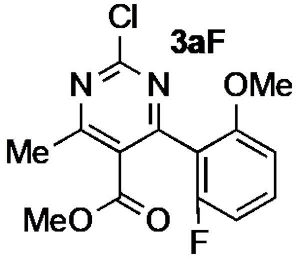

3aF

Prepared according to General Procedure D. Product was isolated by column chromatography (silica gel, 10% to 20% to 50% EtOAc:Hexanes) as an off-white crystalline solid (5.5 g, 35%).

Analytical data for **3aF**:

^{1}H NMR (500 MHz, Chloroform-*d₁*) δ 7.36 (q, *J* = 7.8 Hz, 1H, ***ArH***), 6.79 (t, *J* = 8.7 Hz, 1H, ***ArH***), 6.73 (d, *J* = 8.5 Hz, 1H, ***ArH***), 3.75 (s, 3H, *o*-***ArOCH₃***), 3.63 (s, 3H, CO_2***CH₃***), 2.69 (s, 3H, –***CH₃***). Xx check adam

^{13}C NMR (126 MHz, Chloroform-*d₁*) δ 169.64, 166.03, 161.83, 161.19, 160.91, 159.21, 157.59, 131.81, 126.32, 114.65, 108.65, 108.48, 106.49, 56.18, 52.50, 23.60.

^{19}F NMR (282 MHz, Chloroform-*d₁*) δ –114.49.

IR (thin film, cm^{-1}): 2952, 2845, 1729, 1615, 1528, 1470, 1438, 1281, 1230, 1068, 918, 862, 785, 753, 733.

HRMS *m/z* **calculated for $C_{14}H_{13}ClFN_2O_3^+$:** 311.0593 and 313.0564 **found**: 311.0604 and 313.0576.

NMR Spectra (^{1}H, ^{13}C, ^{19}F): S150-S151

dimethyl 4,4'-dimethyl-6,6'-bis(2-(trifluoromethyl)phenyl)-[2,2'-bipyrimidine]-5,5'-dicarboxylate (4a)

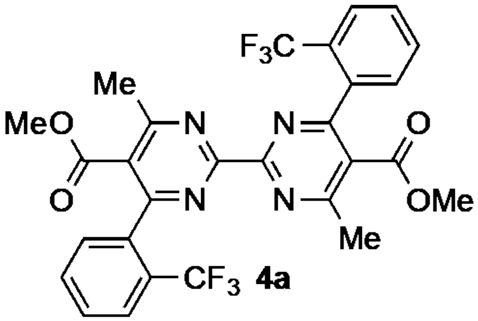

4a

Prepared according to General Procedure E. Product was isolated via column chromatography (silica gel, 10% to 50% EtOAc:hexanes) and then recrystallized from EtOAc:Hexanes (white solid, 190 mg, 8.1% yield). Characterization matched previously reported data.[2]

diethyl 4,4'-dimethyl-6,6'-bis(2-(trifluoromethyl)phenyl)-[2,2'-bipyrimidine]-5,5'-dicarboxylate (4b)

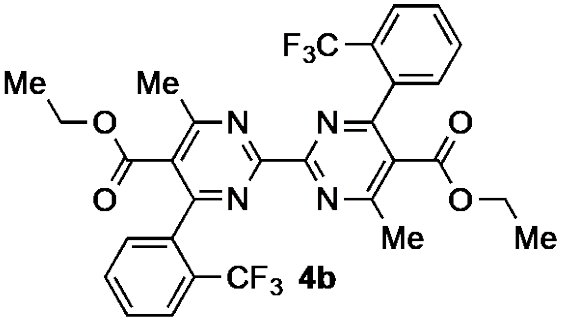

4b

Prepared according to General Procedure E. Product was isolated via column chromatography (10% to 50% EtOAc:hexanes) and then recrystallized from EtOAc:Hexanes (white crystals, 397 mg, 18% yield).

Analytical data for **4b**:

^{1}H NMR ^{1}H NMR (500 MHz, Chloroform-*d$_1$*) δ 7.79-7.72 (m, 2H, ***ArH***), 7.61-7.52 (m, 4H, ***ArH***), 7.41-7.35 (m, 2H, ***ArH***), 4.05 (q, *J* = 7.1 Hz, 4H, $CO_2\boldsymbol{CH_2}CH_3$), 2.83 (s, 6H, –$\boldsymbol{CH_3}$), 0.89 (t, *J* = 7.2 Hz, 6H, $CO_2CH_2\boldsymbol{CH_3}$).

^{13}C NMR (126 MHz, Chloroform-*d$_1$*) δ 167.26, 166.16, 164.90, 161.48, 136.45, 131.33, 130.45, 129.42, 129.10, 128.85, 126.77, 126.25, 124.88, 122.70, 61.84, 23.74, 13.53.

^{19}F NMR (282 MHz, Chloroform-*d$_1$*) δ -57.56.

IR (thin film, cm^{-1}): 2977, 1728, 1533, 1316, 1222, 1113, 1034, 768.

HRMS *m/z* **calculated for $C_{30}H_{25}F_6N_4O_4^+$:** 619.1775 **found**: 619.1780.

NMR Spectra (^{1}H, ^{13}C, ^{19}F): S152-S153

diisopropyl 4,4'-dimethyl-6,6'-bis(2-(trifluoromethyl)phenyl)-[2,2'-bipyrimidine]-5,5'-dicarboxylate (4c)

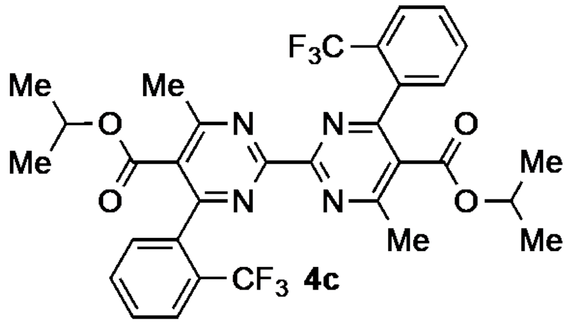

4c

Prepared according to General Procedure E. Product was isolated via column chromotography (silica gel, 10% to 50% EtOAc:hexanes) and then recrystallized from EtOAc (white crystals, 423 mg, 16% yield).

Analytical data for **4c**:

^{1}H NMR (500 MHz, Chloroform-*d_1*) δ 7.78-7.73 (m, 2H, ***ArH***), 7.59-7.52 (m, 4H, ***ArH***), 7.40-7.36 (m, 1H, ***ArH***), 4.95 (hept, *J* = 6.3 Hz, 2H, CO_2***CH***$(CH_3)_2$), 2.82 (s, 6H, –***CH_3***), 0.94 (s, 12H, CO_2CH(***CH_3***$)_2$).

^{13}C NMR (126 MHz, Chloroform-*d_1*) δ 166.99, 165.68, 164.61, 161.36, 136.37, 131.31, 130.56, 129.41, 128.93, 126.77, 126.55, 124.83, 122.64, 69.82, 60.55, 23.63, 21.17, 14.34.

^{19}F NMR (282 MHz, Chloroform-*d_1*) δ -57.55.

IR (thin film, cm^{-1}): 2984, 1717, 1537, 1314, 1230, 1111, 1082, 780.

HRMS *m/z* **calculated for $C_{32}H_{29}F_6N_4O_4^+$:** 647.2088 **found**: 647.2099.

NMR Spectra (^{1}H, ^{13}C, ^{19}F): S154-S155

diisobutyl 4,4'-dimethyl-6,6'-bis(2-(trifluoromethyl)phenyl)-[2,2'-bipyrimidine]-5,5'-dicarboxylate (4d)

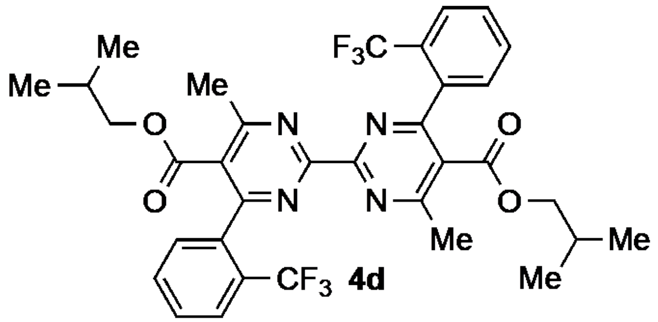

4d

Prepared according to General Procedure E. Product was isolated via column chromatography (silica gel, 5% to 10% to 25% to 50% EtOAc:hexanes) and recrystallized from MeCN:Et_2O (white crystals, 125 mg, 4.6% yield).

Analytical data for **4d**:

^{1}H NMR (500 MHz, Chloroform-*d_1*) δ 7.78-7.73 (m, 2H, ***ArH***), 7.59-7.51 (m, 4H, ***ArH***), 7.41-7.37 (m, 2H, ***ArH***), 3.78 (d, *J* = 6.5 Hz, 4H, CO_2***CH_2***$CH(CH_3)_2$), 2.83 (s, 6H, –***CH_3***), 1.60 (*apparent* hept, *J* = 6.8 Hz, 2H, CO_2CH_2***CH***$(CH_3)_2$), 0.71 (d, *J* = 6.7 Hz, 12H, CO_2CH_2CH(***CH_3***$)_2$). Xx check adam

^{13}C NMR (126 MHz, Chloroform-*d*$_1$) δ 167.15, 166.55, 164.59, 161.39, 136.38, 131.40, 130.45, 129.52, 128.90, 127.02, 126.42, 124.84, 122.67, 72.27, 27.44, 23.80, 18.92.

^{19}F NMR (282 MHz, Chloroform-*d*$_1$) δ -57.43.

IR (thin film, cm^{-1}): 2962, 1724, 1532, 1312, 1118, 1034, 760.

HRMS *m/z* **calculated for $C_{34}H_{33}F_6N_4O_4^+$:** 675.2401 **found**: 675.2416.

NMR Spectra (^{1}H, ^{13}C): S156-S157

bis(2-methoxyethyl) 4,4'-dimethyl-6,6'-bis(2-(trifluoromethyl)phenyl)-[2,2'-bipyrimidine]-5,5'-dicarboxylate (4e)

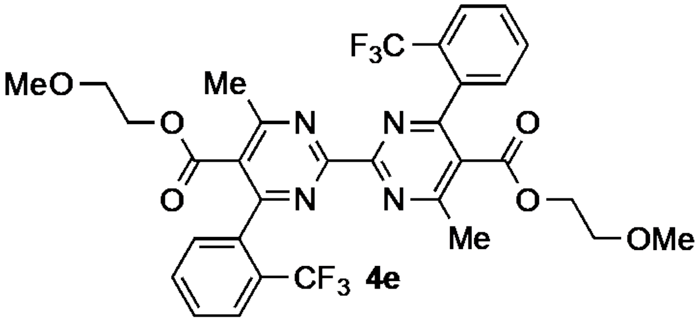

4e

Prepared according to General Procedure E. Product was isolated by column chromatography (silica gel, 5% to 10% to 25% to 50% EtOAc:Hexanes followed by an EtOAc flush) and recrystallized in EtOAc:Heptanes (light yellow crystals, 569 mg, 21% yield).

Analytical data for **4e**:

^{1}H NMR (500 MHz, Chloroform-*d*$_1$) δ 7.80-7.71 (m, 2H, ***ArH***), 7.60-7.52 (m, 4H, ***ArH***), 7.43-7.37 (m, 2H, ***ArH***), 4.21-4.08 (m, 4H, $CO_2\boldsymbol{CH_2}CH_2OCH_3$), 3.29-3.24 (m, 4H, $CO_2CH_2\boldsymbol{CH_2}OCH_3$), 3.22 (s, 6H, $CO_2CH_2CH_2\boldsymbol{OCH_3}$), 2.84 (s, 6H, $-\boldsymbol{CH_3}$).

^{13}C NMR (126 MHz, Chloroform-*d*$_1$) δ 167.40, 166.18, 164.91, 161.54, 136.40, 131.35, 130.50, 129.40, 129.10, 128.85, 128.60, 127.05, 126.79, 125.99, 124.87, 122.70, 120.51, 69.74, 64.63, 58.89, 23.79.

^{19}F NMR (282 MHz, Chloroform-*d*$_1$) δ -57.55.

IR (thin film, cm^{-1}): 2922, 1723, 1538, 1316, 1220, 1116, 1032, 766.

HRMS *m/z* **calculated for $C_{32}H_{29}F_6N_4O_6^+$:** 679.1986 **found**: 679.1998.

NMR Spectra (^{1}H, ^{13}C, ^{19}F): S158-S159

dimethyl 4,4'-dimethyl-6,6'-di-*o*-tolyl-[2,2'-bipyrimidine]-5,5'-dicarboxylate (4f)

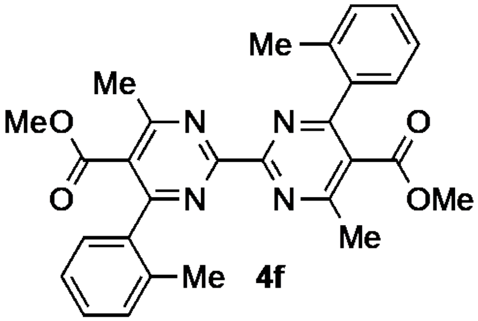

4f

Prepared according to General Procedure E. Product was isolated by column chromatography (silica gel, 10% to 30% EtOAc:hexanes) and recrystallized from EtOAc/hexanes (white crystals, 363 mg, 19%). Characterization matched previously reported data.[2]

dimethyl 4,4'-bis(2,6-dimethylphenyl)-6,6'-dimethyl-[2,2'-bipyrimidine]-5,5'-dicarboxylate (4g)

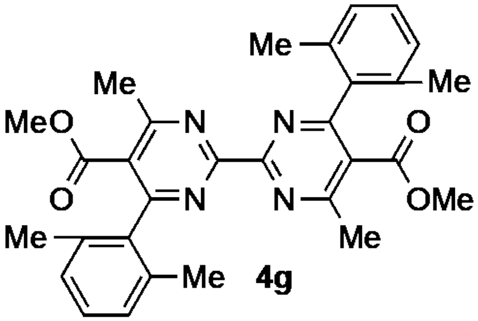

4g

Prepared according to General Procedure E. Product was isolated by column chromatography (silica gel, 10% to 50% EtOAc:hexanes) and recrystallized from EtOAc/Hexanes (light orange crystals, 481 mg, 23% yield).

Analytical data for **4g**:

^{1}H NMR (500 MHz, Chloroform-d_1) δ 7.17 (t, *J* = 7.6 Hz, 2H, *p-**ArH***), 7.04 (d, *J* = 7.5 Hz, 4H, *m-**ArH***), 3.56 (s, 6H, CO_2***CH₃***), 2.75 (s, 6H, –***CH₃***), 2.06 (s, 12H, *o-**ArCH₃***).

^{13}C NMR ^{13}C NMR (126 MHz, Chloroform-d_1) δ 167.31, 166.48, 166.13, 163.16, 136.76, 135.72, 128.93, 127.45, 52.57, 23.26, 20.09.

IR (thin film, cm^{-1}): 2953, 2925, 1732, 1532, 1220, 1078, 773.

HRMS *m/z* **calculated for $C_{30}H_{31}N_4O_4^+$:** 511.2340 **found:** 511.2349.

NMR Spectra (^{1}H, ^{13}C): S160

dimethyl 4,4'-dimesityl-6,6'-dimethyl-[2,2'-bipyrimidine]-5,5'-dicarboxylate (4h)

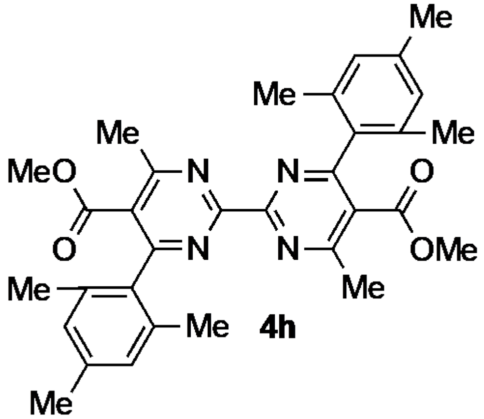

4h

Prepared according to General Procedure E. Product was isolated by column chromatography (silica gel, 10% to 50% EtOAc:hexanes) and recrystallized from EtOAc/hexanes (white crystals, 267 mg, 25%). Characterization matched previously reported data.[2]

dimethyl 4,4'-bis(2,6-dimethyl-4-propoxyphenyl)-6,6'-dimethyl-[2,2'-bipyrimidine]-5,5'-dicarboxylate (4i)

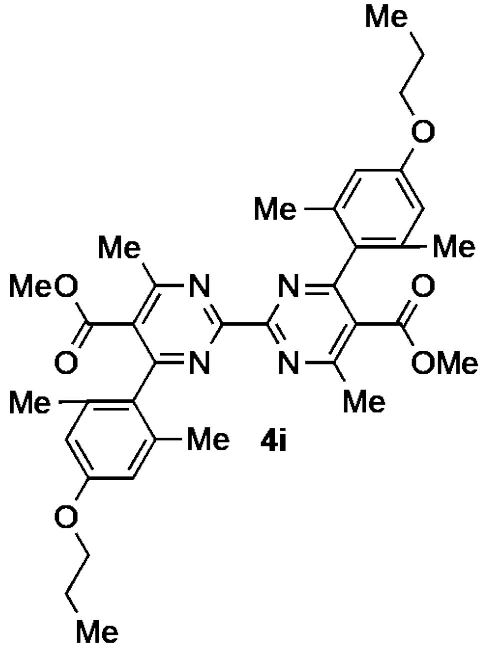

4i

Prepared according to General Procedure E. Product was isolated by column chromatography (10% to 50% EtOAc:Hexanes) and then recrystallized from EtOAc:Hexanes (white crystals, 324 mg, 15% yield).

Analytical data for **4i**:

^{1}H NMR (400 MHz, Chloroform-*d_1*) δ 6.59 (s, 4H, *m-**ArH***), 3.90 (t, *J* = 6.6 Hz, 4H, *p*-ArO***CH_2***CH_2CH_3), 3.60 (s, 6H, CO_2***CH_3***), 2.73 (s, 6H, –***CH_3***), 2.03 (s, 12H, *o-**ArCH_3***), 1.79 (h, *J* = 7.1 Hz, 4H, *p*-ArOCH_2***CH_2***CH_3), 1.03 (t, *J* = 7.4 Hz, 6H, *p*-ArOCH_2CH_2***CH_3***).

^{13}C NMR (101 MHz, Chloroform-*d_1*) δ 167.52, 166.45, 165.82, 163.06, 159.25, 137.34, 129.42, 128.05, 113.43, 69.41, 52.66, 23.19, 22.73, 20.38, 10.66.

IR (thin film, cm^{-1}): 2952, 2878, 1735, 1533, 1223, 1082, 836.

HRMS *m/z* **calculated for $C_{36}H_{43}N_4O_6^+$:** 627.3177 **found**: 627.3184.

NMR Spectra (^{1}H, ^{13}C): S161

dimethyl 4,4'-bis(3,5-di-*tert*-butylphenyl)-6,6'-dimethyl-[2,2'-bipyrimidine]-5,5'-dicarboxylate (4j)

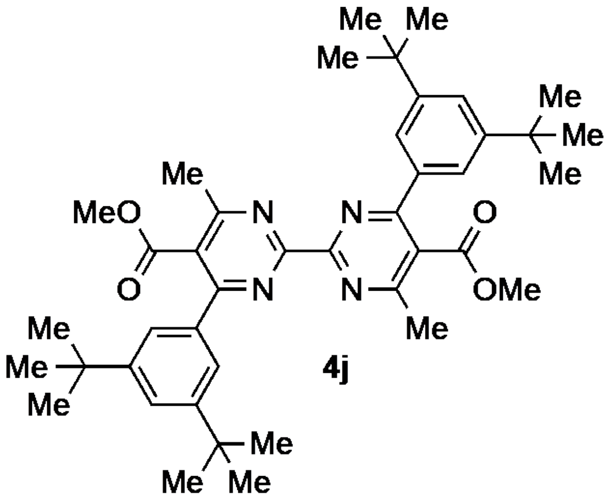

4j

Prepared according to General Procedure E. Product was isolated by column chromatography (5% to 10% to 50% EtOAc:Hexanes) and then recrystallized from EtOAc:Hexanes (pale yellow crystals, 329 mg, 12% yield).

Analytical data for **4j**:

^{1}H NMR (500 MHz, Chloroform-*d$_1$*) δ 7.59-7.56 (d, *J* = 1.8 Hz, 4H, *o*-***ArH***), 7.51 (t, *J* = 1.8 Hz, 2H, *p*-***ArH***), 3.75 (s, 6H, CO$_2$***CH$_3$***), 2.76 (s, 6H, –***CH$_3$***), 1.34 (s, 36H, *m*-***ArC***(***CH$_3$***)$_3$).

^{13}C NMR (126 MHz, Chloroform-*d$_1$*) δ 168.84, 165.97, 165.62, 162.24, 151.20, 137.03, 125.81, 124.47, 123.12, 52.85, 35.12, 31.62, 23.11.

IR (thin film, cm^{-1}): 2955, 1725, 1224, 1083, 865, 710.

HRMS *m/z* **calculated for $C_{42}H_{55}N_4O_4^+$:** 679.4218 **found**: 679.4222.

NMR Spectra (^{1}H, ^{13}C): S162

dimethyl 4,4'-bis(3,5-dimethylphenyl)-6,6'-dimethyl-[2,2'-bipyrimidine]-5,5'-dicarboxylate (4k)

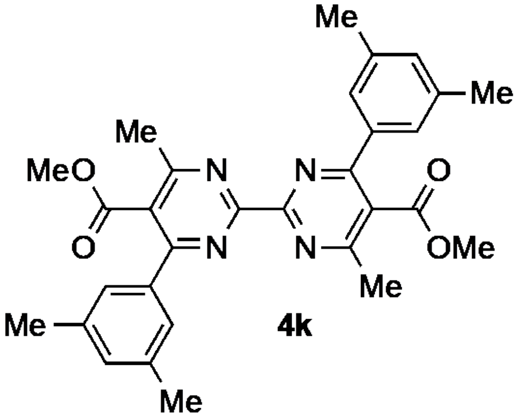

4k

Prepared according to General Procedure E. Product was isolated via column chromatography (silica gel, 10% to 25% EtOAc:Hexanes) and then recrystallized from EtOAc:Hexanes (white crystals, 172 mg, 6% yield).

Analytical data for **4k**:

^{1}H NMR (500 MHz, Chloroform-*d$_1$*) δ 7.40 (s, 4H, *o-**ArH***), 7.11 (s, 2H, *p-**ArH***), 3.77 (s, 6H, CO_2***CH$_3$***), 2.76 (s, 6H, –***CH$_3$***), 2.37 (s, 12H, *m-**ArCH$_3$***).

^{13}C NMR (126 MHz, Chloroform-*d$_1$*) δ 168.71, 166.28, 164.58, 162.40, 138.37, 137.41, 132.26, 126.52, 125.50, 52.83, 23.12, 21.46.

IR (thin film, cm^{-1}): 2951, 1732, 1533, 1223, 1083, 860, 703.

HRMS *m/z* **calculated for $C_{30}H_{31}N_4O_4^+$:** 511.2340 **found**: 511.2345.

NMR Spectra (^{1}H, ^{13}C): S163

dimethyl 4,4'-bis(3,5-bis(trifluoromethyl)phenyl)-6,6'-dimethyl-[2,2'-bipyrimidine]-5,5'-dicarboxylate (4l)

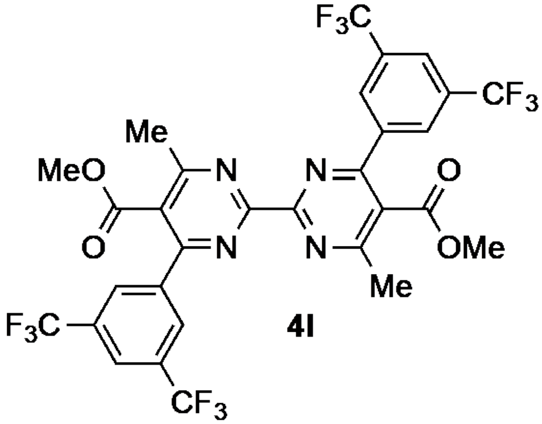

4l

Prepared according to General Procedure E. Product isolated via column chromatography (10% to 25% to 50% EtOAc:Hexanes) and then recrystallized from EtOAc:Hexanes (off-white flocculent solid, 344 mg, 14% yield).

Analytical data for **4l**:

^{1}H NMR (500 MHz, Chloroform-*d_1*) δ 8.26 (s, 4H, *o*-***ArH***), 8.03 (s, 2H, *p*-***ArH***), 3.84 (s, 6H, CO_2***CH_3***), 2.84 (s, 6H, –***CH_3***).

^{13}C NMR (126 MHz, Chloroform-*d_1*) δ 167.86, 167.46, 161.95, 161.23, 139.19, 132.83, 132.57, 132.30, 132.03, 129.06, 126.34, 125.97, 124.23, 124.17, 122.00, 119.83, 53.28, 23.41.

^{19}F NMR (282 MHz, Chloroform-*d_1*) δ -62.99.

IR (thin film, cm^{-1}): 2962, 1725, 1544, 1179, 1115, 1088, 903, 684.

HRMS *m/z* **calculated for $C_{30}H_{19}F_{12}N_4O_4^+$:** 727.1209 **found**: 727.1220.

NMR Spectra (^{1}H, ^{13}C, ^{19}F): S164-S165

bis(2-methoxyethyl) 4,4'-bis(2,6-dimethyl-4-propoxyphenyl)-6,6'-dimethyl-[2,2'-bipyrimidine]-5,5'-dicarboxylate (4m)

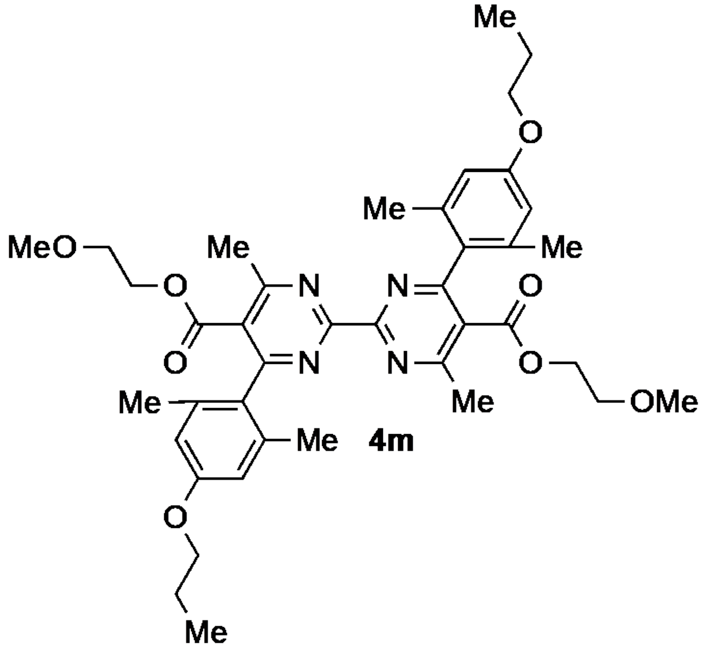

4m

Prepared according to General Procedure E. Product was isolated via column chromatography (silica gel, 10% to 50% EtOAc:Hexanes) and recrystallized from EtOAc:Hexanes (off-white crystals, 730 mg, 34% yield).

Analytical data for **4m**:

^{1}H NMR (500 MHz, Chloroform-*d_1*) δ 6.59 (s, 4H, *m*-***ArH***), 4.17 (m, 4H, CO_2***CH_2***CH_2OCH_3), 3.90 (t, *J* = 6.6 Hz, 4H, *p*-ArO***CH_2***CH_2OCH_3), 3.28-3.25 (m, 10H, CO_2CH_2***CH_2OCH_3***), 2.75 (s, 6H, –***CH_3***), 2.04 (s, 12H, *o*-***$ArCH_3$***), 1.79 (h, *J* = 7.1 Hz, 4H, *p*-$ArOCH_2$***CH_2***CH_3), 1.03 (t, *J* = 7.4 Hz, 6H, *p*-$ArOCH_2CH_2$***CH_3***).

^{13}C NMR (126 MHz, Chloroform-*d_1*) δ 166.97, 166.35, 166.06, 163.09, 159.28, 137.52, 129.62, 127.88, 113.43, 70.00, 69.43, 64.41, 58.91, 23.20, 22.72, 20.40, 10.66.

IR (thin film, cm^{-1}): 2969, 2926, 2880, 1722, 1537, 1220, 1079, 854.

HRMS *m/z* **calculated for $C_{40}H_{51}N_4O_8^+$:** 715.3701 **found**: 715.3697.

NMR Spectra (^{1}H, ^{13}C): S166

diisopropyl 4,4'-dimesityl-6,6'-dimethyl-[2,2'-bipyrimidine]-5,5'-dicarboxylate (4n)

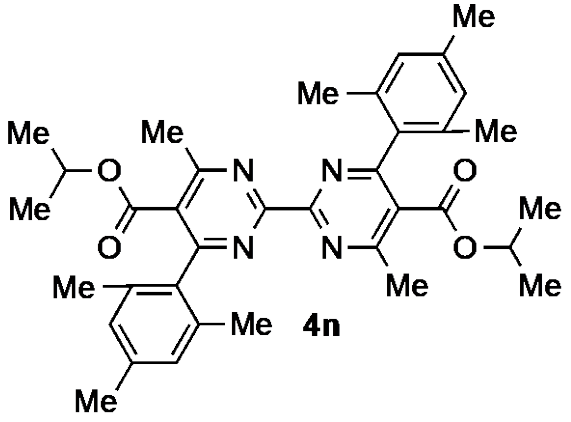

4n

Prepared according to General Procedure E. Product isolated via column chromatography (silica gel, 10% to 50% EtOAc:Hexanes) and then recrystallized from EtOAc:Hexanes (white crystals, 396 mg, 17%).

Analytical data for **4n**:

^{1}H NMR (500 MHz, Chloroform-*d₁*) δ 6.84 (s, 4H, *m-**ArH***), 4.95 (hept, *J* = 5.9 Hz, 2H, CO_2***CH***$(CH_3)_2$), 2.74 (s, 6H, –***CH₃***), 2.27 (s, 6H, *p-**ArCH₃***), 2.02 (s, 12H, *o-**ArCH₃***), 0.96 (d, *J* = 6.3 Hz, 12H, CO_2CH(***CH₃***)$_2$).

^{13}C NMR (126 MHz, Chloroform-*d₁*) δ 166.39, 166.34, 165.73, 163.04, 138.43, 135.74, 134.23, 128.10, 127.97, 69.57, 23.07, 21.26, 21.12, 20.04.

IR (thin film, cm^{-1}): 2979, 2937, 1714, 1531, 1373, 1227, 1079, 854.

HRMS *m/z* **calculated for $C_{36}H_{43}N_4O_4^+$:** 595.3279 **found**: 595.3289.

NMR Spectra (^{1}H, ^{13}C): S167

diisopropyl 4,4'-bis(3,5-di-*tert*-butylphenyl)-6,6'-dimethyl-[2,2'-bipyrimidine]-5,5'-dicarboxylate (4o)

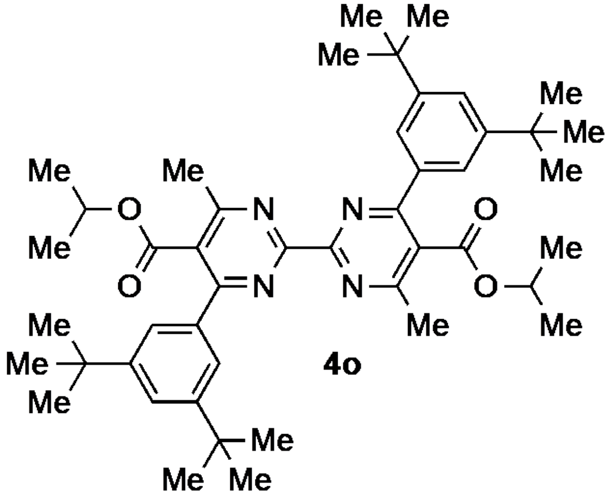

4o

Prepared according to General Procedure E. Product was isolated on column chromatography (silica gel, 5% to 10% to 25% EtOAc:Hexanes) and recrystallized from EtOAc:Hexanes (off-white crystals, 276 mg, 3% yield).

Analytical data for **4o**:

^{1}H NMR (500 MHz, Chloroform-*d$_1$*) δ 7.49 (s, 6H, *p-**ArH*** and *o-**ArH***), 4.97 (hept, *J* = 6.3 Hz, 2H, CO_2***CH***$(CH_3)_2$), 2.77 (s, 6H, –***CH$_3$***), 1.33 (s, 36H, *m-**ArC**(**CH$_3$**)$_3$*), 0.98 (d, *J* = 6.3 Hz, 12H, CO_2CH(***CH$_3$***)$_2$).

^{13}C NMR (126 MHz, Chloroform-*d$_1$*) δ 167.59, 166.23, 165.76, 162.19, 151.10, 137.44, 126.35, 124.06, 123.09, 69.77, 35.09, 31.57, 22.92, 21.39.

IR (thin film, cm^{-1}): 2952, 2866, 1729, 1538, 1221, 1079, 876, 711, 695.

HRMS *m/z* **calculated for $C_{46}H_{63}N_4O_4^+$:** 735.4844 **found**: 735.4843.

NMR Spectra (^{1}H, ^{13}C): S168

bis(2-methoxyethyl) 4,4'-bis(2,6-dimethylphenyl)-6,6'-dimethyl-[2,2'-bipyrimidine]-5,5'-dicarboxylate (4p)

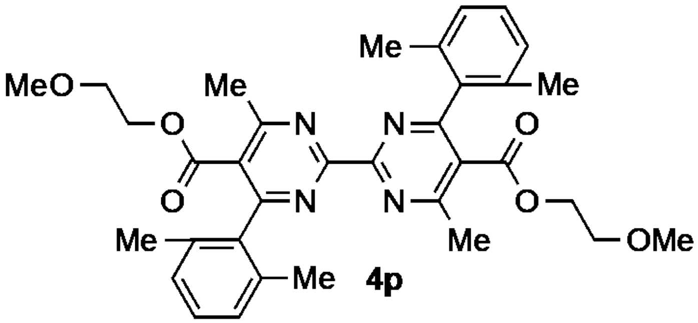

4p

Prepared according to General Procedure E. Product was isolated on column chromatography (silica gel, 10% to 25% to 50% EtOAc:Hexanes) and recrystallized from EtOAc:hexanes (light yellow crystals, 314 mg, 13% yield).

Analytical data for **4p**:

^{1}H NMR (500 MHz, Chloroform-*d$_1$*) 7.17 (t, *J* = 7.6 Hz, 2H, *p-**ArH***), 7.04 (d, *J* = 7.6 Hz, 4H, *m-**ArH***), 4.16-4.11 (m, 4H, CO_2***CH$_2$***CH_2OCH_3), 3.26 (s, 6H, $CO_2CH_2CH_2$***OCH$_3$***), 3.23-3.19 (m, 4H, CO_2CH_2***CH$_2$***OCH_3), 2.77 (s, 6H, –***CH$_3$***), 2.07 (s, 12H, *o-**ArCH$_3$***).

^{13}C NMR δ (126 MHz, Chloroform-*d$_1$*) δ 166.75, 166.42, 166.37, 163.11, 136.92, 135.91, 128.86, 127.45, 127.28, 69.85, 64.42, 58.88, 23.29, 20.10.

IR (thin film, cm^{-1}): 2950, 2923, 2893, 1728, 1540, 1219, 1076, 863, 784.

HRMS *m/z* **calculated for $C_{34}H_{39}N_4O_6^+$:** 599.2864 **found**: 599.2880.

NMR Spectra (^{1}H, ^{13}C): S169

dimethyl 4,4',6,6'-tetramethyl-[2,2'-bipyrimidine]-5,5'-dicarboxylate (4aB)

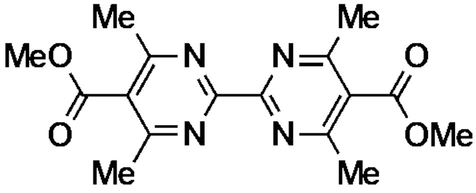

4aB

Prepared according to General Procedure D. Product was purified via column chromatography (silica gel, 20% EtOAc:DCM) and recrystallized from EtOAc:Hexanes (flocculent white solid, 250 mg, 9 % yield).

Analytical data for **4aB**:

^{1}H NMR (500 MHz, Chloroform-*d_1*) δ 3.99 (s, 6H, CO_2***CH_3***), 2.70 (s, 12H, –***CH_3***).

^{13}C NMR (126 MHz, Chloroform-*d_1*) δ 167.80, 165.89, 161.81, 126.62, 52.91, 23.43.

IR (thin film, cm^{-1}): 2958, 2929, 1717, 1541, 1437, 1293, 1224, 1085, 860, 788.

HRMS *m/z* **calculated for $C_{16}H_{19}N_4O_4^+$:** 331.1401 **found**: 331.1410.

NMR Spectra (^{1}H, ^{13}C): S170

diethyl 4,4',6,6'-tetramethyl-[2,2'-bipyrimidine]-5,5'-dicarboxylate (4aC)

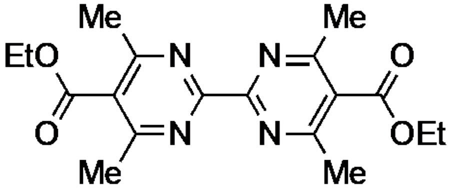

4aC

Prepared according to General Procedure E. Product was purified via column chromatography (silica gel, 20% EtOAc:DCM) and recrystallized from EtOAc:Hexanes (off-white crystals, 196 mg, 9% yield). Characterization matched previously reported data.[2]

diisobutyl 4,4',6,6'-tetramethyl-[2,2'-bipyrimidine]-5,5'-dicarboxylate (4aD)

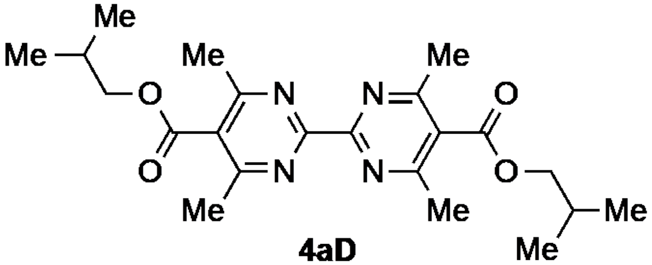

4aD

Prepared according to General Procedure E. Product was purified via column chromatography (20% EtOAc:DCM) to obtain the product as a viscous light yellow oil/low melting off-white solid (599 mg, 18% yield).

Analytical data for **4aD**:

^{1}H NMR (500 MHz, Chloroform-*d$_1$*) δ 4.18 (d, *J* = 6.6 Hz, 4H, CO_2***CH_2***CH(CH_3)$_2$), 2.70 (s, 12H, –***CH_3***), 2.08 (*apparent* hept, *J* = 6.7 Hz, 2H, CO_2CH_2***CH***(CH_3)$_2$), 1.01 (d, *J* = 6.7 Hz, 12H, CO_2CH_2CH(***CH_3***)$_2$). Xx check adam

^{13}C NMR (126 MHz, Chloroform-*d$_1$*) δ 167.48, 165.61, 161.84, 126.94, 72.35, 27.89, 23.39, 19.32.

IR (thin film, cm^{-1}): 2963, 2875, 1723, 1543, 1291, 1216, 1081.

HRMS *m/z* **calculated for $C_{22}H_{31}N_4O_4^+$:** 415.2340 **found**: 415.2343.

NMR Spectra (^{1}H, ^{13}C): S171

diisopropyl 4,4'-dimethyl-6,6'-di-*o*-tolyl-[2,2'-bipyrimidine]-5,5'-dicarboxylate (4aE)

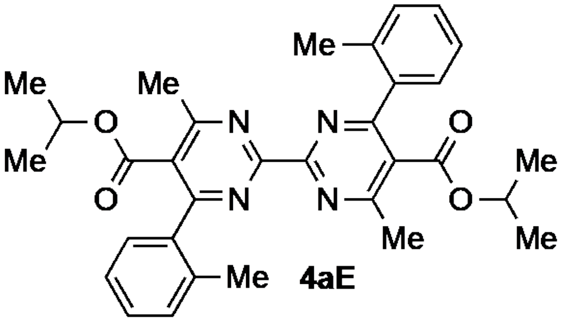

4aE

Prepared according to General Procedure E. Product was purified via column chromatography (silica gel, 10% EtOAc:DCM) and recrystallized in EtOAc:Hexanes (flocculent white solid, 537 mgs, 25% yield.

Analytical data for **4aE**:

^{1}H NMR (500 MHz, Chloroform-*d$_1$*) δ 7.29 (t, *J* = 7.4 Hz, 2H, ***ArH***), 7.25-7.21 (m, 4H, ***ArH***), 7.18 (t, *J* = 7.4 Hz, 2H, ***ArH***), 4.96 (hept, *J* = 6.2 Hz, 2H, CO_2***CH***(CH_3)$_2$), 2.77 (s, 6H, –***CH_3***), 2.27 (s, 6H, *o*-***ArCH_3***), 0.96 (d, *J* = 6.3 Hz, 12H, CO_2CH(***CH_3***)$_2$).

^{13}C NMR (126 MHz, Chloroform-*d$_1$*) δ 166.52, 166.29, 166.16, 162.10, 137.34, 136.54, 130.52, 129.40, 128.85, 127.45, 125.53, 69.75, 23.13, 21.24, 19.90.

IR (thin film, cm^{-1}): 2979, 2933, 1719, 1534, 1223, 1079, 778, 754.

HRMS *m/z* **calculated for $C_{32}H_{35}N_4O_4^+$:** 539.2653 **found**: 539.2656.

NMR Spectra (^{1}H, ^{13}C): S172

dimethyl 4,4'-bis(2-fluoro-6-methoxyphenyl)-6,6'-dimethyl-[2,2'-bipyrimidine]-5,5'-dicarboxylate (4aF)

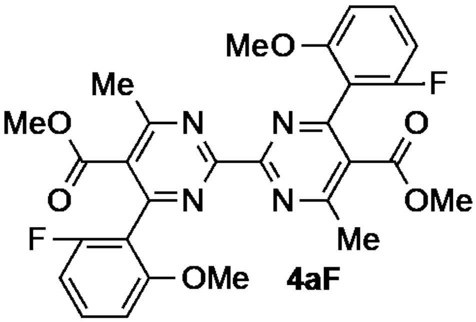

4aF

Prepared according to General Procedure E. Product isolated via column chromatography (silica gel, 10% to 20% to 50% EtOAc:Hexanes) and recrystallized from EtOAc:heptane (white crystals, 798 mg, 36%). Analytical data for **4aF**:

^{1}H NMR (500 MHz, Chloroform-*d_1*) δ 7.35-7.28 (m, 2H, ***ArH***), 6.77 (t, *J* = 8.7 Hz, 2H, ***ArH***), 6.70 (d, *J* = 8.4 Hz, 2H, ***ArH***), 3.72 (s, 6H, *o*-***$ArOCH_3$***), 3.65 (s, 6H, CO_2***CH_3***), 2.81 (s, 6H, –***CH_3***).

^{13}C NMR (126 MHz, Chloroform-*d_1*) δ 166.89, 166.86, 162.79, 161.60, 159.77, 159.62, 158.00, 131.28, 131.20, 127.55, 115.98, 108.64, 108.47, 106.54, 56.17, 53.56, 52.34, 23.90.

^{19}F NMR (282 MHz, Chloroform-*d_1*) δ -113.90, -113.99.

IR (thin film, cm^{-1}): 3044, 3013, 2971, 2954, 2841, 1724, 1618, 1538, 1470, 1227, 1082, 844, 729.

HRMS *m/z* **calculated for $C_{28}H_{25}F_2N_4O_6^+$:** 551.1737 **found**: 551.1755.

NMR Spectra (^{1}H, ^{13}C, ^{19}F): S173-S174

VII. References:

(1) Y. Yamamoto, K., Y. G. Chen and F. G. Buono, *Org. Lett.* **2005**, *7*, 4673-4676.

(2) J. D. Griffin, A. R. Pancoast and M. S. Sigman, *J. Am. Chem. Soc.* **2021**, *143*, 992-1004.

(3) B. T. Boyle, M. C. Hilton and A. McNally, *J. Am. Chem. Soc.* **2019**, *141*, 15441-15449.

(4) H. Wang, S. S. Sayed, E. J. Luber, B. C. Olsen, S. M. Shirurkar, S. Venkatakrishnan, U. M. Tefashe, A. K. Farquhar, E. S. Smotkin, R. L. McCreery and J. M. Buriak, *ACS Nano* **2020**, *14*, 2575-2584.

(5) A. J. Bard and L. R. Faulkner, Potential Sweep Methods. In *Electrochemical Methods: Fundamentals and Applications*, 2nd Edition; John Wiley & Sons, Inc.: Hoboken, New Jersey, United States of America, 2001; p 231.

(6) (*a*) C. S. Sevov, S. K. Samaroo and M. S. Sanford, M.S. *Adv. Energy Mater.* **2017**, *7*, 1602027 (*b*) S. G. Robinson, Y. Yan, K. H. Hendriks, M. S. Sanford and M. S. Sigman, *J. Am. Chem. Soc.* **2019**, *141*, 10171-10176 (c) Y. Yan, D. B. Vogt, T. P. Vaid, M. S. Sigman, M. S. Sanford, *Angew. Chem. Int. Ed.* **2021**, *60*, 27039-27045.

(7) N. G. Connelly and W. E. Geiger, *Chem. Rev.* **1996**, *96*, 877-910.

(8) (*a*) C. Lu, C. Wu, C., D. Ghoreishi, W. Chen, L. Wang, W. Damm, G. A. Ross, M. K. Dahlgren, E. Russell, C. D. Von Bargen, R. Abel, R. A. Friesner and E. D. Harder, *J. Chem. Theory Comput.* **2021**, *17*, 4291-4300 (*b*) **Schrödinger Release 2022-1**: MacroModel, Schrödinger, LLC, New York, NY, 2021.

(9) Gaussian 16, Revision C.01, Frisch, M. J.; Trucks, G. W.; Schlegel, H. B.; Scuseria, G. E.; Robb, M. A.; Cheeseman, J. R.; Scalmani, G.; Barone, V.; Petersson, G. A.; Nakatsuji, H.; Li, X.; Caricato, M.; Marenich, A. V.; Bloino, J.; Janesko, B. G.; Gomperts, R.; Mennucci, B.; Hratchian, H. P.; Ortiz, J. V.; Izmaylov, A. F.; Sonnenberg, J. L.; Williams-Young, D.; Ding, F.; Lipparini, F.; Egidi, F.; Goings, J.; Peng, B.; Petrone, A.; Henderson, T.; Ranasinghe, D.; Zakrzewski, V. G.; Gao, J.; Rega, N.; Zheng, G.; Liang, W.; Hada, M.; Ehara, M.; Toyota, K.; Fukuda, R.; Hasegawa, J.; Ishida, M.; Nakajima, T.; Honda, Y.; Kitao, O.; Nakai, H.; Vreven, T.; Throssell, K.; Montgomery, J. A., Jr.; Peralta, J. E.; Ogliaro, F.; Bearpark, M. J.; Heyd, J. J.; Brothers, E. N.; Kudin, K. N.; Staroverov, V. N.; Keith, T. A.; Kobayashi, R.; Normand, J.; Raghavachari, K.; Rendell, A. P.; Burant, J. C.; Iyengar, S. S.; Tomasi, J.; Cossi, M.; Millam, J. M.; Klene, M.; Adamo, C.; Cammi, R.; Ochterski, J. W.; Martin, R. L.; Morokuma, K.; Farkas, O.; Foresman, J. B.; Fox, D. J. Gaussian, Inc., Wallingford CT, 2016.

(10) (*a*) Y. Zhao and D. G. Truhlar, *Theor. Chem. Acc.* **2008**, *120*, 215-241 (*b*) V. Barone and M. Cossi, *J. Phys. Chem. A* **1998**, *102*, 1995-2001 (*c*) M. Cossi, N. Rega, G. Scalmani, and V. Barone, *J. Comput. Chem.* **2003**, *24*, 669-681.

(11) ***NBO 7.0.*** E. D. Glendening, J, K. Badenhoop, A. E. Reed, J. E. Carpenter, J. A. Bohmann, C. M. Morales, P. Karafiloglou, C. R. Landis, and F. Weinhold, Theoretical Chemistry Institute, University of Wisconsin, Madison (2018).

(12) **Schrödinger Release 2022-1**: QikProp, Schrödinger, LLC, New York, NY, 2021.

(13) S. H. Newman-Stonebraker, S. R. Smith, J. E. Borowski, E. Peters, T. Gensch, H. C. Johnson, M. S. Sigman and A. G. Doyle, *Science* **2021**, *374* (6565), 301-308.

(14) https://github.com/SigmanGroup/Threshold

(15) https://zenodo.org/record/5227162#.ZCc6aeLMIqs

VIII. ^{1}H, ^{13}C, and ^{19}F NMR Spectra:

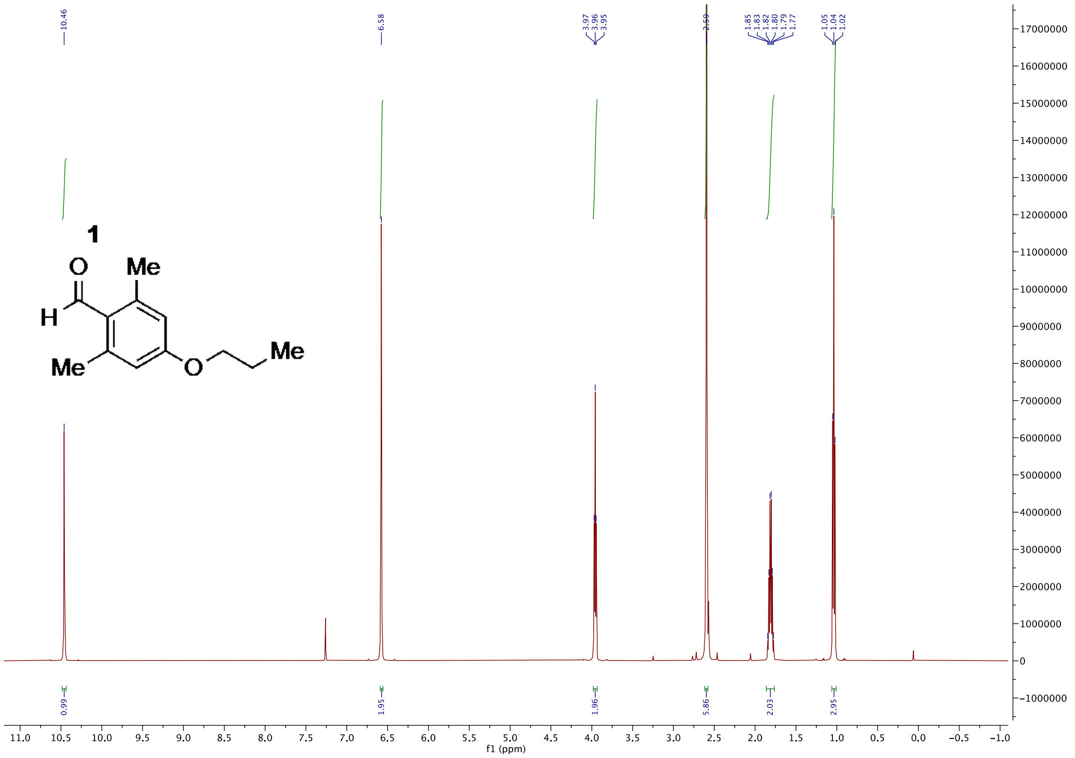

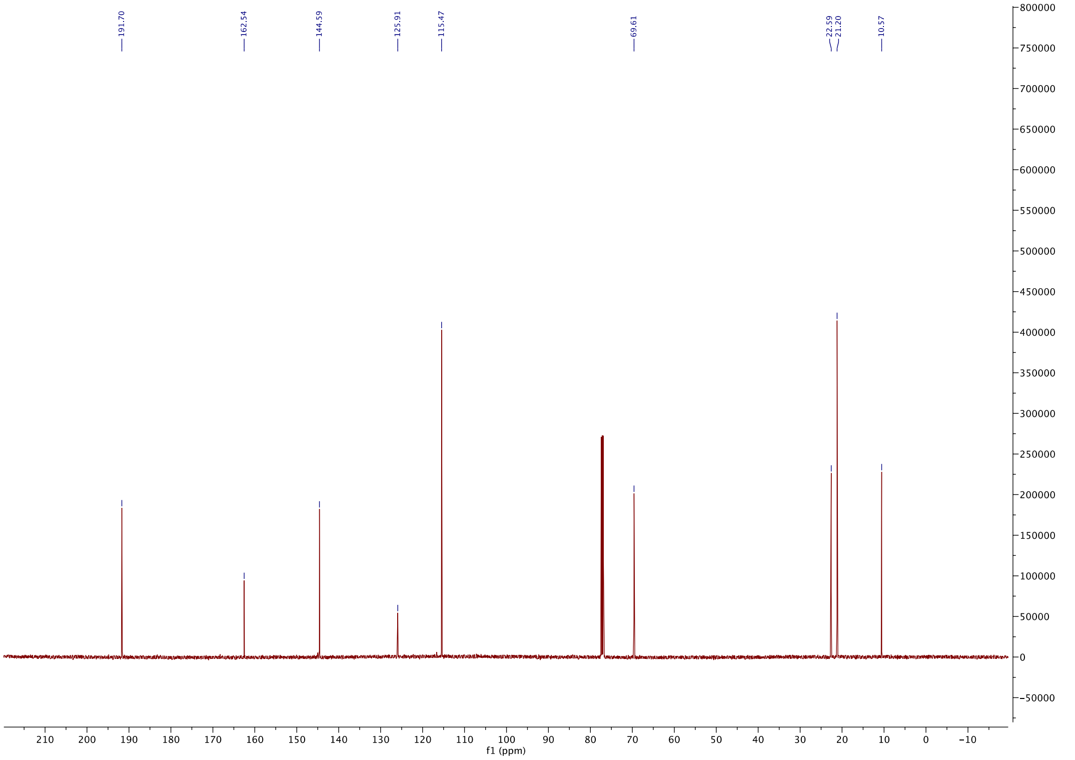

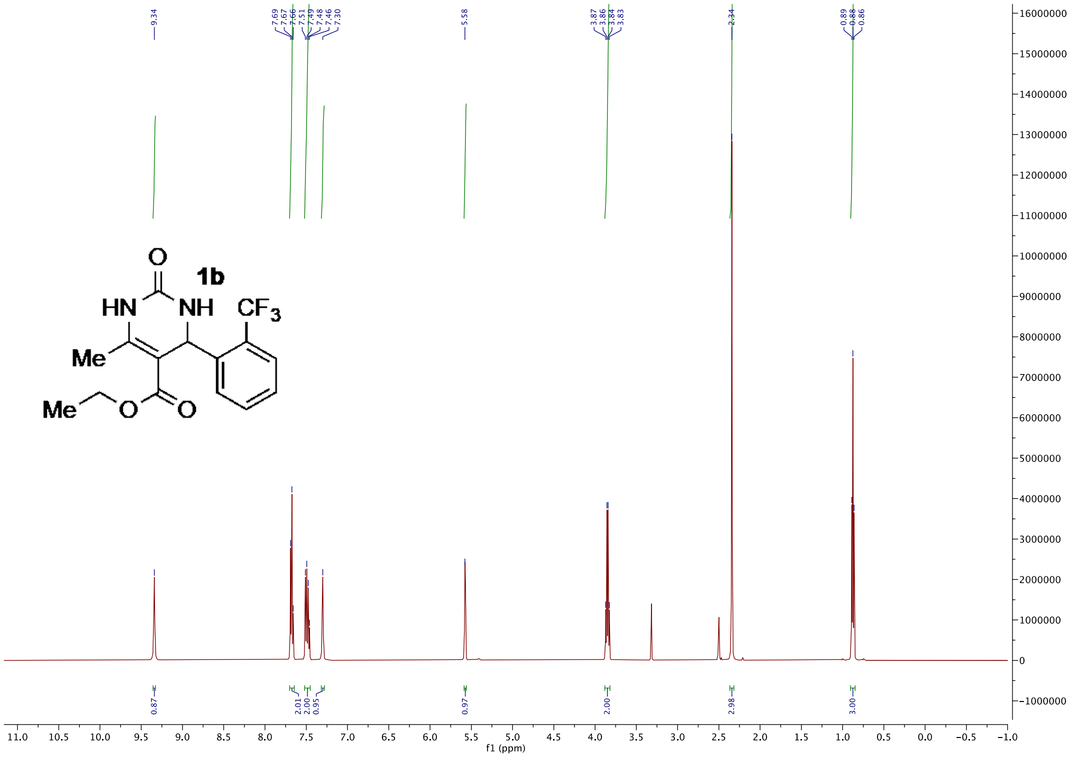
9.34
7.69
7.67
7.66
7.51
7.49
7.48
7.46
7.30
5.58
3.87
3.86
3.84
3.83
2.34
0.89
0.88
0.86
O
1b
HN
NH
CF3
Me
Me
O
O
0.87
2.01
2.00
0.95
0.97
2.00
2.98
3.00
f1 (ppm)

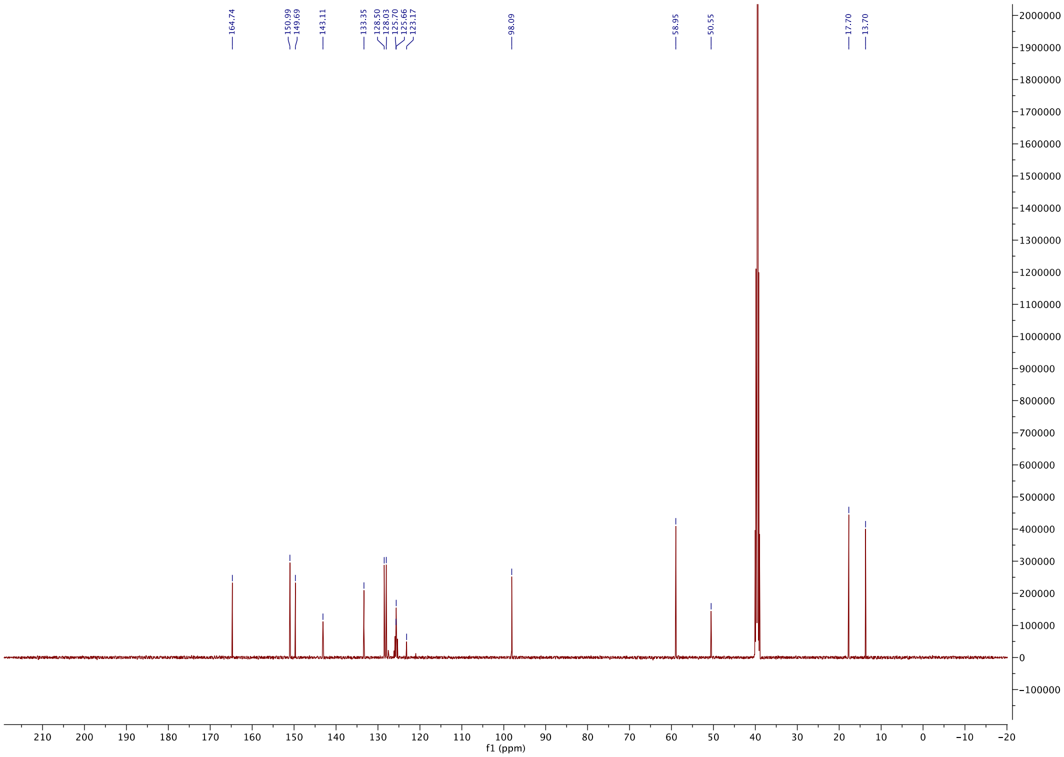
164.74
150.99
149.69
143.11
133.35
128.50
128.03
125.70
125.66
123.17
98.09
58.95
50.55
17.70
13.70
f1 (ppm)

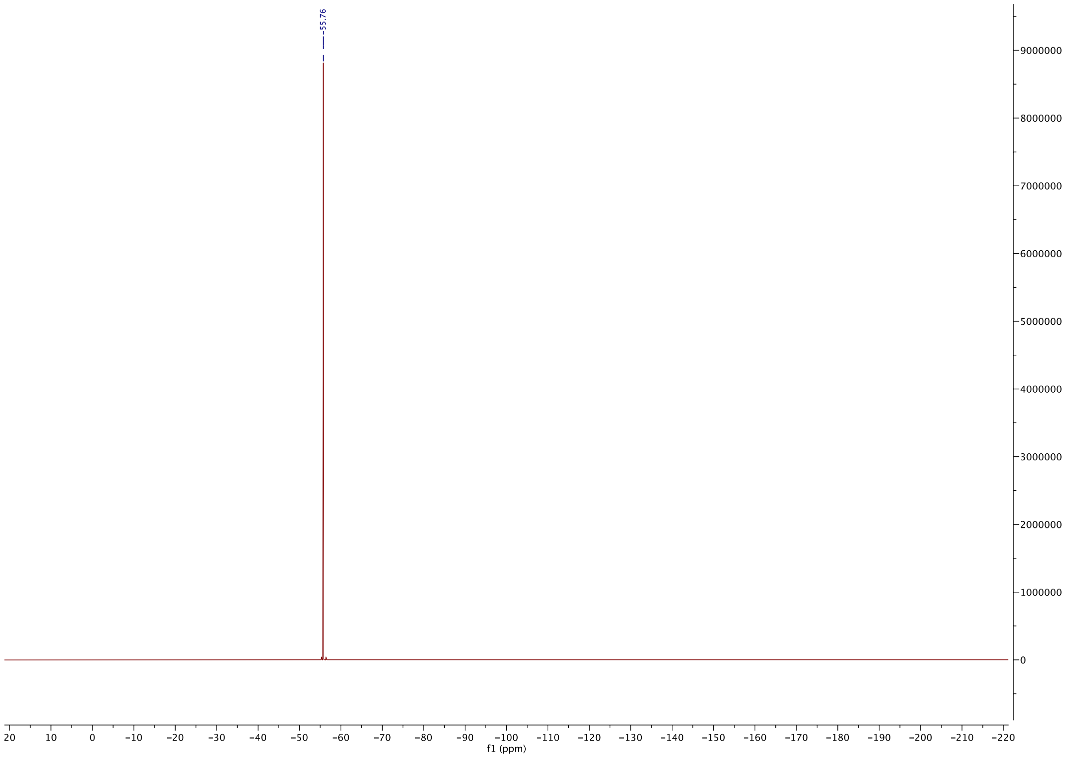

-55.76
9000000
8000000
7000000
6000000
5000000
4000000
3000000
2000000
1000000
0
20 10 0 −10 −20 −30 −40 −50 −60 −70 −80 −90 −100 −110 −120 −130 −140 −150 −160 −170 −180 −190 −200 −210 −220
f1 (ppm)

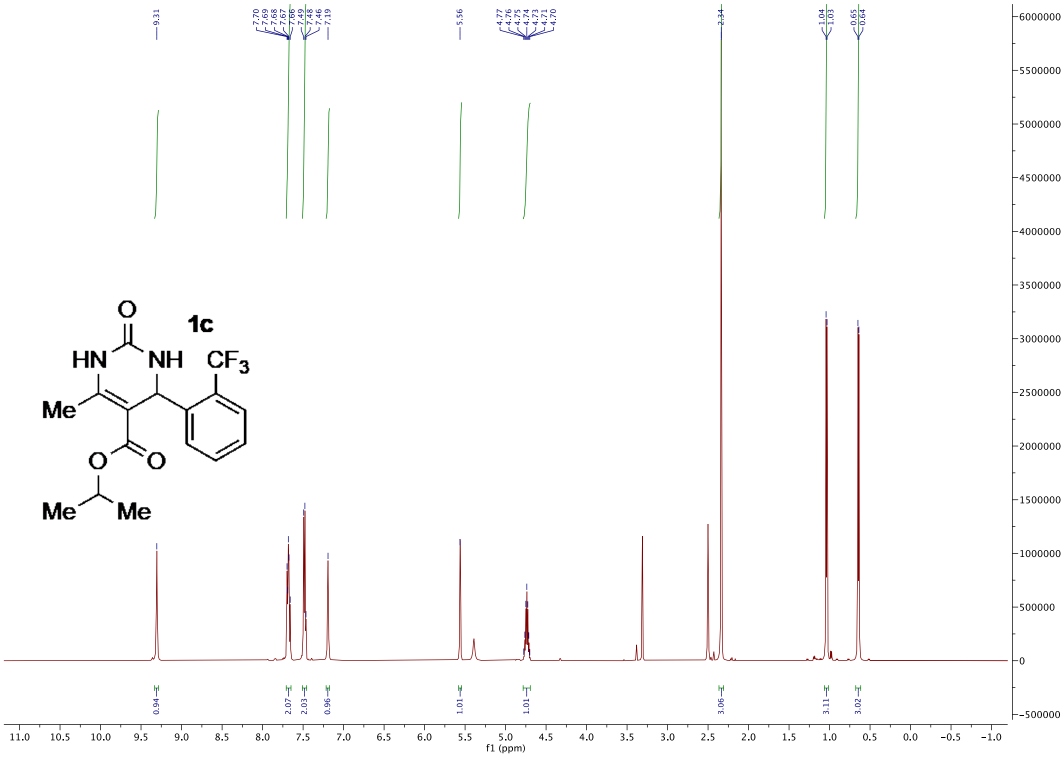
9.31
7.70
7.69
7.68
7.67
7.66
7.49
7.48
7.46
7.19
5.56
4.77
4.76
4.75
4.74
4.73
4.71
4.70
2.34
1.04
1.03
0.65
0.64
1c
0.94
2.07
2.03
0.96
1.01
1.01
3.06
3.11
3.02
f1 (ppm)

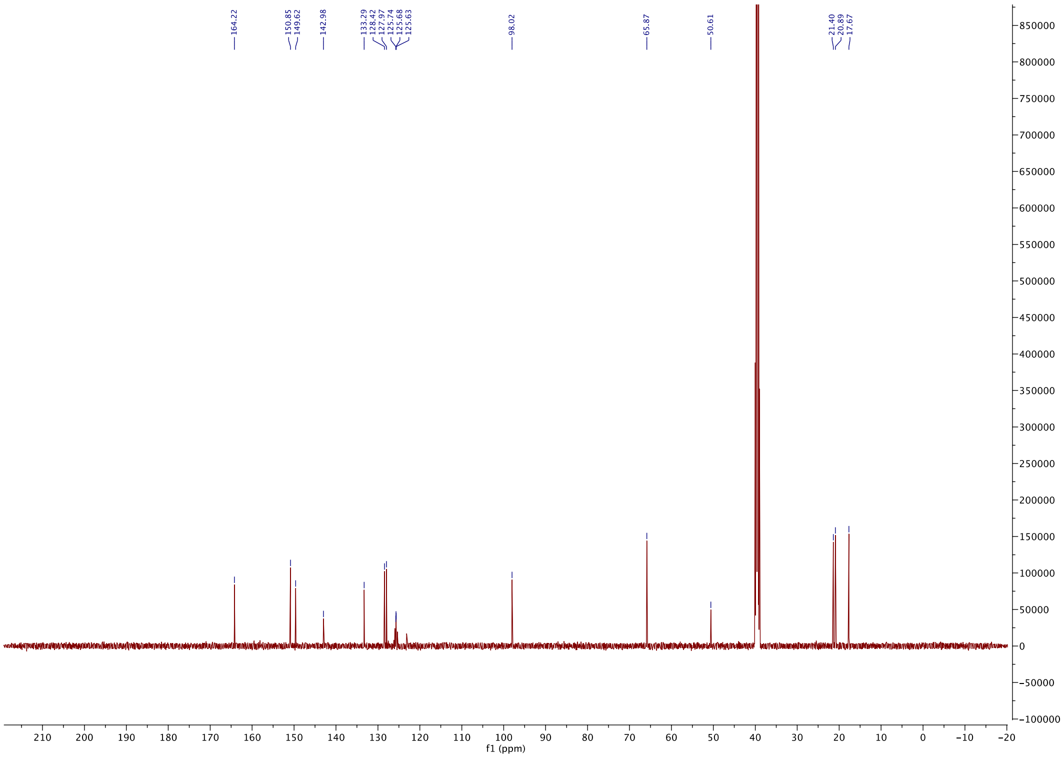
164.22
150.85
149.62
142.98
133.29
128.42
127.97
125.74
125.68
125.63
98.02
65.87
50.61
21.40
20.89
17.67
f1 (ppm)

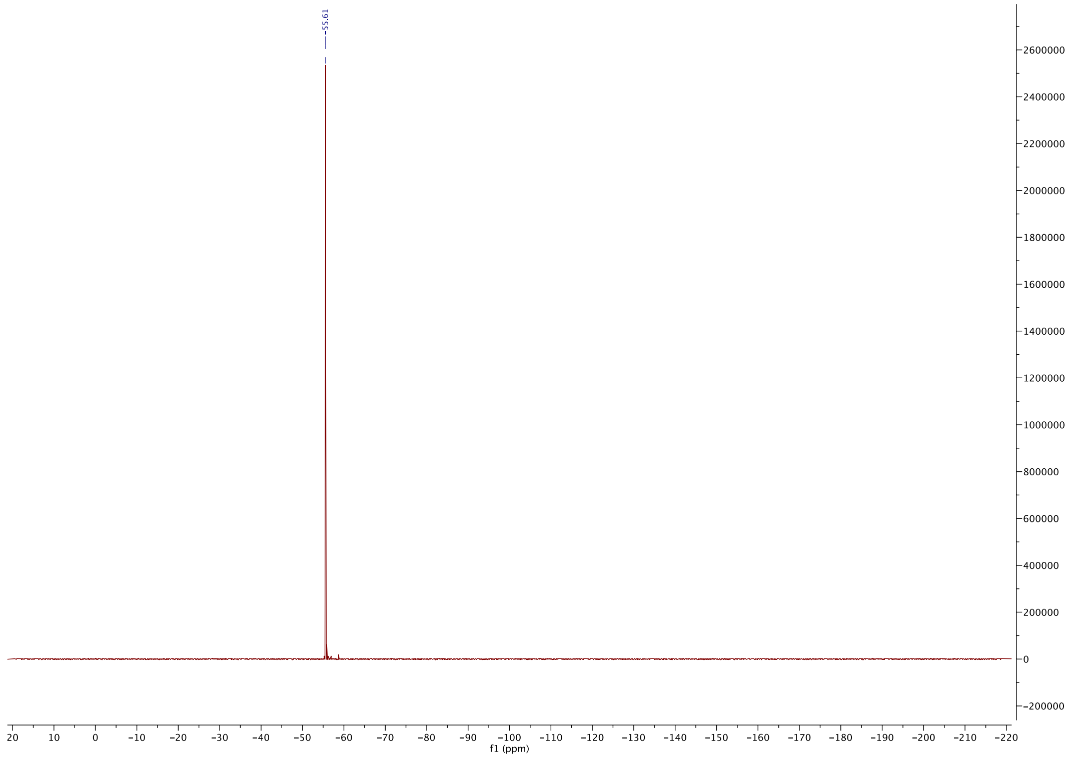

-55.61
2600000
2400000
2200000
2000000
1800000
1600000
1400000
1200000
1000000
800000
600000
400000
200000
0
-200000
20 10 0 -10 -20 -30 -40 -50 -60 -70 -80 -90 -100 -110 -120 -130 -140 -150 -160 -170 -180 -190 -200 -210 -220
f1 (ppm)

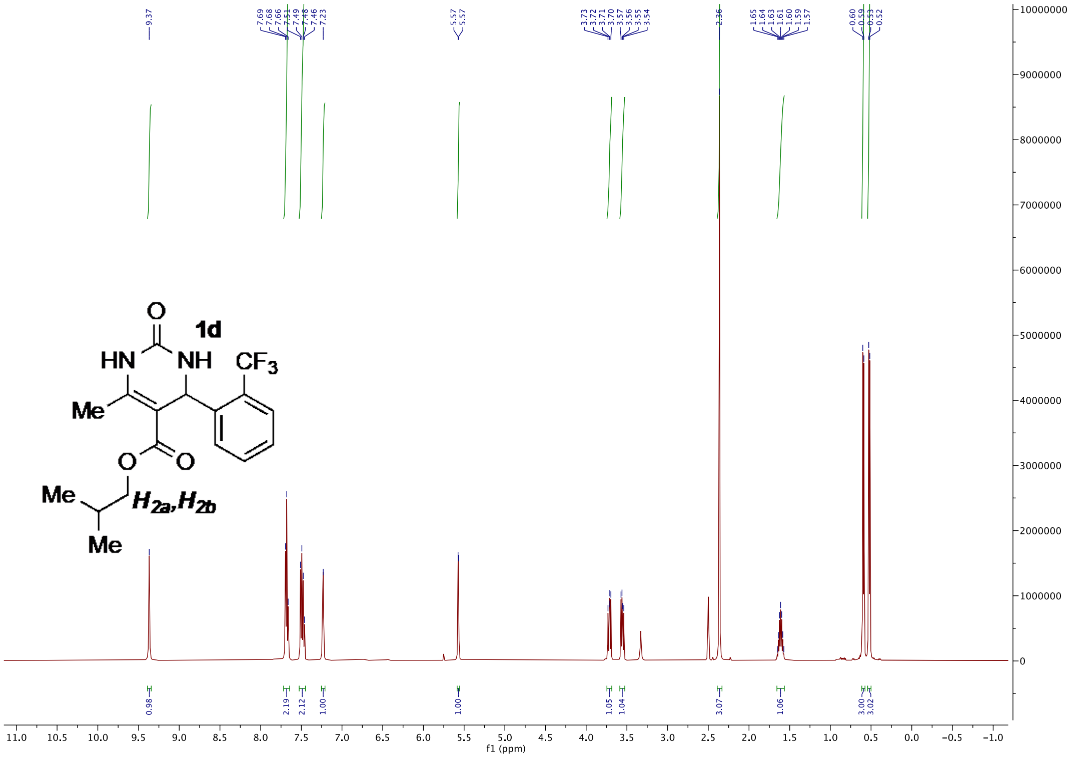
1d
HN
NH
CF3
Me
O
Me
H2a,H2b
Me
f1 (ppm)

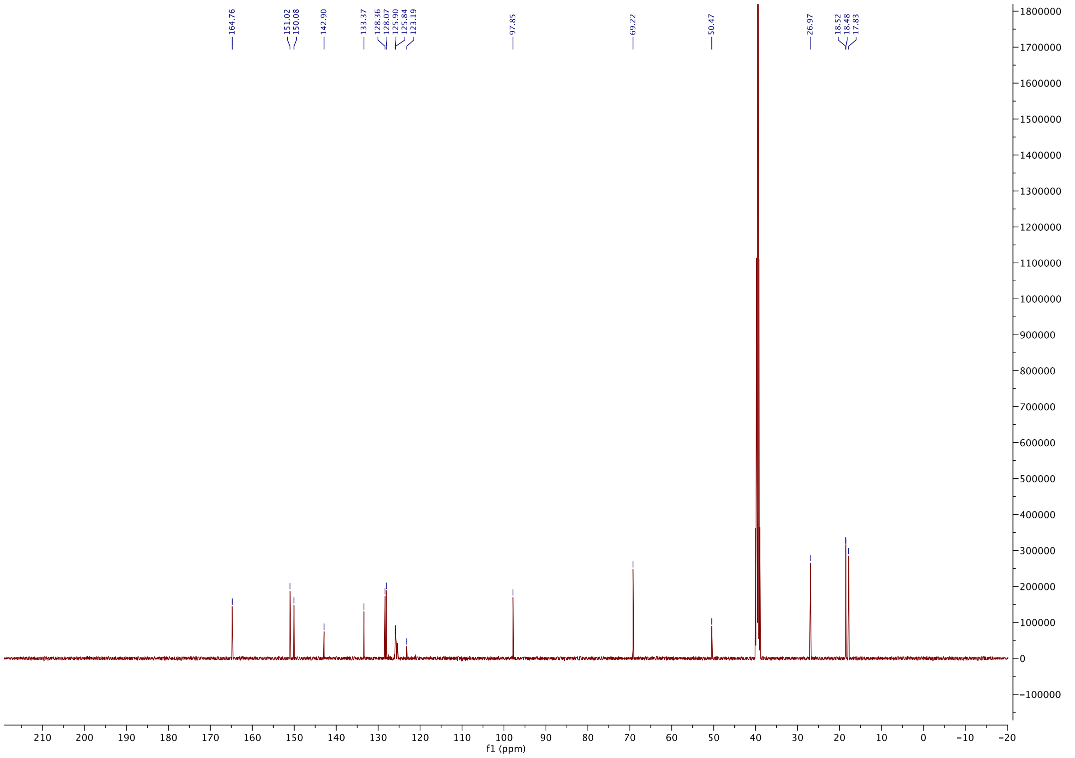
164.76
151.02
150.08
142.90
133.37
128.36
128.07
125.90
125.84
123.19
97.85
69.22
50.47
26.97
18.52
18.48
17.83
f1 (ppm)

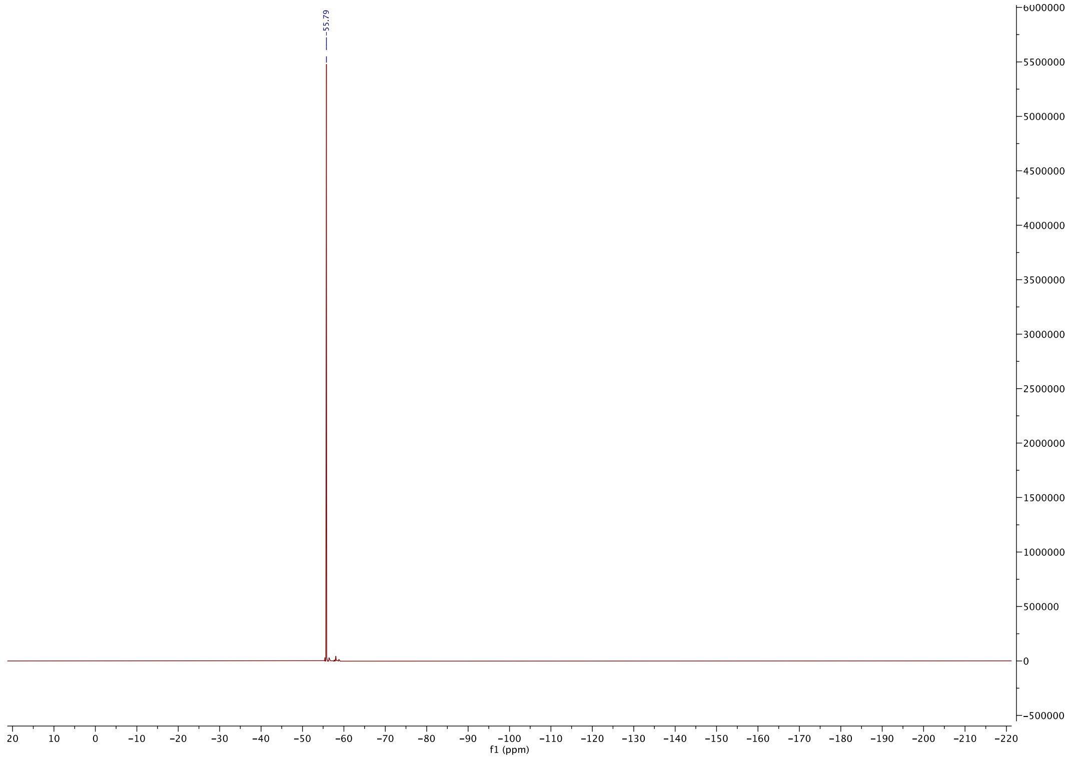
-55.79
f1 (ppm)

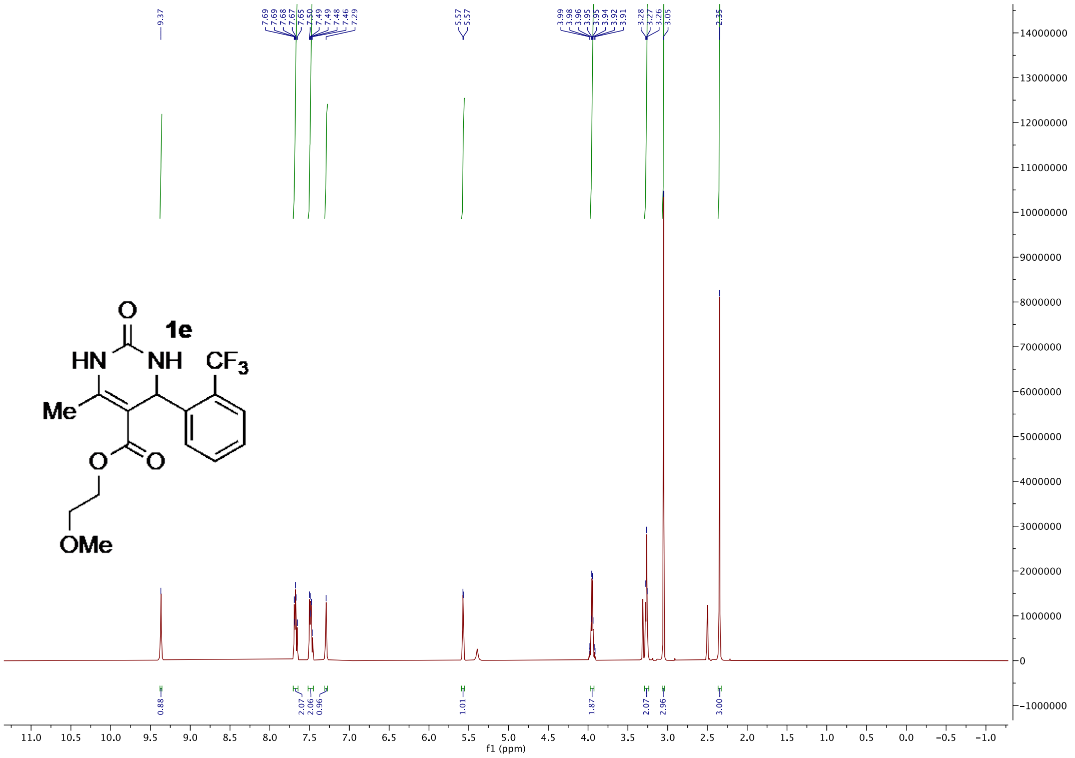
1e
HN
NH
CF3
Me
O
OMe
9.37
7.69 7.69 7.68 7.67 7.65 7.50 7.49 7.49 7.48 7.46 7.29
5.57 5.57
3.99 3.98 3.96 3.95 3.95 3.94 3.92 3.91
3.28 3.27 3.26 3.05
2.35
0.88
2.07 2.06 0.96
1.01
1.87
2.07 2.96
3.00
f1 (ppm)

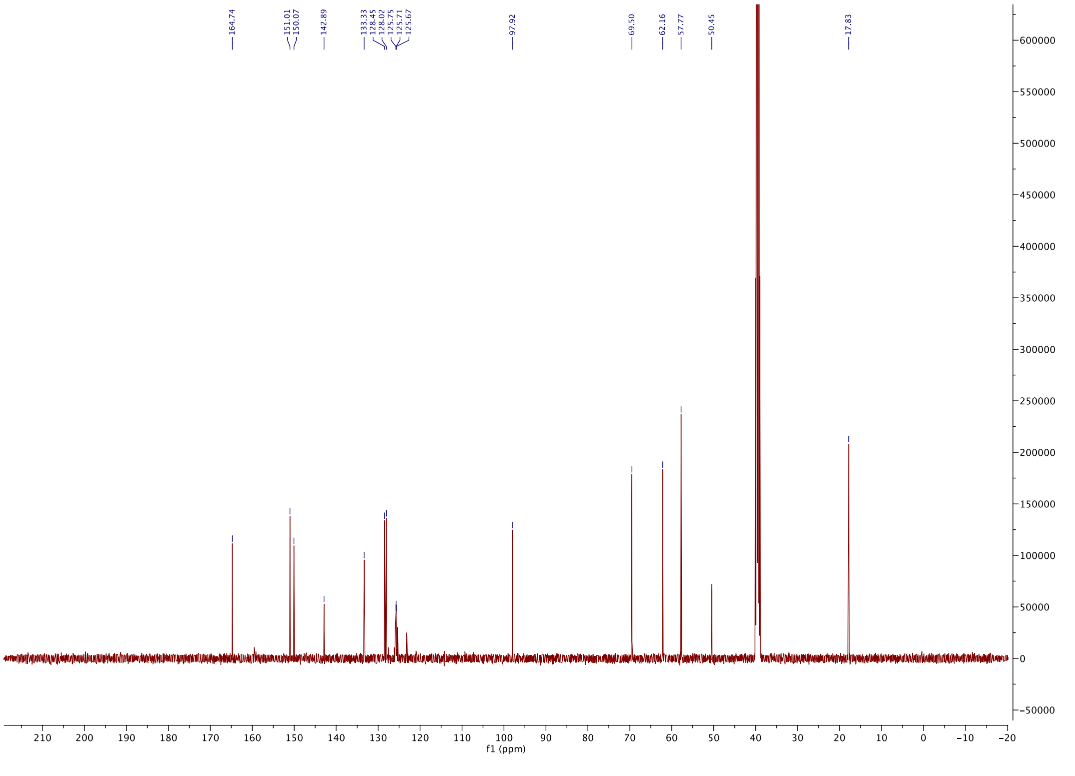
164.74
151.01 150.07
142.89
133.33 128.45 128.02 125.75 125.71 125.67
97.92
69.50
62.16
57.77
50.45
17.83
f1 (ppm)

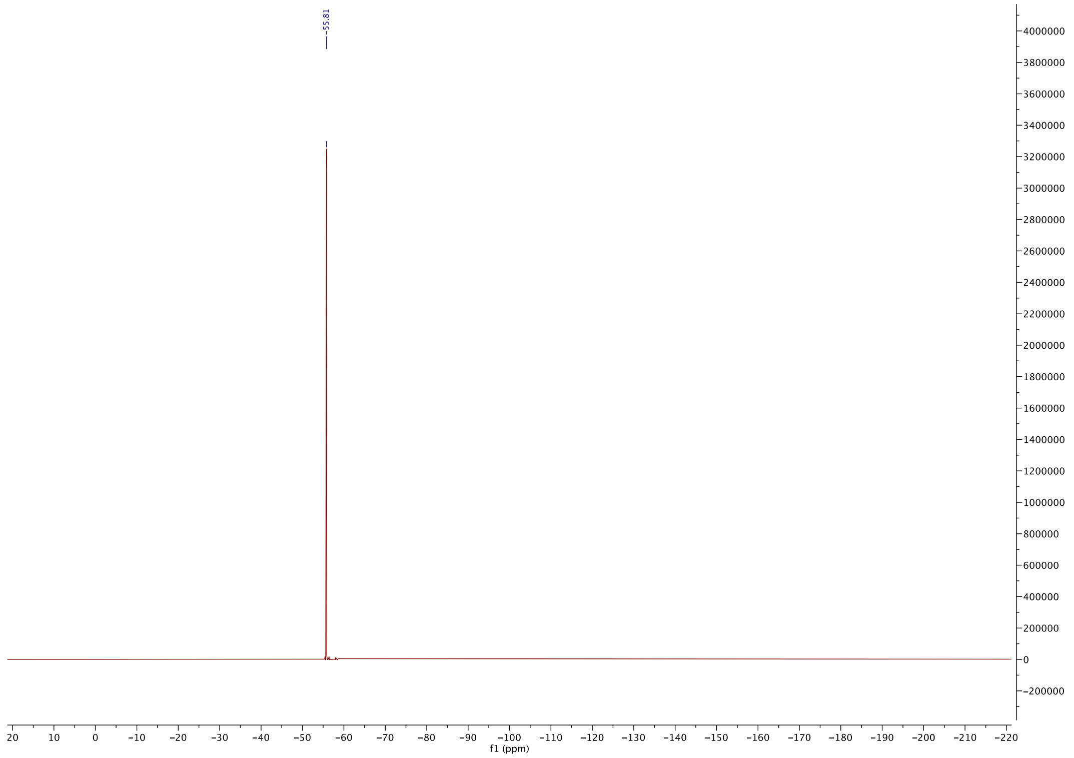

-55.81
4000000
3800000
3600000
3400000
3200000
3000000
2800000
2600000
2400000
2200000
2000000
1800000
1600000
1400000
1200000
1000000
800000
600000
400000
200000
0
-200000
20 10 0 -10 -20 -30 -40 -50 -60 -70 -80 -90 -100 -110 -120 -130 -140 -150 -160 -170 -180 -190 -200 -210 -220
f1 (ppm)

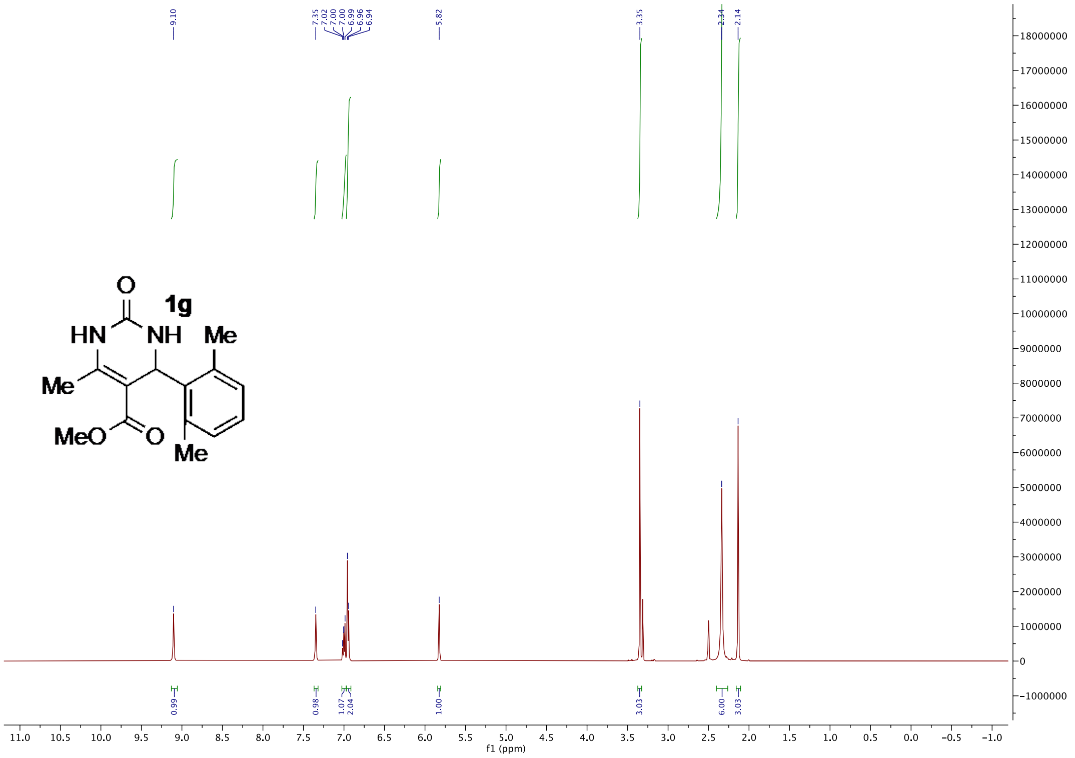
1g
9.10
7.35
7.02
7.00
7.00
6.99
6.96
6.94
5.82
3.35
2.34
2.14
0.99
0.98
1.07
2.04
1.00
3.03
6.00
3.03
f1 (ppm)

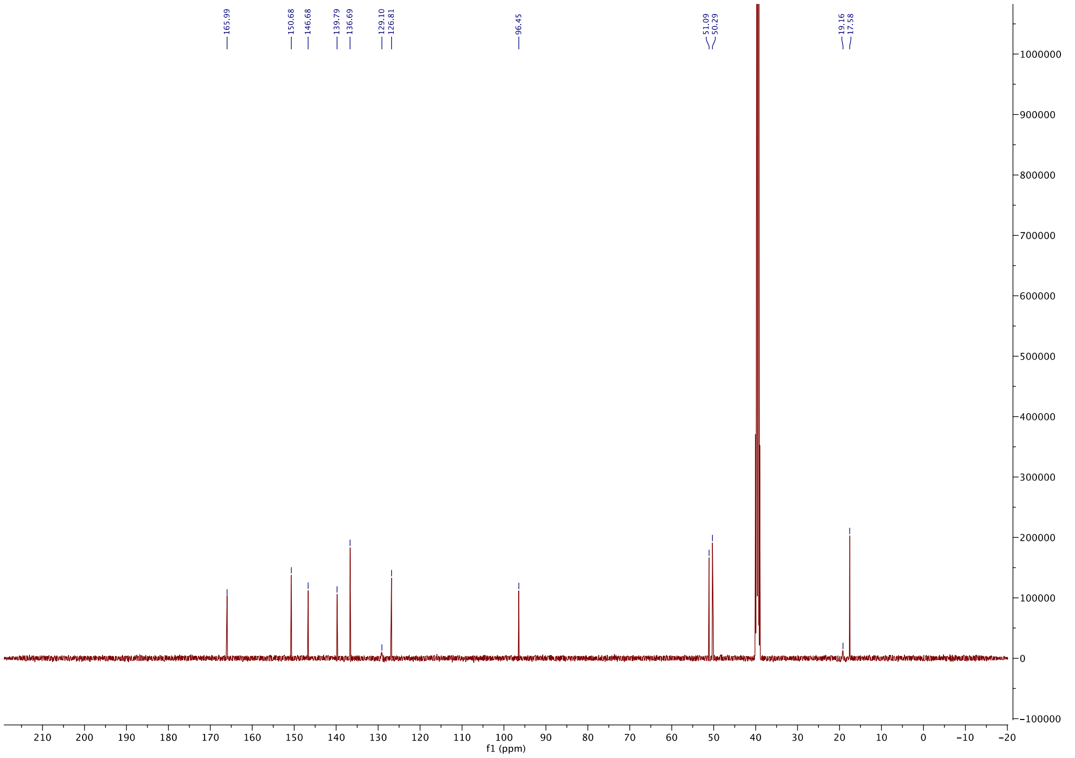
165.99
150.68
146.68
139.79
136.69
129.10
126.81
96.45
51.09
50.29
19.16
17.58
f1 (ppm)

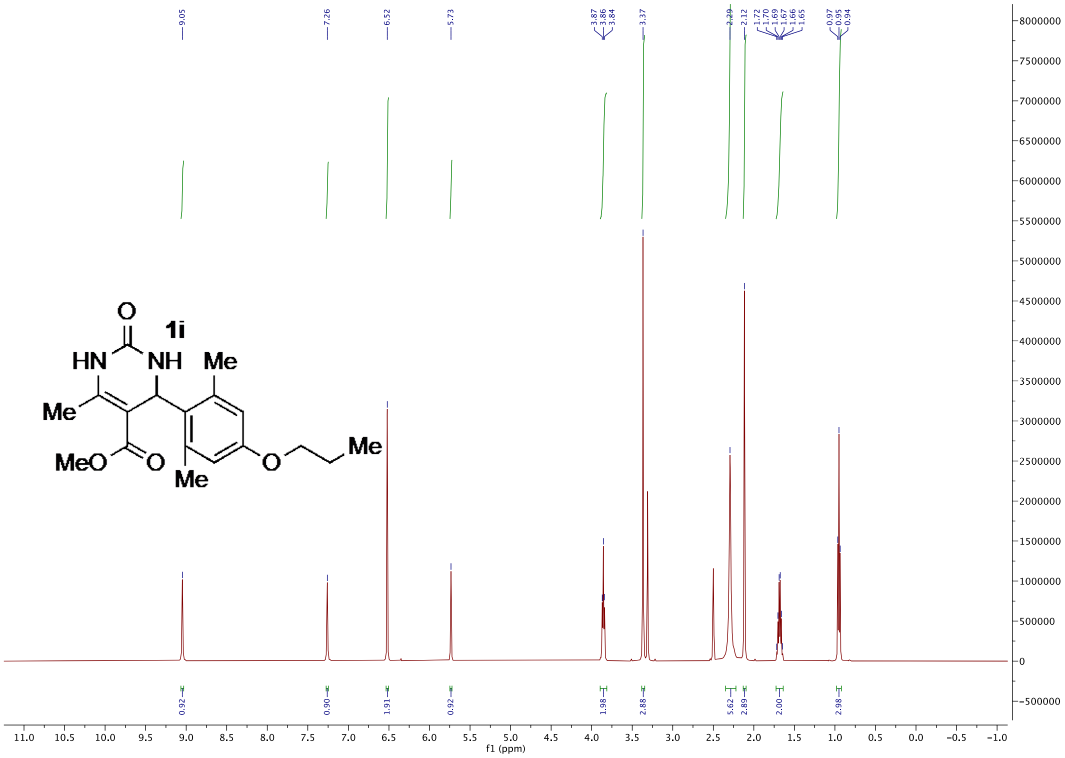
1i
O
HN
NH
Me
MeO
O
Me
O
Me
Me
9.05
7.26
6.52
5.73
3.87
3.86
3.84
3.37
2.29
2.12
1.72
1.70
1.69
1.67
1.66
1.65
0.97
0.95
0.94
0.92
0.90
1.91
0.92
1.98
2.88
5.62
2.89
2.00
2.98
f1 (ppm)

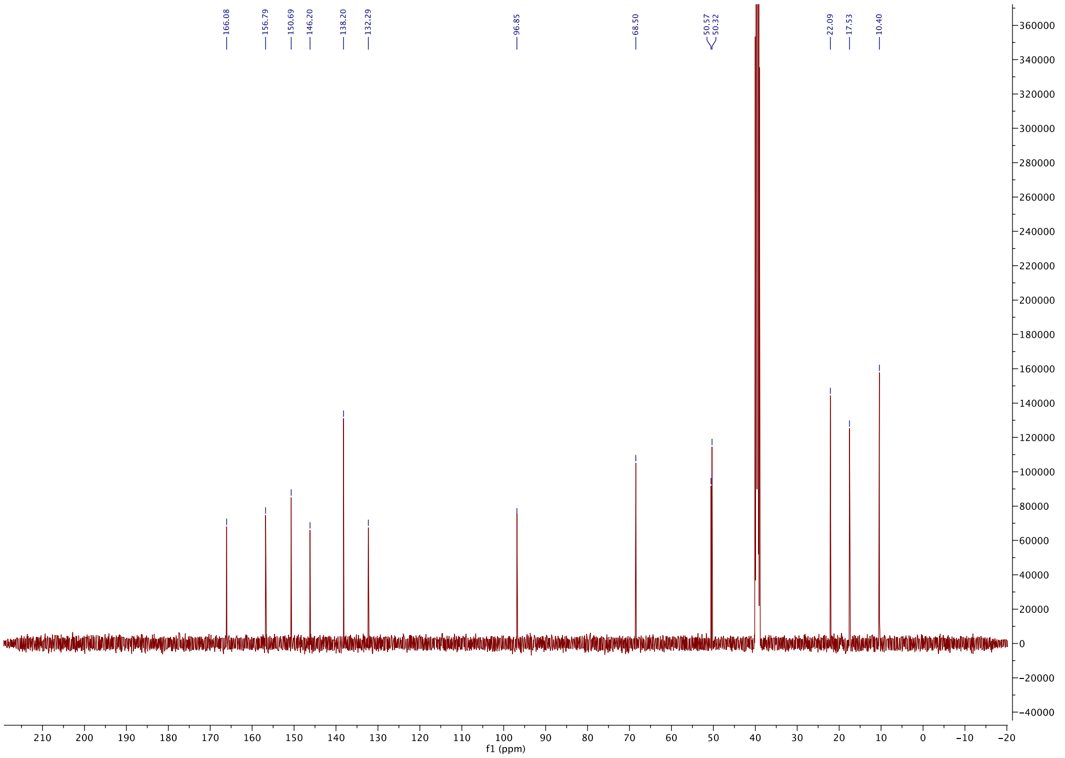
166.08
156.79
150.69
146.20
138.20
132.29
96.85
68.50
50.57
50.32
22.09
17.53
10.40
f1 (ppm)

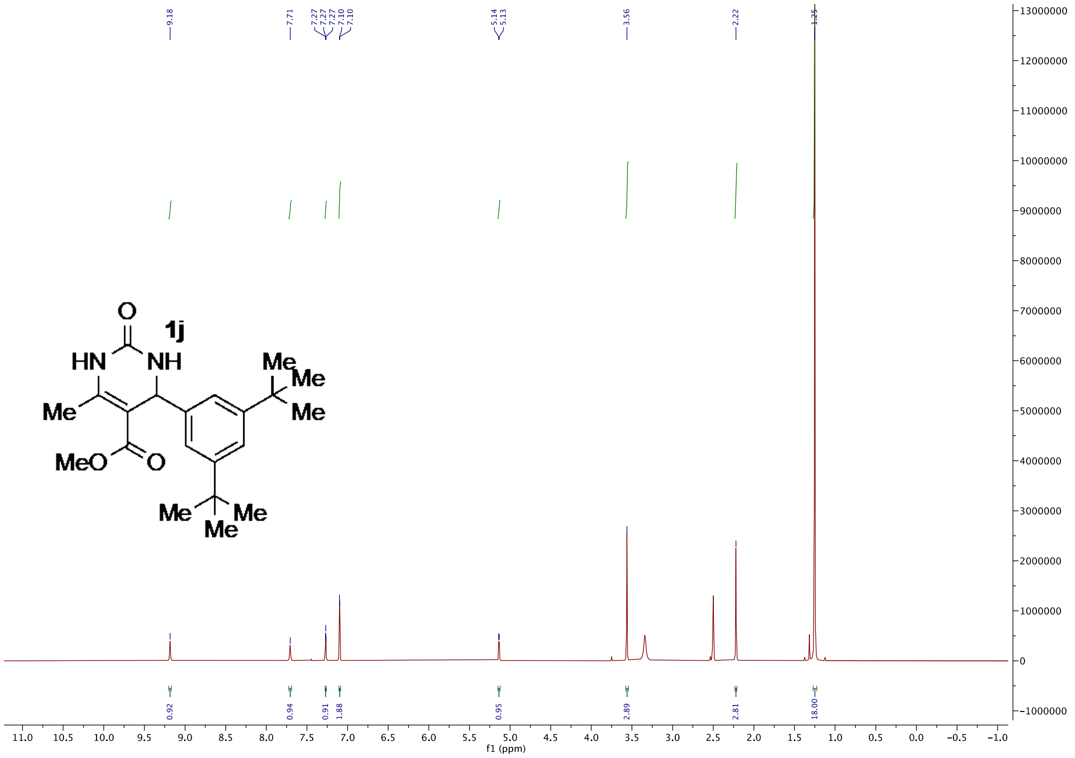
1j
f1 (ppm)

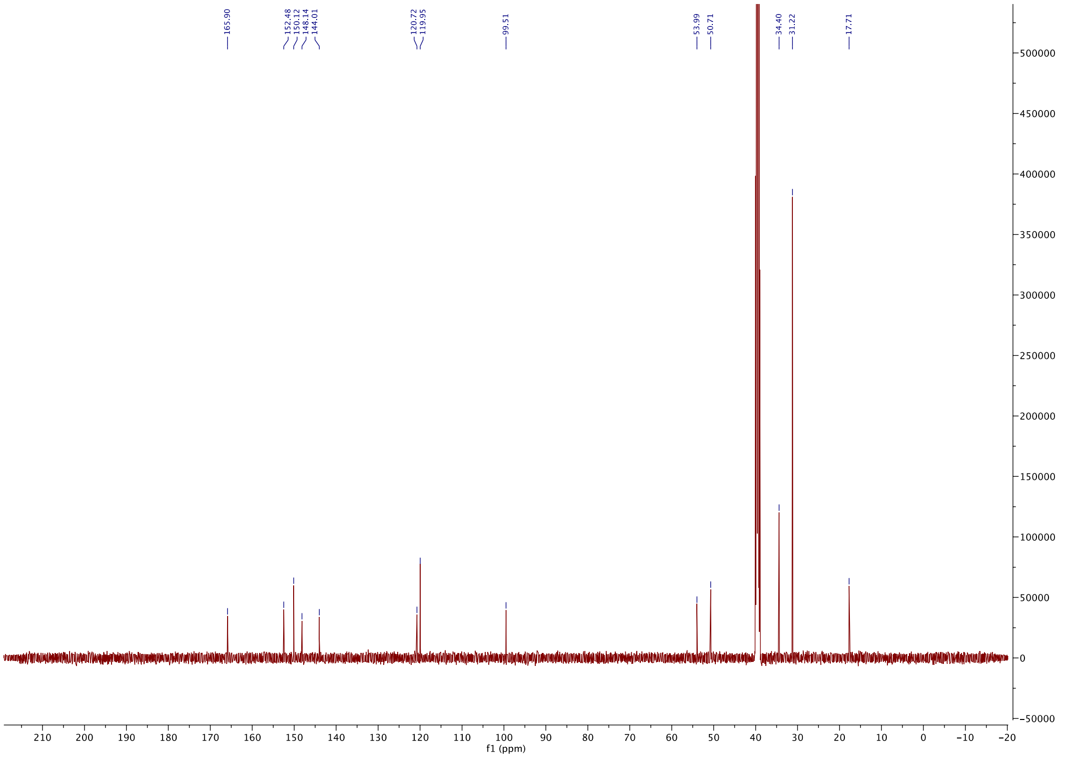
f1 (ppm)

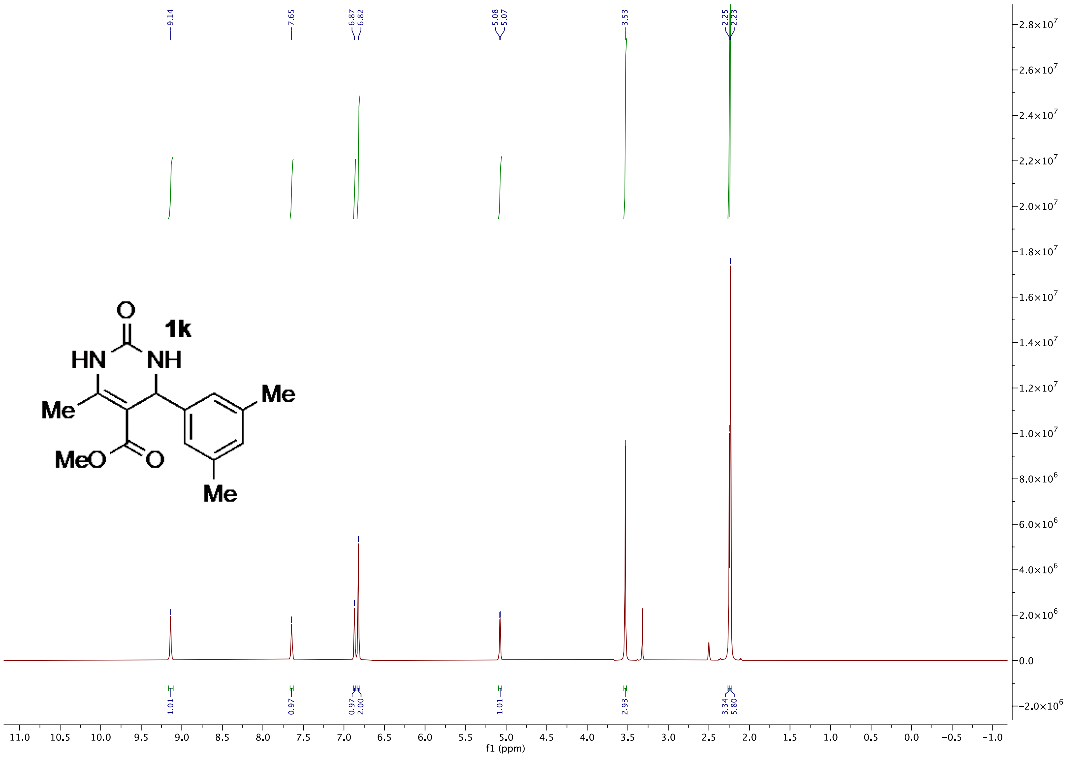
1k

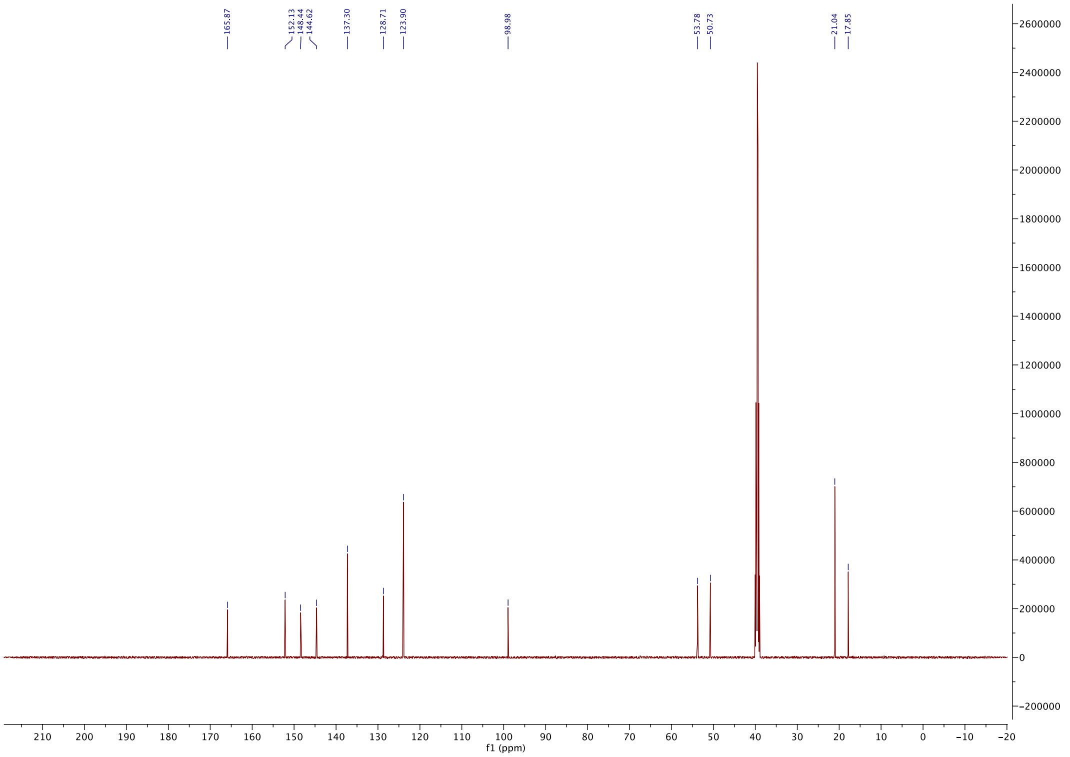

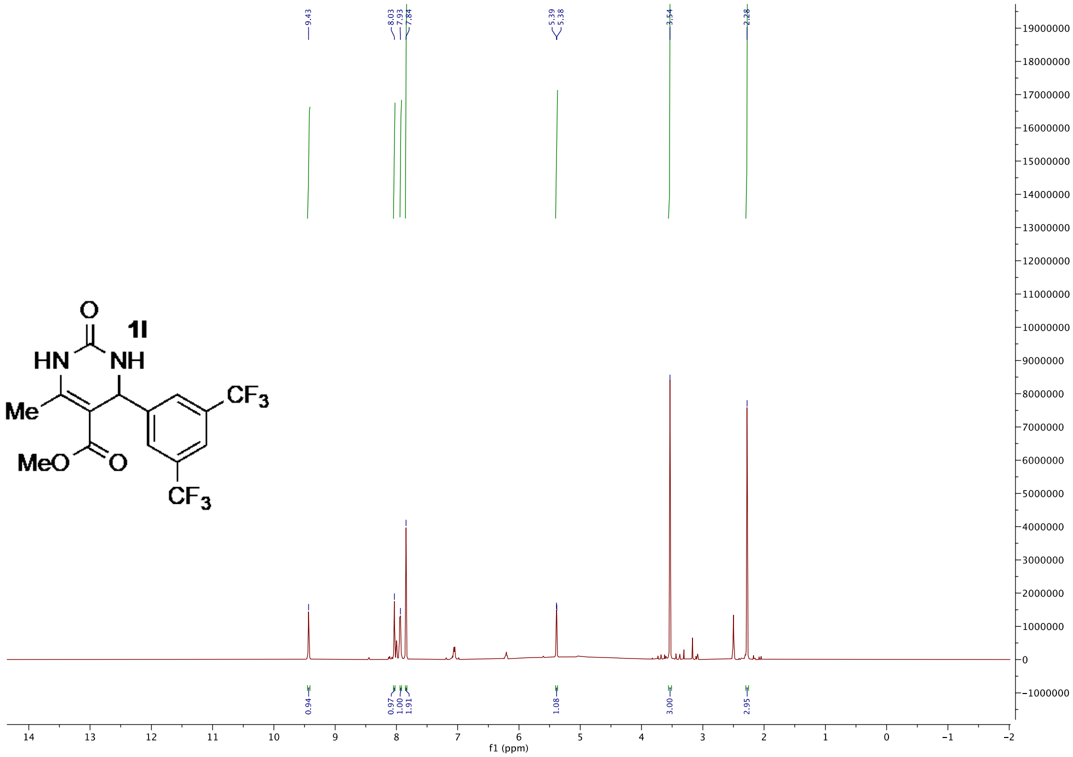
1l

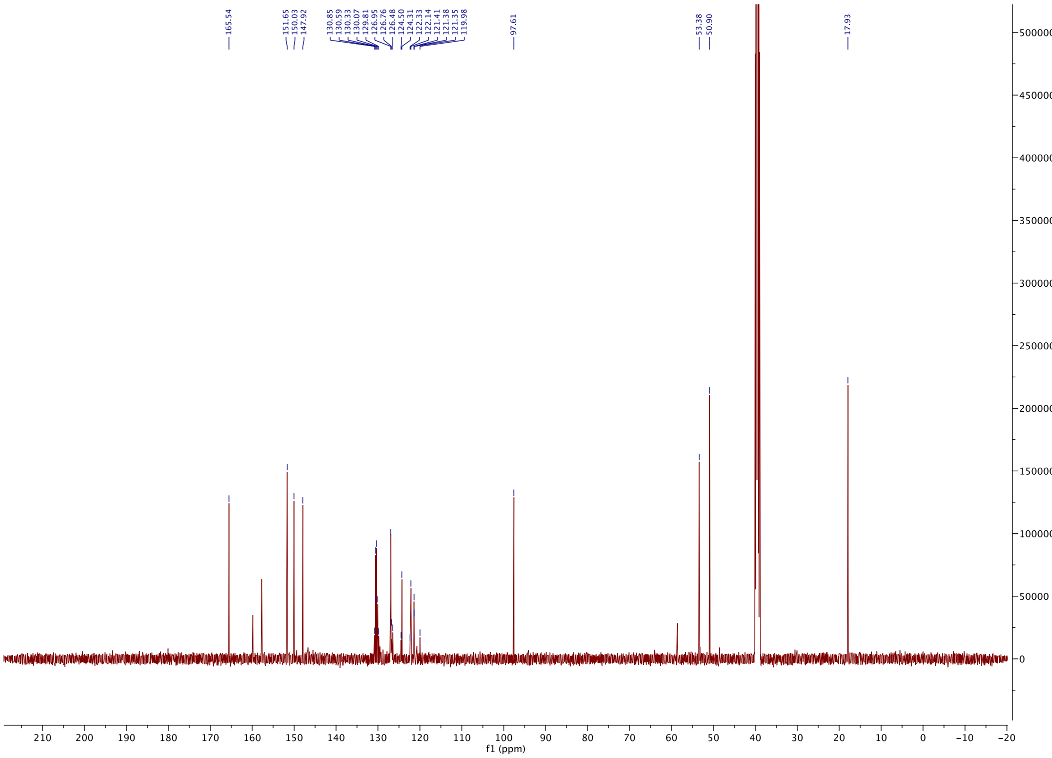

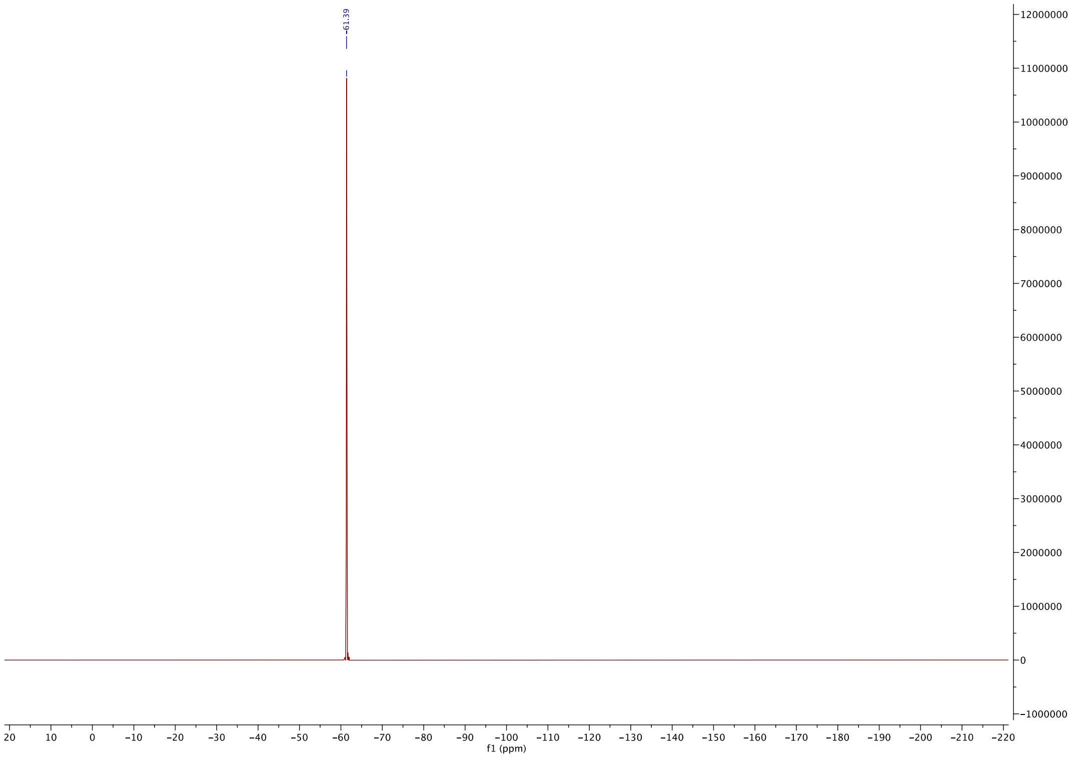
-61.39
f1 (ppm)

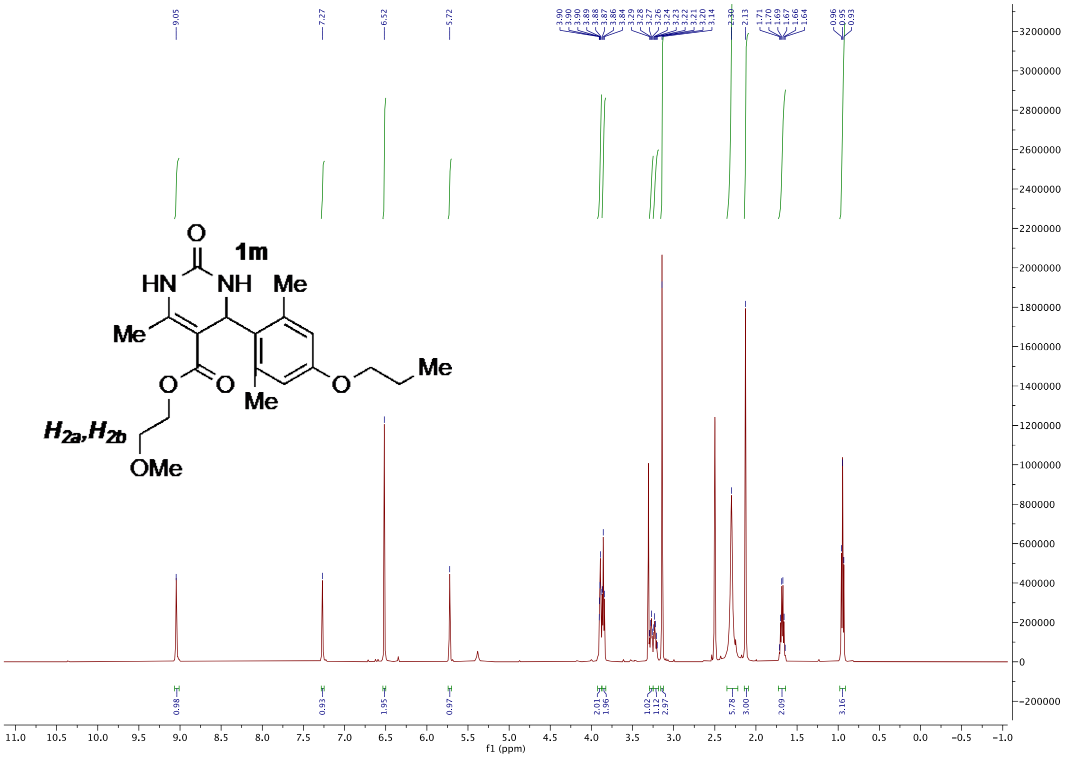
1m
HN
NH
Me
Me
O
Me
Me
OMe
H2a,H2b
f1 (ppm)

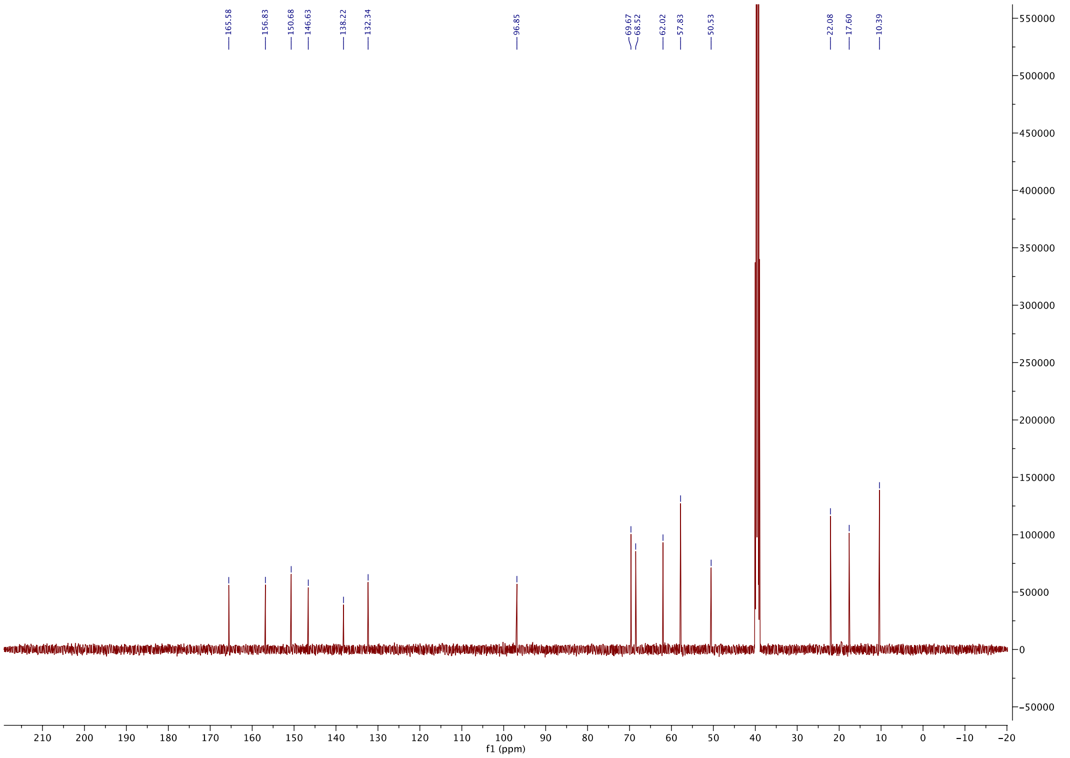
165.58
156.83
150.68
146.63
138.22
132.34
96.85
69.67
68.52
62.02
57.83
50.53
22.08
17.60
10.39
f1 (ppm)

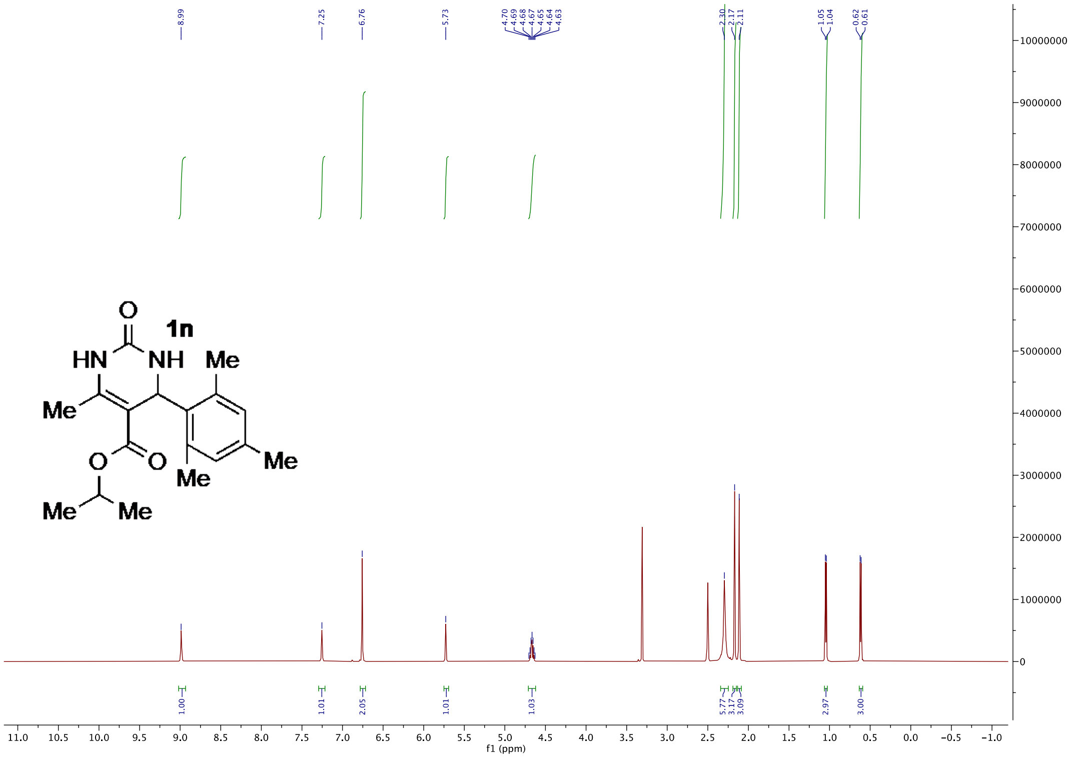
8.99
7.25
6.76
5.73
4.70
4.69
4.68
4.67
4.65
4.64
4.63
2.30
2.17
2.11
1.05
1.04
0.62
0.61
1n
HN
NH
Me
Me
O
O
O
Me
Me
Me
Me
1.00
1.01
2.05
1.01
1.03
5.72
3.17
3.09
2.97
3.00
f1 (ppm)

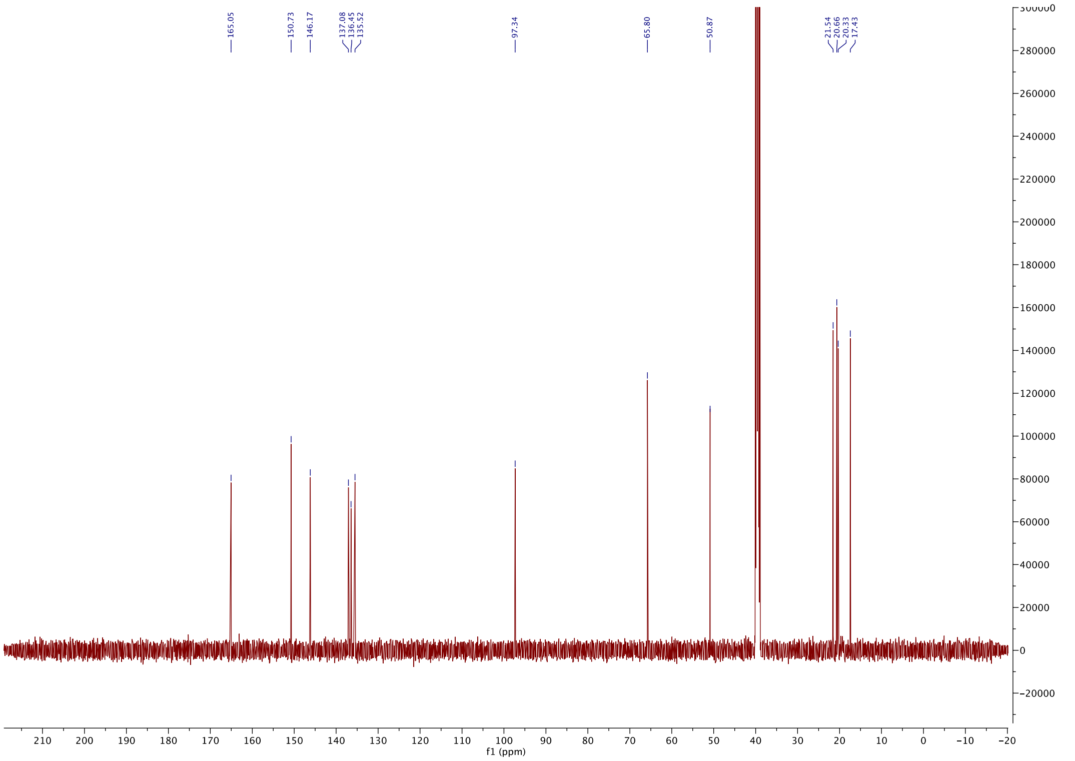
165.05
150.73
146.17
137.08
136.45
135.52
97.34
65.80
50.87
21.54
20.66
20.33
17.43
f1 (ppm)

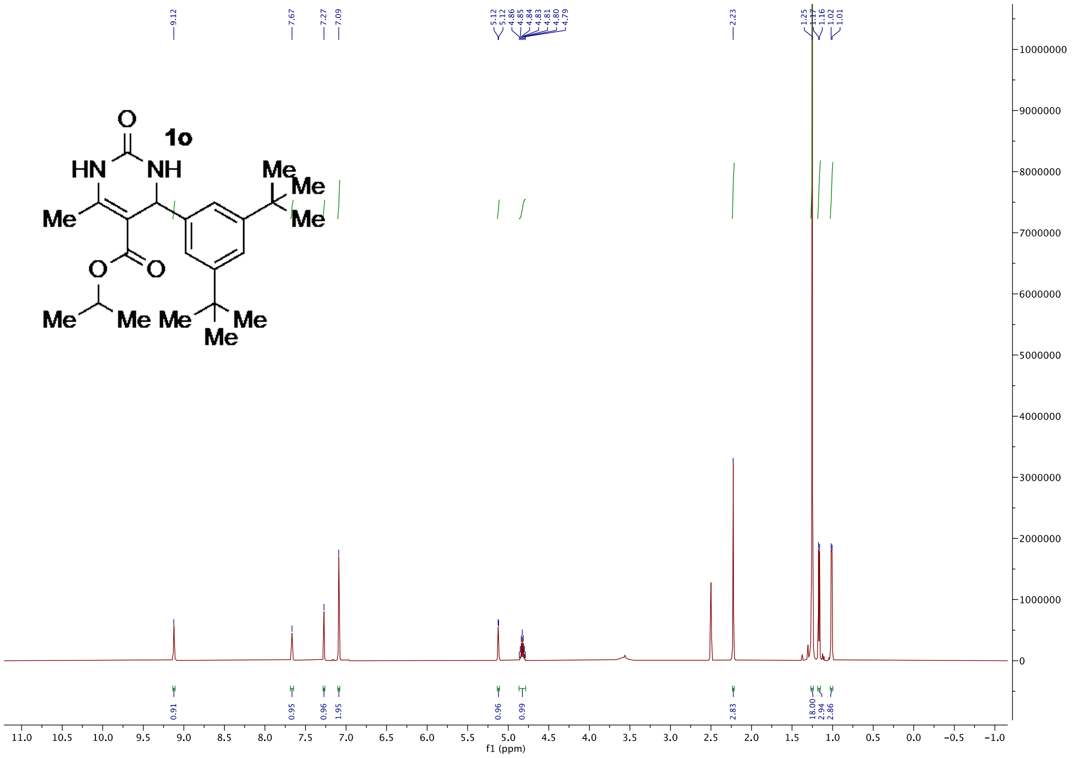
1o
HN NH O Me O O Me Me Me Me Me Me Me Me
9.12
7.67
7.27
7.09
5.12
5.12
4.86
4.85
4.84
4.83
4.81
4.80
4.79
2.23
1.25
1.17
1.16
1.02
1.01
0.91
0.95
0.96
1.95
0.96
0.99
2.83
18.00
2.94
2.86
f1 (ppm)

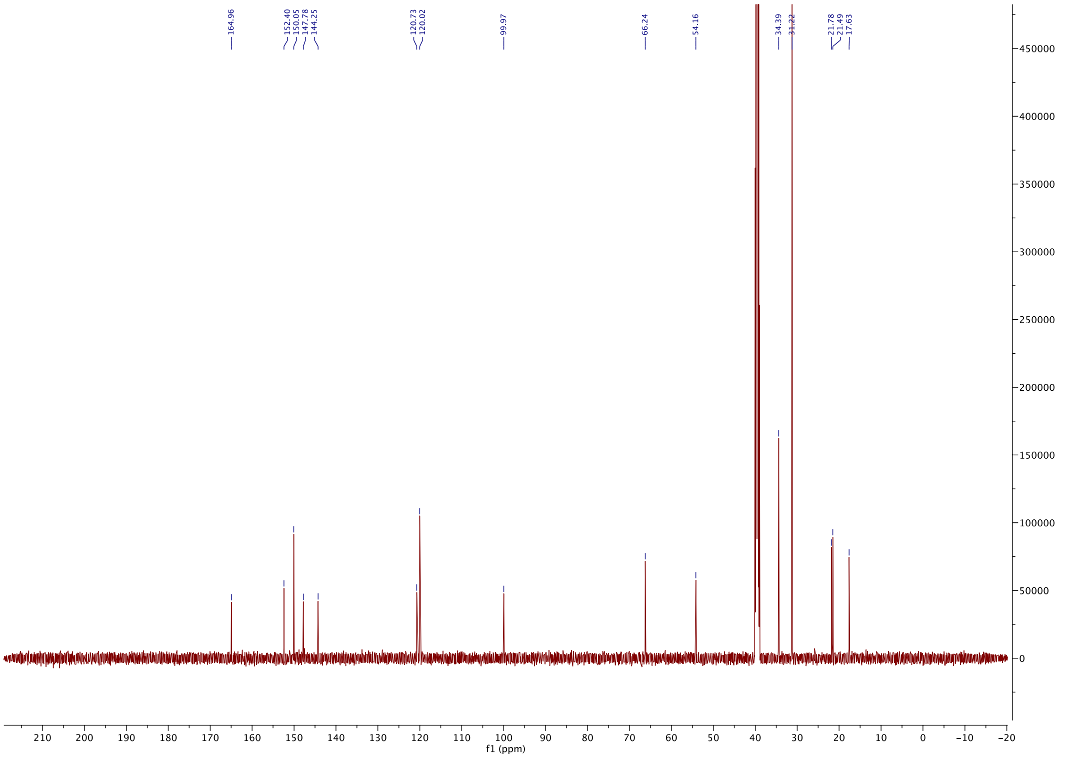
164.96
152.40
150.05
147.78
144.25
120.73
120.02
99.97
66.24
54.16
34.39
31.22
21.78
21.49
17.83
f1 (ppm)

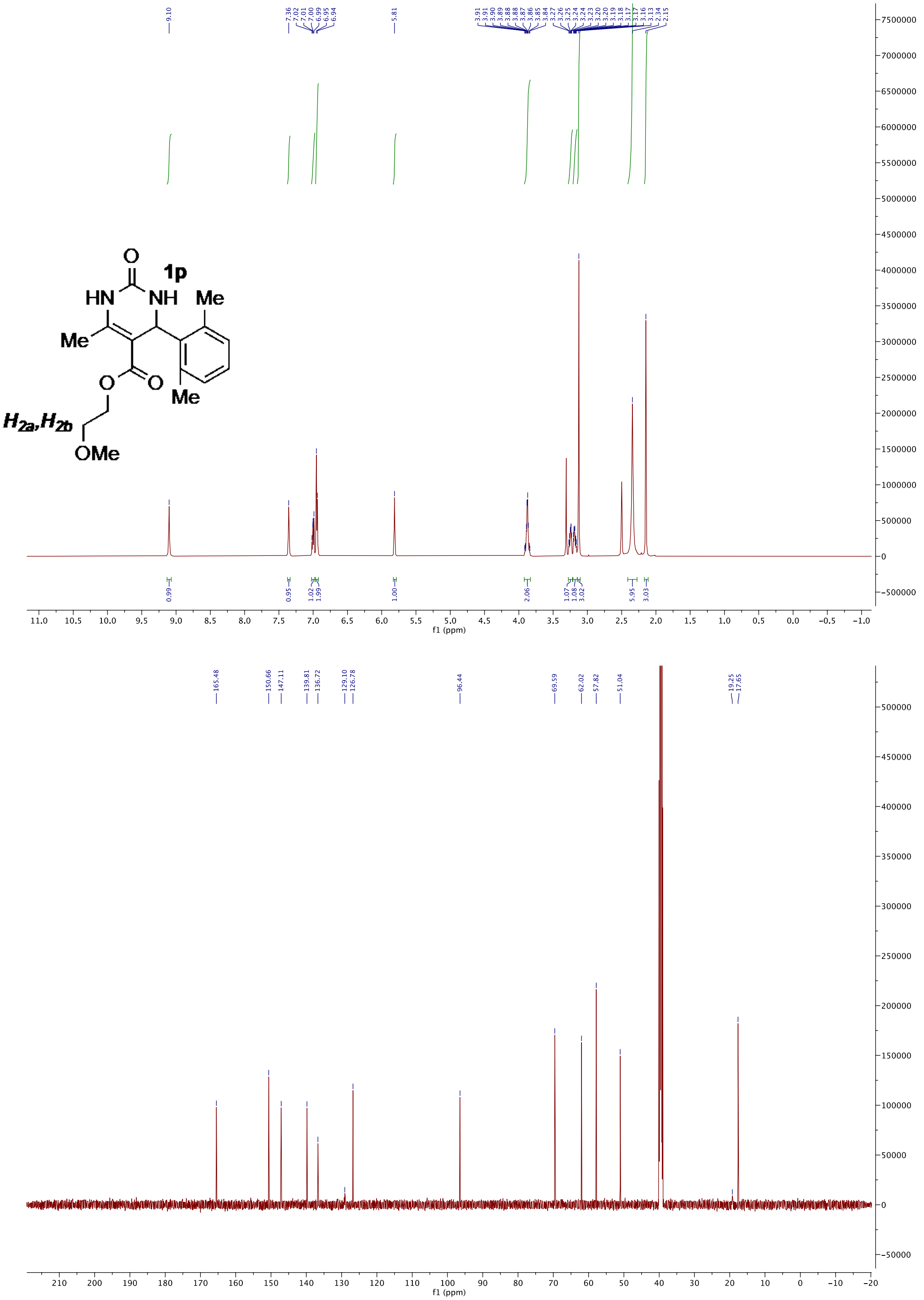
1p
HN
NH
Me
Me
Me
O
O
O
H_{2a},H_{2b}
OMe
f1 (ppm)
f1 (ppm)

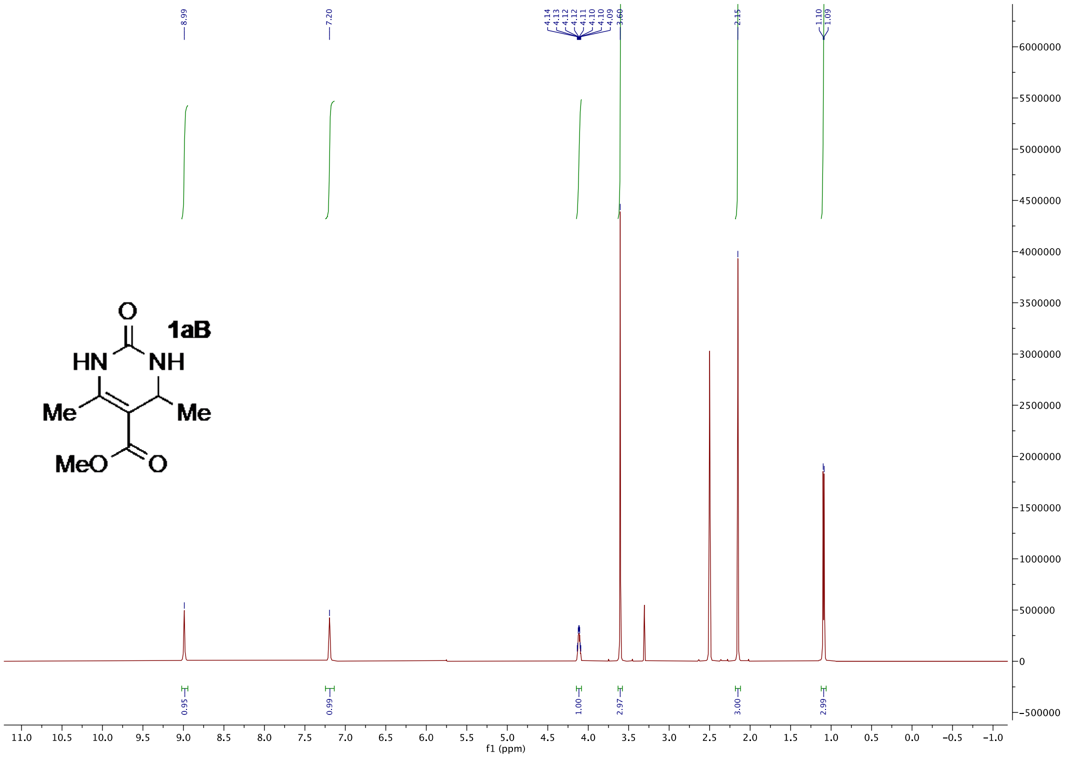
8.99
7.20
4.14
4.13
4.12
4.12
4.11
4.10
4.10
4.09
3.60
2.15
1.10
1.09
1aB
O
HN
NH
Me
Me
MeO
O
0.95
0.99
1.00
2.97
3.00
2.99
f1 (ppm)

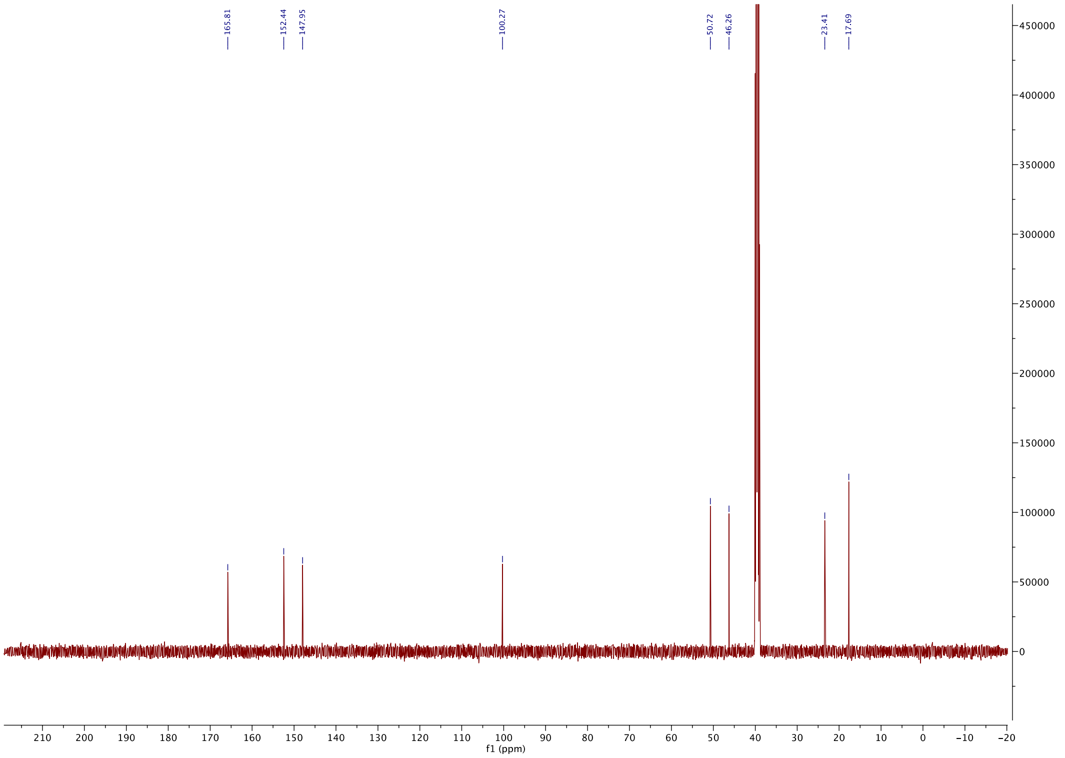
165.81
152.44
147.95
100.27
50.72
46.26
23.41
17.69
f1 (ppm)

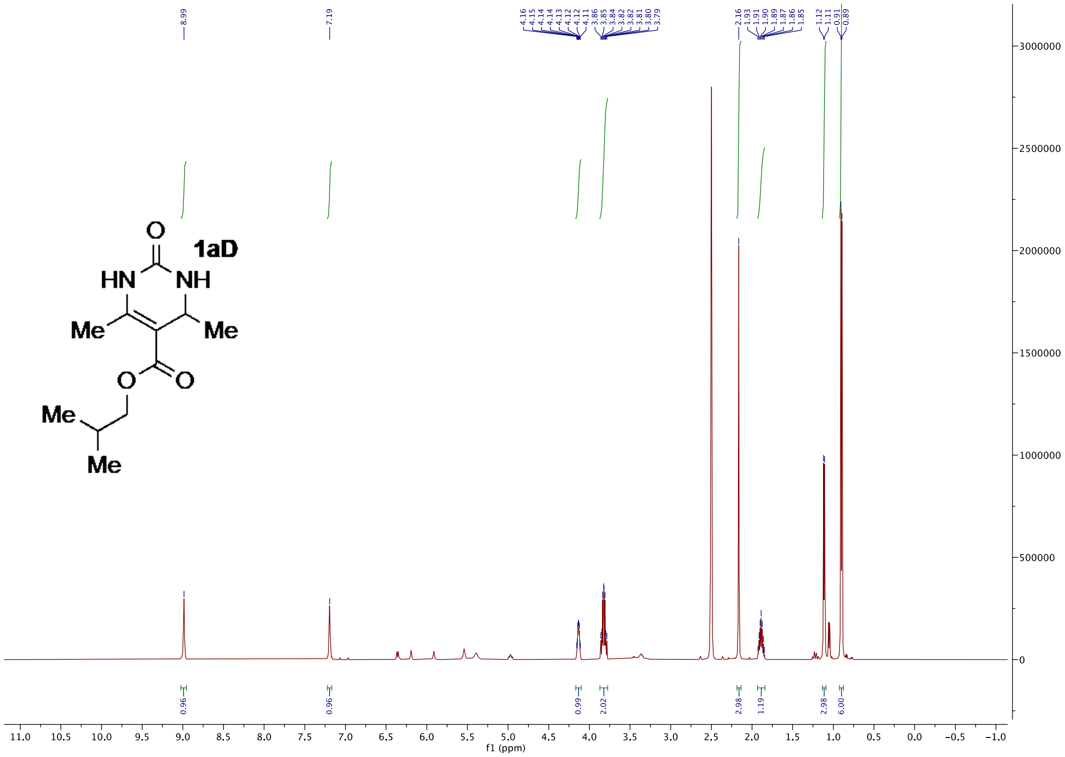

1aD
HN
NH
Me
Me
Me
Me
f1 (ppm)

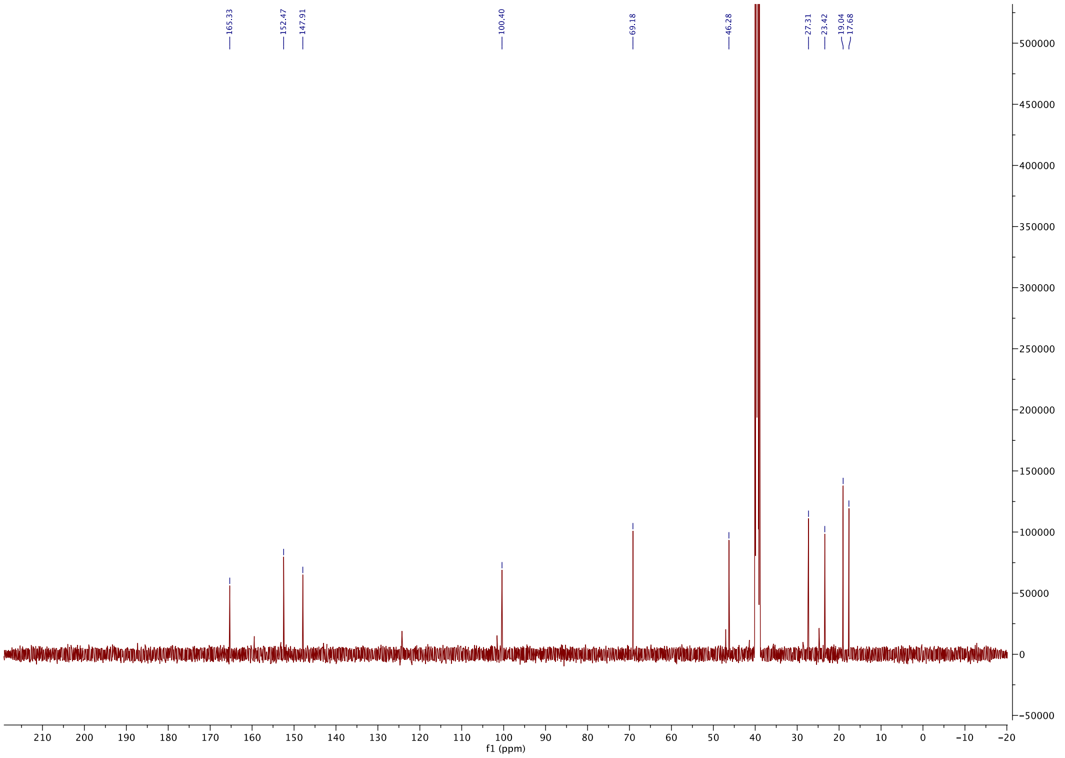

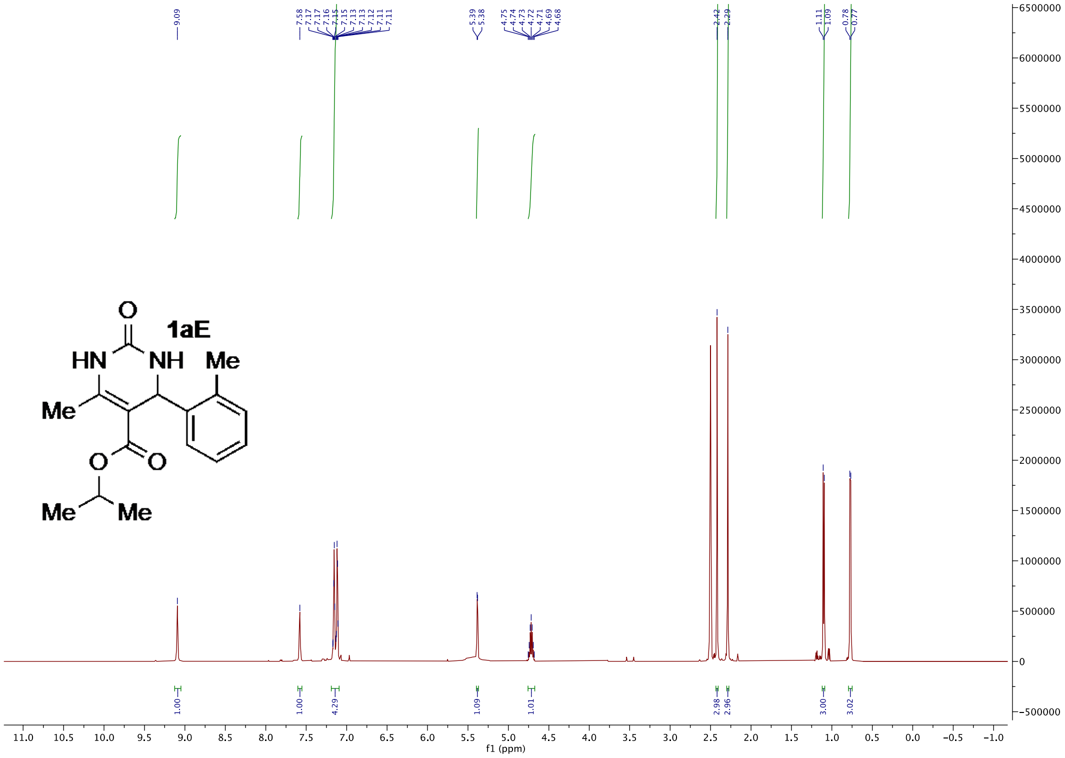
1aE
O
HN
NH
Me
Me
O
O
Me
Me
9.09
7.58 7.17 7.17 7.16 7.15 7.15 7.13 7.13 7.12 7.11 7.11
5.39 5.38
4.75 4.74 4.73 4.72 4.71 4.69 4.68
2.42 2.29
1.11 1.09
0.78 0.77
1.00 1.00 4.29 1.09 1.01 2.98 2.96 3.00 3.02
f1 (ppm)

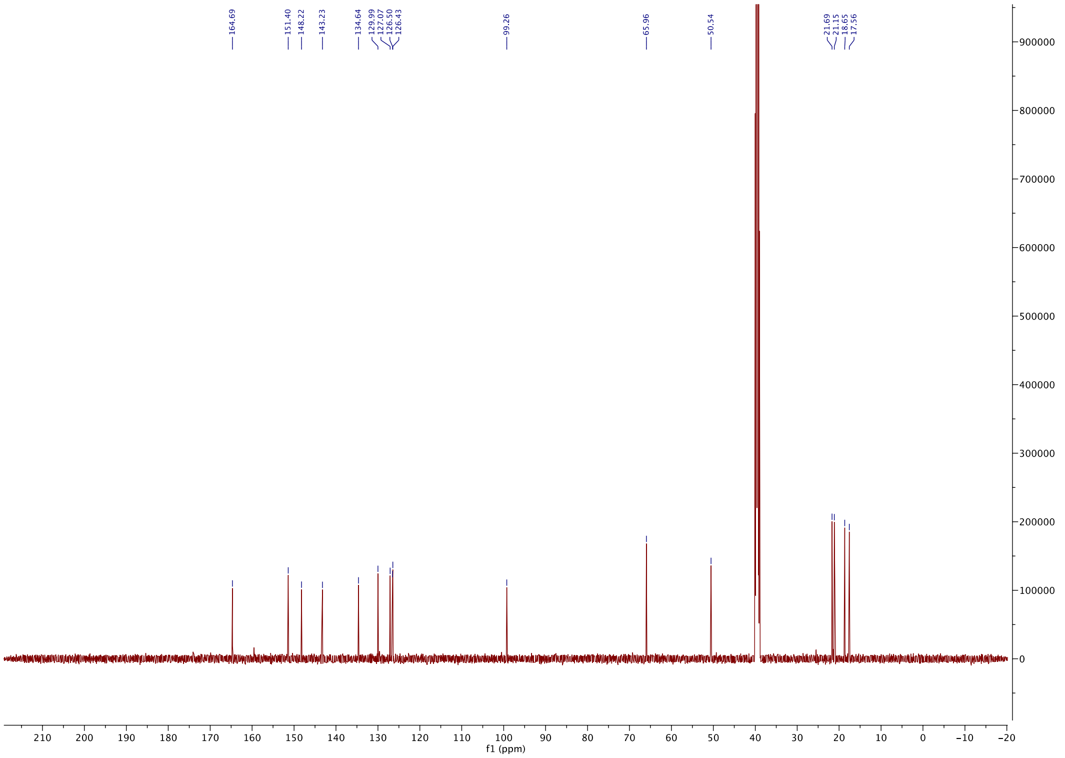
164.69
151.40
148.22
143.23
134.64 129.99 127.07 126.50 126.43
99.26
65.96
50.54
21.69 21.15 18.65 17.56
f1 (ppm)

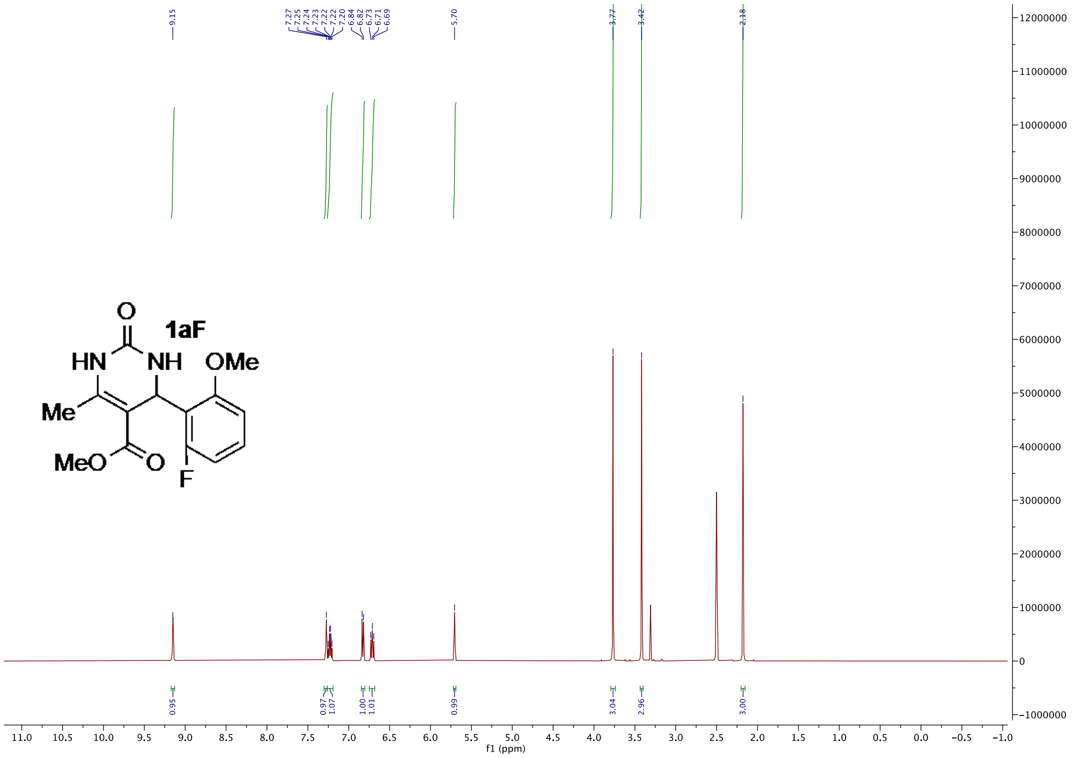
9.15
7.27
7.25
7.24
7.23
7.22
7.22
7.20
6.84
6.82
6.73
6.71
6.69
5.70
3.77
3.42
2.18
1aF
0.95
0.97
1.07
1.00
1.01
0.99
3.04
2.96
3.00
f1 (ppm)

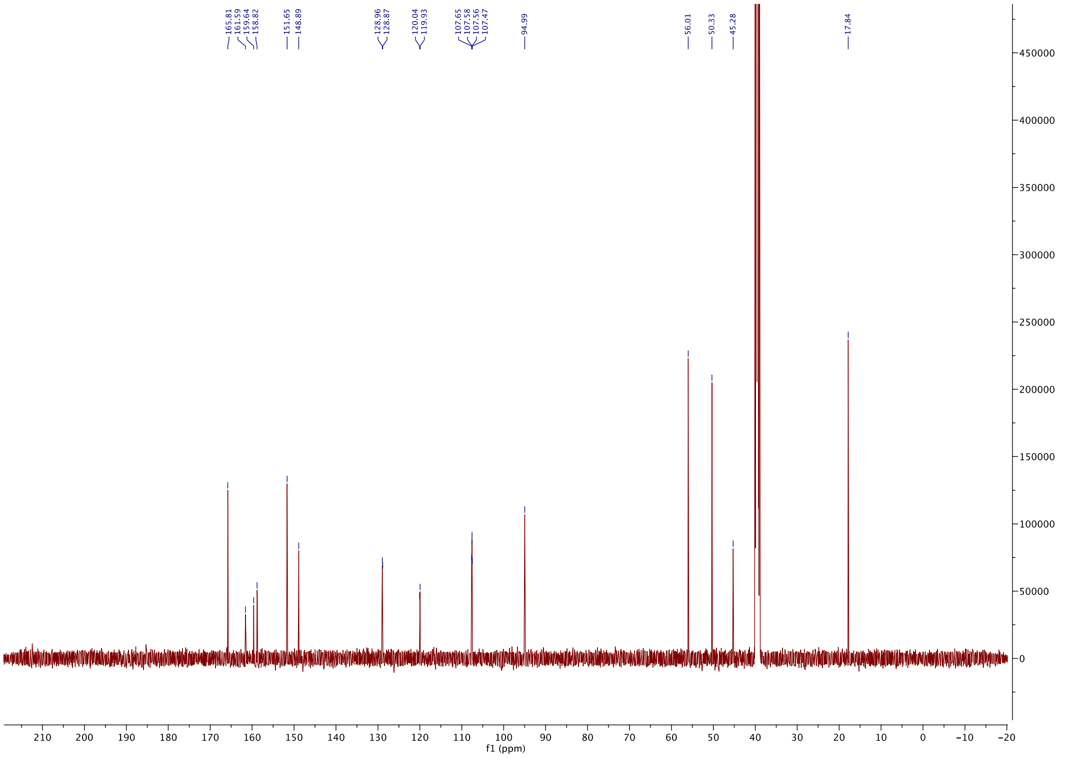
165.81
161.59
159.64
158.82
151.65
148.89
128.96
128.87
120.04
119.93
107.65
107.58
107.56
107.47
94.99
56.01
50.33
45.28
17.84
f1 (ppm)

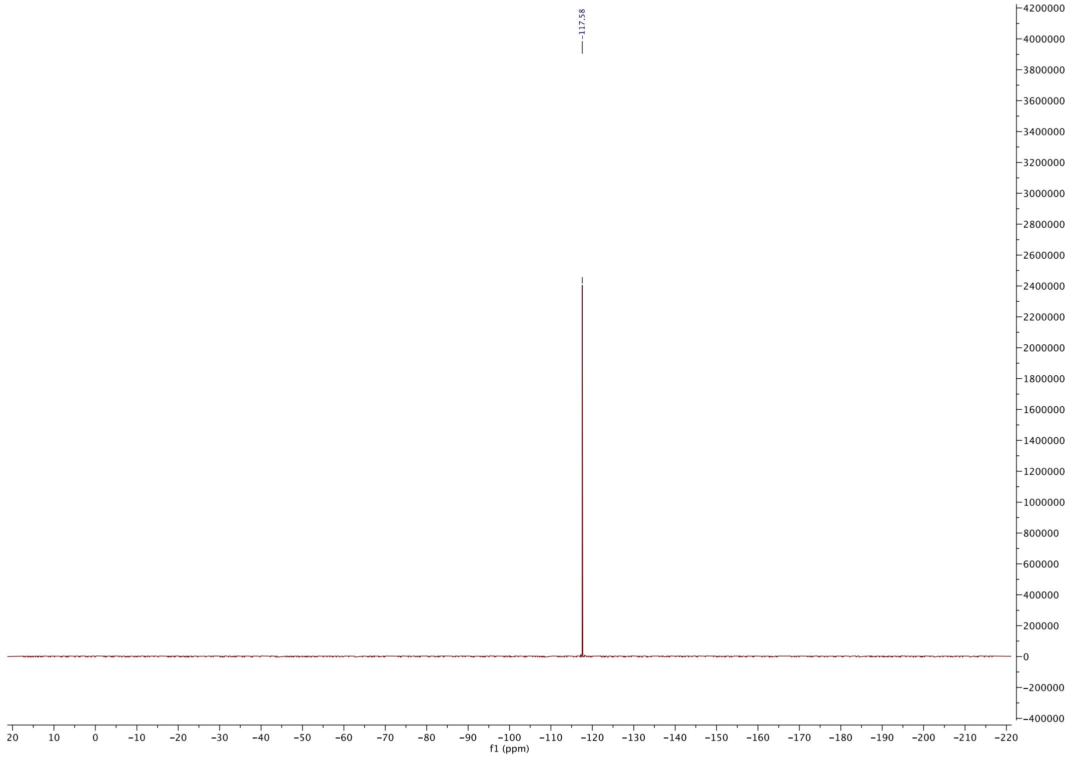
-117.58
f1 (ppm)

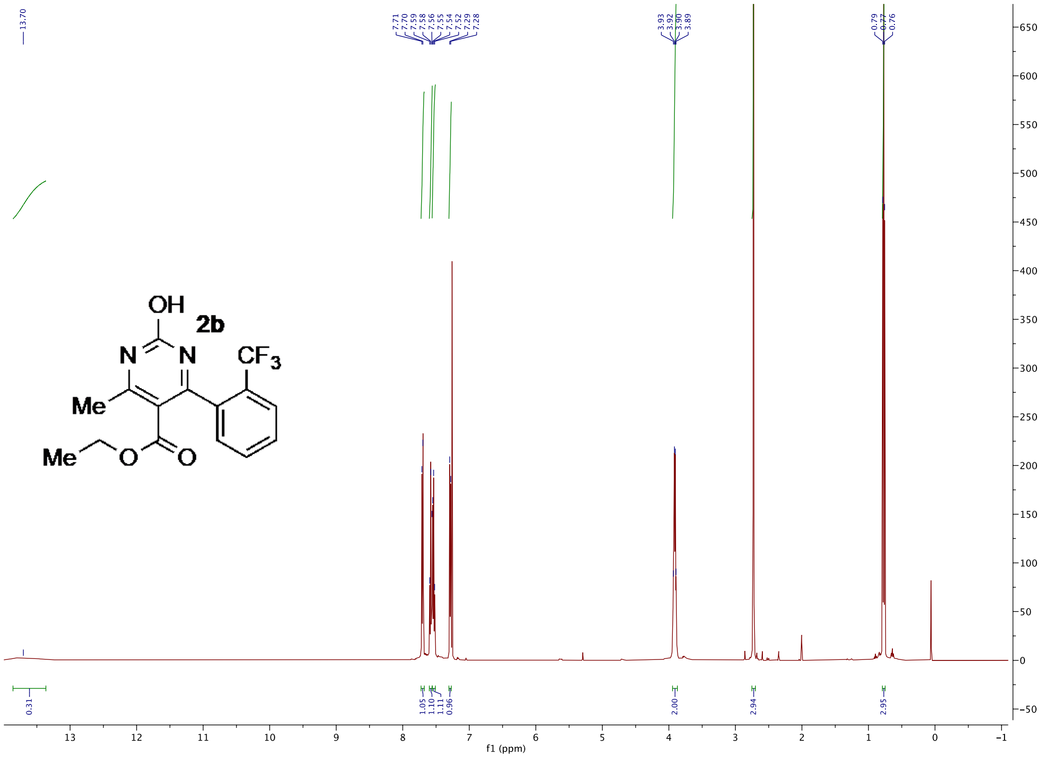
OH
2b
N
N
CF3
Me
Me
O
O
f1 (ppm)

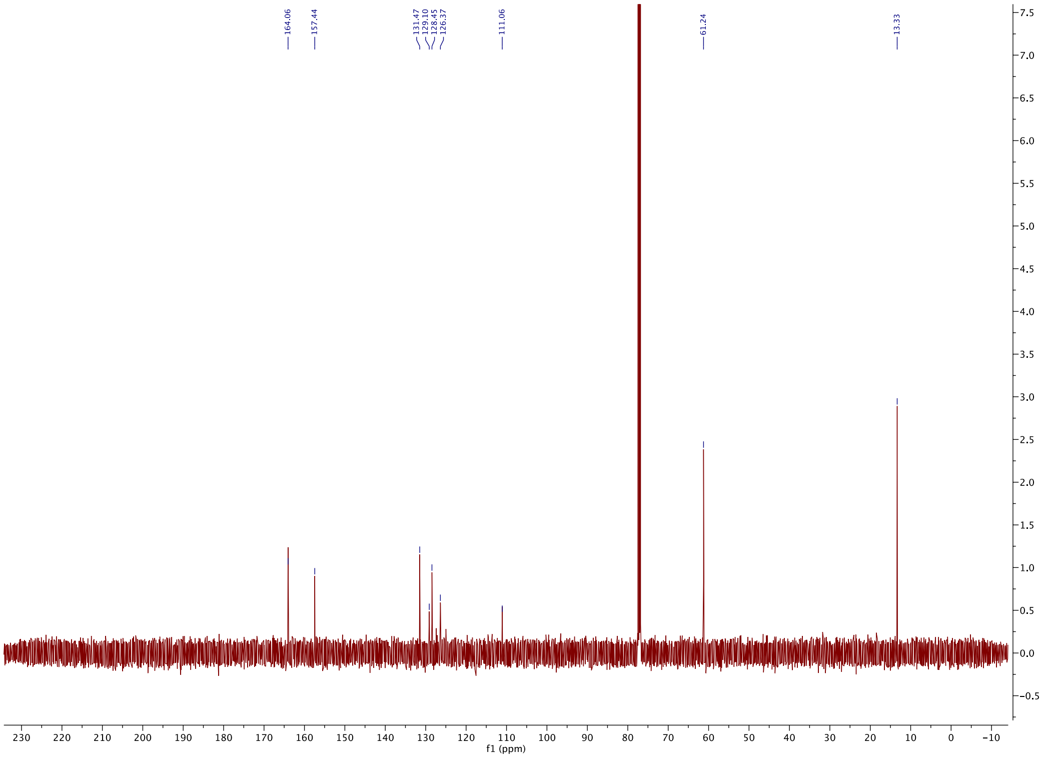
164.06
157.44
131.47
129.10
128.45
126.37
111.06
61.24
13.33
f1 (ppm)

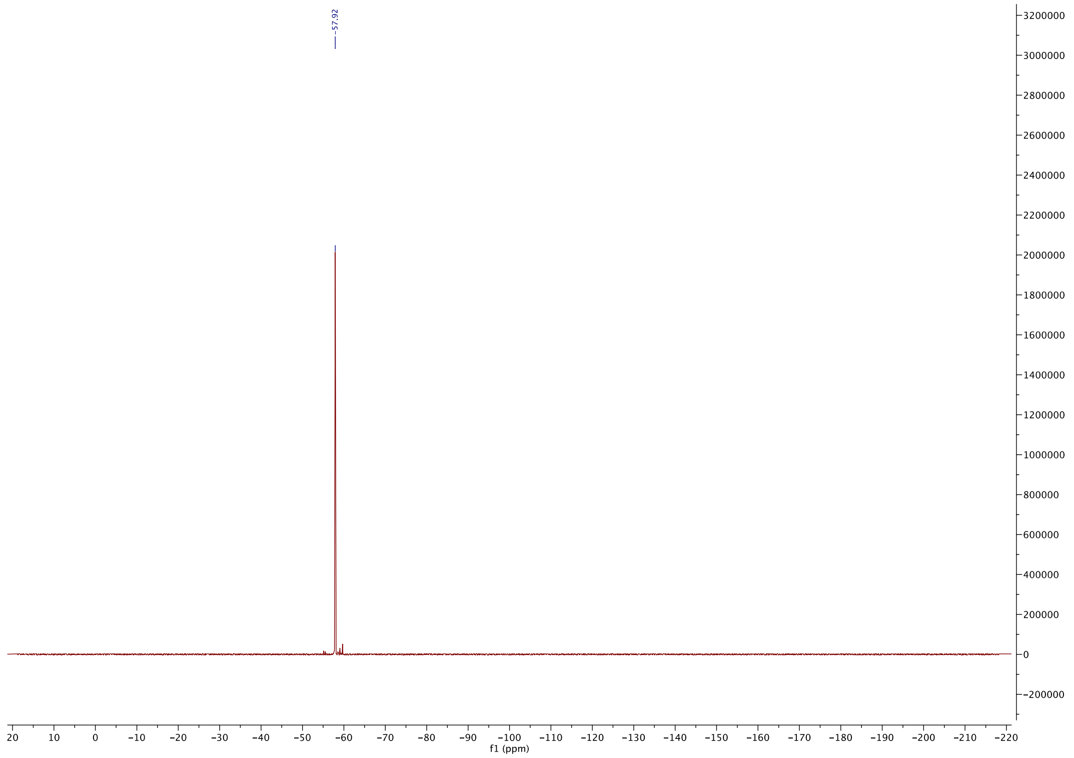

-57.92
3200000
3000000
2800000
2600000
2400000
2200000
2000000
1800000
1600000
1400000
1200000
1000000
800000
600000
400000
200000
0
-200000
20 10 0 -10 -20 -30 -40 -50 -60 -70 -80 -90 -100 -110 -120 -130 -140 -150 -160 -170 -180 -190 -200 -210 -220
f1 (ppm)

2c

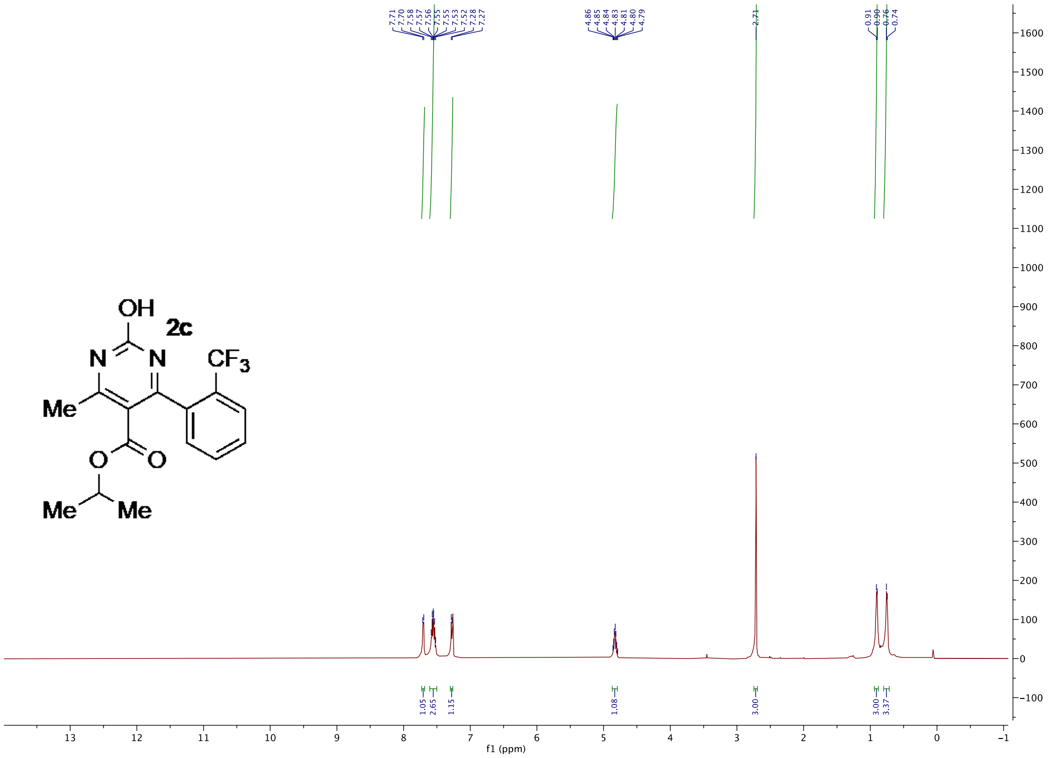
OH
N
N
CF3
Me
O
O
Me
Me
7.71
7.70
7.58
7.57
7.56
7.55
7.55
7.53
7.52
7.28
7.27
4.86
4.85
4.84
4.83
4.81
4.80
4.79
2.71
0.91
0.90
0.76
0.74
1.05
2.65
1.15
1.08
3.00
3.00
3.37
f1 (ppm)

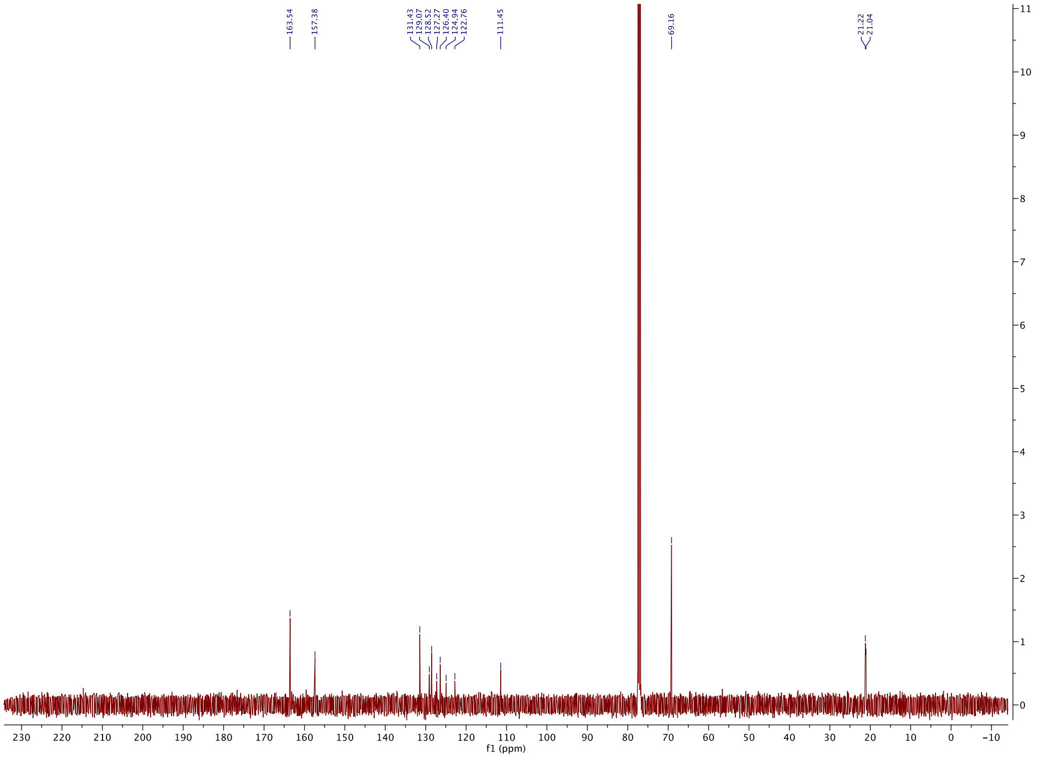
163.54
157.38
131.43
129.07
128.52
127.27
126.40
124.94
122.76
111.45
69.16
21.22
21.04
f1 (ppm)

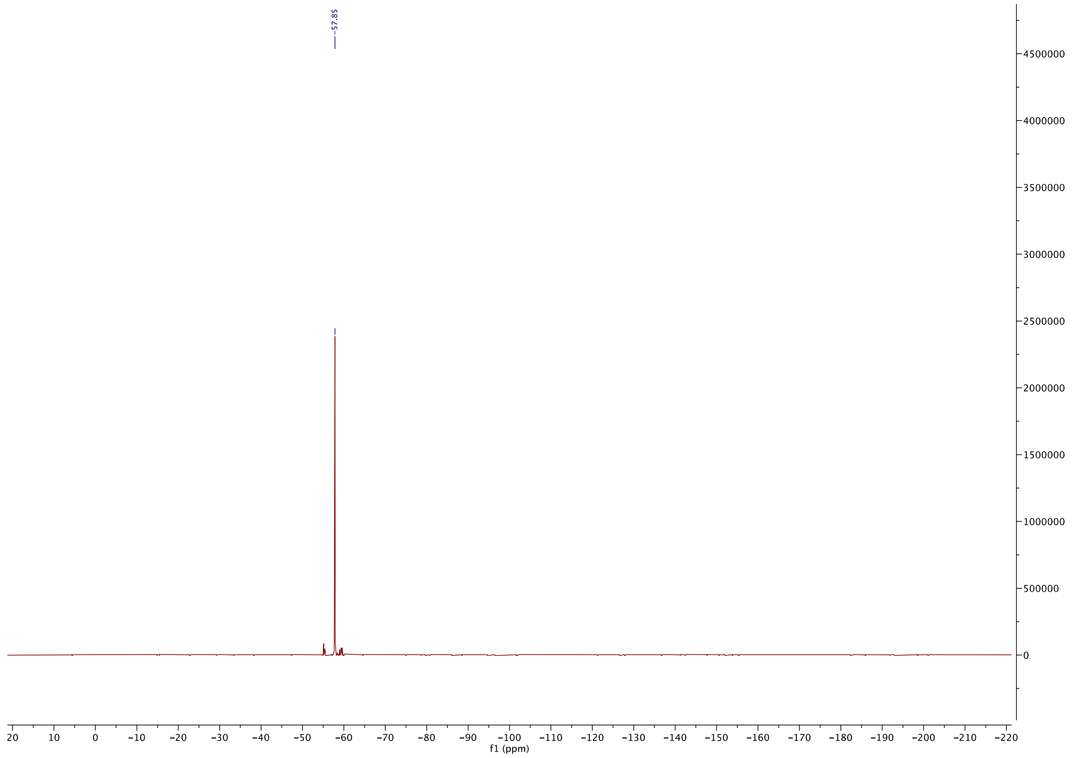

-57.85
4500000
4000000
3500000
3000000
2500000
2000000
1500000
1000000
500000
0
20 10 0 −10 −20 −30 −40 −50 −60 −70 −80 −90 −100 −110 −120 −130 −140 −150 −160 −170 −180 −190 −200 −210 −220
f1 (ppm)

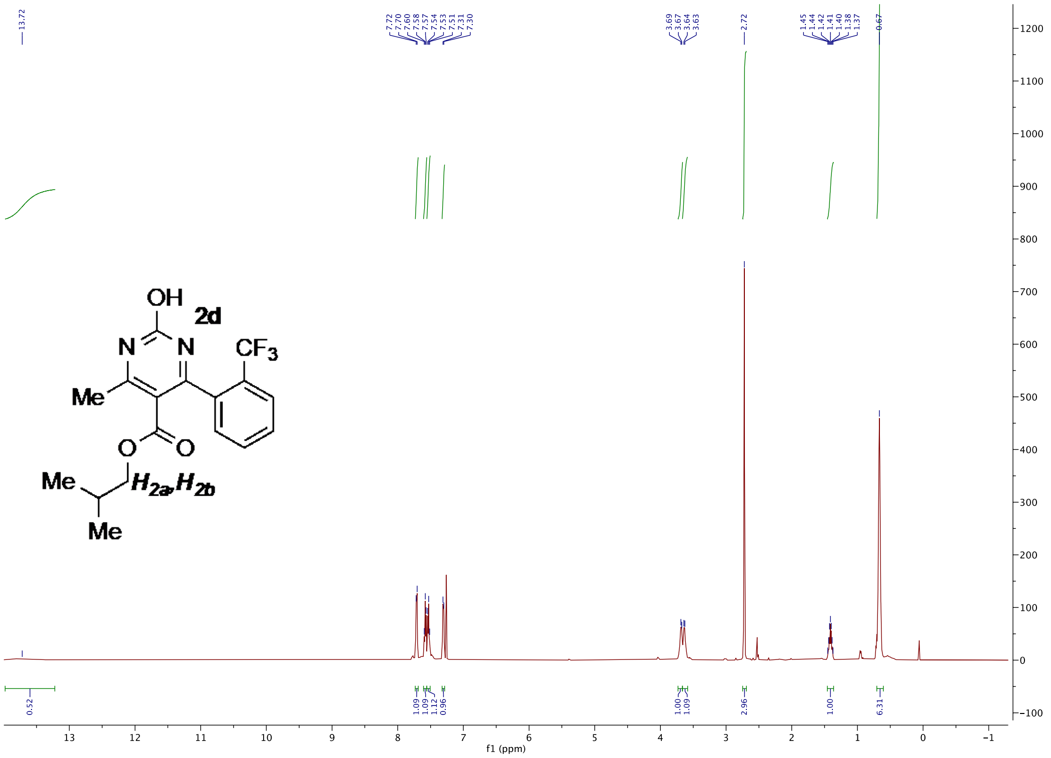

13.72
7.72 7.70 7.60 7.58 7.57 7.54 7.53 7.51 7.31 7.30
3.69 3.67 3.64 3.63
2.72
1.45 1.44 1.42 1.41 1.40 1.38 1.37
0.67
2d
0.52
1.09 1.09 1.12 0.96
1.00 1.09
2.96
1.00
6.31
f1 (ppm)

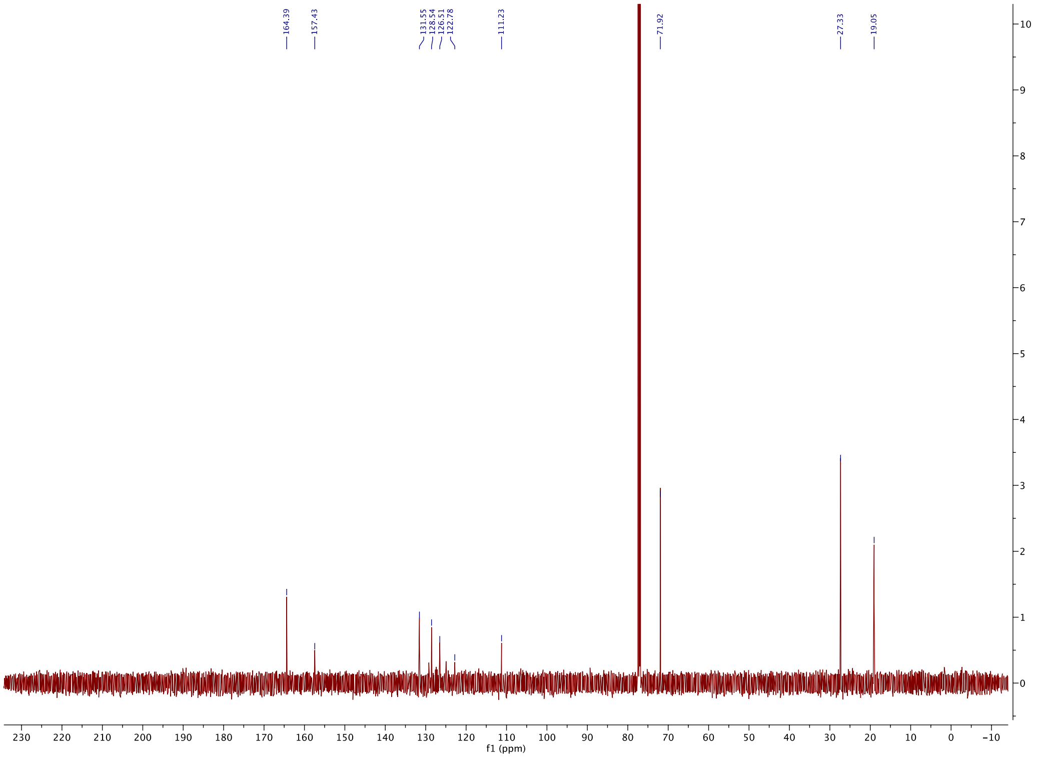

164.39
157.43
131.55 128.54 126.51 122.78
111.23
71.92
27.33
19.05
f1 (ppm)

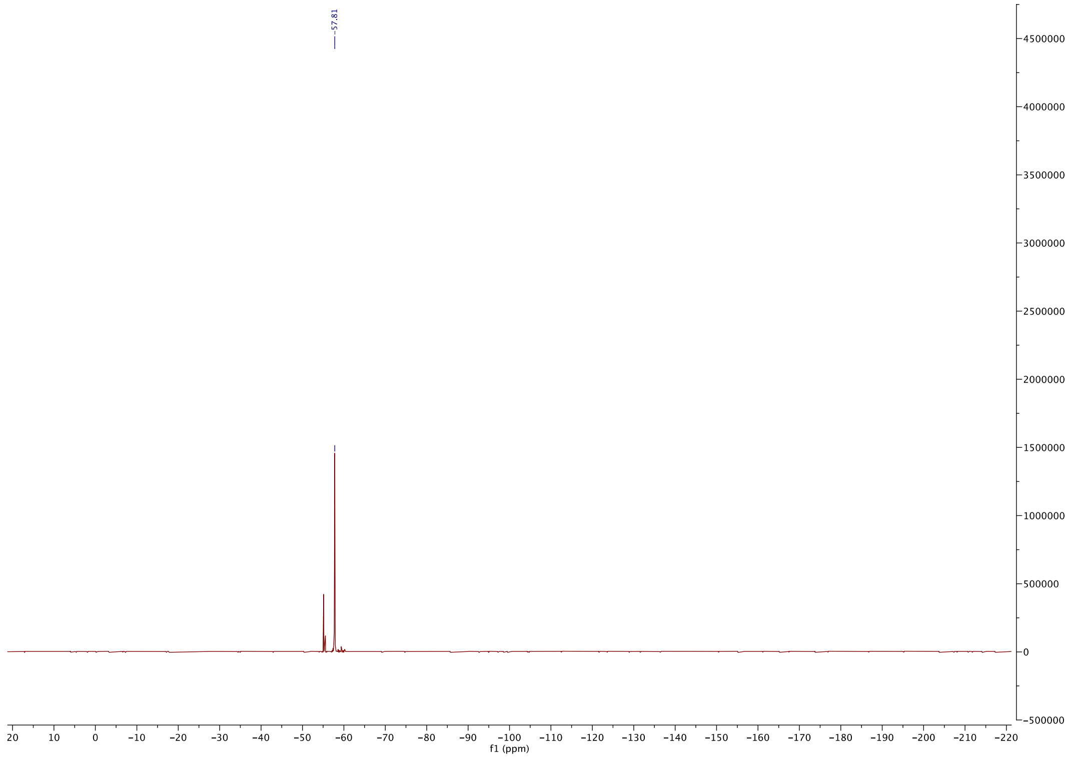
-57.81
4500000
4000000
3500000
3000000
2500000
2000000
1500000
1000000
500000
0
-500000
20 10 0 -10 -20 -30 -40 -50 -60 -70 -80 -90 -100 -110 -120 -130 -140 -150 -160 -170 -180 -190 -200 -210 -220
f1 (ppm)

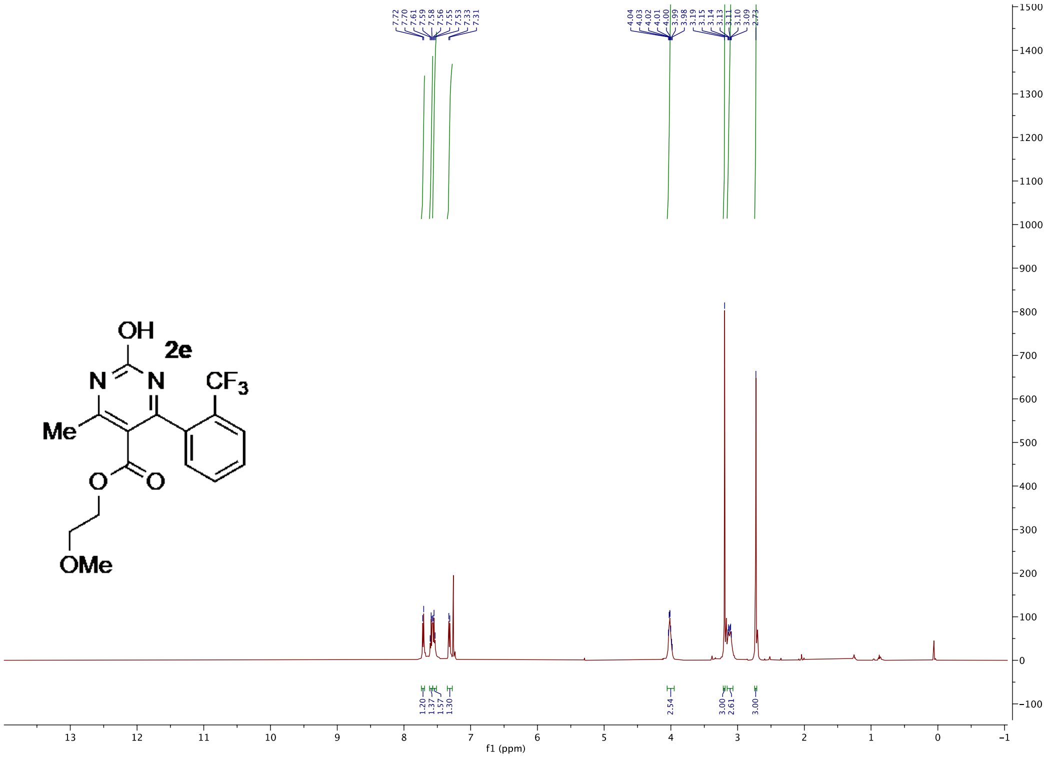
OH
2e
N
N
CF3
Me
O
O
OMe
7.72
7.70
7.61
7.59
7.58
7.56
7.55
7.53
7.33
7.31
4.04
4.03
4.02
4.01
4.00
3.99
3.98
3.19
3.15
3.14
3.13
3.11
3.10
3.09
2.73
1.20
1.37
1.57
1.30
2.54
3.00
2.61
3.00
f1 (ppm)

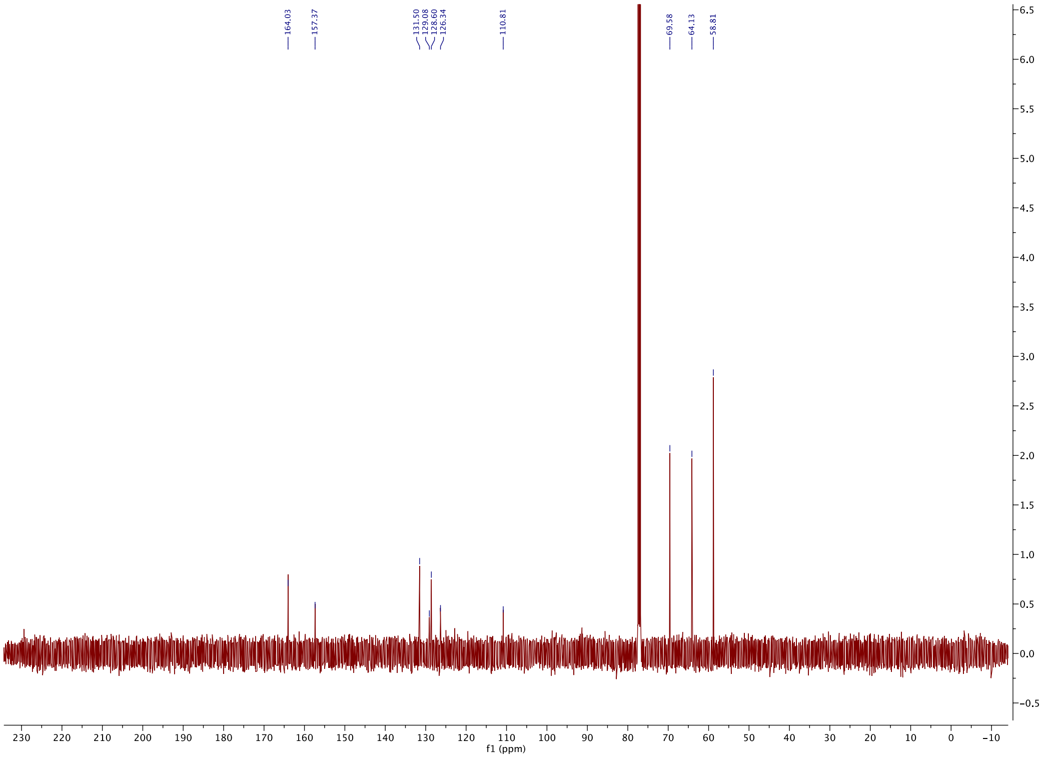
164.03
157.37
131.50
129.08
128.60
126.34
110.81
69.58
64.13
58.81
f1 (ppm)

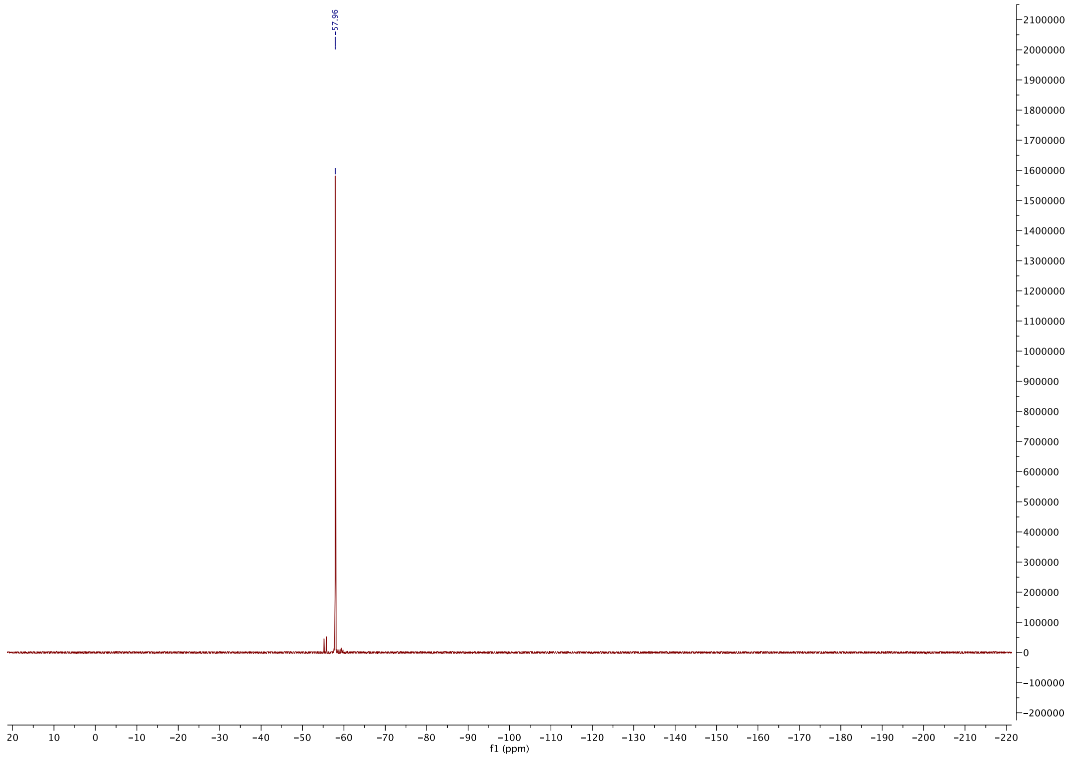
-57.96
f1 (ppm)

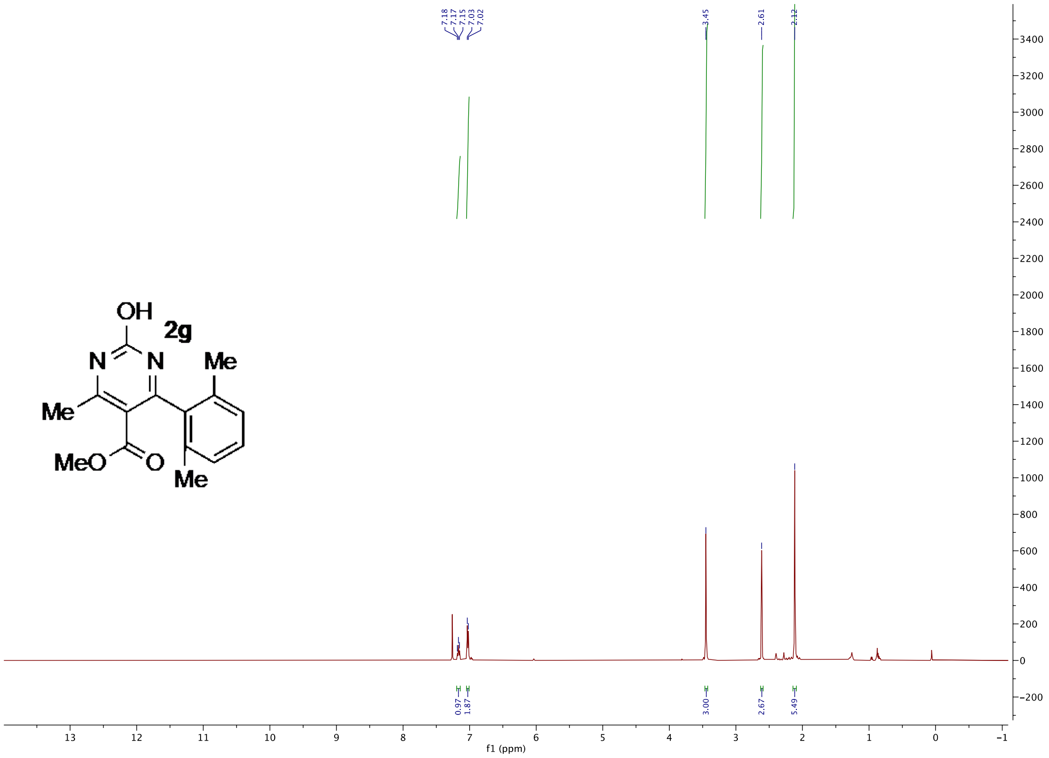
2g
OH
N
N
Me
Me
MeO
O
Me
7.18
7.17
7.15
7.03
7.02
3.45
2.61
2.12
0.97
1.87
3.00
2.67
5.49
f1 (ppm)

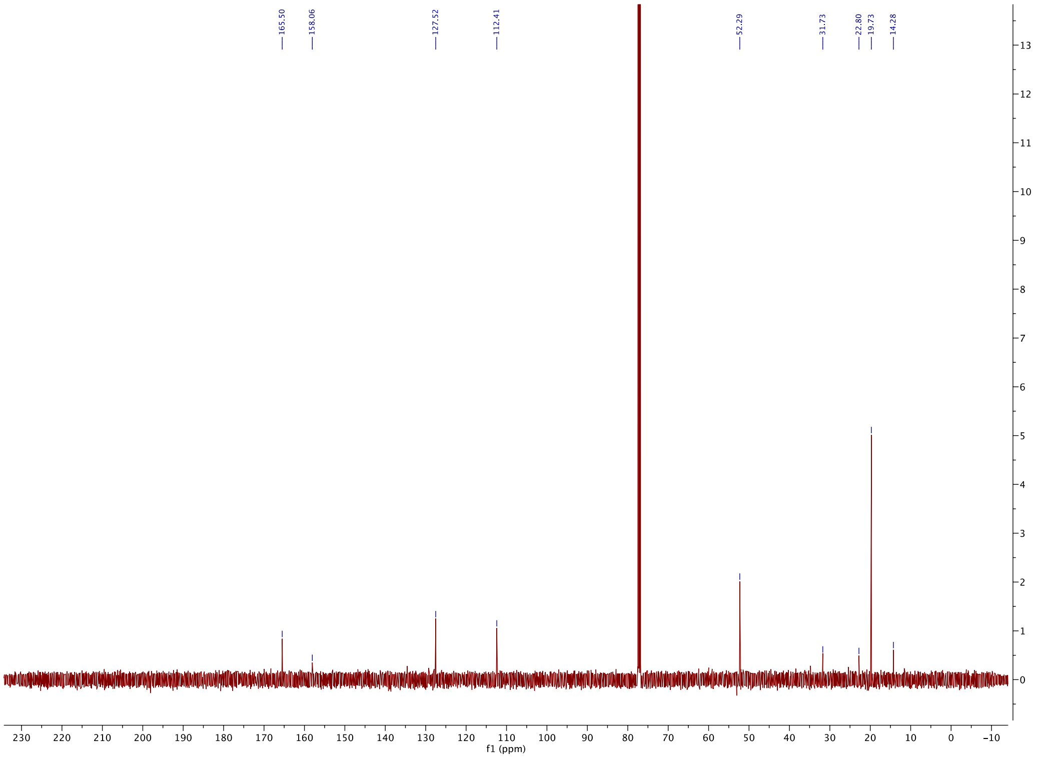
165.50
158.06
127.52
112.41
52.29
31.73
22.80
19.73
14.28
f1 (ppm)

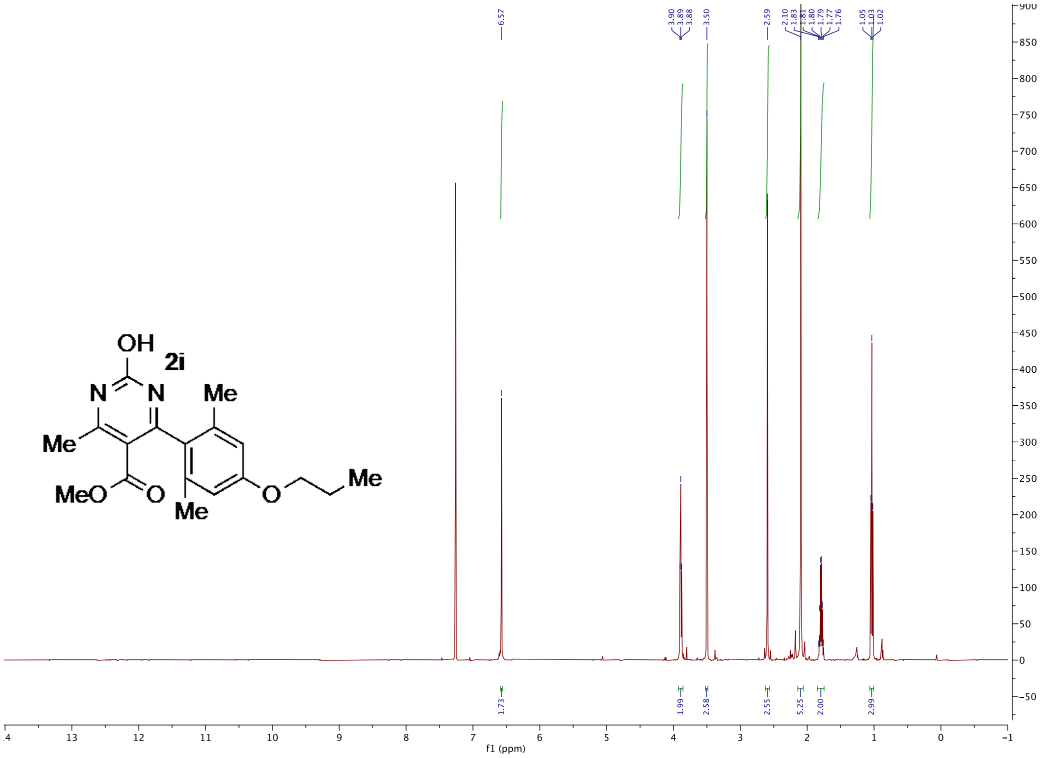
OH
2i
N
N
Me
Me
MeO
O
Me
O
Me
f1 (ppm)

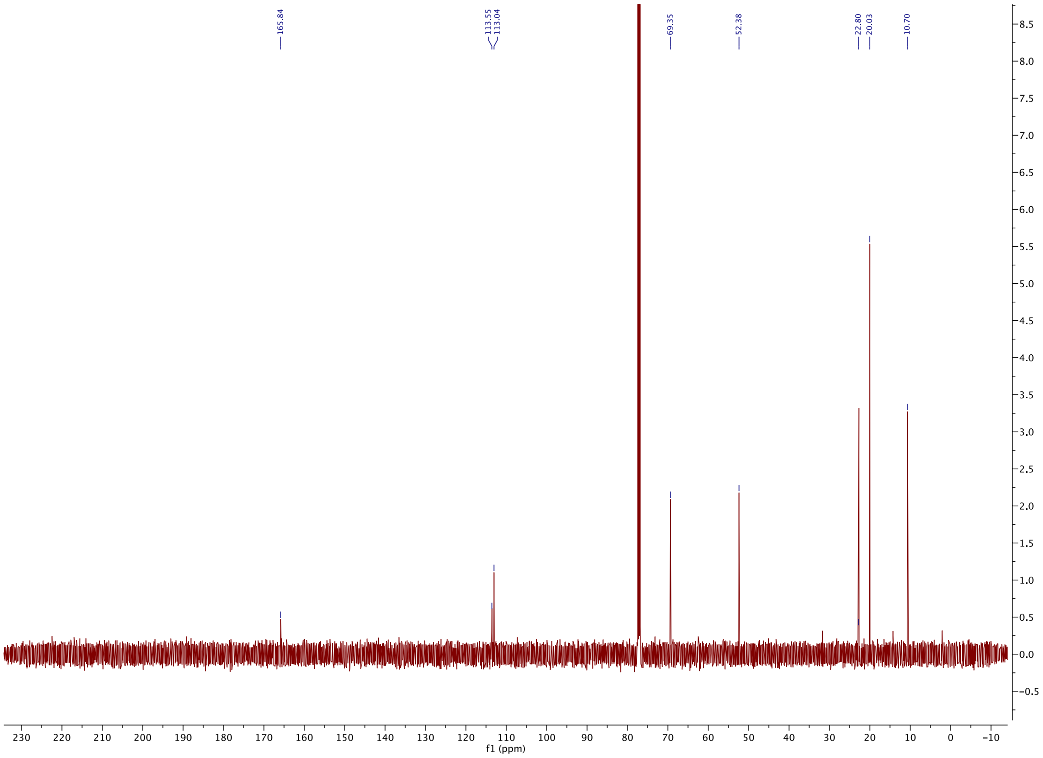
f1 (ppm)

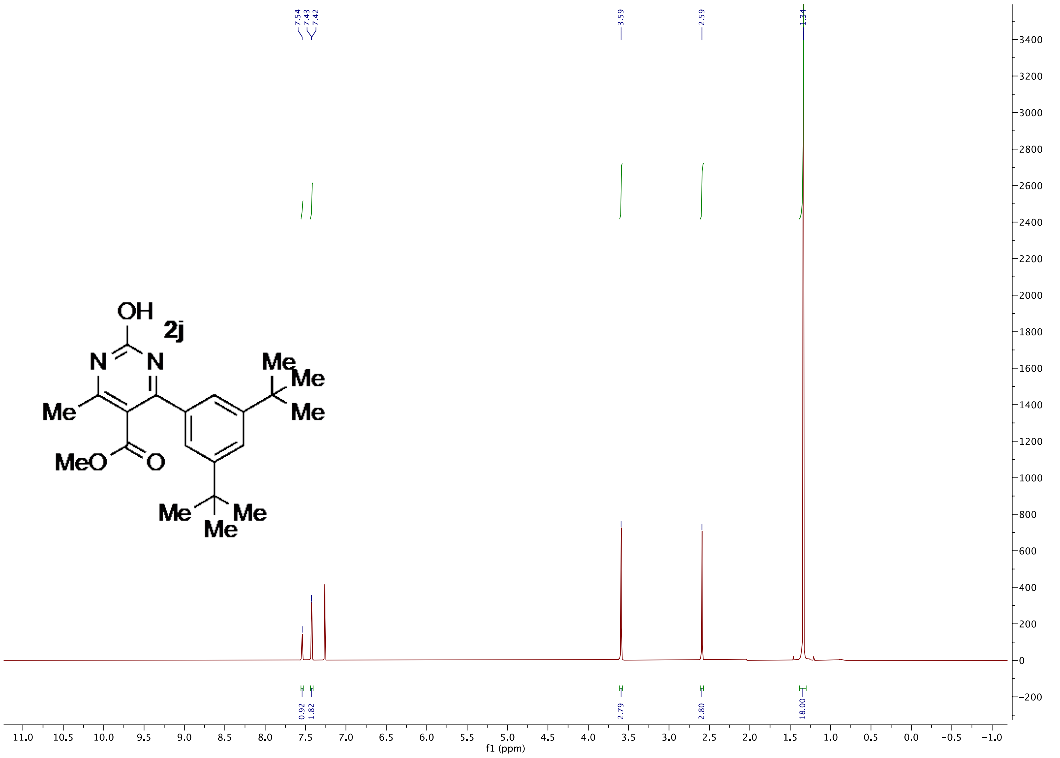
7.54
7.43
7.42
3.59
2.59
1.34
OH
2j
N
N
Me
Me
Me
Me
MeO
O
Me
Me
Me
0.92
1.82
2.79
2.80
18.00
f1 (ppm)

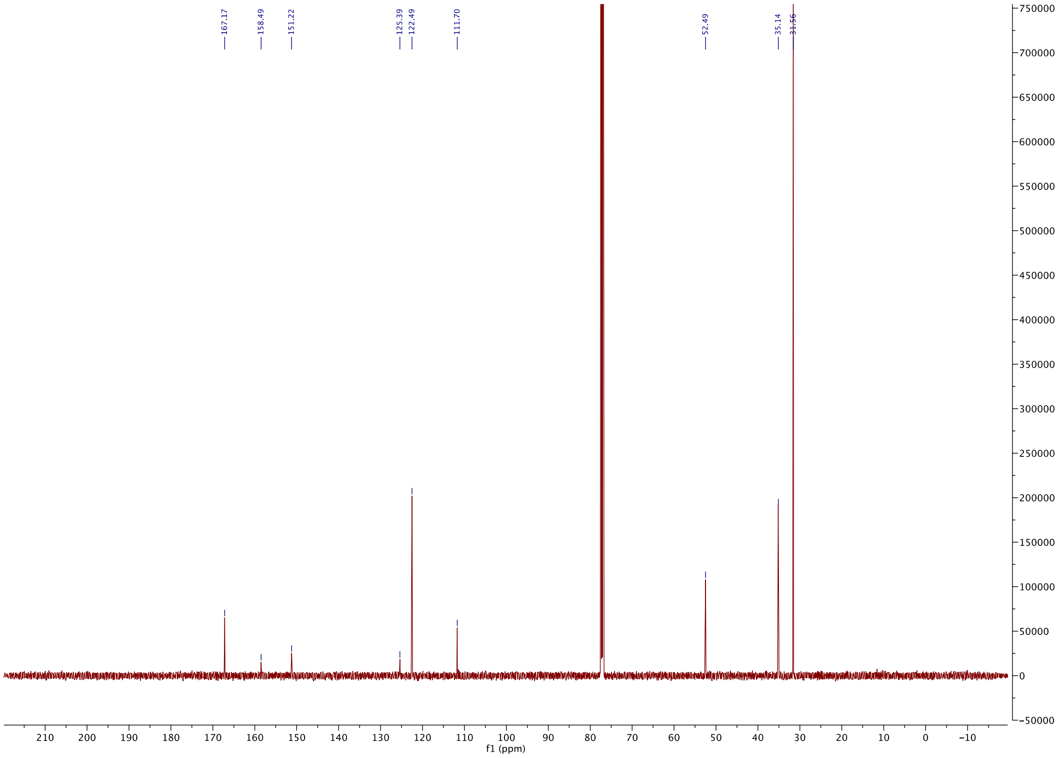
167.17
158.49
151.22
125.39
122.49
111.70
52.49
35.14
31.56
f1 (ppm)

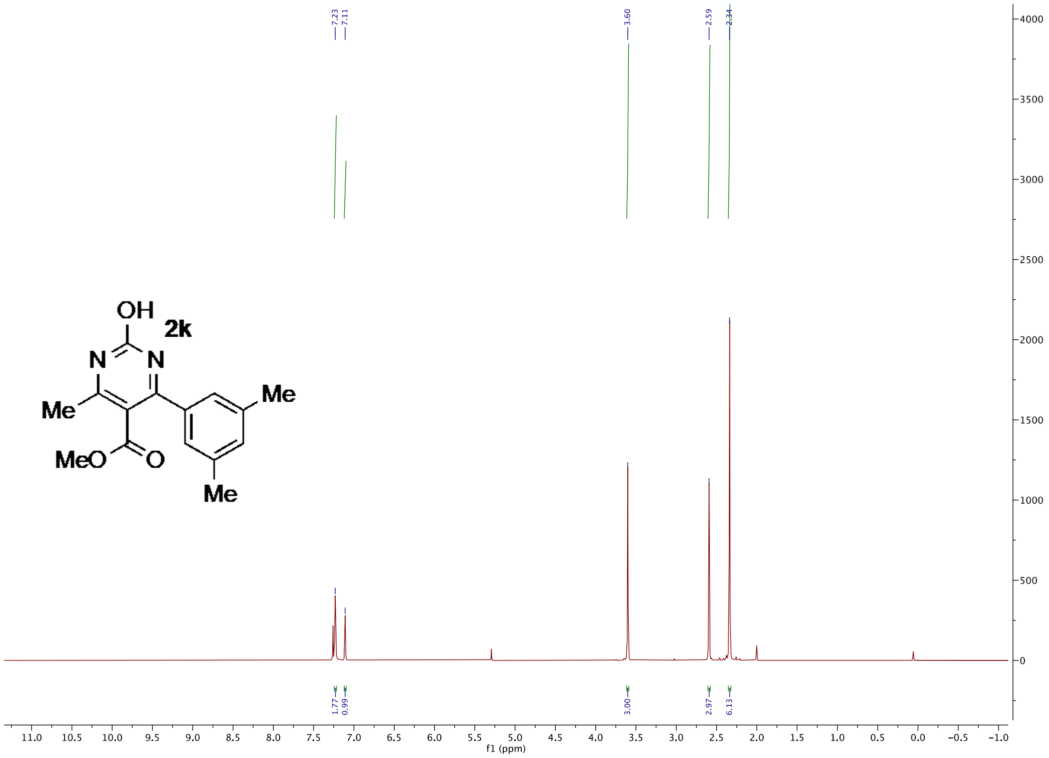
7.23
7.11
3.60
2.59
2.34
OH
2k
N
N
Me
Me
MeO
O
Me
1.77
0.99
3.00
2.97
6.13
f1 (ppm)

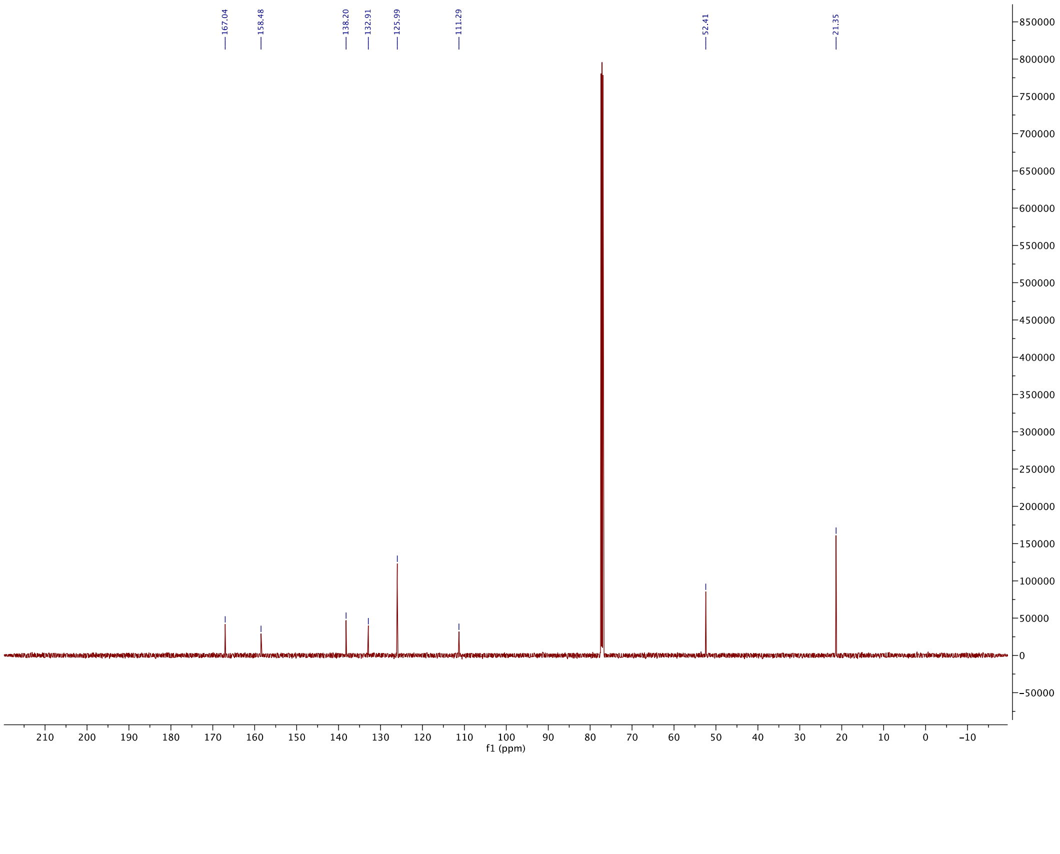
167.04
158.48
138.20
132.91
125.99
111.29
52.41
21.35
f1 (ppm)

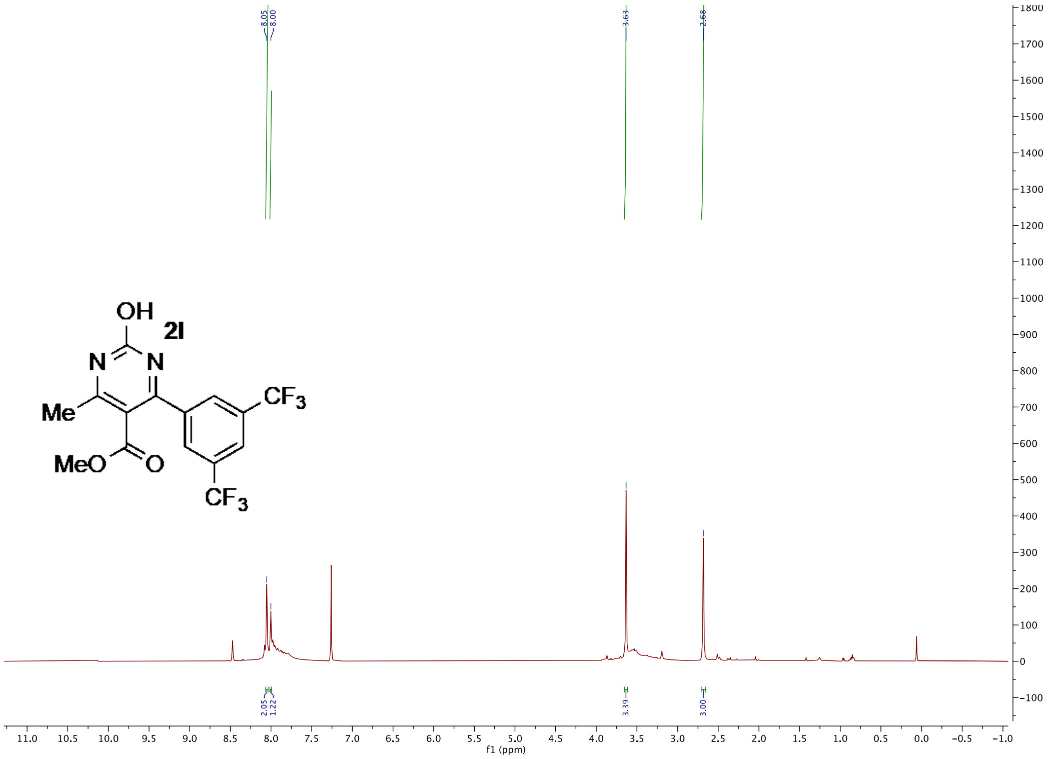
8.05
8.00
3.63
2.68
OH
2l
N
N
Me
CF_3
MeO
O
CF_3
2.05
1.22
3.39
3.00
f1 (ppm)

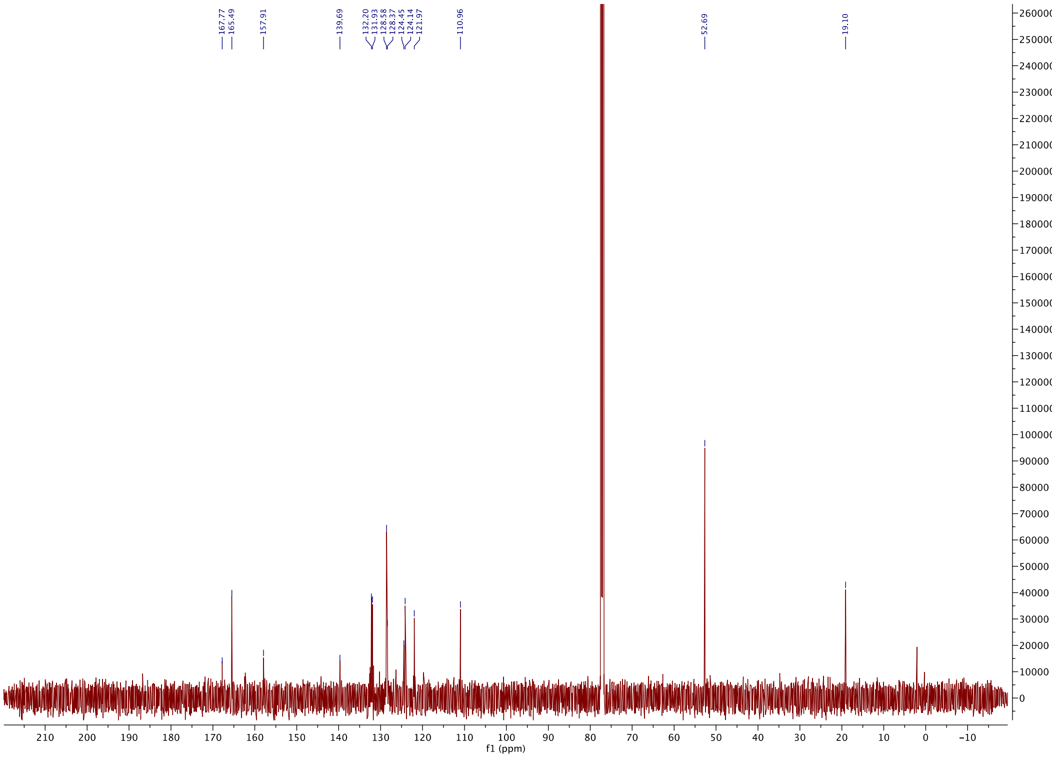
167.77
165.49
157.91
139.69
132.20
131.93
128.58
128.37
124.45
124.14
121.97
110.96
52.69
19.10
f1 (ppm)

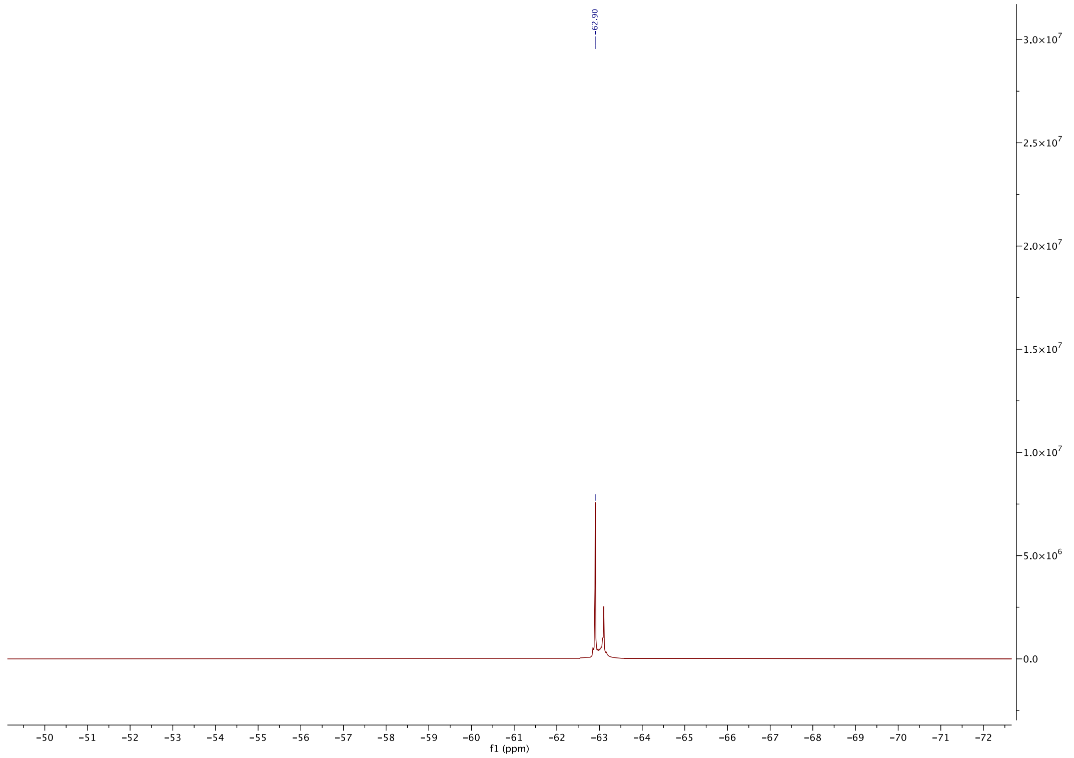
-62.90
3.0×10^7
2.5×10^7
2.0×10^7
1.5×10^7
1.0×10^7
5.0×10^6
0.0
-50 -51 -52 -53 -54 -55 -56 -57 -58 -59 -60 -61 -62 -63 -64 -65 -66 -67 -68 -69 -70 -71 -72
f1 (ppm)

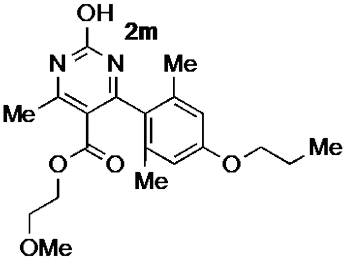
OH
N
N
Me
Me
O
O
Me
O
Me
OMe
2m

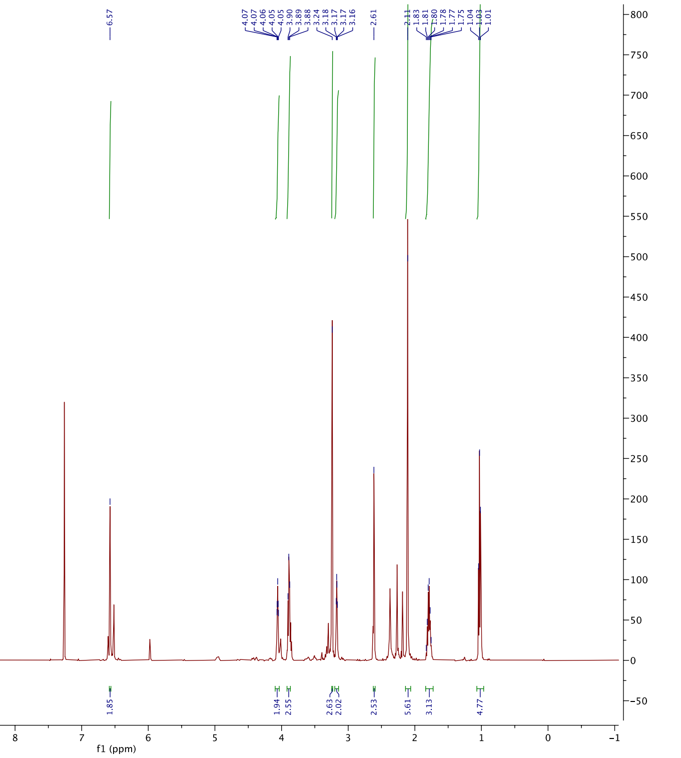
6.57
4.07
4.07
4.06
4.05
4.05
3.90
3.89
3.88
3.24
3.18
3.17
3.17
3.16
2.61
2.11
1.83
1.81
1.80
1.78
1.77
1.75
1.04
1.03
1.01
1.85
1.94
2.55
2.63
2.02
2.53
5.61
3.13
4.77
f1 (ppm)

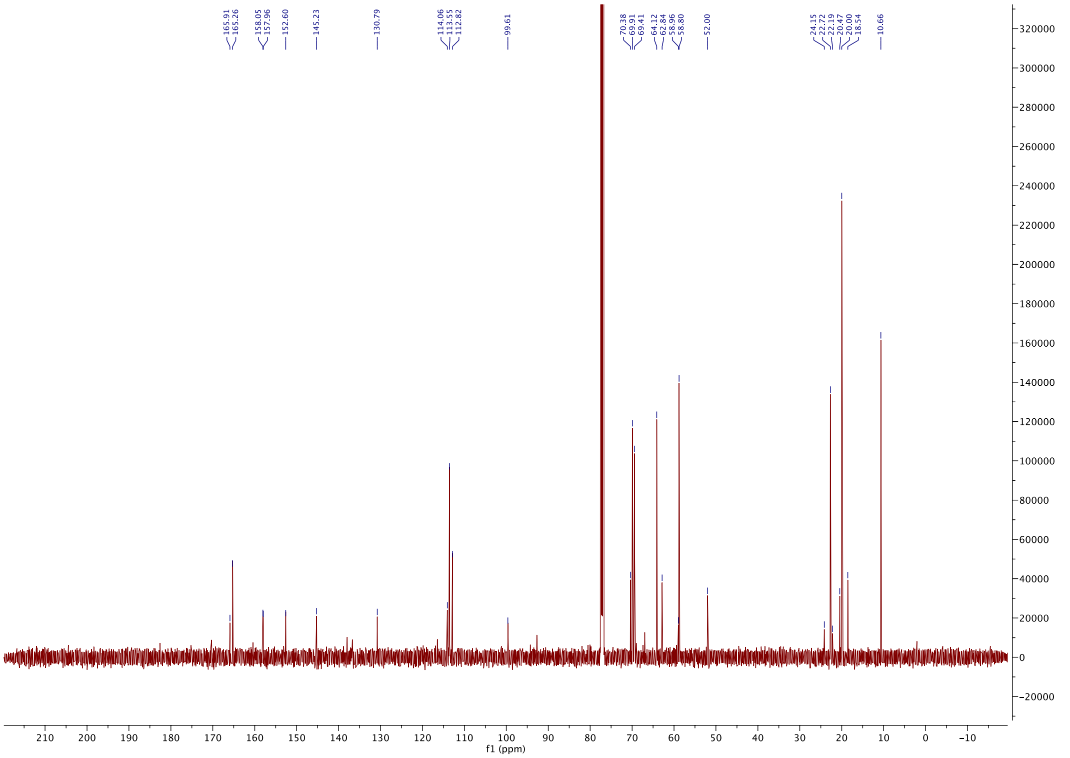
165.91
165.26
158.05
157.96
152.60
145.23
130.79
114.06
113.55
112.82
99.61
70.38
69.91
69.41
64.12
62.84
58.96
58.80
52.00
24.15
22.72
22.19
20.47
20.00
18.54
10.66
f1 (ppm)

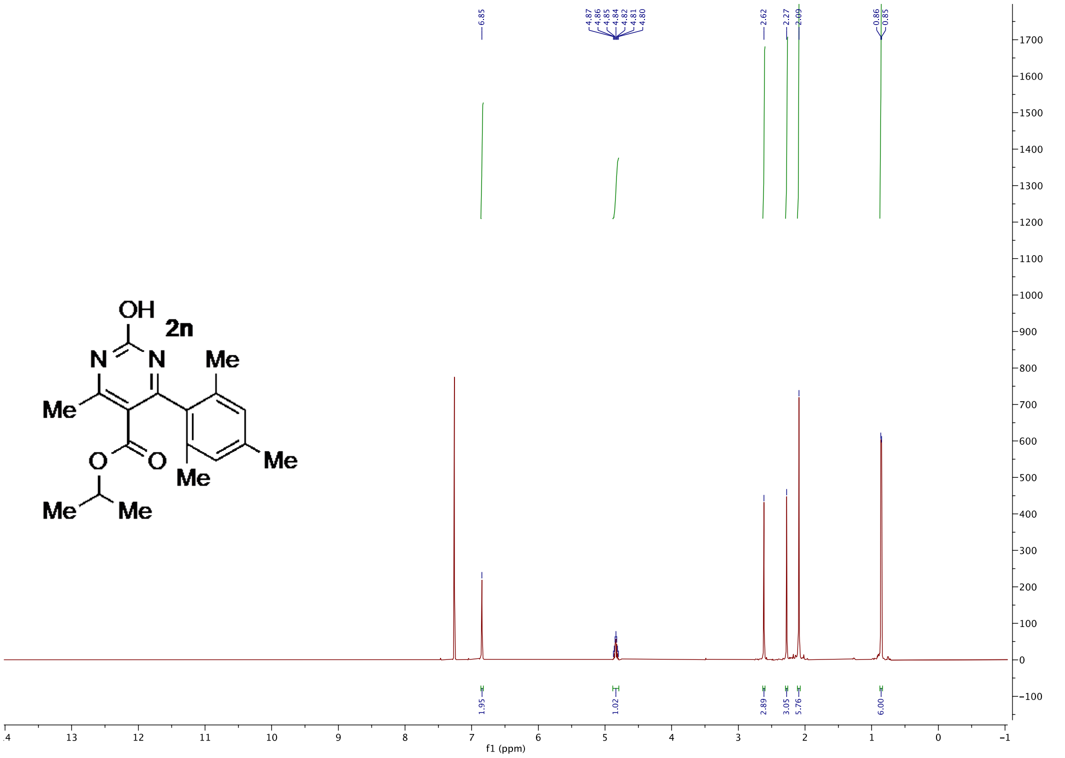

6.85
4.87
4.86
4.85
4.84
4.82
4.81
4.80
2.62
2.27
2.09
0.86
0.85
1.95
1.02
2.89
3.05
5.76
6.00
f1 (ppm)
OH
2n
N
N
Me
Me
O
O
Me
Me
Me
Me

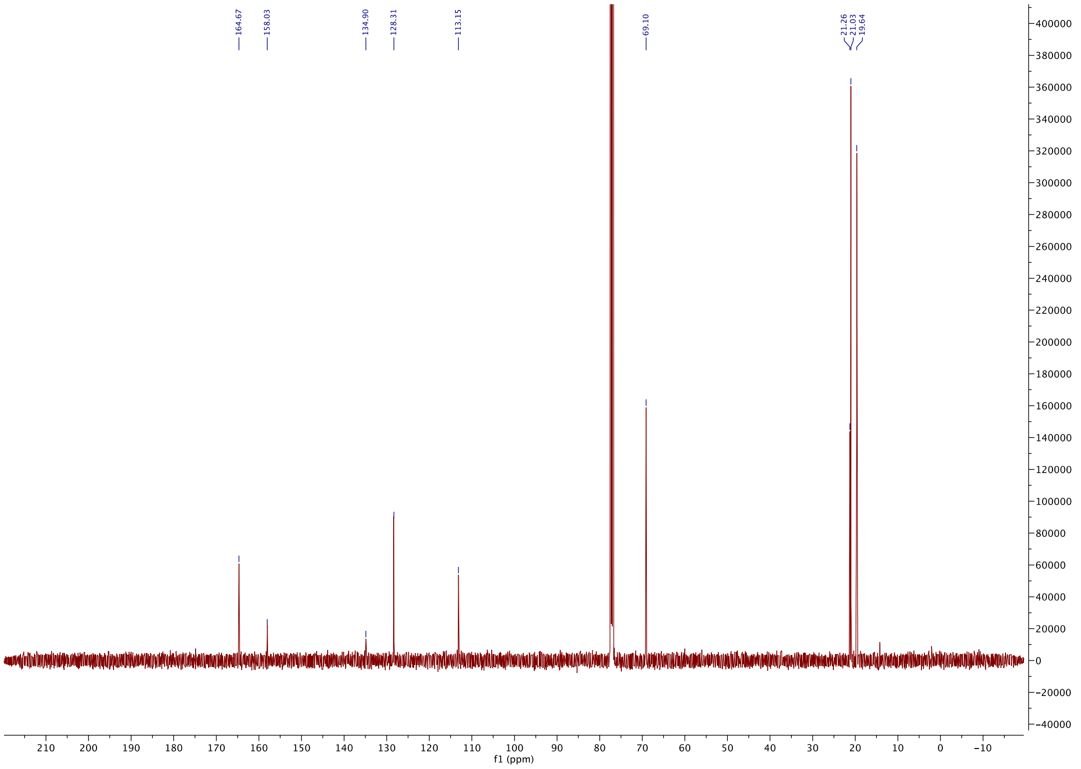

164.67
158.03
134.90
128.31
113.15
69.10
21.26
21.03
19.64
f1 (ppm)

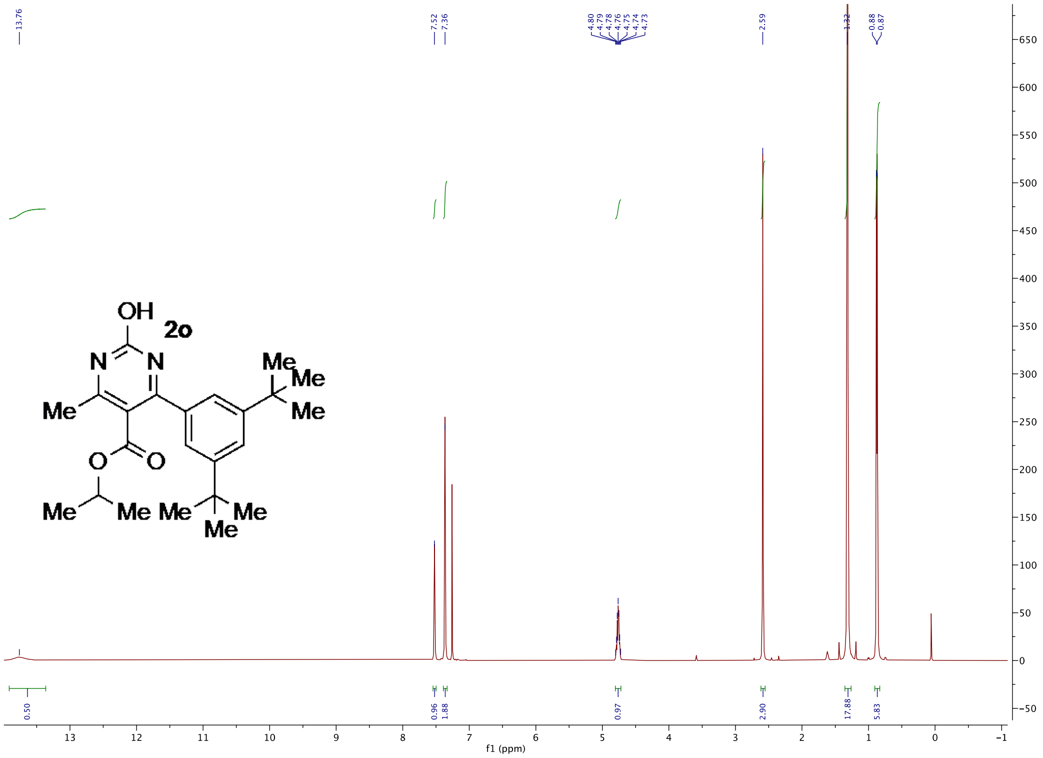
13.76
7.52
7.36
4.80
4.79
4.78
4.76
4.75
4.74
4.73
2.59
1.32
0.88
0.87
OH
2o
N
N
Me
Me
Me
Me
O
O
Me
Me Me
Me
Me
0.50
0.96
1.88
0.97
2.90
17.88
5.83
f1 (ppm)

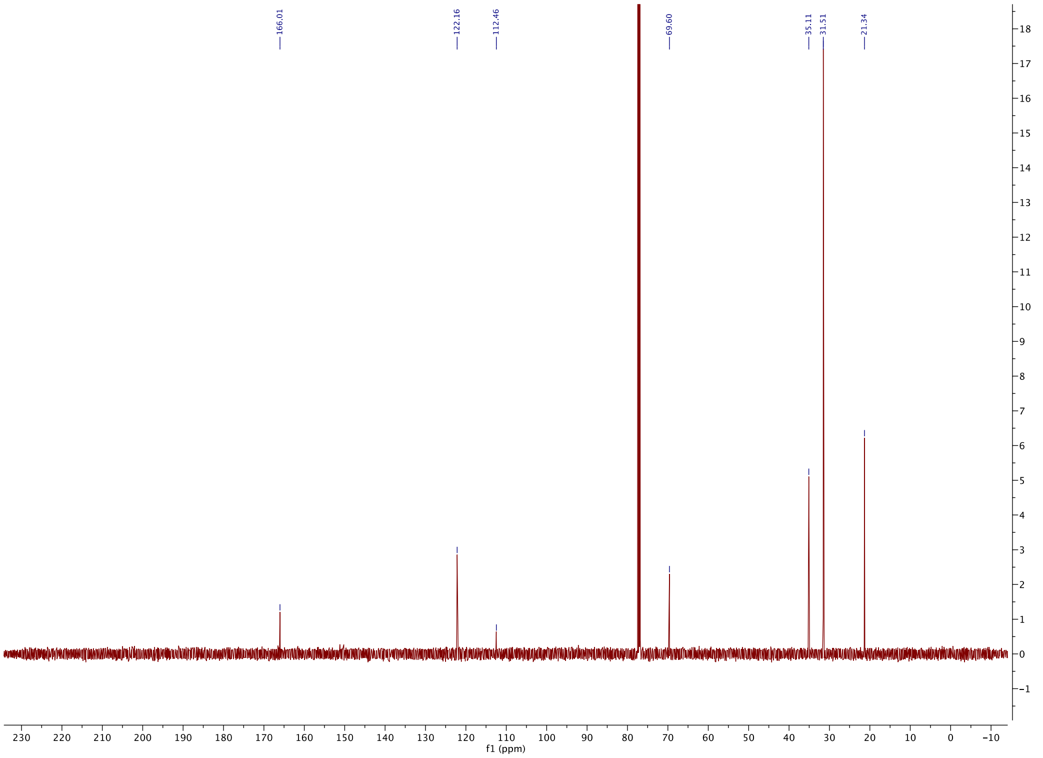
166.01
122.16
112.46
69.60
35.11
31.51
21.34
f1 (ppm)

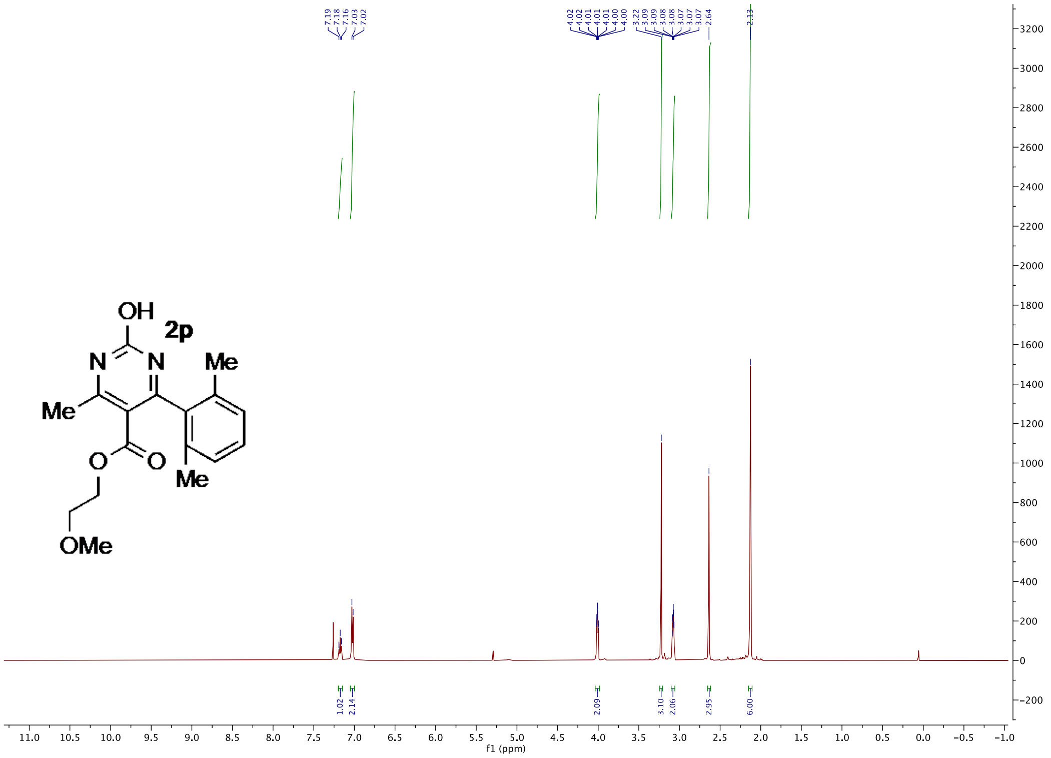
7.19
7.18
7.16
7.03
7.02
4.02
4.02
4.01
4.01
4.01
4.00
4.00
3.22
3.09
3.09
3.08
3.08
3.07
3.07
3.07
2.64
2.13
OH
2p
N
N
Me
Me
Me
O
O
OMe
1.02
2.14
2.09
3.10
2.06
2.95
6.00
f1 (ppm)

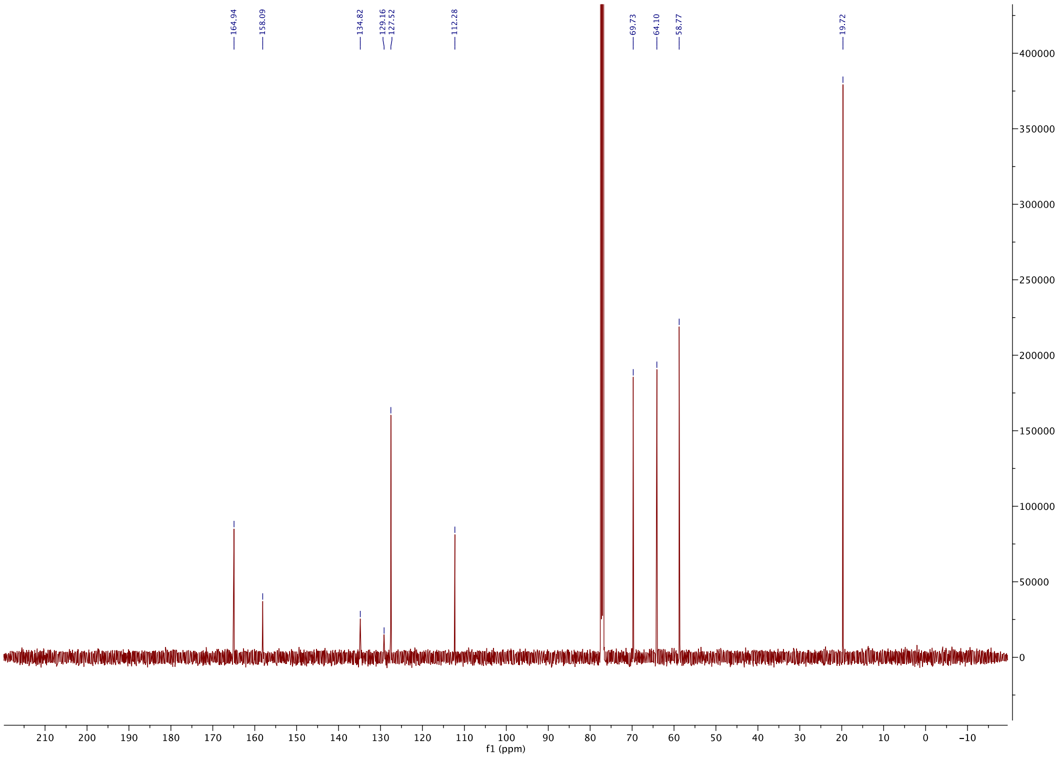
164.94
158.09
134.82
129.16
127.52
112.28
69.73
64.10
58.77
19.72
f1 (ppm)

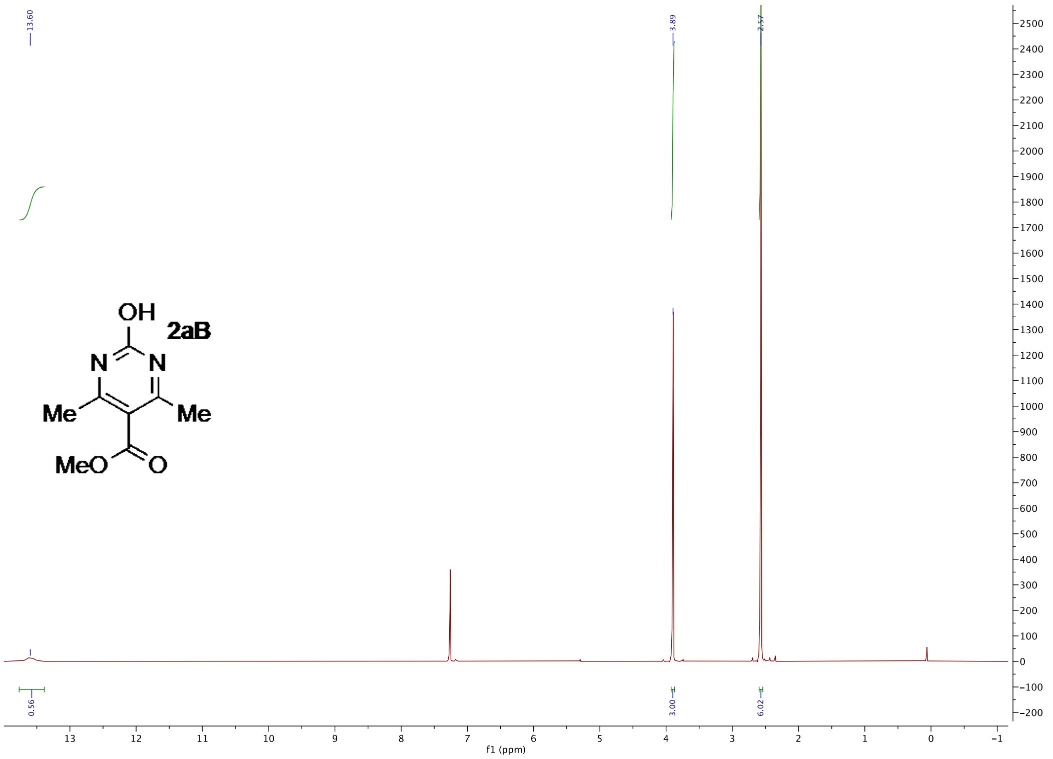
13.60
3.89
2.57
OH
2aB
N
N
Me
Me
MeO
O
0.56
3.00
6.02
f1 (ppm)

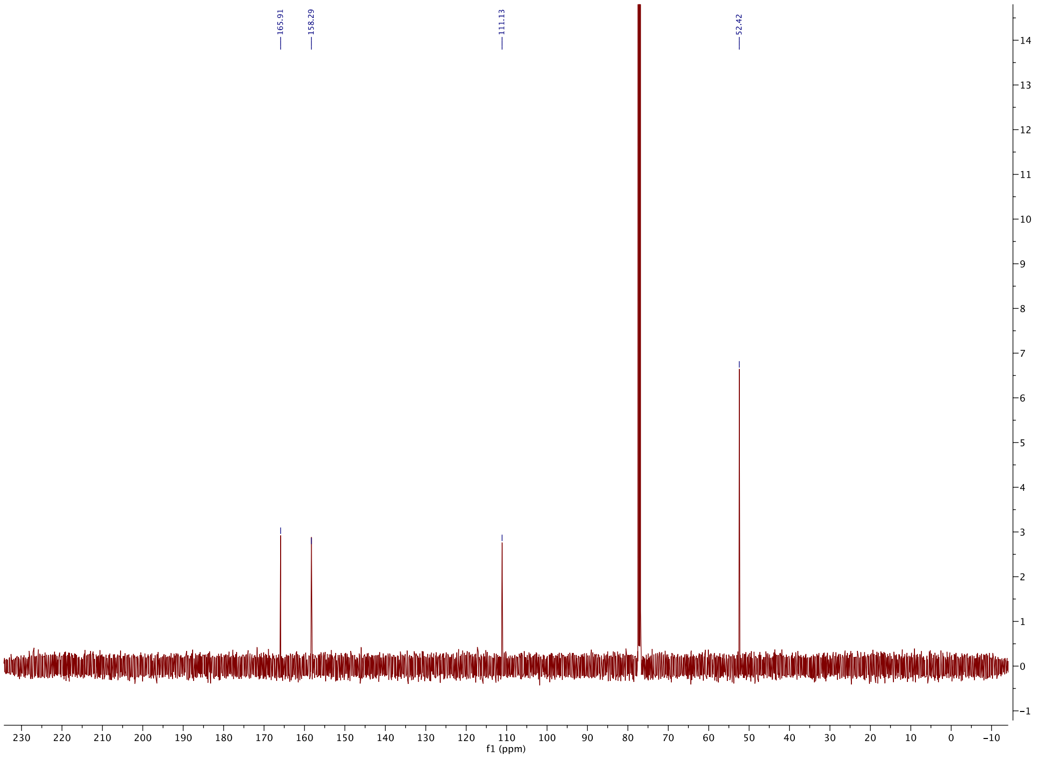
165.91
158.29
111.13
52.42
f1 (ppm)

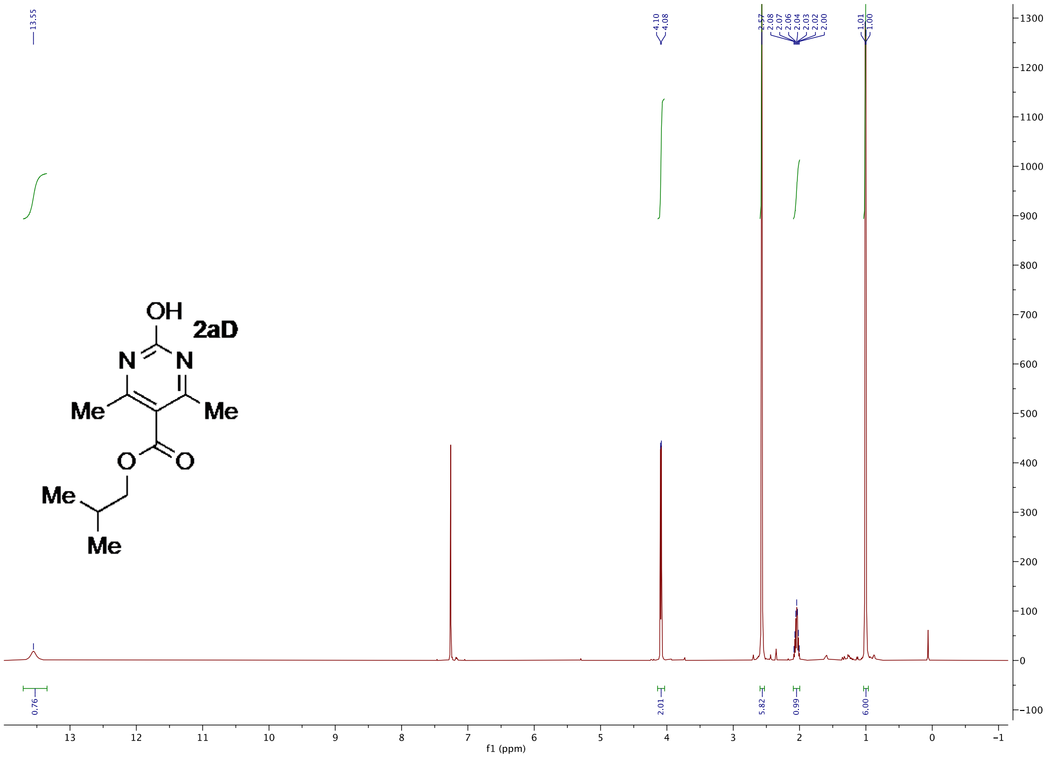
2aD
OH
N
N
Me
Me
O
O
Me
Me
13.55
4.10
4.08
2.57
2.08
2.07
2.06
2.04
2.03
2.02
2.00
1.01
1.00
0.76
2.01
5.82
0.99
6.00
f1 (ppm)

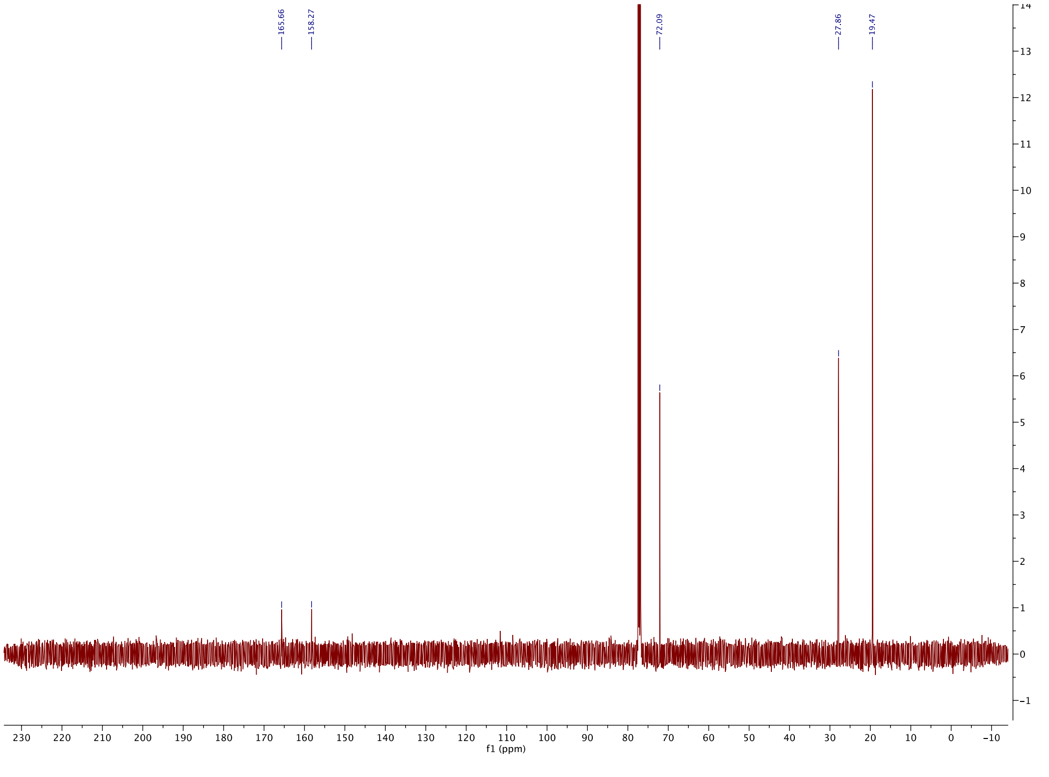
165.66
158.27
72.09
27.86
19.47
f1 (ppm)

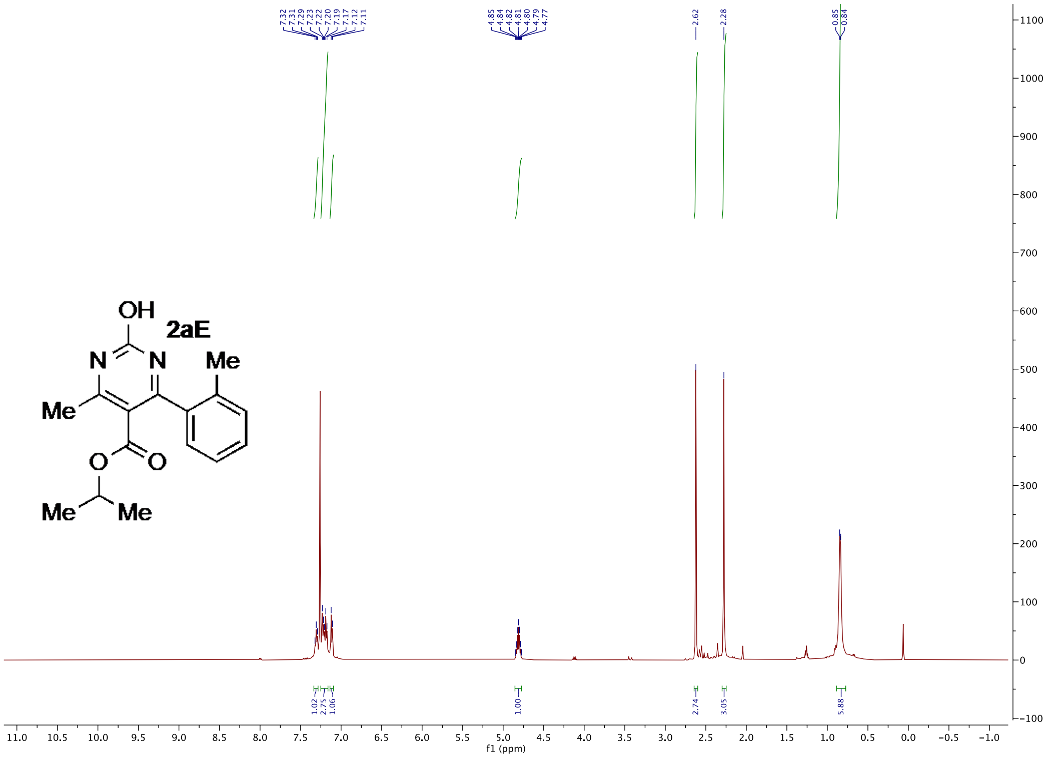
7.32 7.31 7.29 7.23 7.22 7.20 7.19 7.17 7.12 7.11
4.85 4.84 4.82 4.81 4.80 4.79 4.77
2.62
2.28
0.85 0.84
OH
2aE
N N
Me
Me
O O
Me Me
1.02 2.75 1.06
1.00
2.74
3.05
5.88
f1 (ppm)

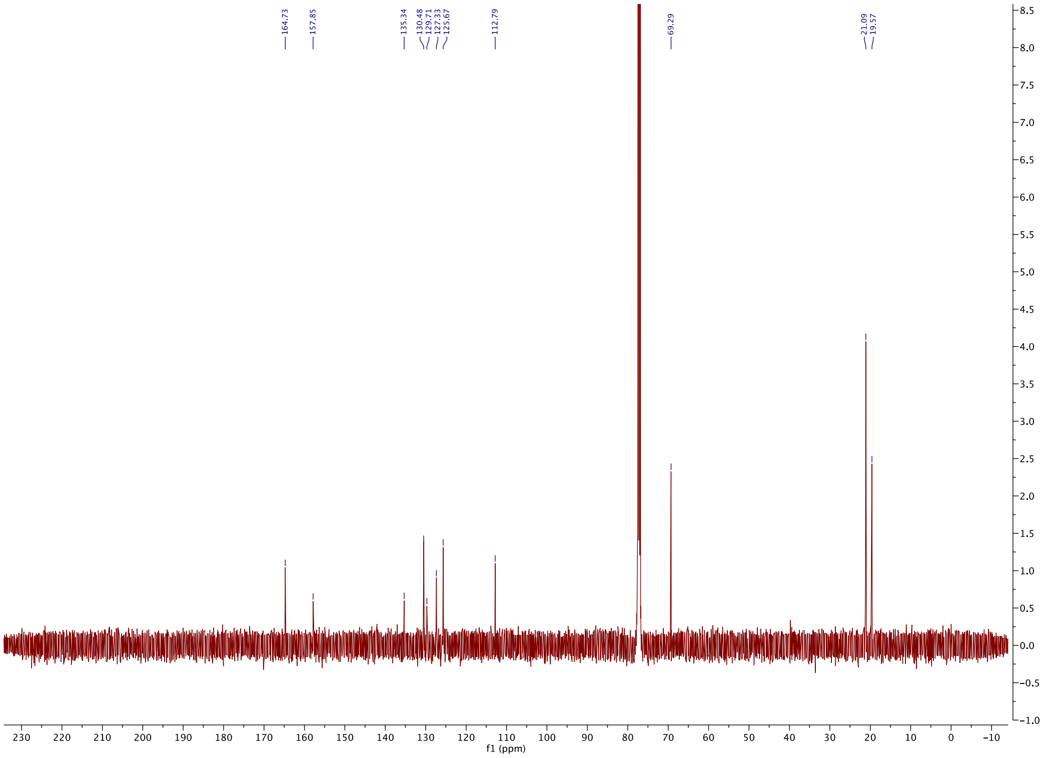
164.73
157.85
135.34 130.48 129.71 127.33 125.67
112.79
69.29
21.09 19.57
f1 (ppm)

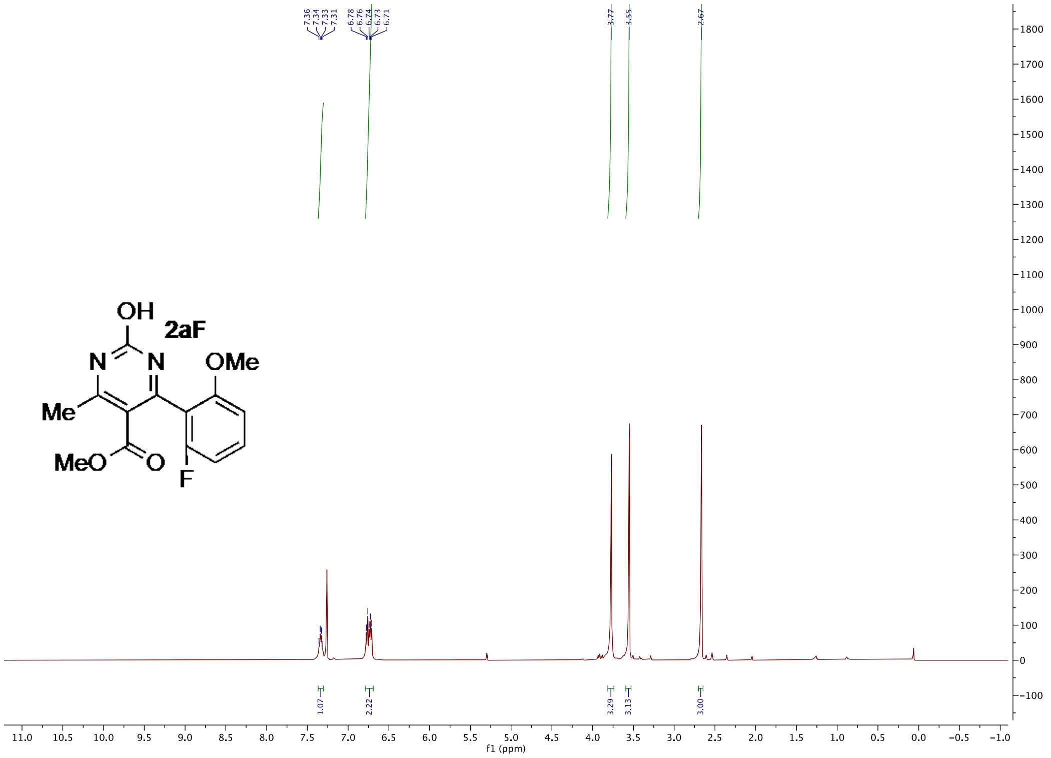
7.36
7.34
7.33
7.31
6.78
6.76
6.74
6.73
6.71
3.77
3.55
2.67
OH
2aF
N
N
OMe
Me
MeO
O
F
1.07
2.22
3.29
3.13
3.00
f1 (ppm)

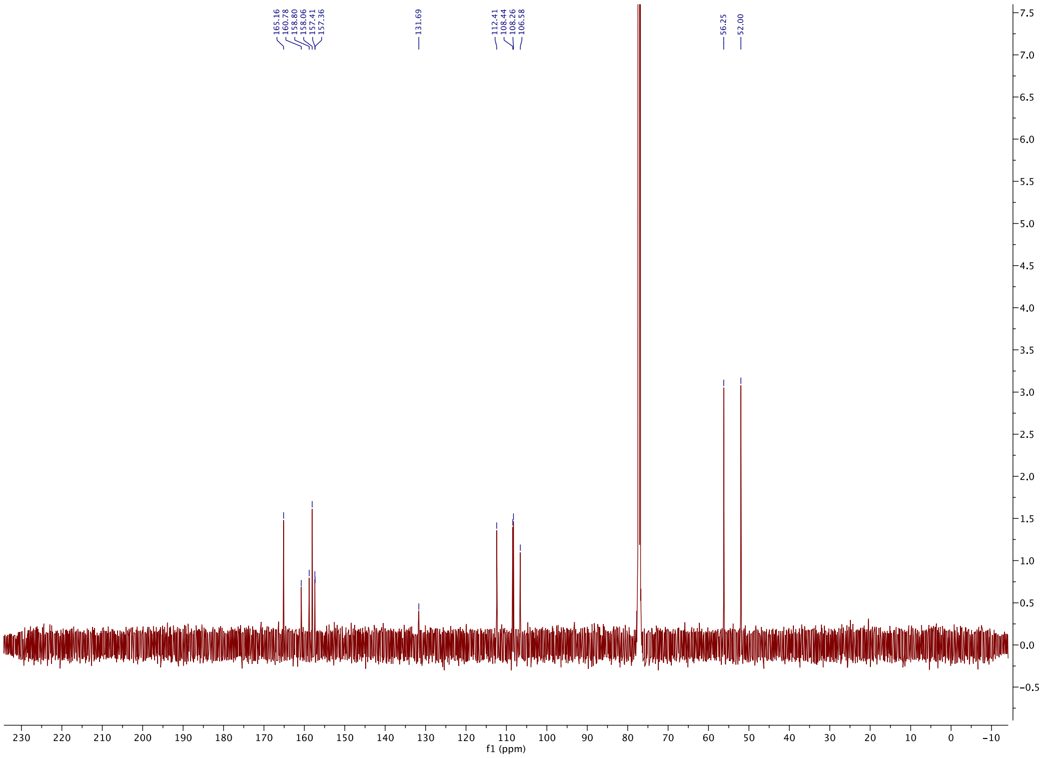
165.16
160.78
158.80
158.06
157.41
157.36
131.69
112.41
108.44
108.26
106.58
56.25
52.00
f1 (ppm)

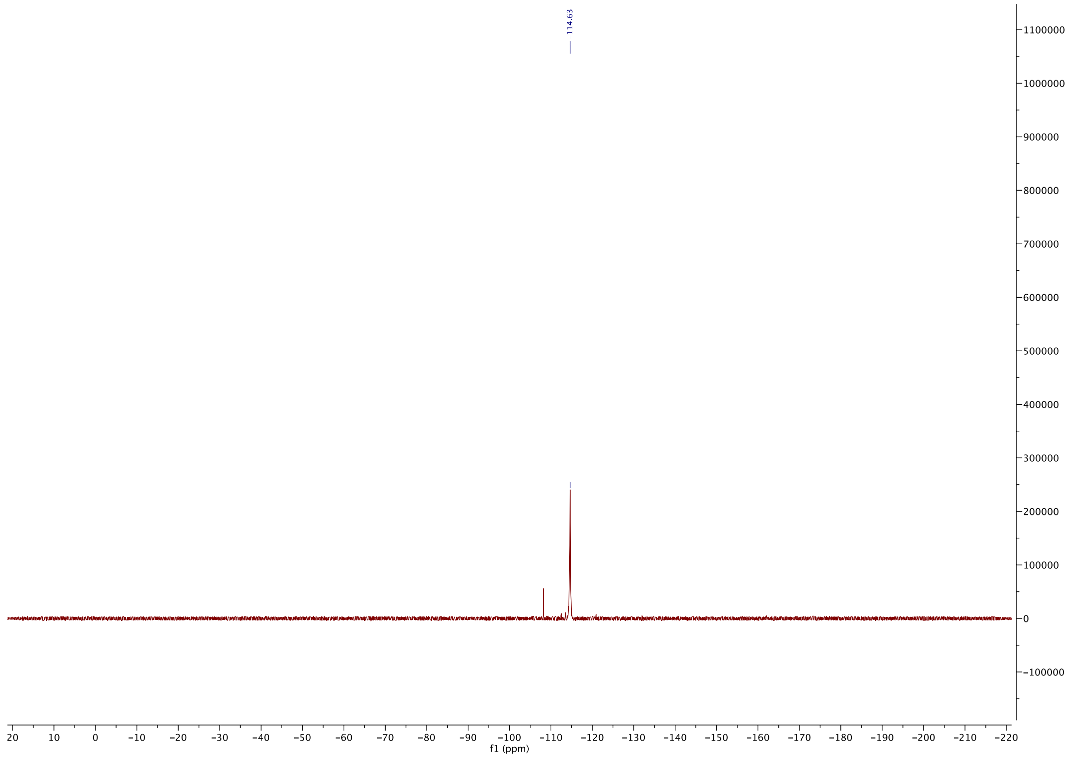

-114.63
1100000
1000000
900000
800000
700000
600000
500000
400000
300000
200000
100000
0
-100000
20 10 0 -10 -20 -30 -40 -50 -60 -70 -80 -90 -100 -110 -120 -130 -140 -150 -160 -170 -180 -190 -200 -210 -220
f1 (ppm)

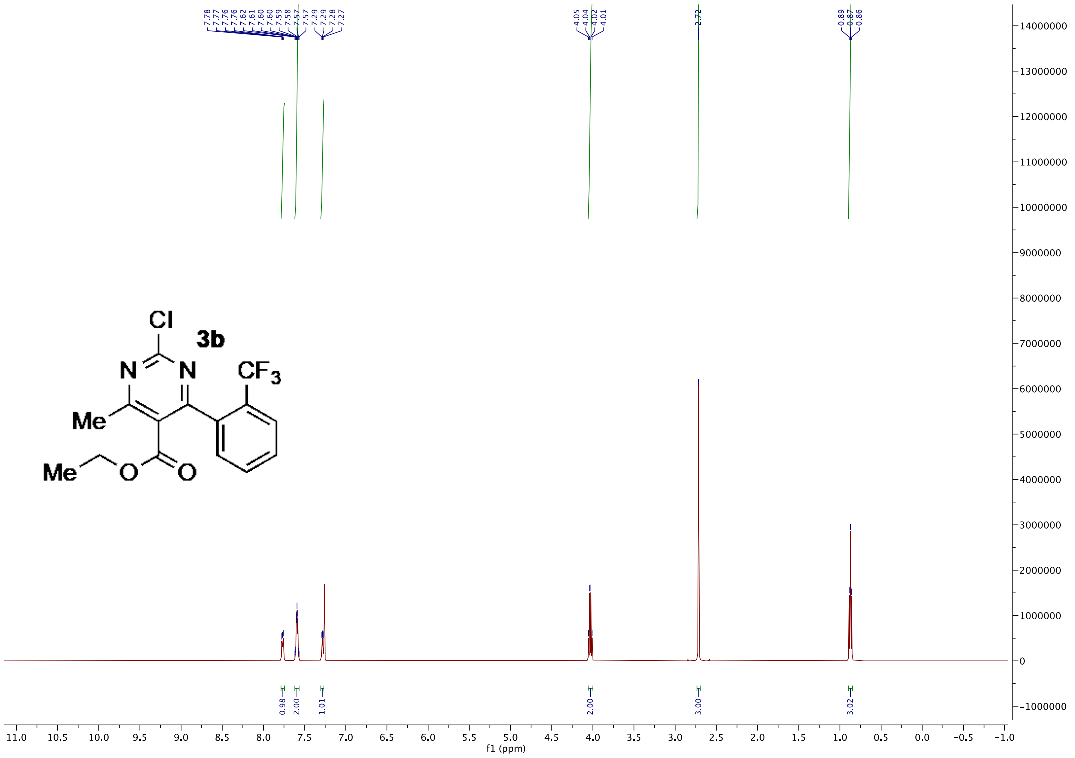
Cl
3b
N
N
CF$_3$
Me
Me
O
O
f1 (ppm)
0.98
2.00
1.01
2.00
3.00
3.02

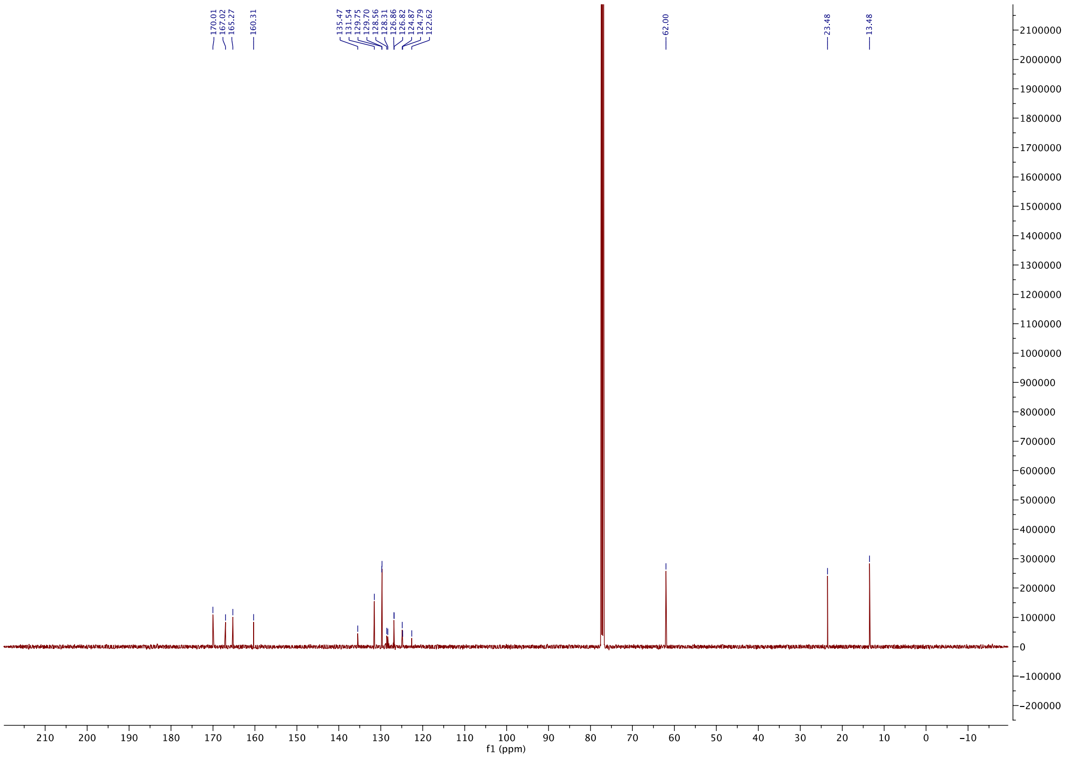
f1 (ppm)

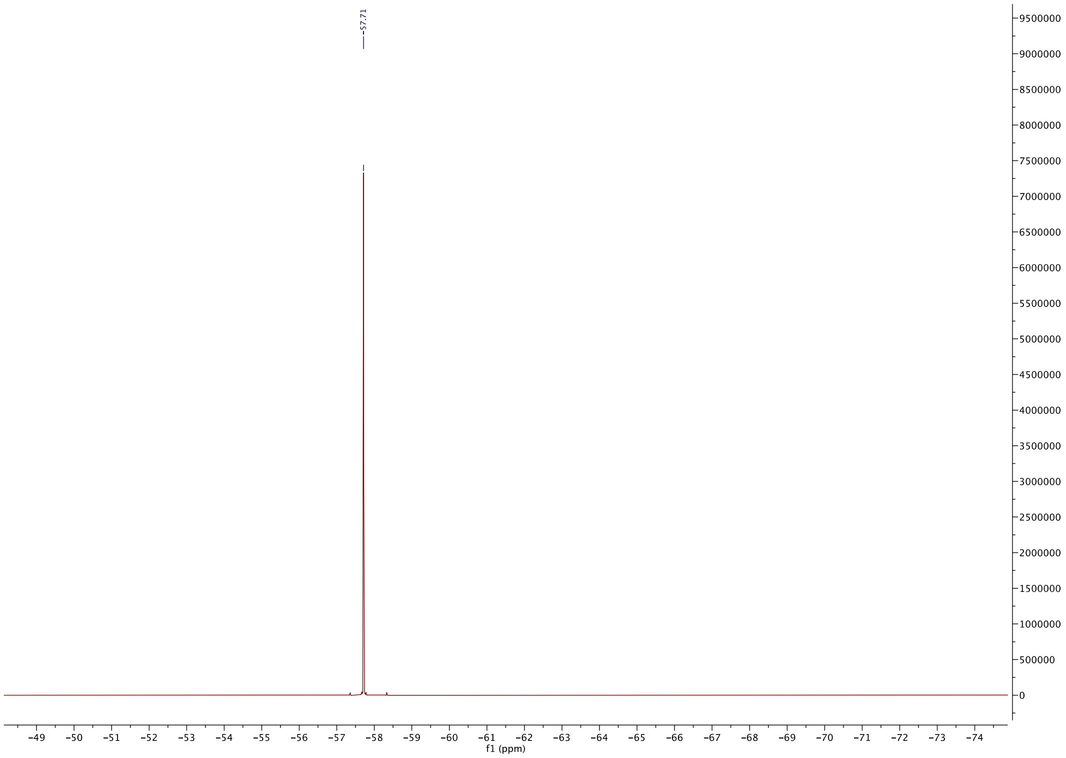
-57.71
9500000
9000000
8500000
8000000
7500000
7000000
6500000
6000000
5500000
5000000
4500000
4000000
3500000
3000000
2500000
2000000
1500000
1000000
500000
0
-49 -50 -51 -52 -53 -54 -55 -56 -57 -58 -59 -60 -61 -62 -63 -64 -65 -66 -67 -68 -69 -70 -71 -72 -73 -74
f1 (ppm)

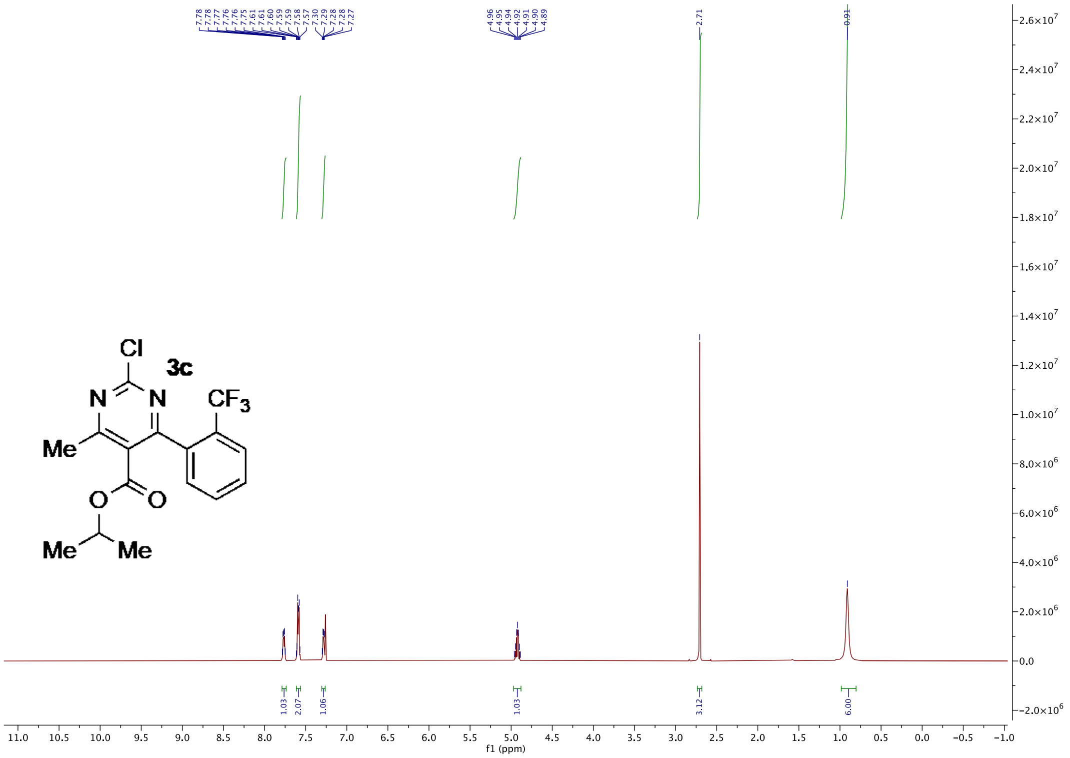
3c
f1 (ppm)

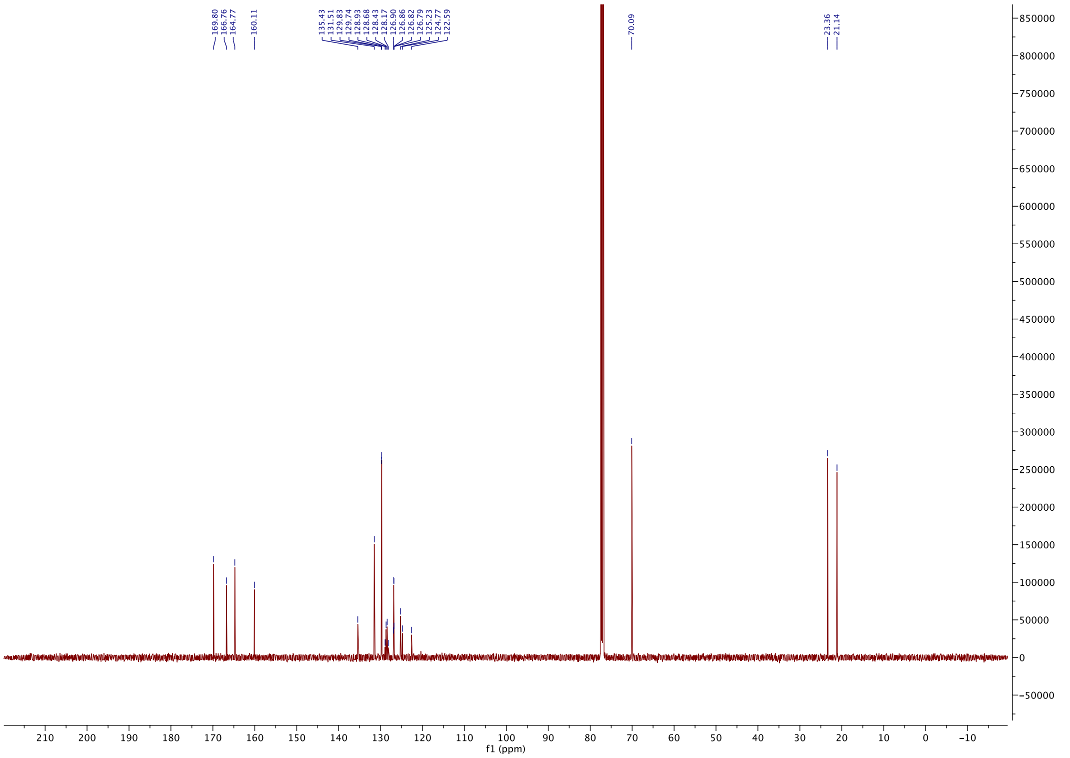
f1 (ppm)

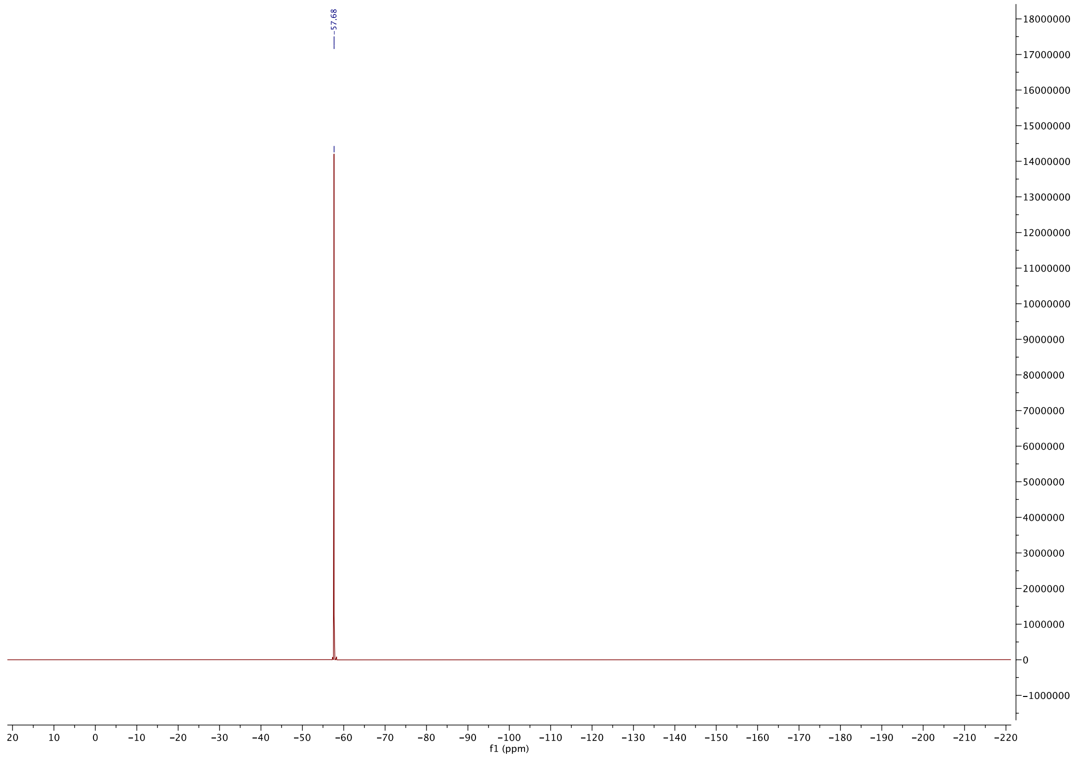
-57.68
f1 (ppm)

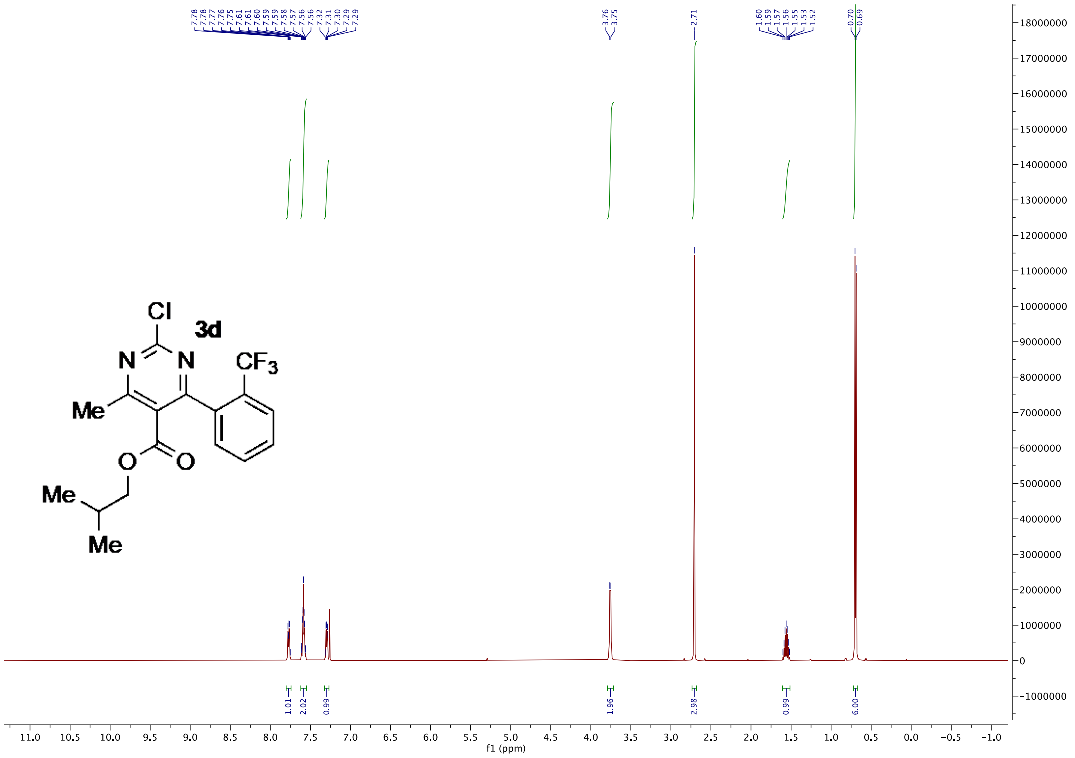

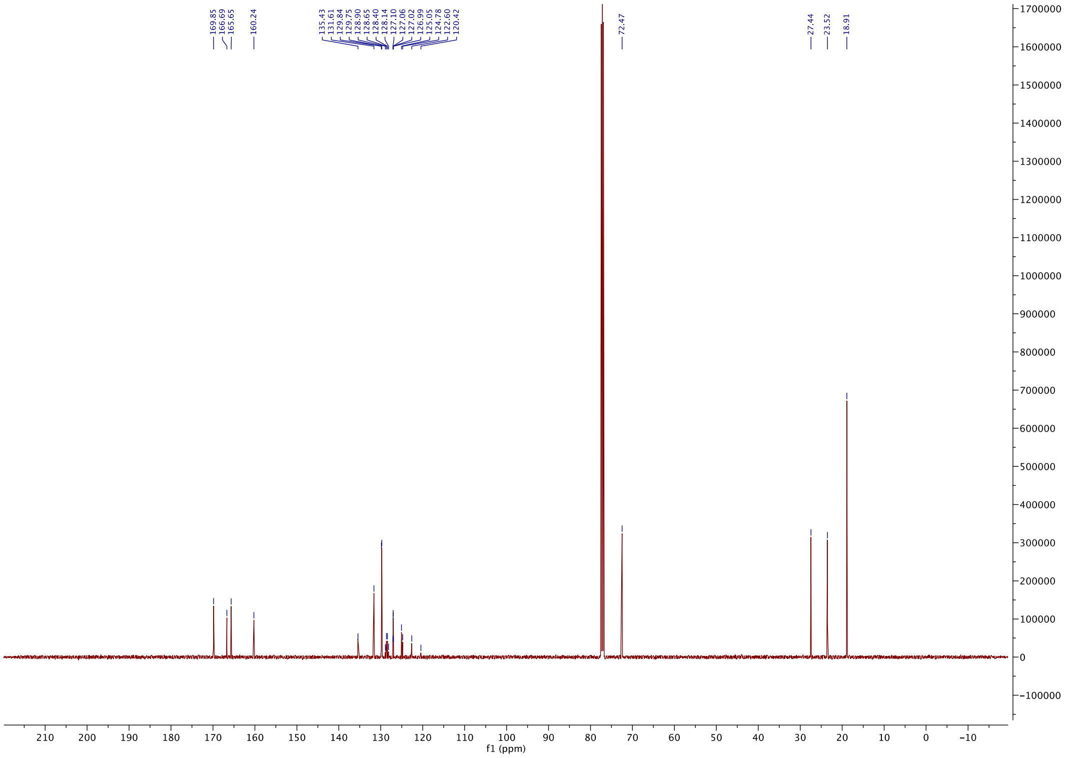

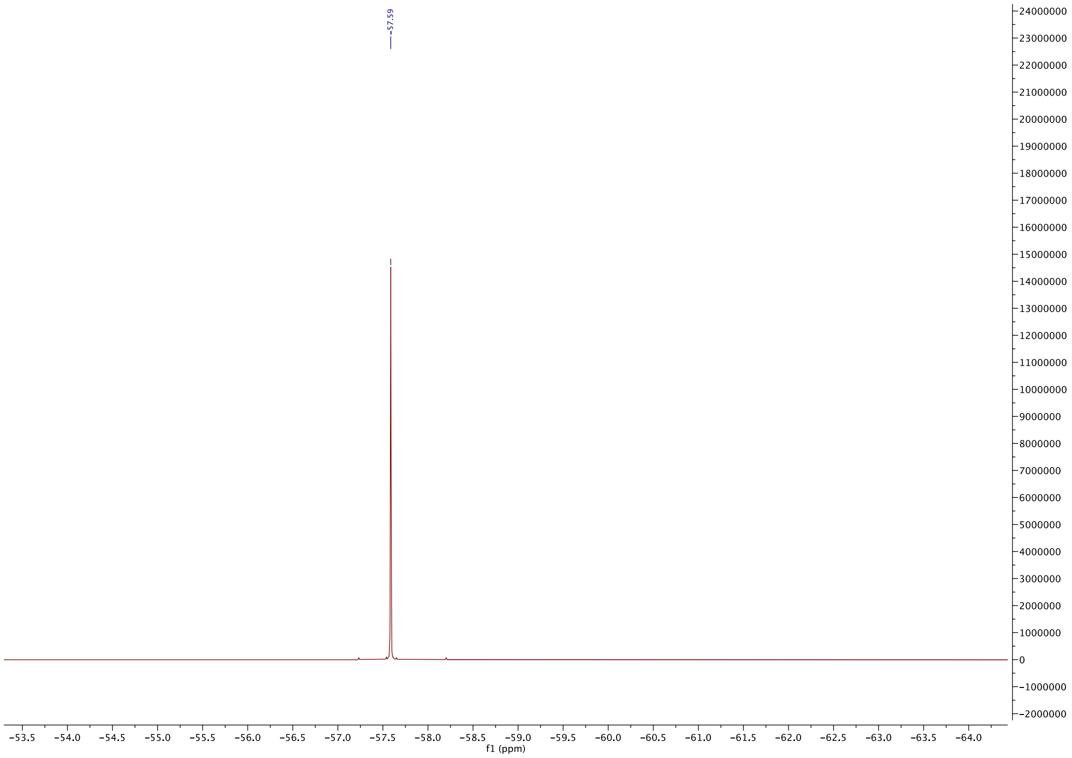
-57.59
f1 (ppm)

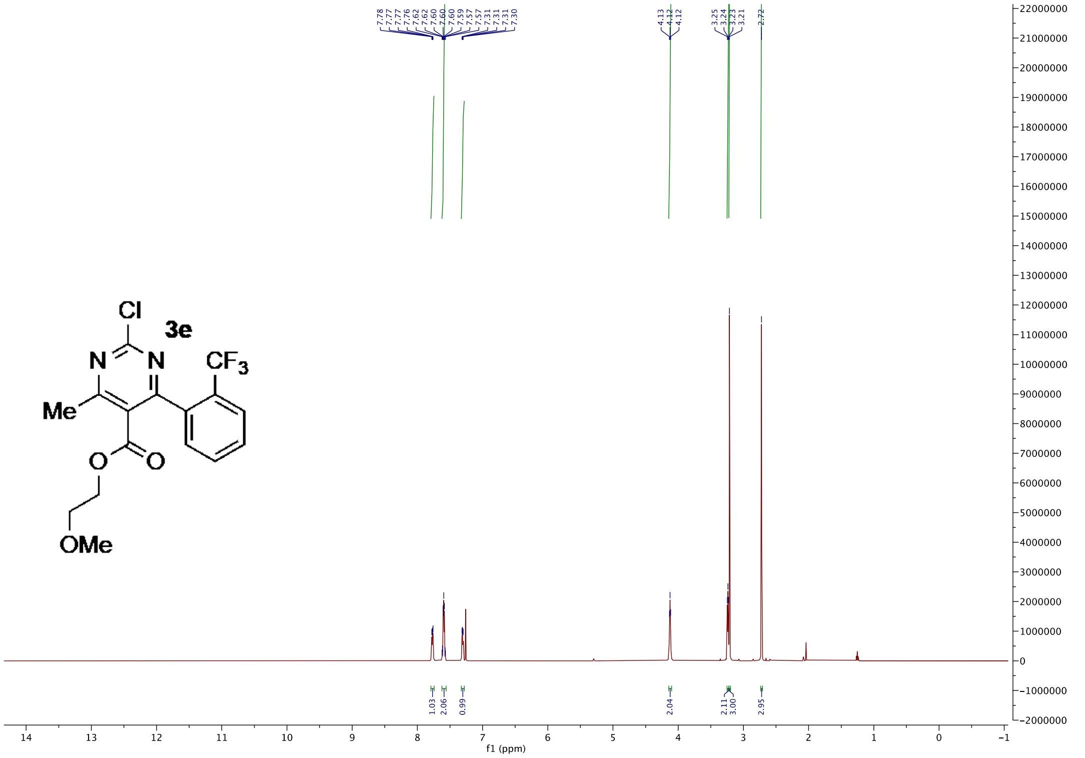

3e

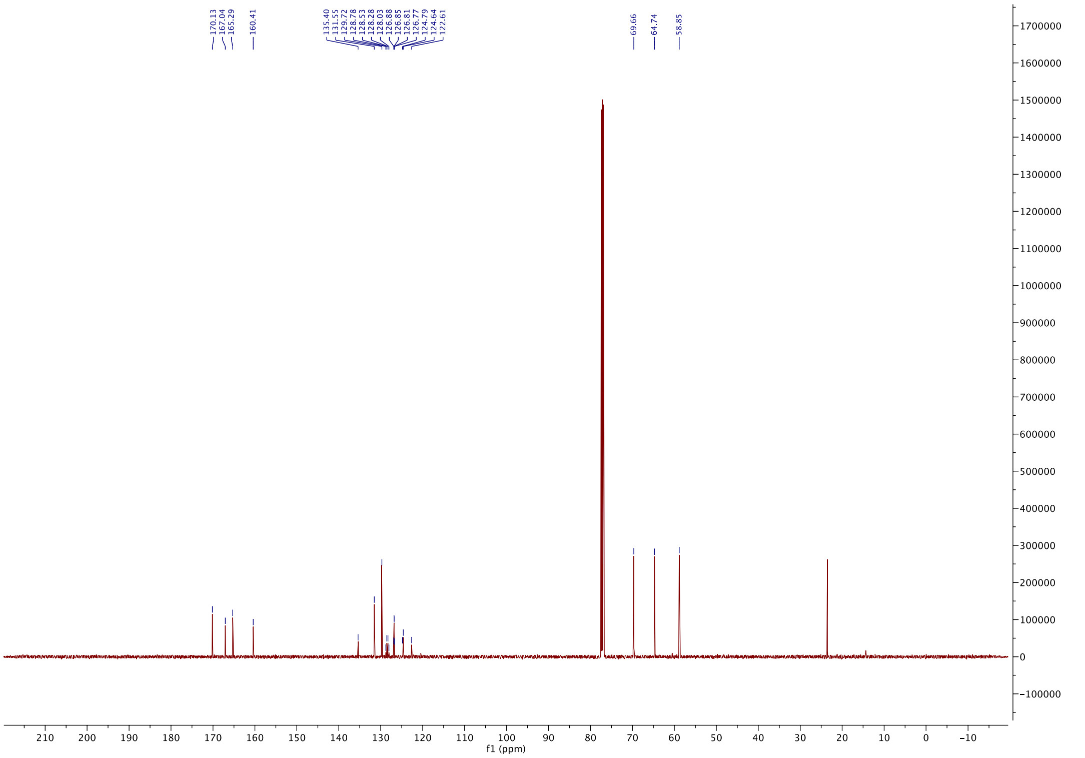

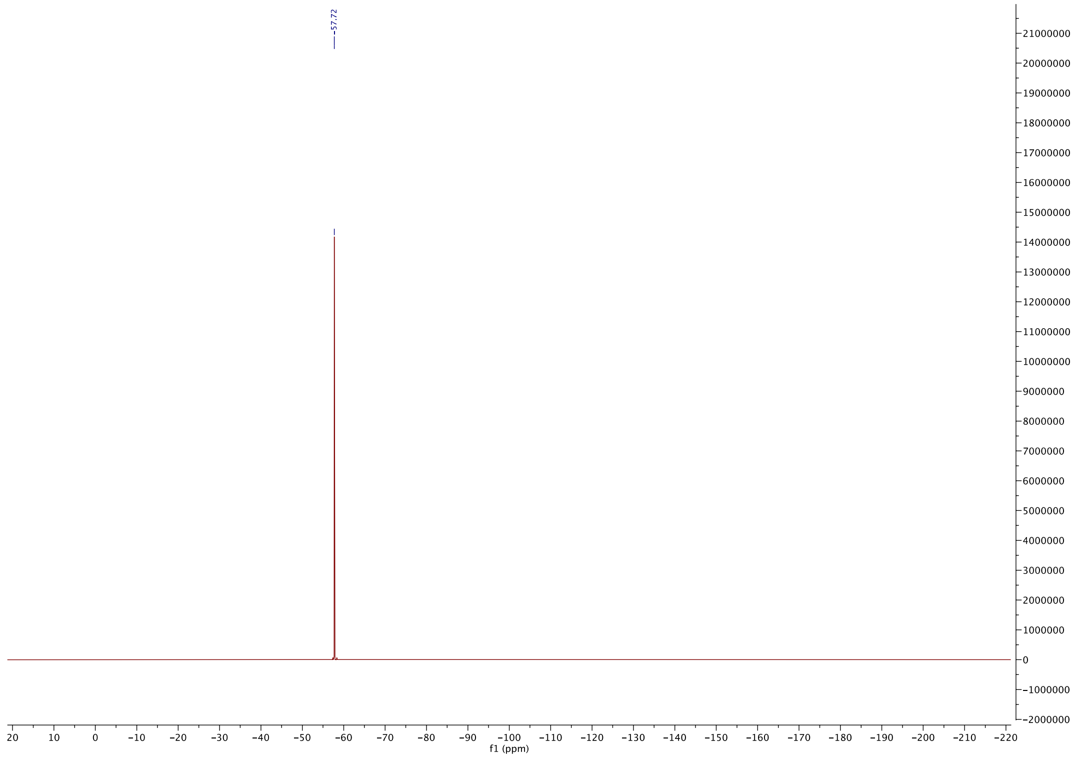

-57.72
21000000
20000000
19000000
18000000
17000000
16000000
15000000
14000000
13000000
12000000
11000000
10000000
9000000
8000000
7000000
6000000
5000000
4000000
3000000
2000000
1000000
0
-1000000
-2000000
20 10 0 -10 -20 -30 -40 -50 -60 -70 -80 -90 -100 -110 -120 -130 -140 -150 -160 -170 -180 -190 -200 -210 -220
f1 (ppm)

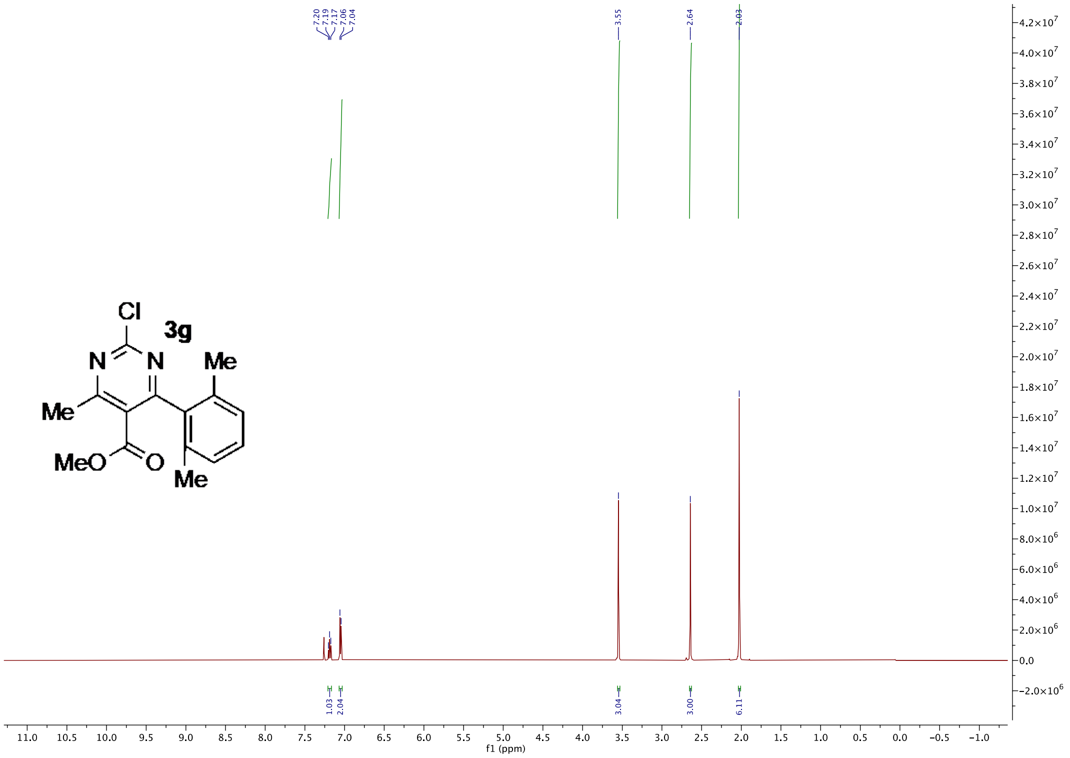

Cl
3g
N
N
Me
Me
MeO
O
Me
7.20
7.19
7.17
7.06
7.04
3.55
2.64
2.03
1.03
2.04
3.04
3.00
6.11
f1 (ppm)

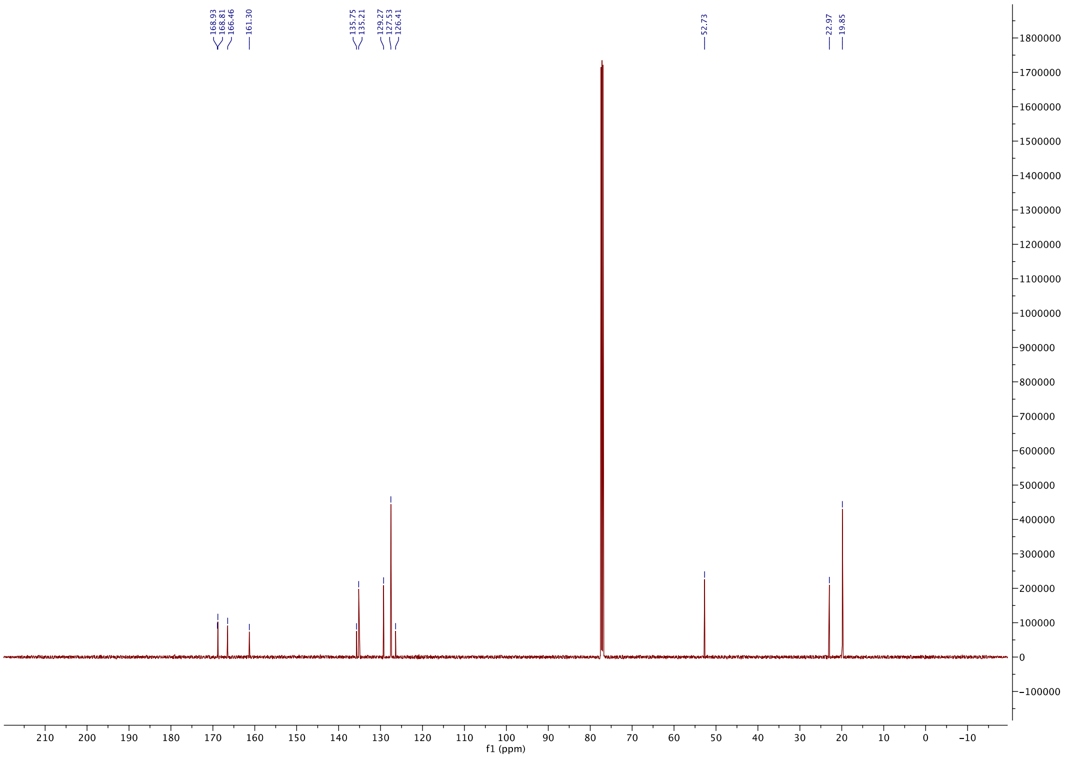

168.93
168.81
166.46
161.30
135.75
135.21
129.27
127.53
126.41
52.73
22.97
19.85
f1 (ppm)

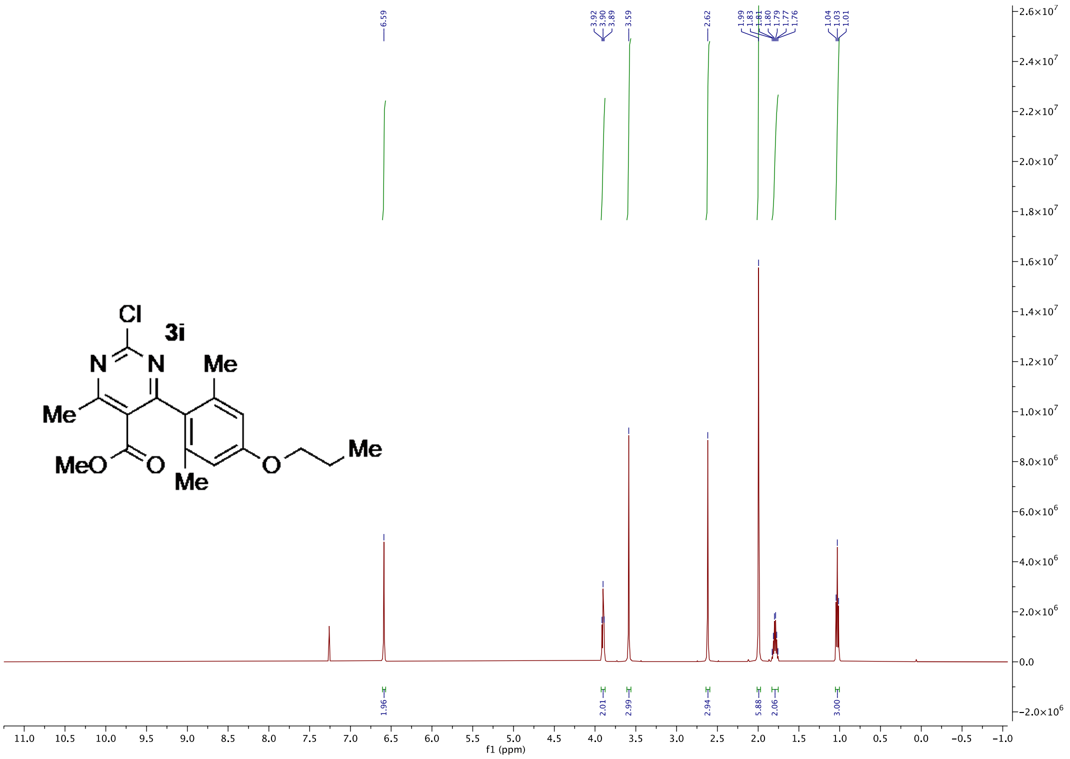
6.59
3.92 3.90 3.89
3.59
2.62
1.99 1.83 1.81 1.80 1.79 1.77 1.76
1.04 1.03 1.01
1.96
2.01
2.99
2.94
5.88
2.06
3.00
Cl
N
N
3i
Me
Me
MeO
O
O
Me
Me
f1 (ppm)

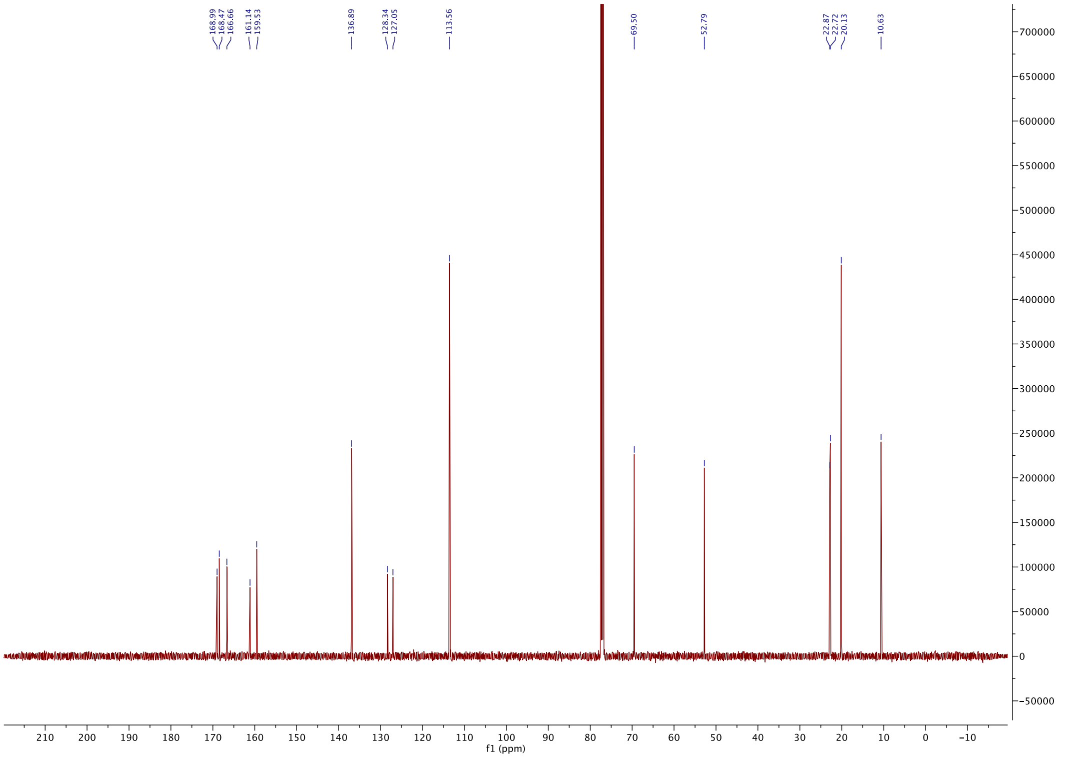
168.99 168.47 166.66
161.14 159.53
136.89
128.34 127.05
113.56
69.50
52.79
22.87 22.72 20.13
10.63
f1 (ppm)

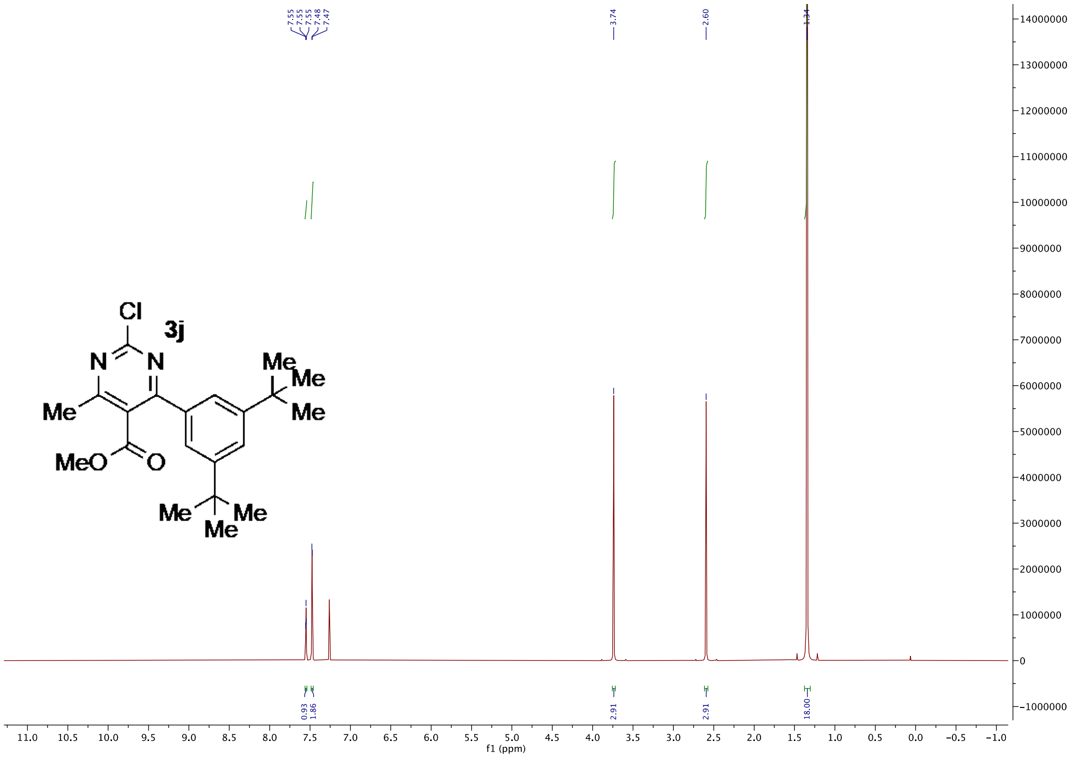

Cl
3j
N
N
Me
Me
Me
Me
MeO
O
Me
Me
Me
7.55
7.55
7.55
7.48
7.47
3.74
2.60
1.34
0.93
1.86
2.91
2.91
18.00
f1 (ppm)

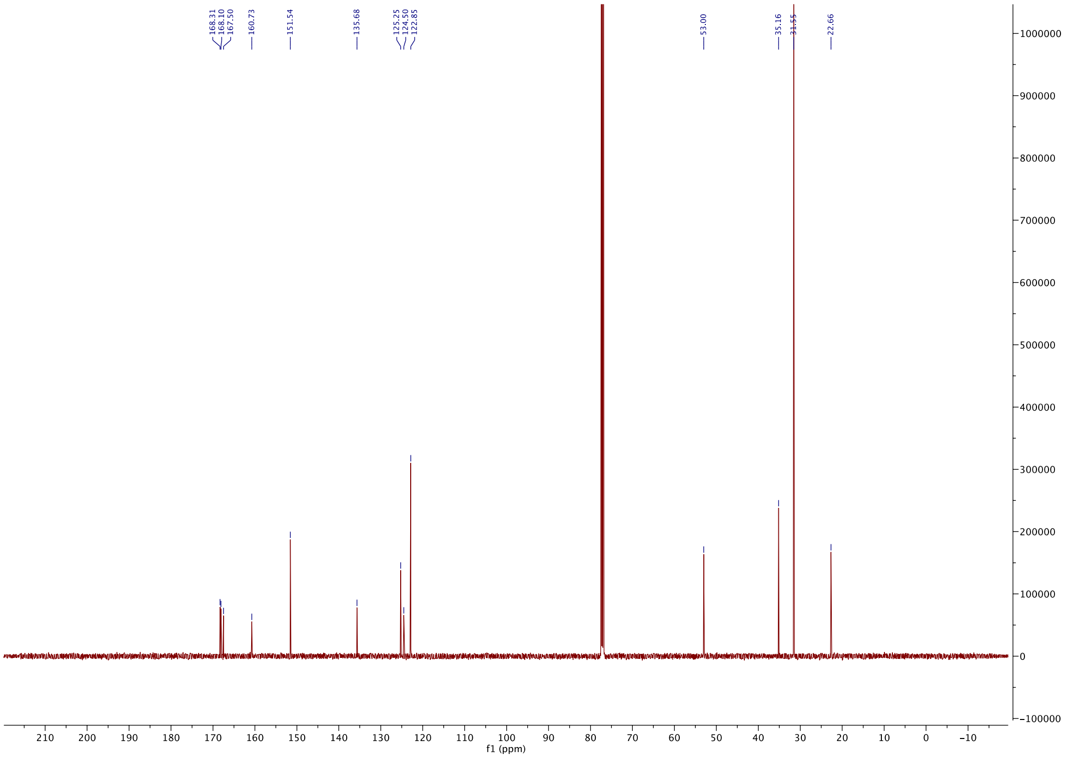

168.31
168.10
167.50
160.73
151.54
135.68
125.25
124.50
122.85
53.00
35.16
31.55
22.66
f1 (ppm)

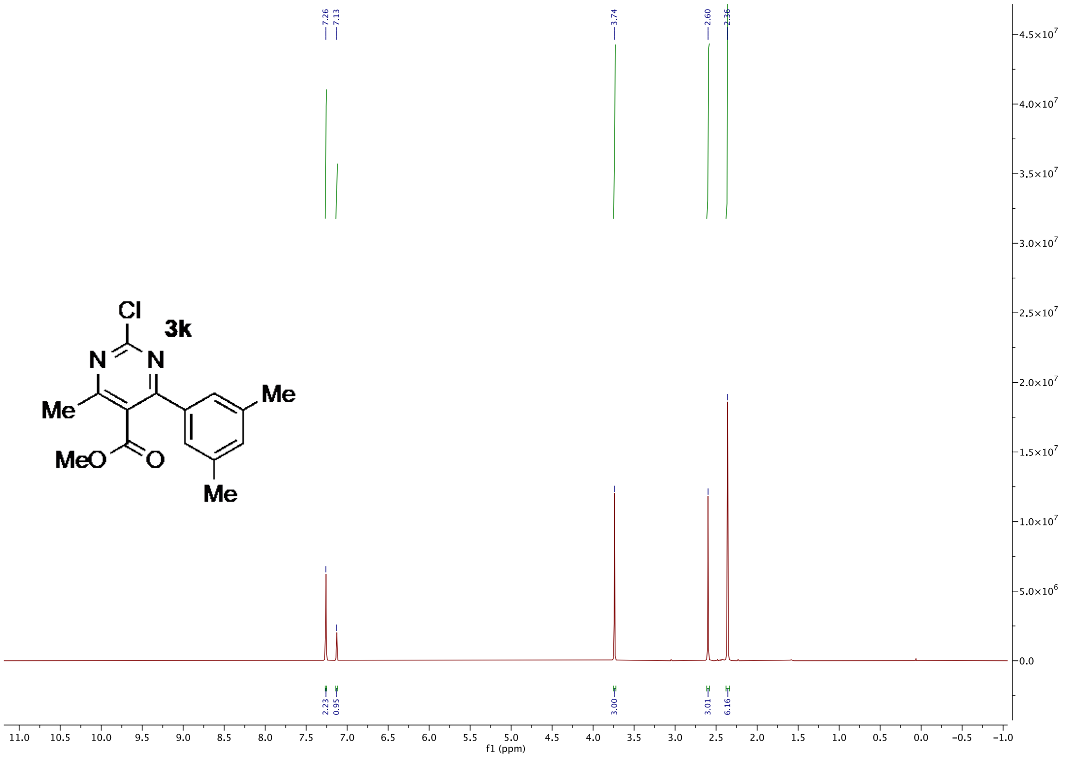

7.26
7.13
3.74
2.60
2.36
2.23
0.95
3.00
3.01
6.16
3k
Cl
N
N
Me
Me
MeO
O
Me
f1 (ppm)

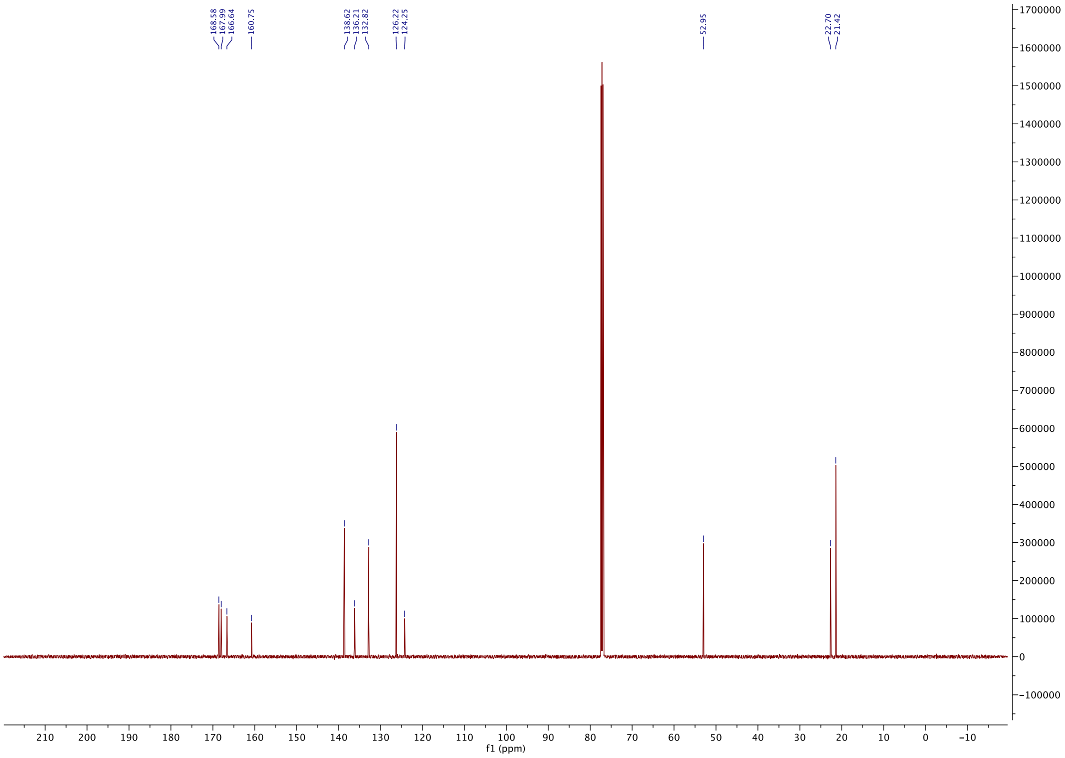

168.58
167.99
166.64
160.75
138.62
136.21
132.82
126.22
124.25
52.95
22.70
21.42
f1 (ppm)

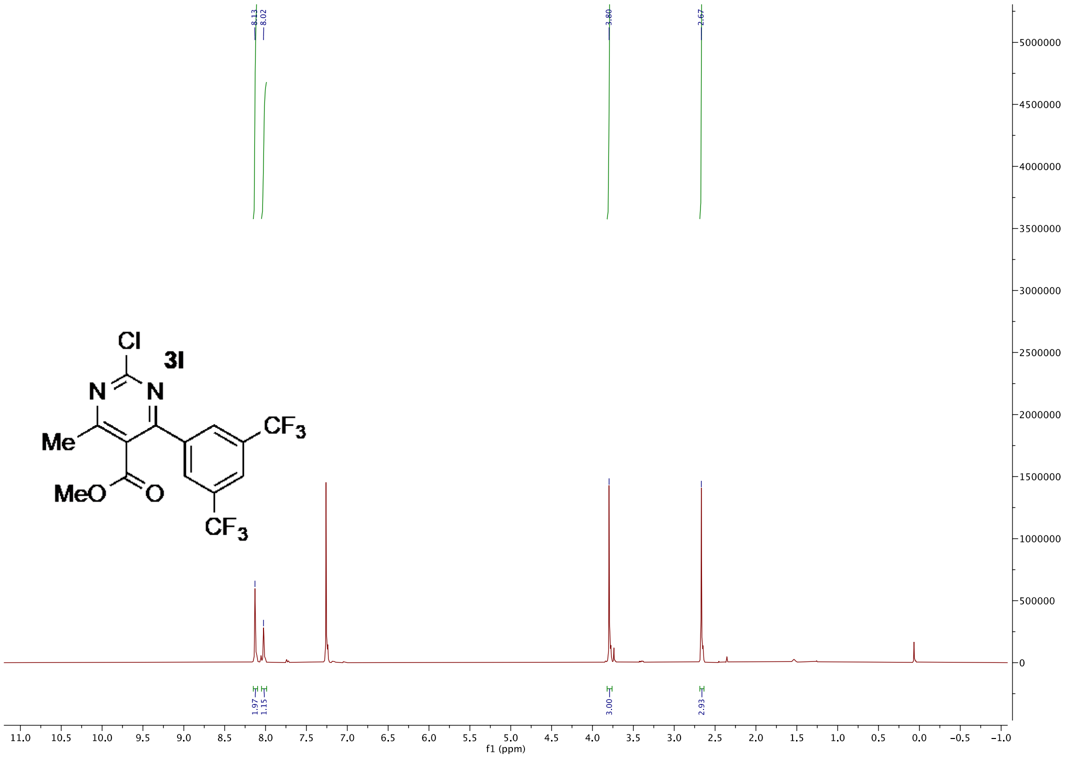
8.12
8.02
3.80
2.67
1.97
1.15
3.00
2.93
Cl
N
N
Me
MeO
O
CF3
CF3
3l
f1 (ppm)

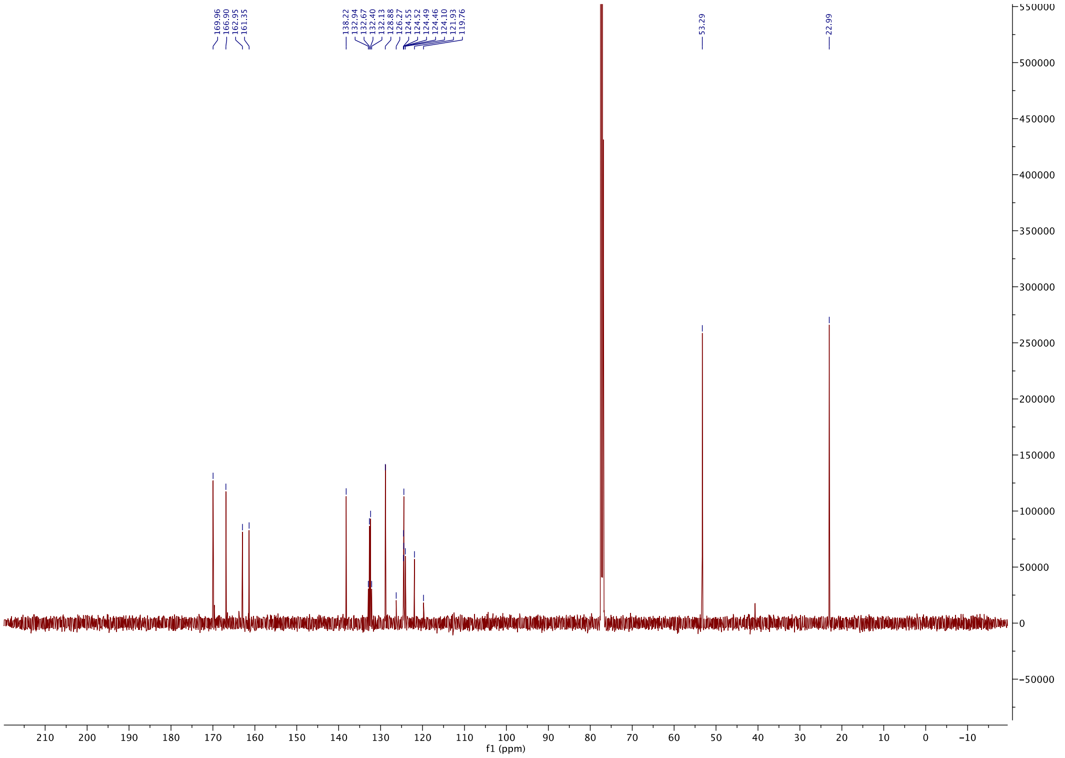
169.96
166.90
162.95
161.35
138.22
132.94
132.67
132.40
132.13
128.88
126.27
124.55
124.52
124.49
124.46
124.10
121.93
119.76
53.29
22.99
f1 (ppm)

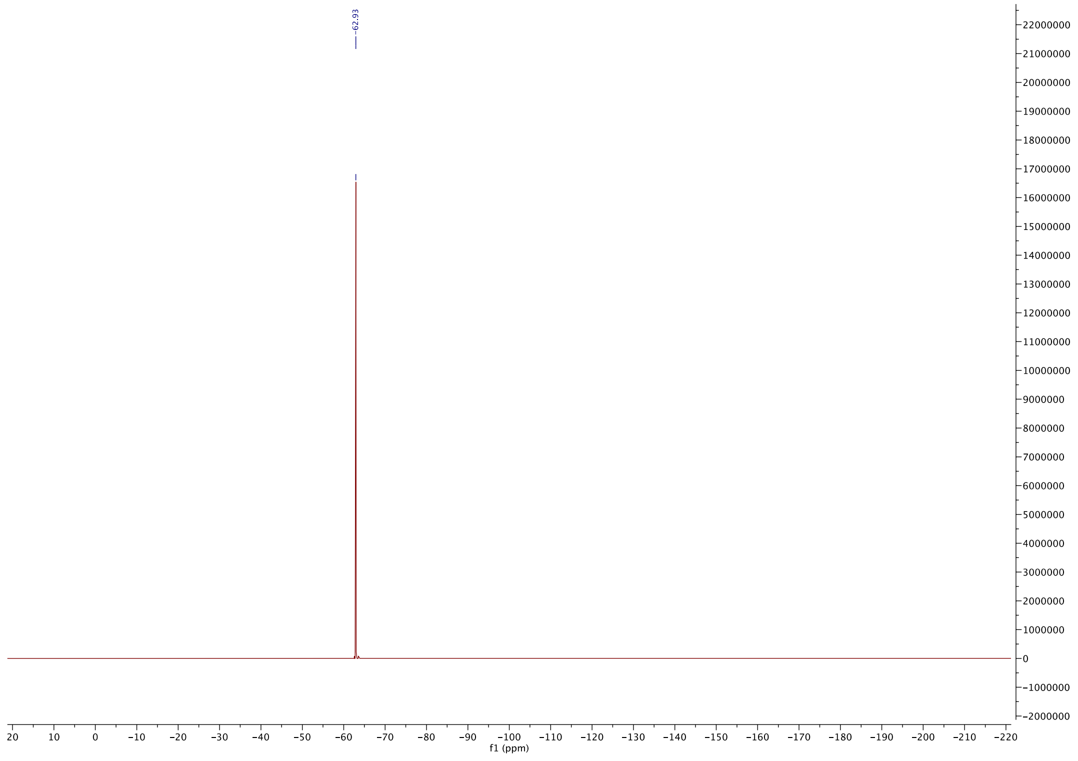

-62.93
22000000
21000000
20000000
19000000
18000000
17000000
16000000
15000000
14000000
13000000
12000000
11000000
10000000
9000000
8000000
7000000
6000000
5000000
4000000
3000000
2000000
1000000
0
-1000000
-2000000
20 10 0 -10 -20 -30 -40 -50 -60 -70 -80 -90 -100 -110 -120 -130 -140 -150 -160 -170 -180 -190 -200 -210 -220
f1 (ppm)

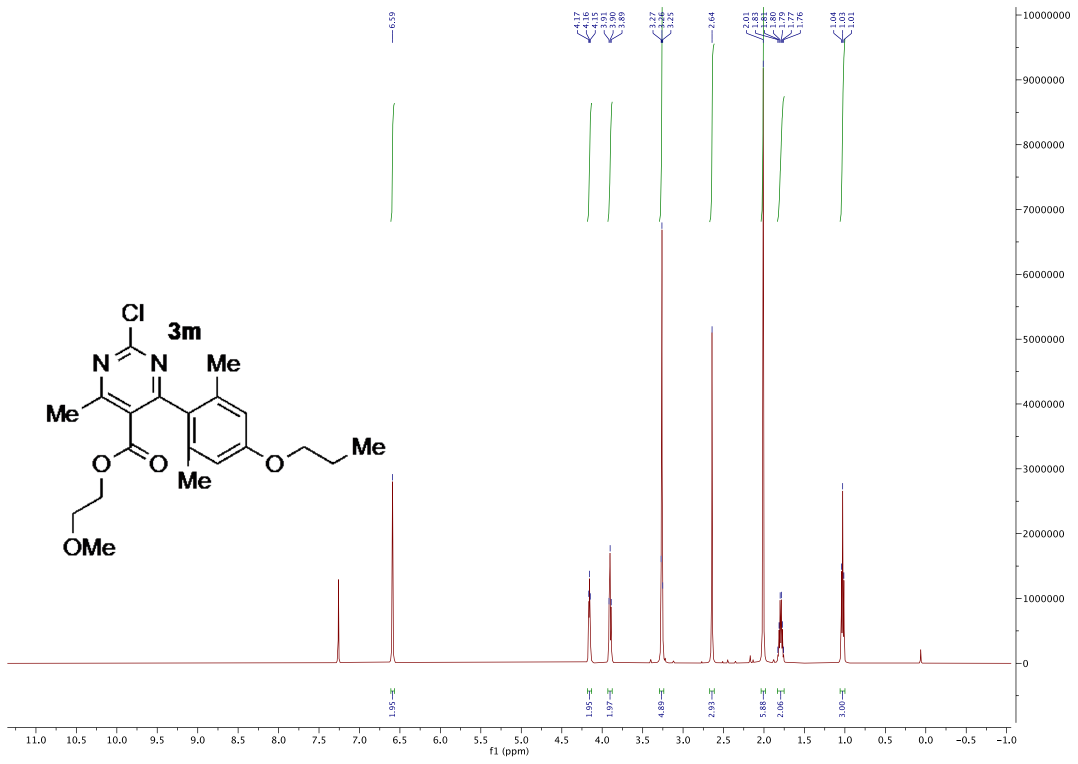
3m
Cl
N
N
Me
Me
O
O
Me
Me
OMe
O
6.59
4.17
4.16
4.15
3.91
3.90
3.89
3.27
3.26
3.25
2.64
2.01
1.83
1.81
1.80
1.79
1.77
1.76
1.04
1.03
1.01
1.95
1.95
1.97
4.89
2.93
5.88
2.06
3.00
f1 (ppm)

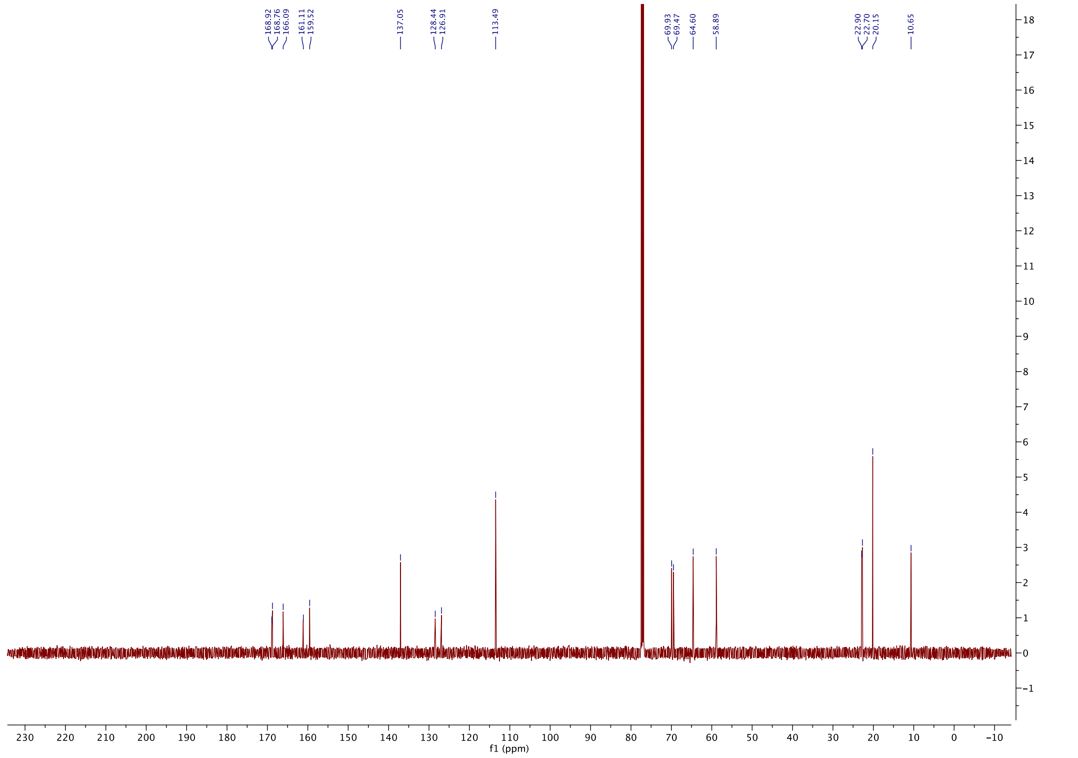
168.92
168.76
166.09
161.11
159.52
137.05
128.44
126.91
113.49
69.93
69.47
64.60
58.89
22.90
22.70
20.15
10.65
f1 (ppm)

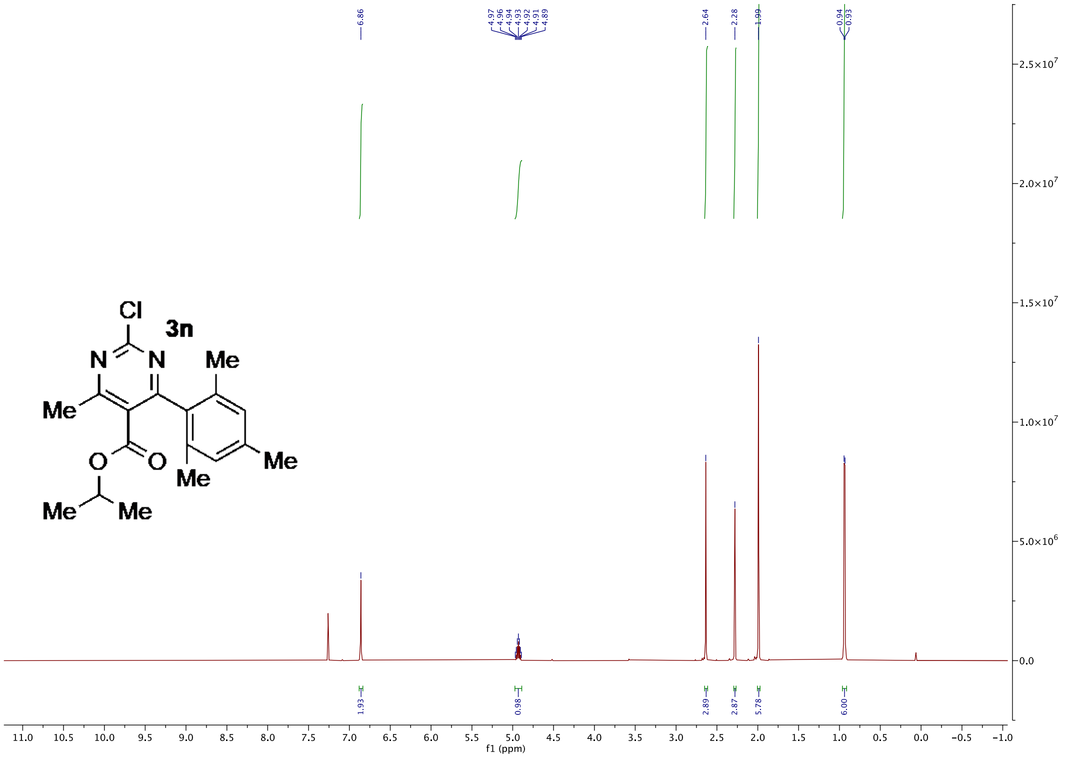
6.86
4.97
4.96
4.94
4.93
4.92
4.91
4.89
2.64
2.28
1.99
0.94
0.93
1.93
0.98
2.89
2.87
5.78
6.00
Cl
N
N
3n
Me
Me
O
O
Me
Me
Me
Me
f1 (ppm)

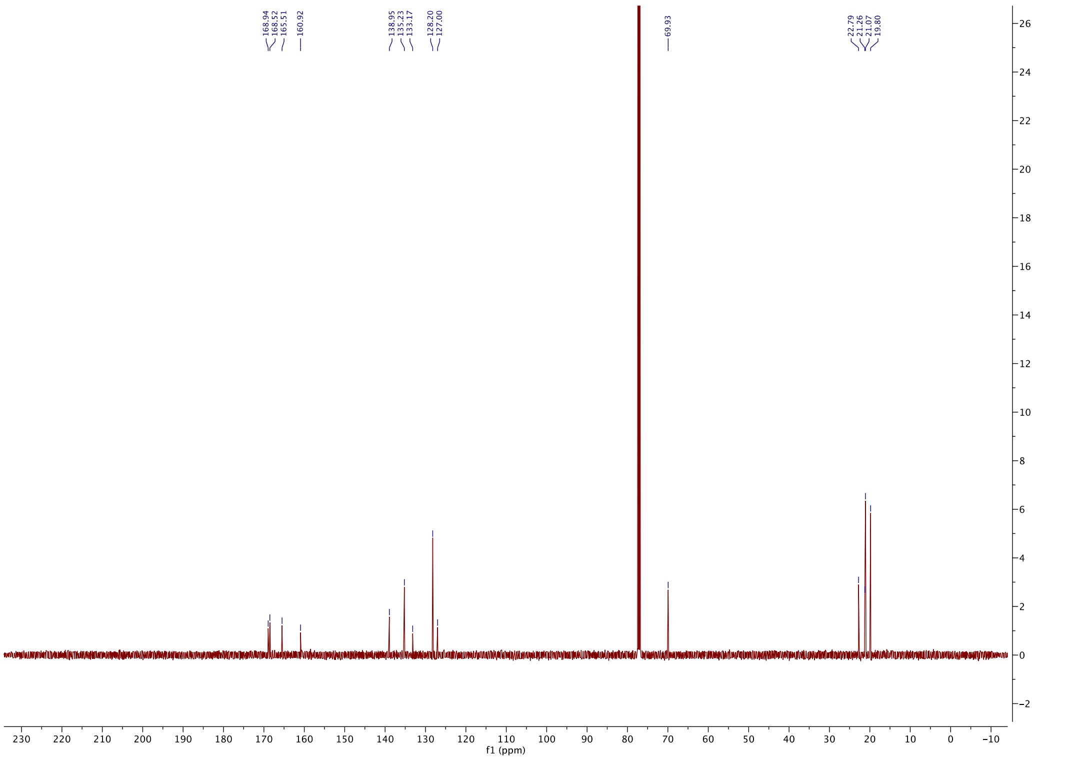
168.94
168.52
165.51
160.92
138.95
135.23
133.17
128.20
127.00
69.93
22.79
21.26
21.07
19.80
f1 (ppm)

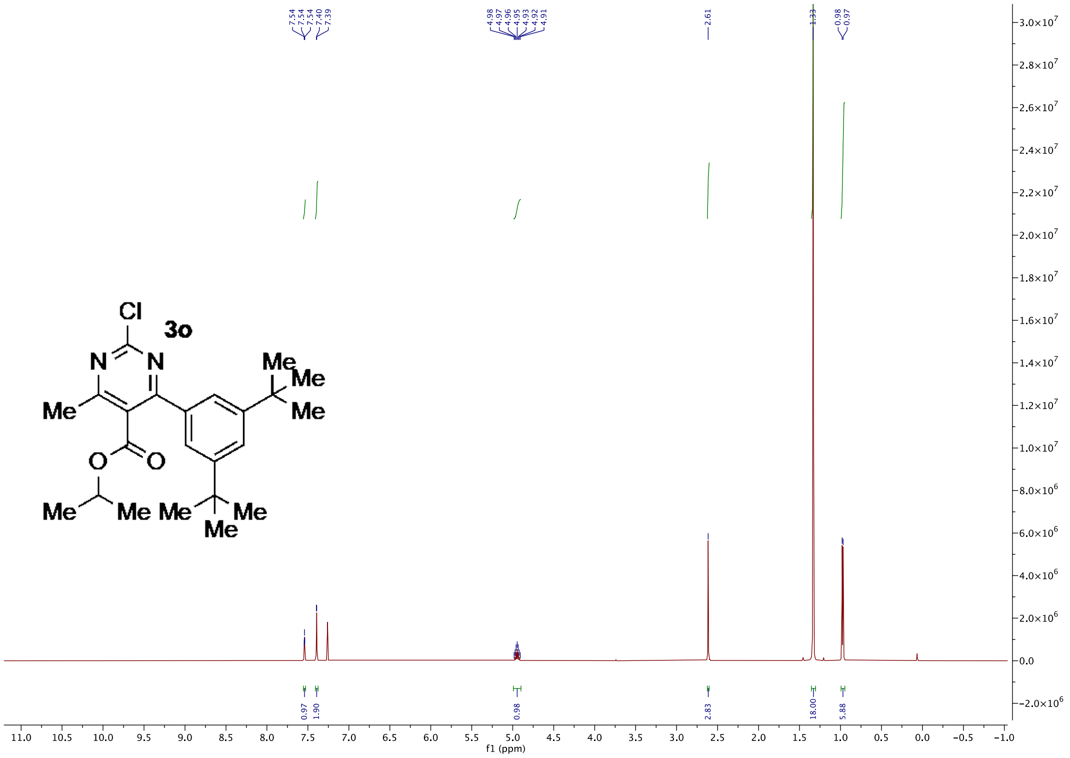
7.54
7.54
7.54
7.40
7.39
4.98
4.97
4.96
4.95
4.93
4.92
4.91
2.61
1.33
0.98
0.97
Cl
3o
N
N
Me
Me
Me
Me
O
O
Me
Me
Me
Me
Me
0.97
1.90
0.98
2.83
18.00
5.88
f1 (ppm)

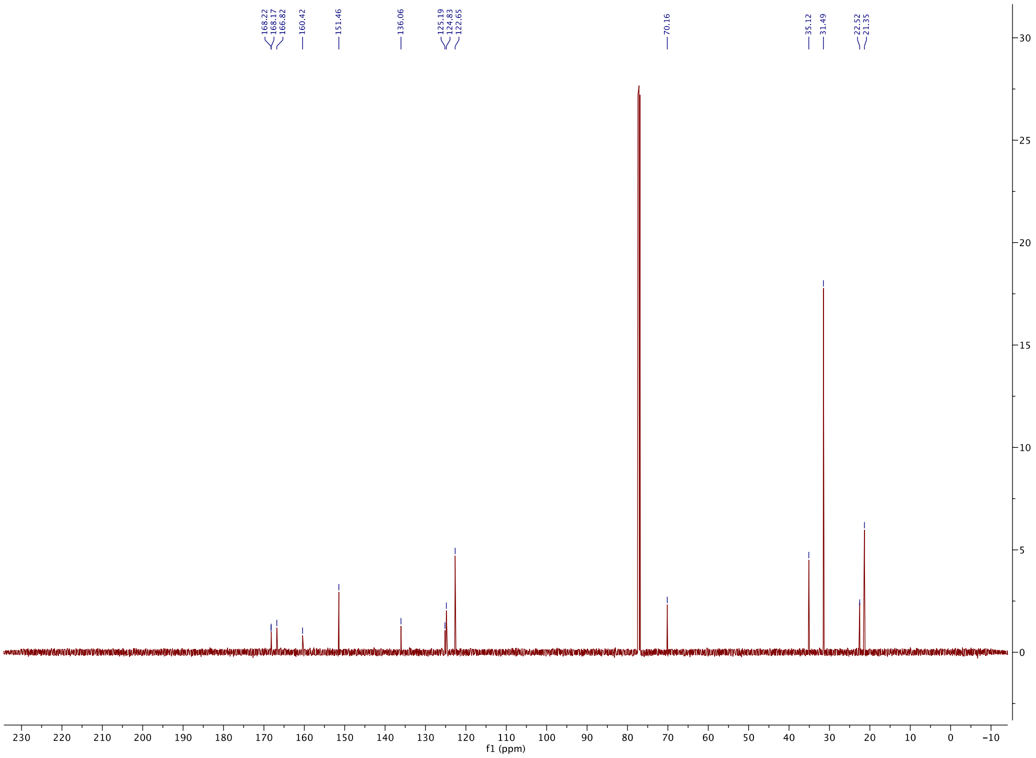
168.22
168.17
166.82
160.42
151.46
136.06
125.19
124.83
122.65
70.16
35.12
31.49
22.52
21.35
f1 (ppm)

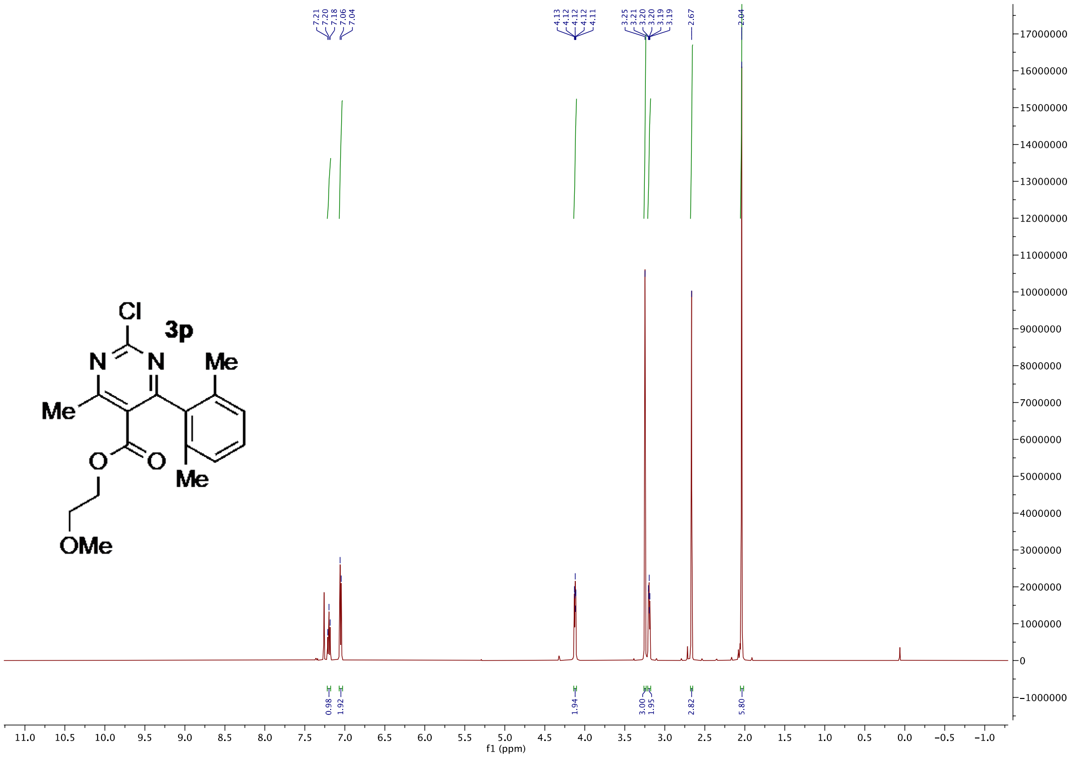
3p
f1 (ppm)

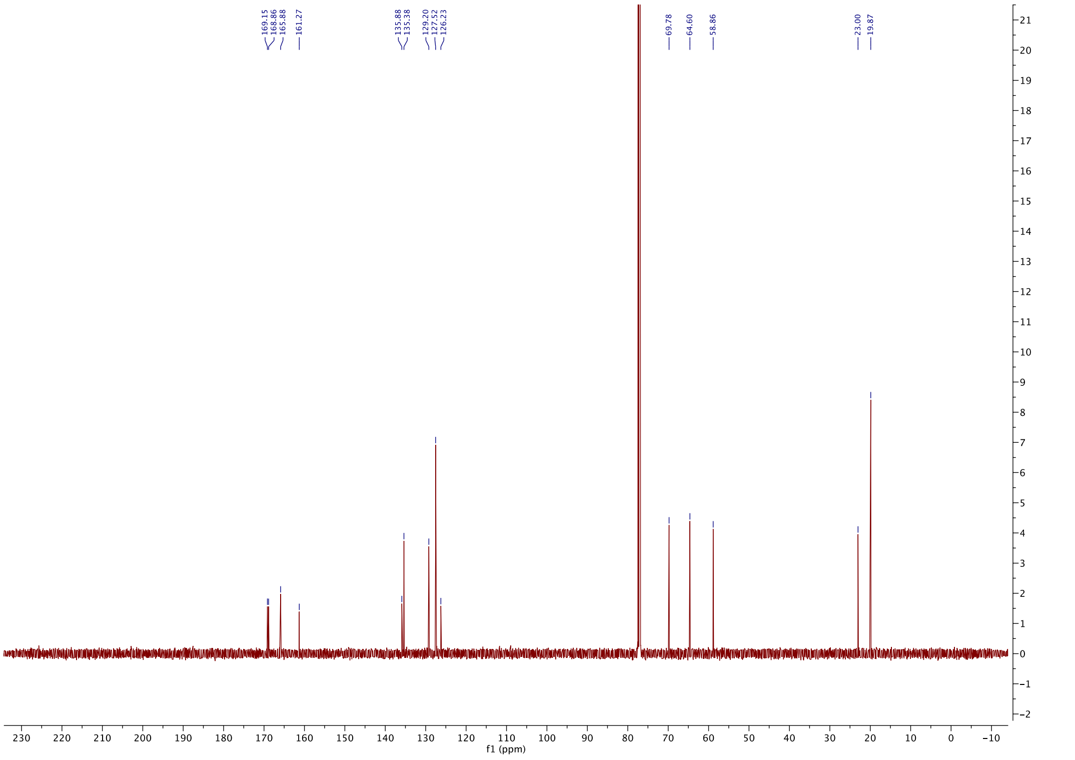
f1 (ppm)

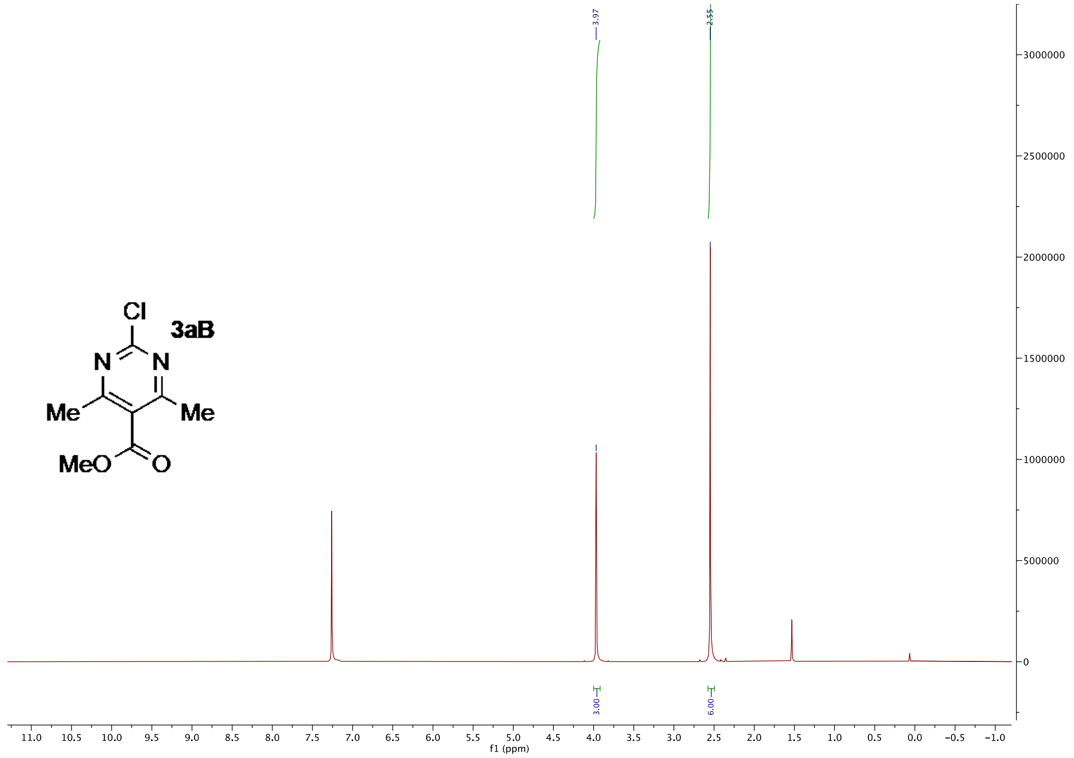
3aB
Cl
N
N
Me
Me
MeO
O
3.97
2.55
3.00
6.00
f1 (ppm)

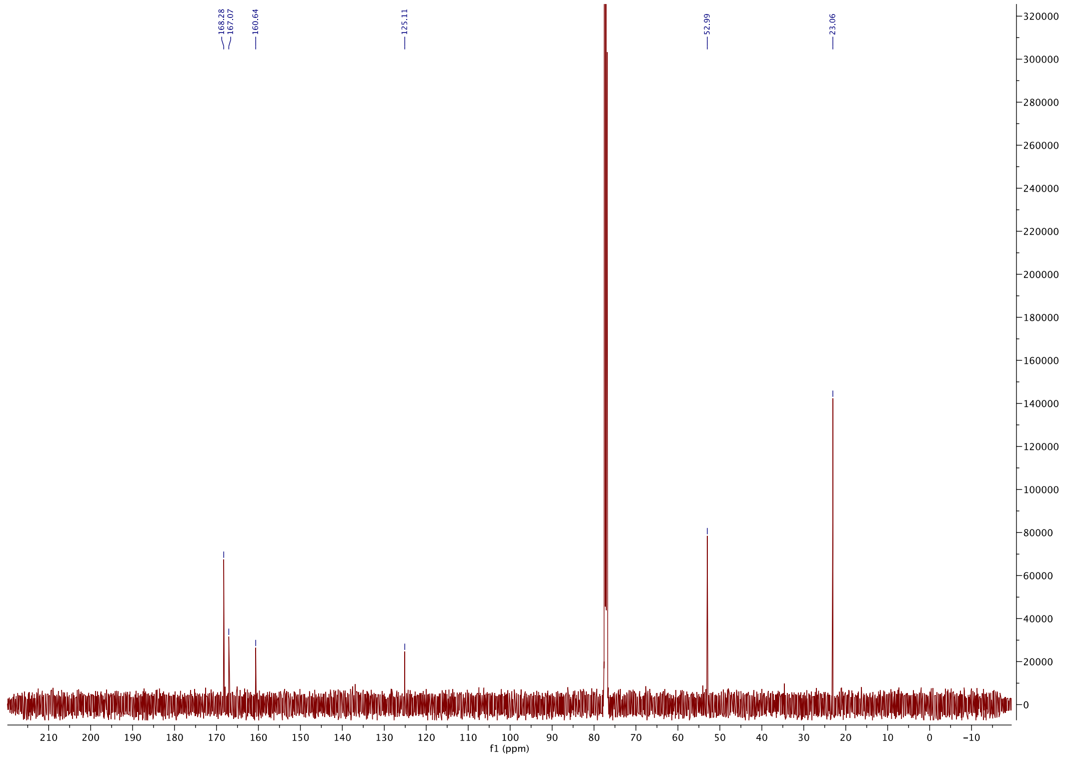
168.28
167.07
160.64
125.11
52.99
23.06
f1 (ppm)

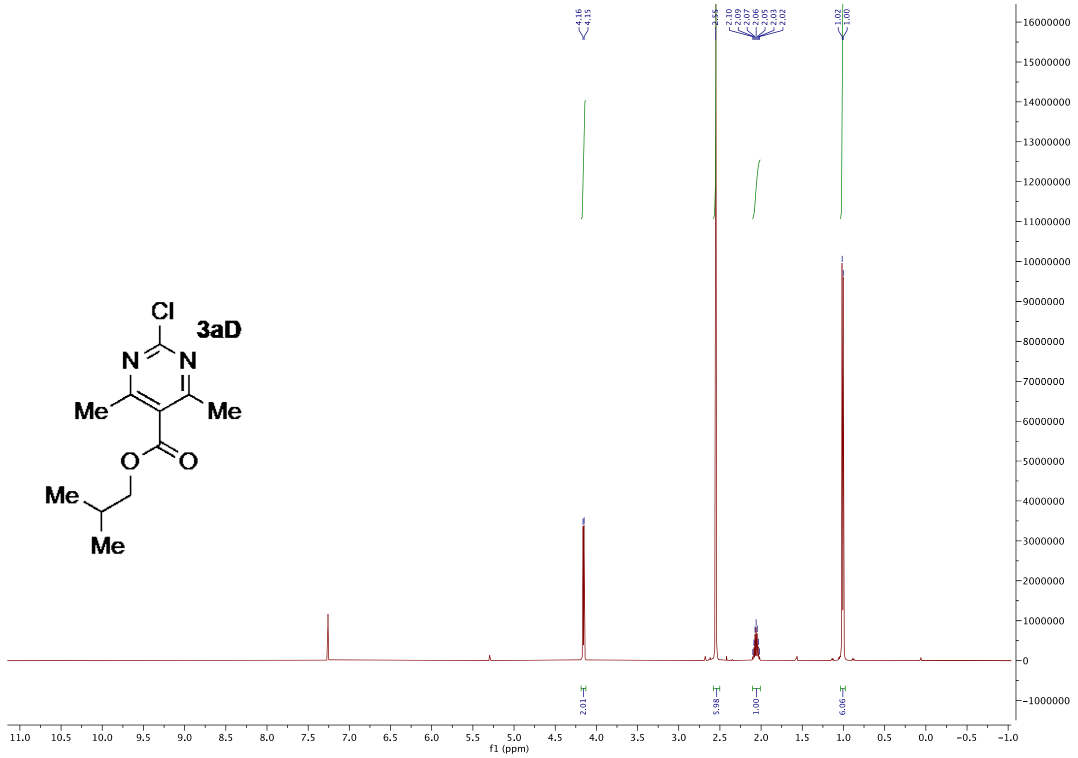

3aD
4.16
4.15
2.55
2.10
2.09
2.07
2.06
2.05
2.03
2.02
1.02
1.00
2.01
5.98
1.00
6.06
f1 (ppm)

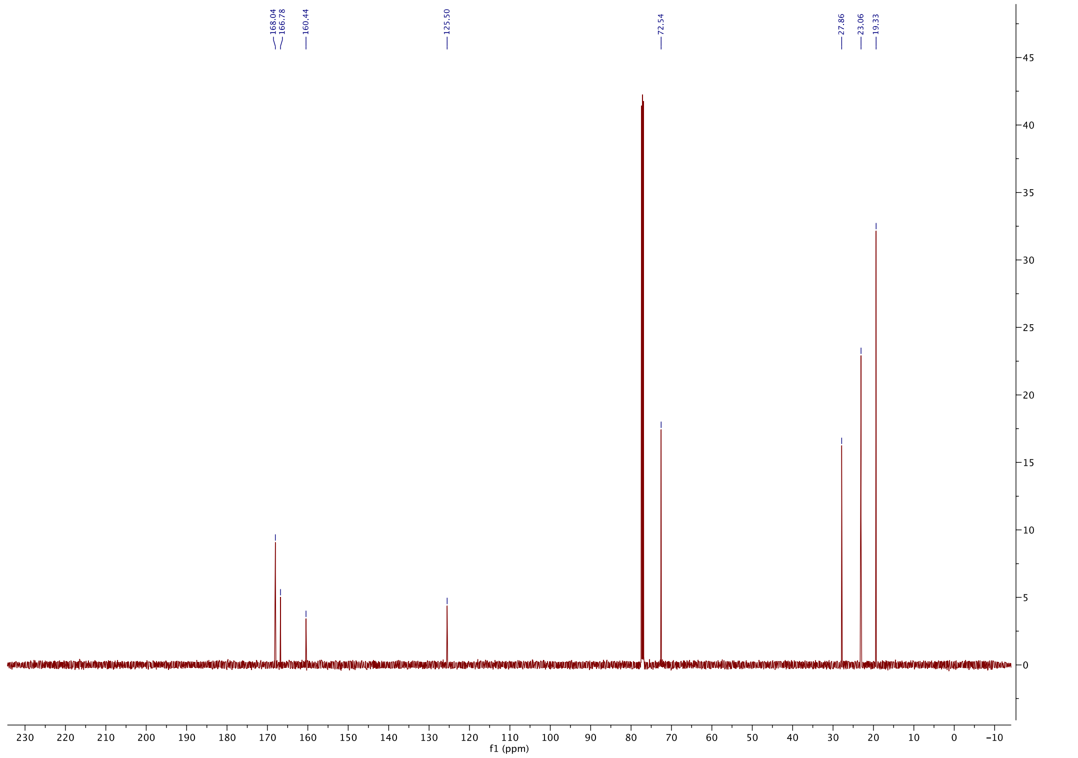

168.04
166.78
160.44
125.50
72.54
27.86
23.06
19.33
f1 (ppm)

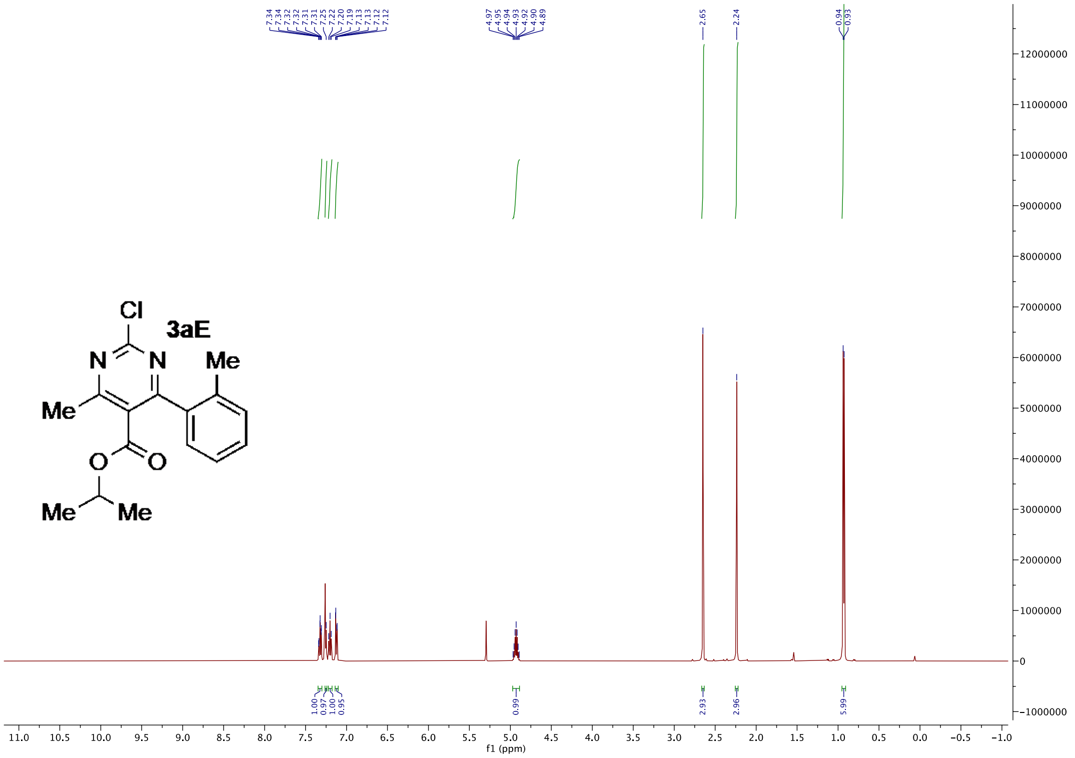
3aE
Cl
N
N
Me
Me
O
O
Me
Me
7.34
7.34
7.32
7.32
7.31
7.31
7.25
7.22
7.20
7.19
7.13
7.13
7.12
7.12
4.97
4.95
4.94
4.93
4.92
4.90
4.89
2.65
2.24
0.94
0.93
1.00
0.97
1.00
0.95
0.99
2.93
2.96
5.99
f1 (ppm)

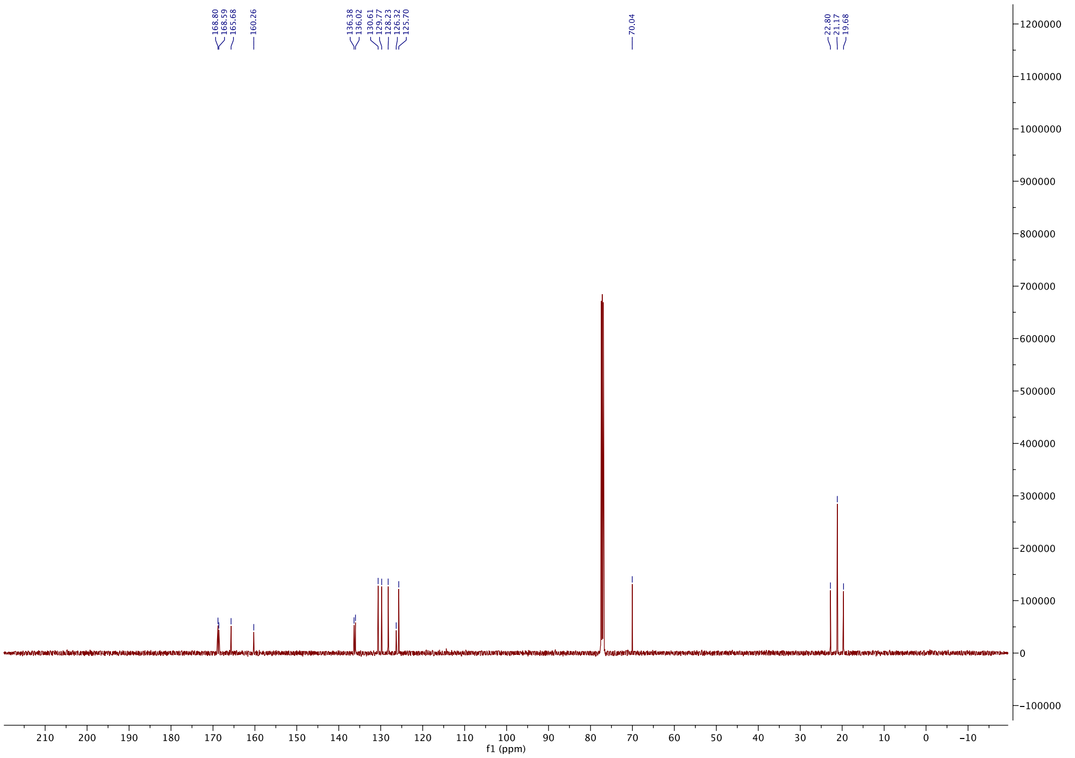
168.80
168.59
165.68
160.26
136.38
136.02
130.61
129.77
128.23
126.32
125.70
70.04
22.80
21.17
19.68
f1 (ppm)

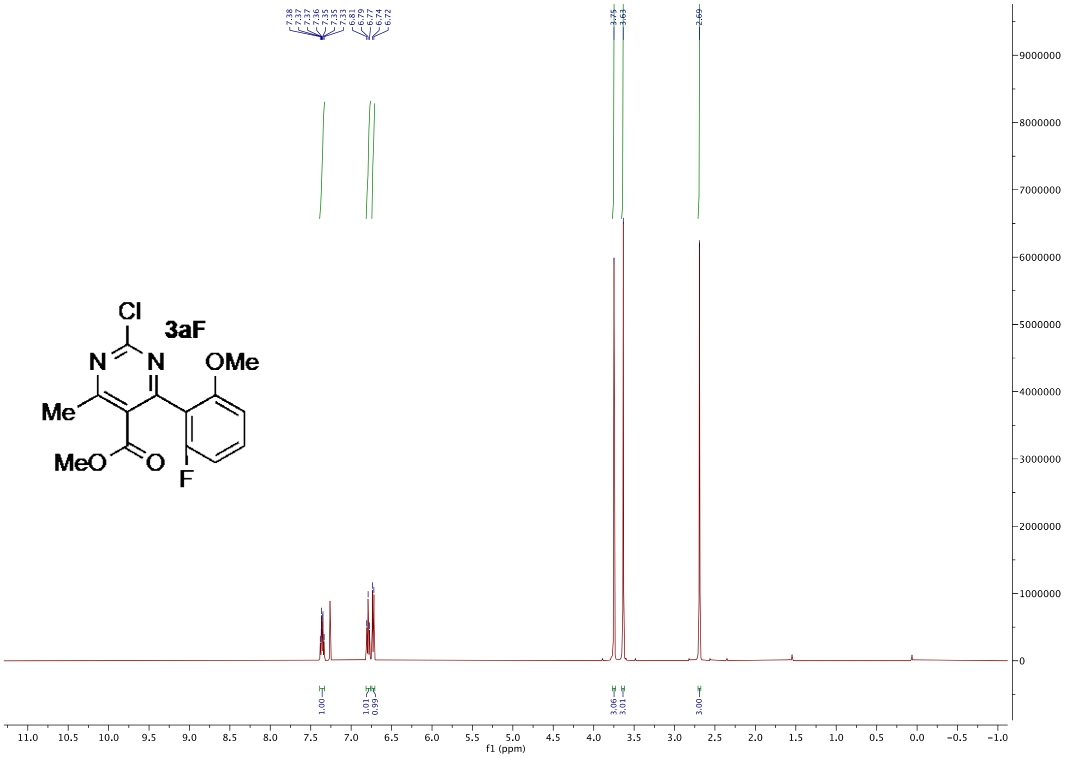
3aF
Cl
N
N
OMe
Me
MeO
O
F
f1 (ppm)

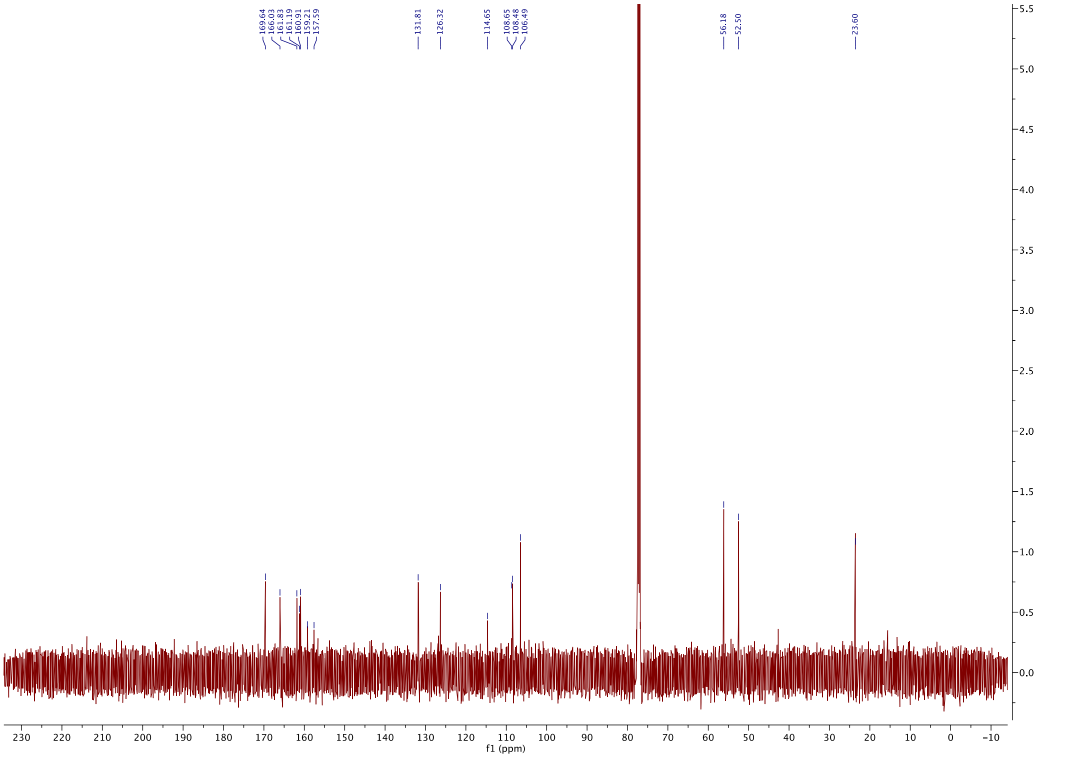
169.64
166.03
161.83
161.19
160.91
159.21
157.59
131.81
126.32
114.65
108.65
108.48
106.49
56.18
52.50
23.60
f1 (ppm)

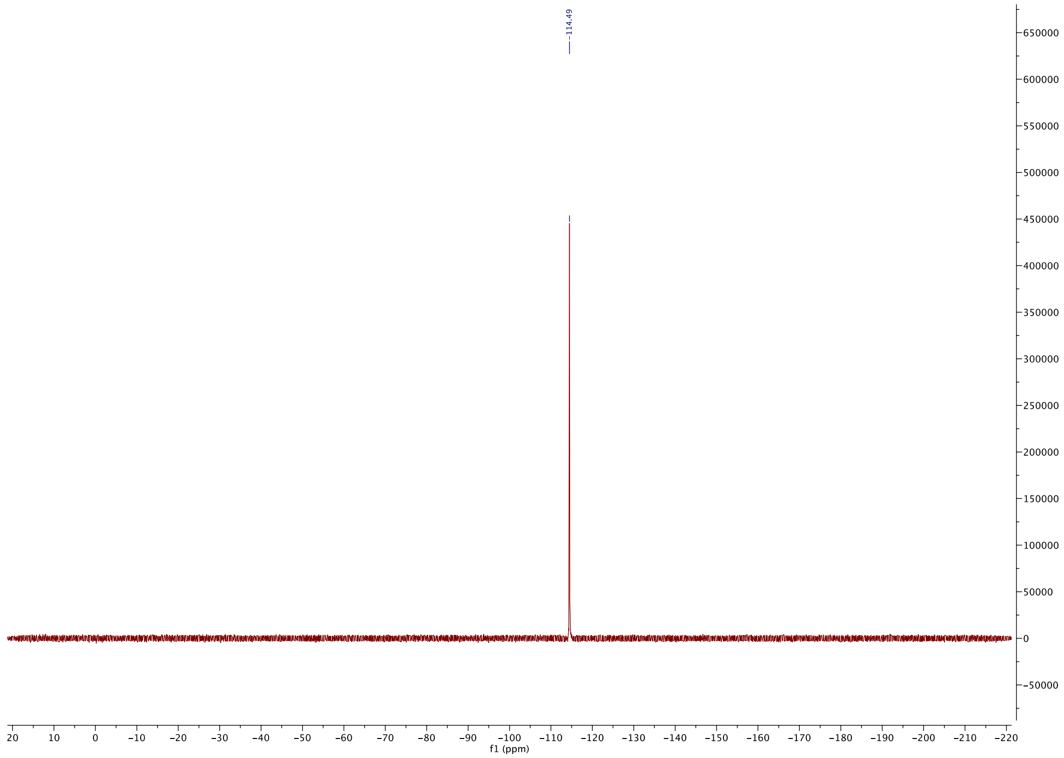

-114.49
650000
600000
550000
500000
450000
400000
350000
300000
250000
200000
150000
100000
50000
0
-50000
20 10 0 -10 -20 -30 -40 -50 -60 -70 -80 -90 -100 -110 -120 -130 -140 -150 -160 -170 -180 -190 -200 -210 -220
f1 (ppm)

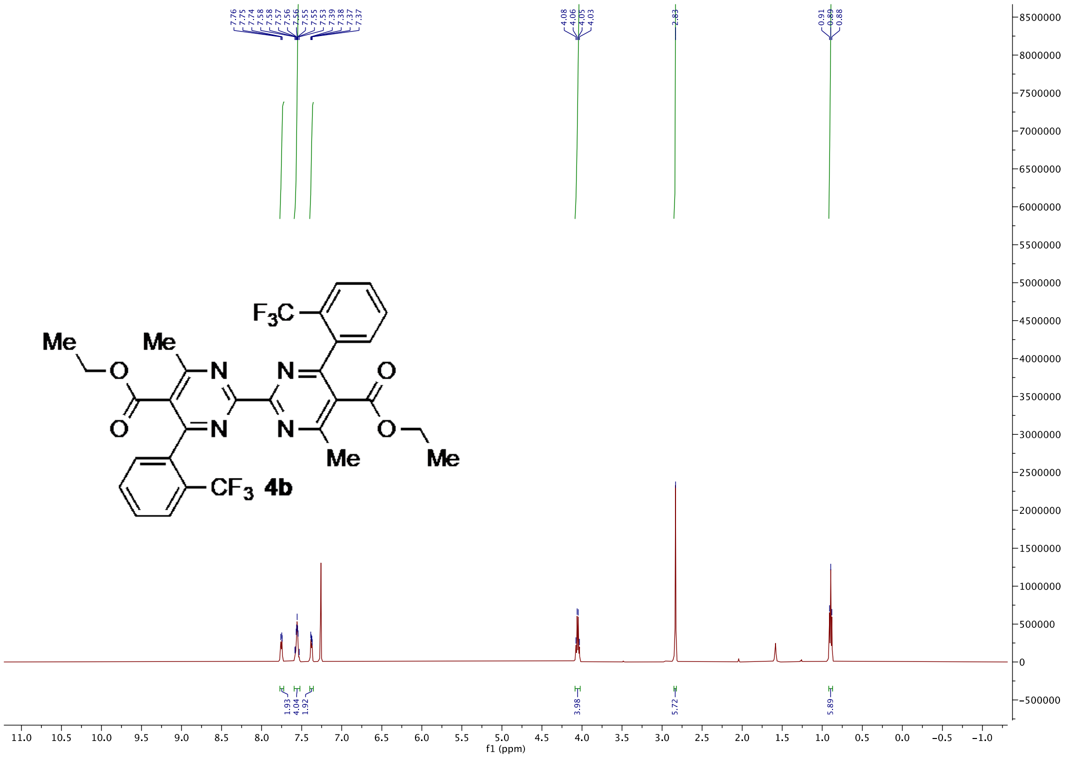
7.76 7.75 7.74 7.58 7.58 7.57 7.56 7.56 7.55 7.55 7.53 7.39 7.38 7.37 7.37
4.08 4.06 4.05 4.03
2.83
0.91 0.89 0.88
1.93 4.04 1.92
3.98
5.72
5.89
F3C
Me
Me
N
N
O
O
O
O
N
N
Me
Me
CF3 4b
f1 (ppm)

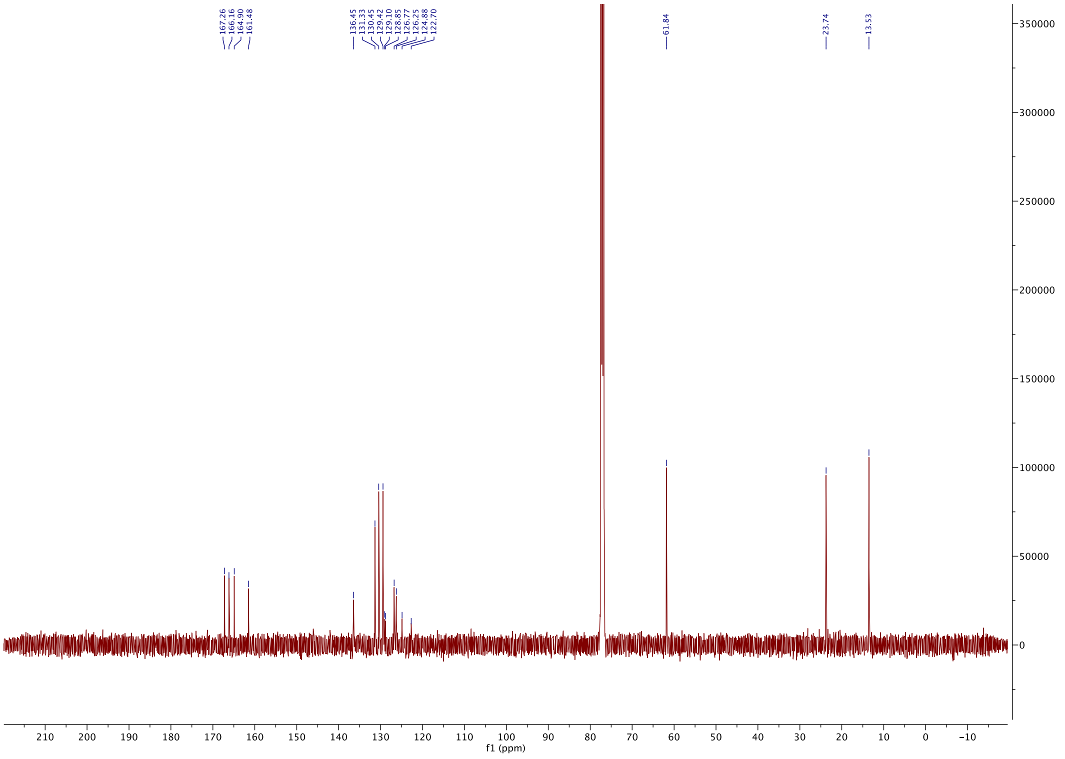
167.26 166.16 164.90 161.48
136.45 131.33 130.45 129.42 129.10 128.85 126.77 126.25 124.88 122.70
61.84
23.74
13.53
f1 (ppm)

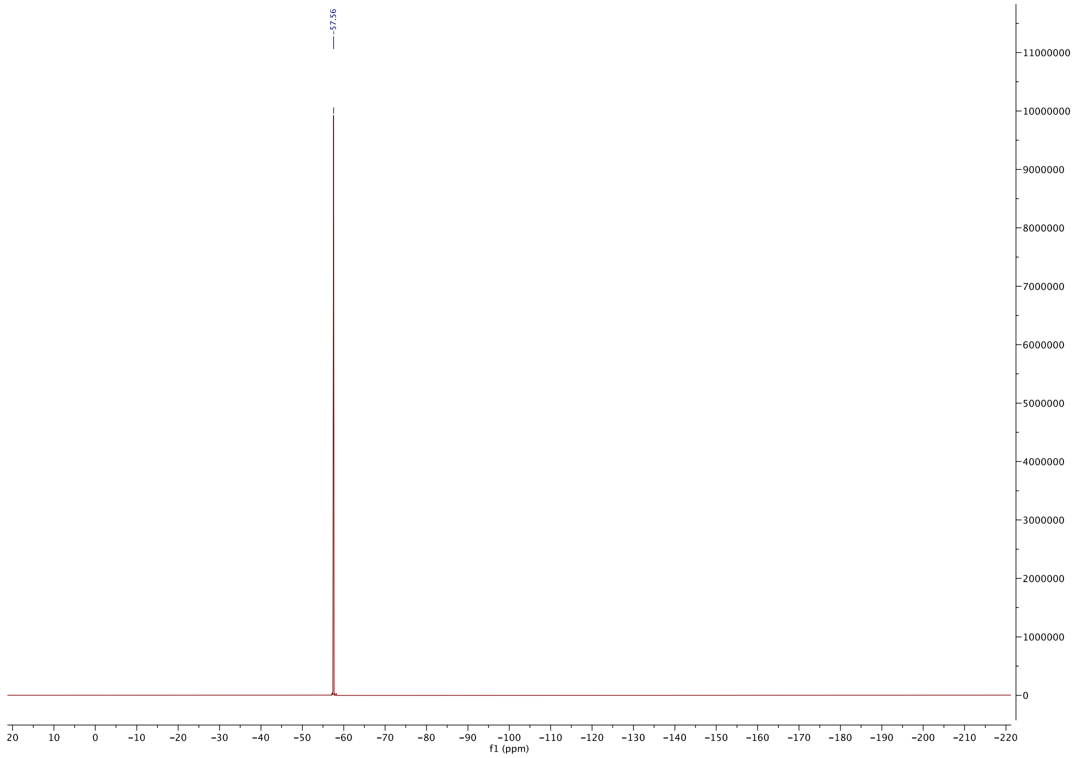

-57.56
11000000
10000000
9000000
8000000
7000000
6000000
5000000
4000000
3000000
2000000
1000000
0
20
10
0
-10
-20
-30
-40
-50
-60
-70
-80
-90
-100
-110
-120
-130
-140
-150
-160
-170
-180
-190
-200
-210
-220
f1 (ppm)

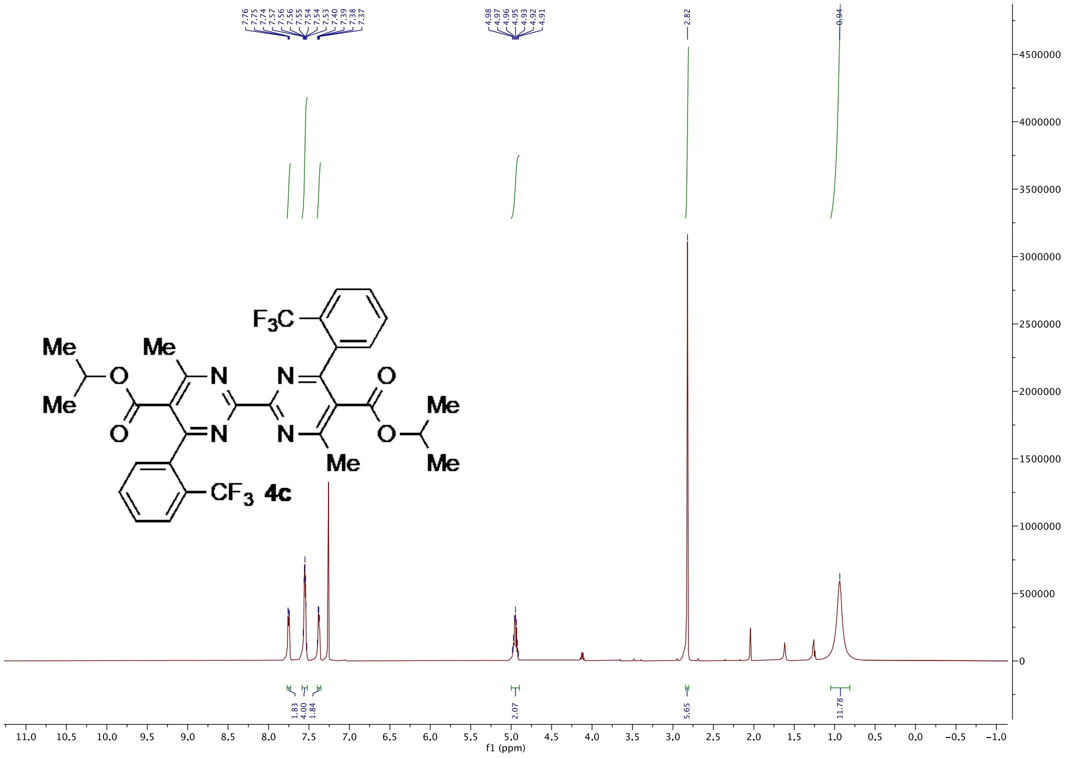
7.76
7.75
7.74
7.57
7.56
7.55
7.54
7.53
7.40
7.39
7.38
7.37
4.98
4.97
4.96
4.95
4.93
4.92
4.91
2.82
0.94
1.83
4.00
1.84
2.07
5.65
11.78
f1 (ppm)
Me
O
N
F3C
CF3
4c

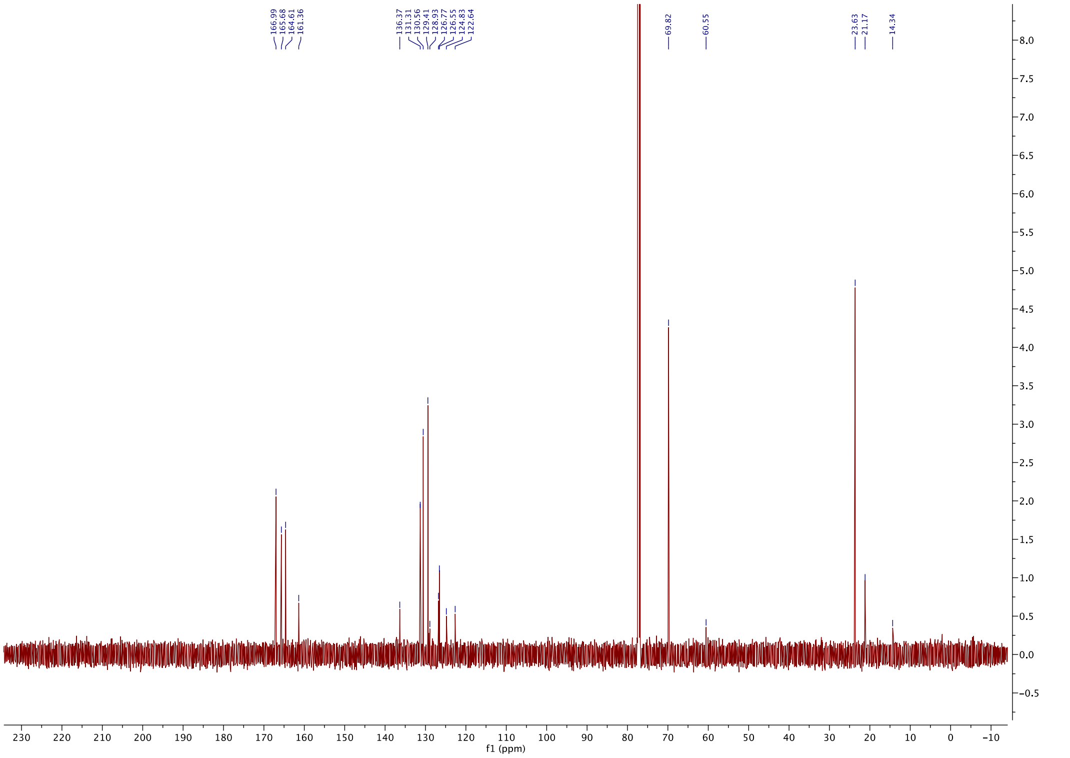
166.99
165.68
164.61
161.36
136.37
131.31
130.56
129.41
128.93
126.77
126.55
124.83
122.64
69.82
60.55
23.63
21.17
14.34
f1 (ppm)

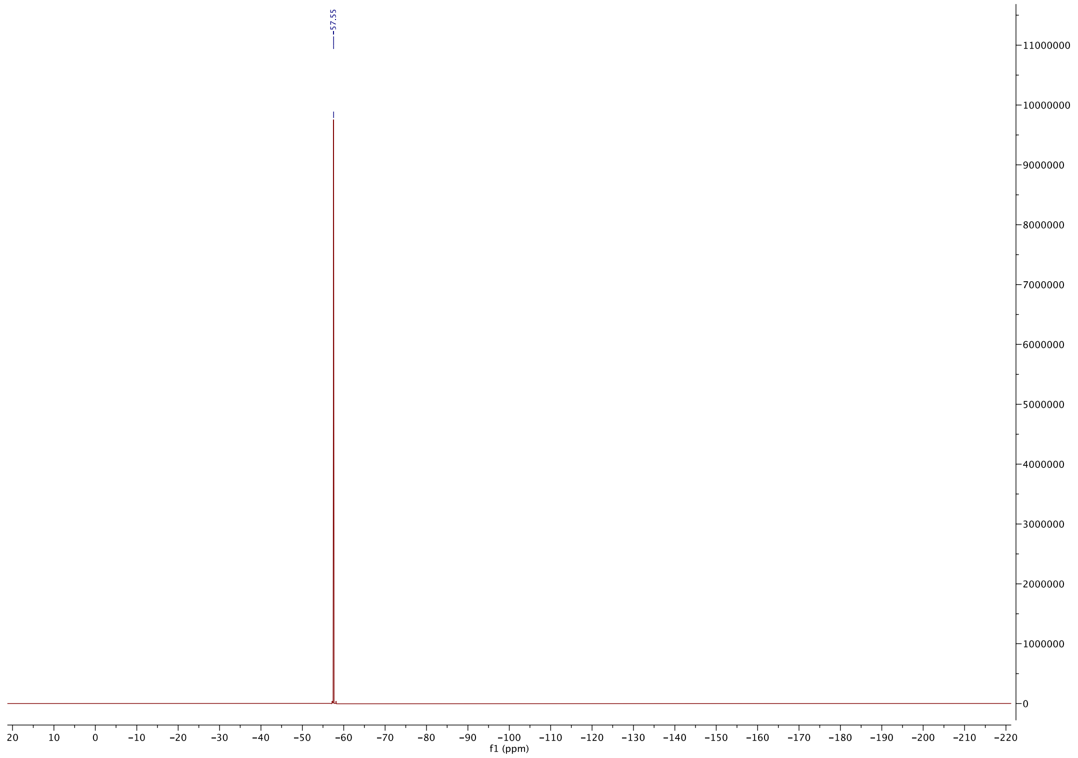

-57.55
11000000
10000000
9000000
8000000
7000000
6000000
5000000
4000000
3000000
2000000
1000000
0
20 10 0 −10 −20 −30 −40 −50 −60 −70 −80 −90 −100 −110 −120 −130 −140 −150 −160 −170 −180 −190 −200 −210 −220
f1 (ppm)

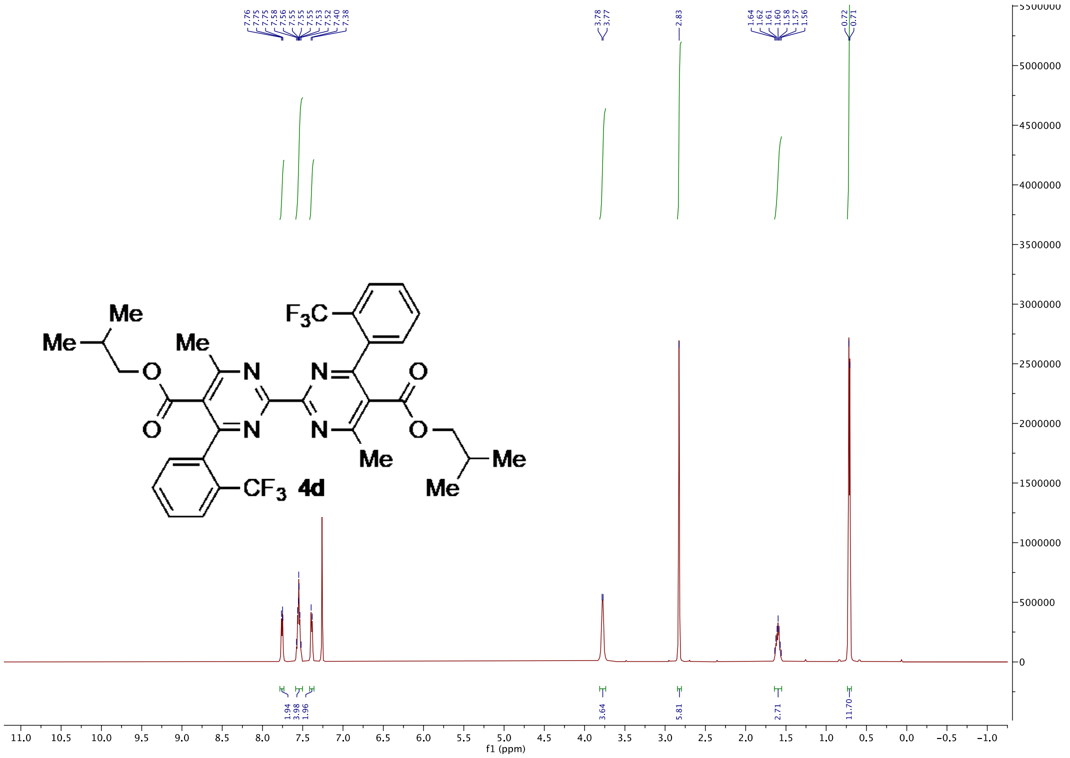

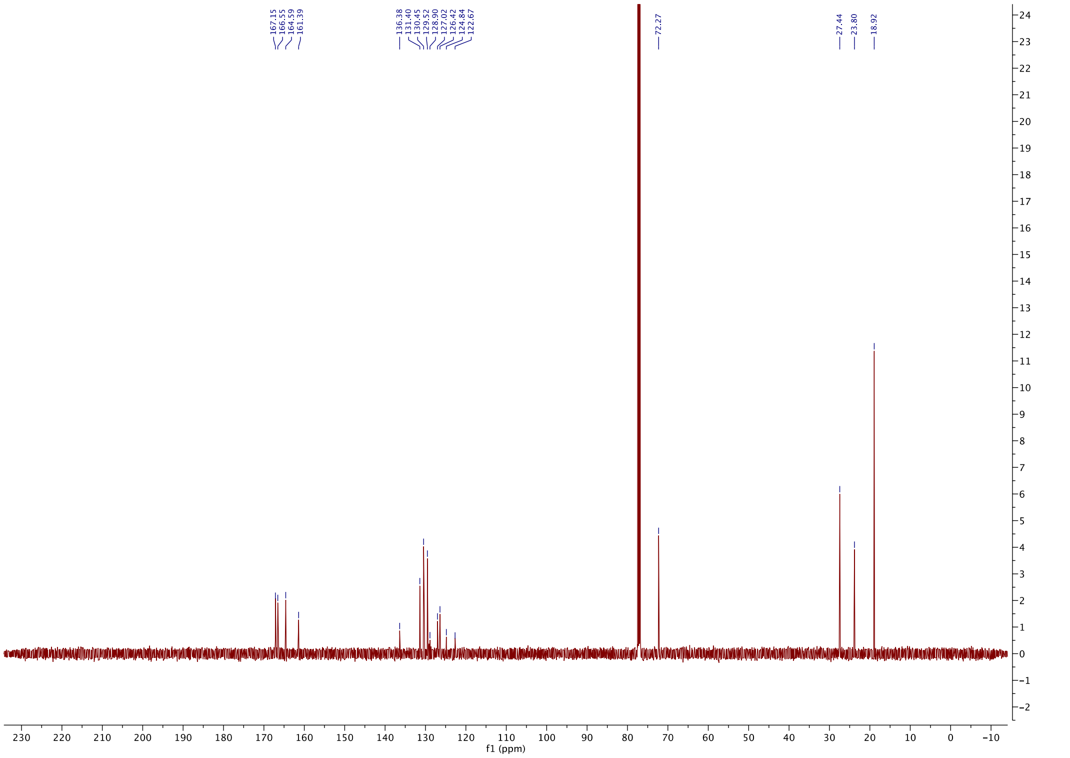

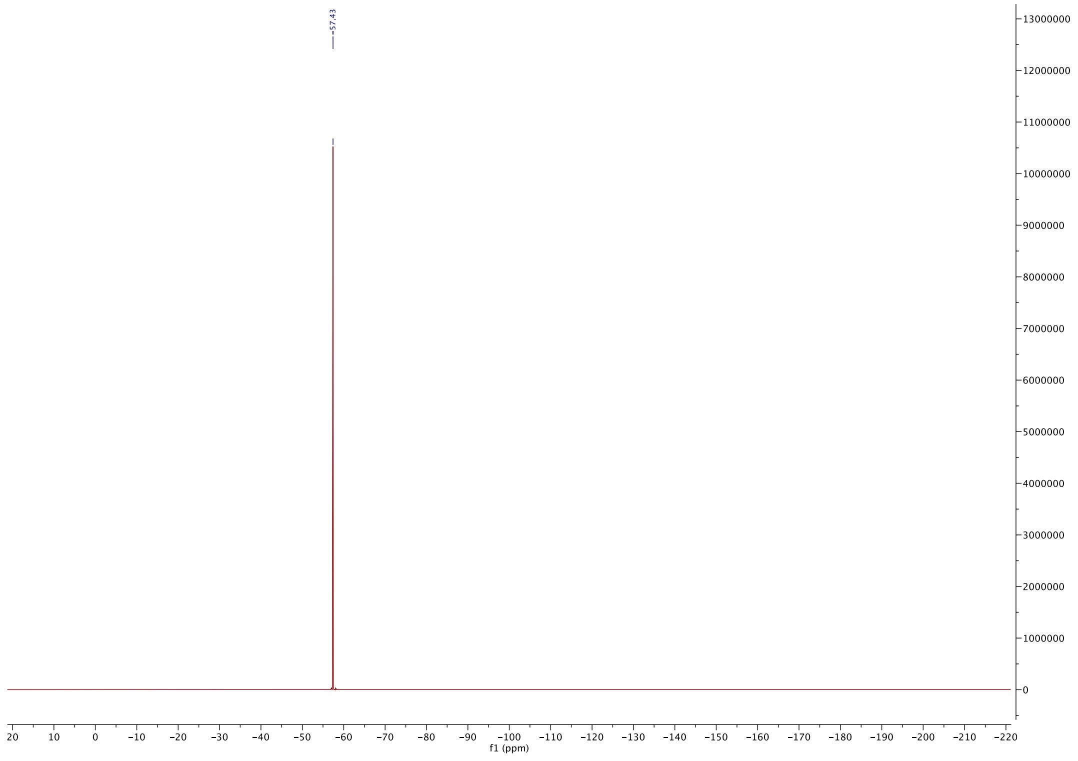

-57.43
13000000
12000000
11000000
10000000
9000000
8000000
7000000
6000000
5000000
4000000
3000000
2000000
1000000
0
20 10 0 −10 −20 −30 −40 −50 −60 −70 −80 −90 −100 −110 −120 −130 −140 −150 −160 −170 −180 −190 −200 −210 −220
f1 (ppm)

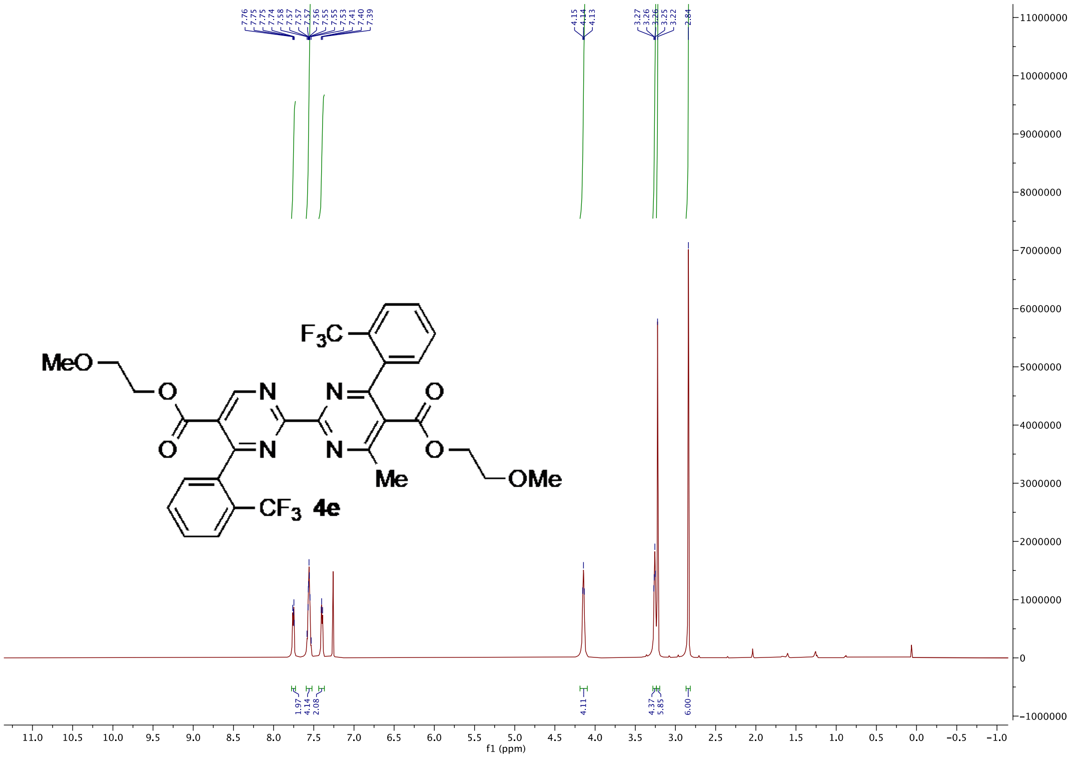
7.76
7.75
7.75
7.74
7.58
7.57
7.57
7.56
7.55
7.55
7.53
7.41
7.40
7.39
4.15
4.14
4.13
3.27
3.26
3.26
3.25
3.22
2.84
MeO
O
O
N
N
N
N
F3C
O
O
Me
OMe
CF3
4e
1.97
4.14
2.08
4.11
4.37
5.85
6.00
f1 (ppm)

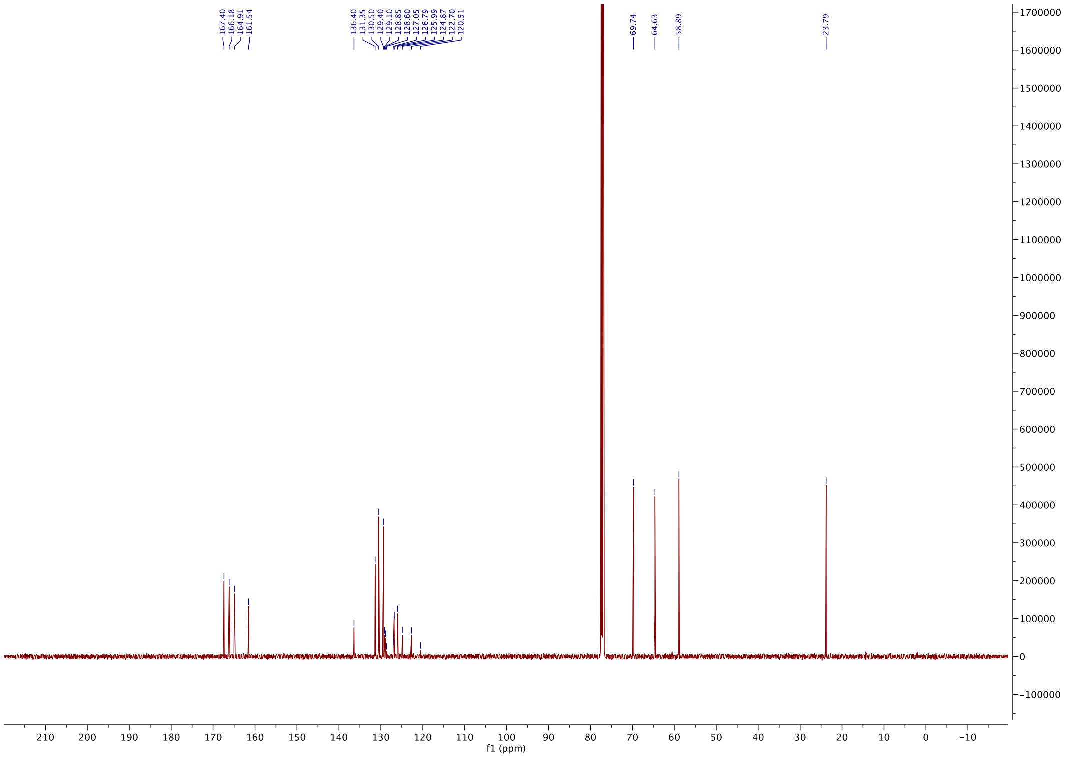
167.40
166.18
164.91
161.54
136.40
131.35
130.50
129.40
129.10
128.85
128.60
127.05
126.79
125.99
124.87
122.70
120.51
69.74
64.63
58.89
23.79
f1 (ppm)

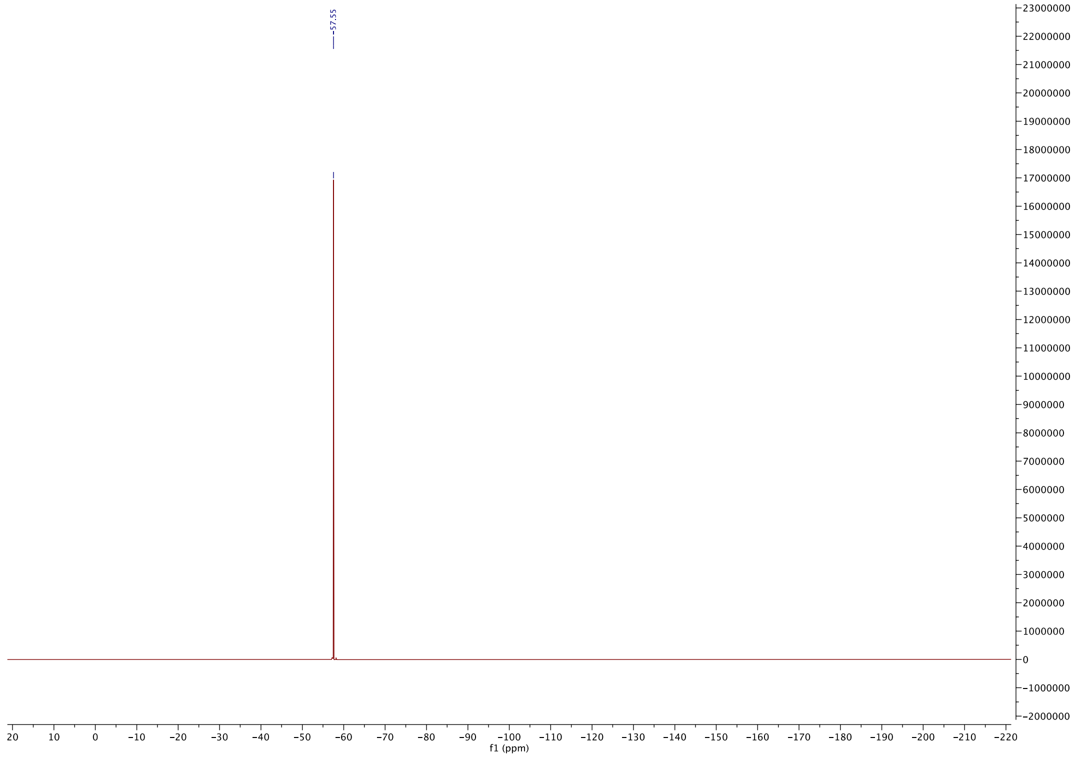

-57.55
f1 (ppm)

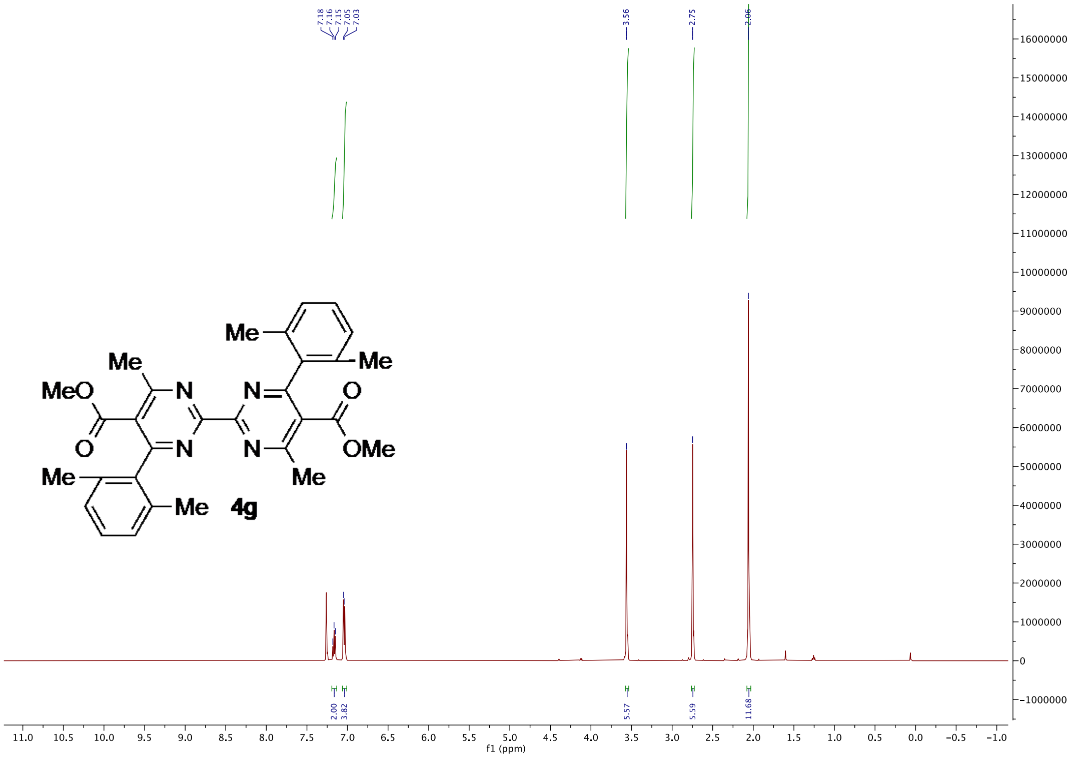
7.18
7.16
7.15
7.05
7.03
3.56
2.75
2.06
2.00
3.82
5.57
5.59
11.68
f1 (ppm)

4g

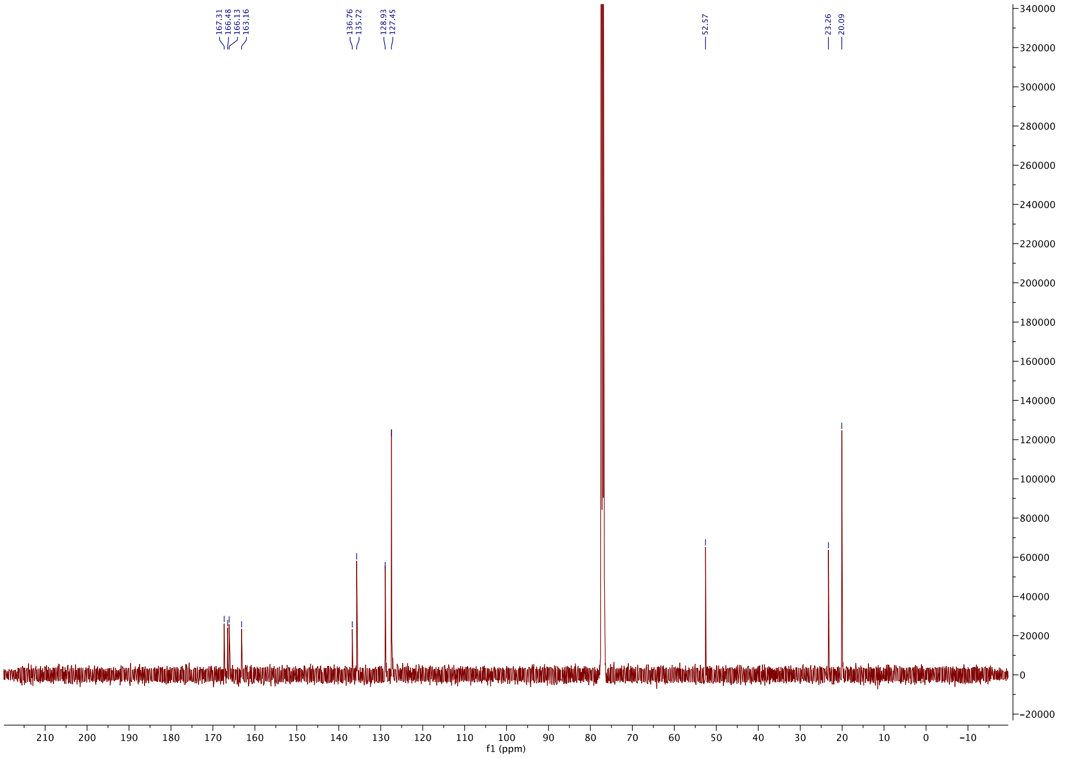
167.31
166.48
166.13
163.16
136.76
135.72
128.93
127.45
52.57
23.26
20.09
f1 (ppm)

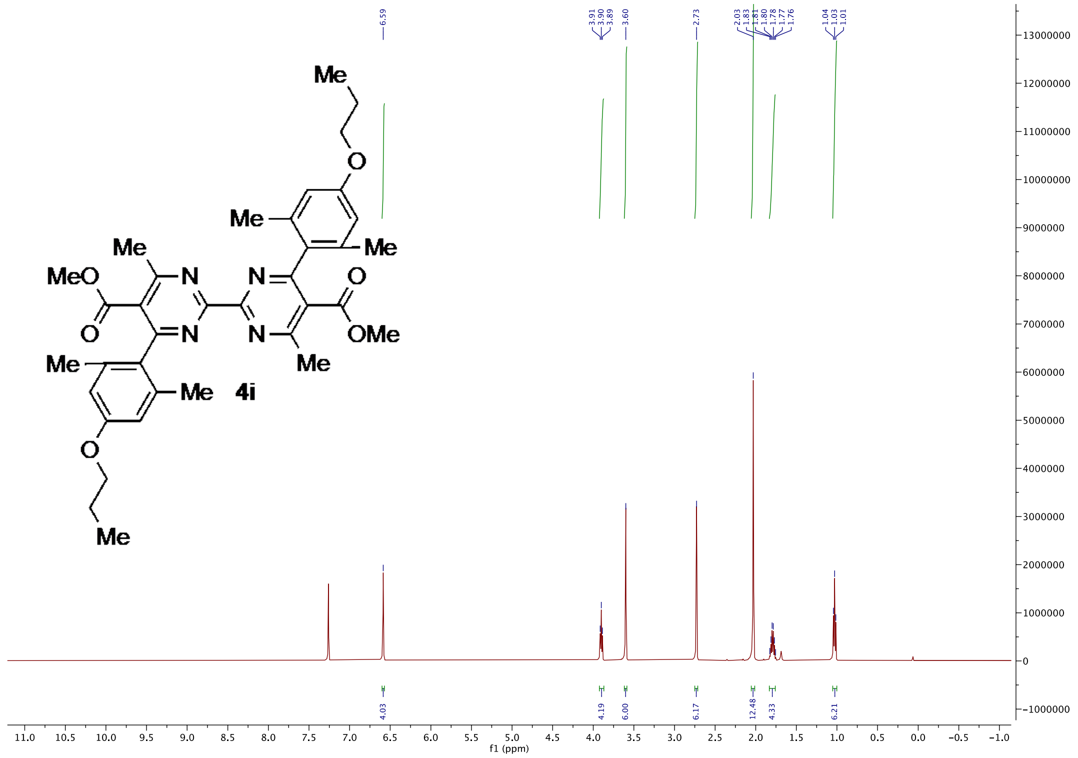
4i

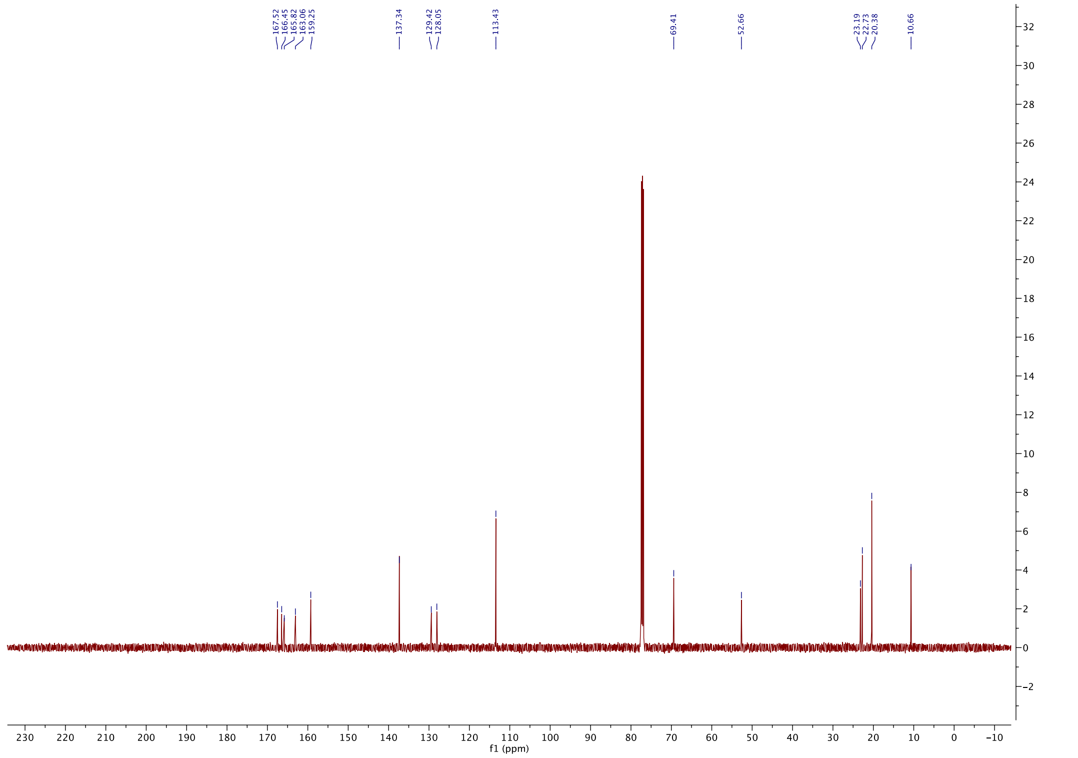

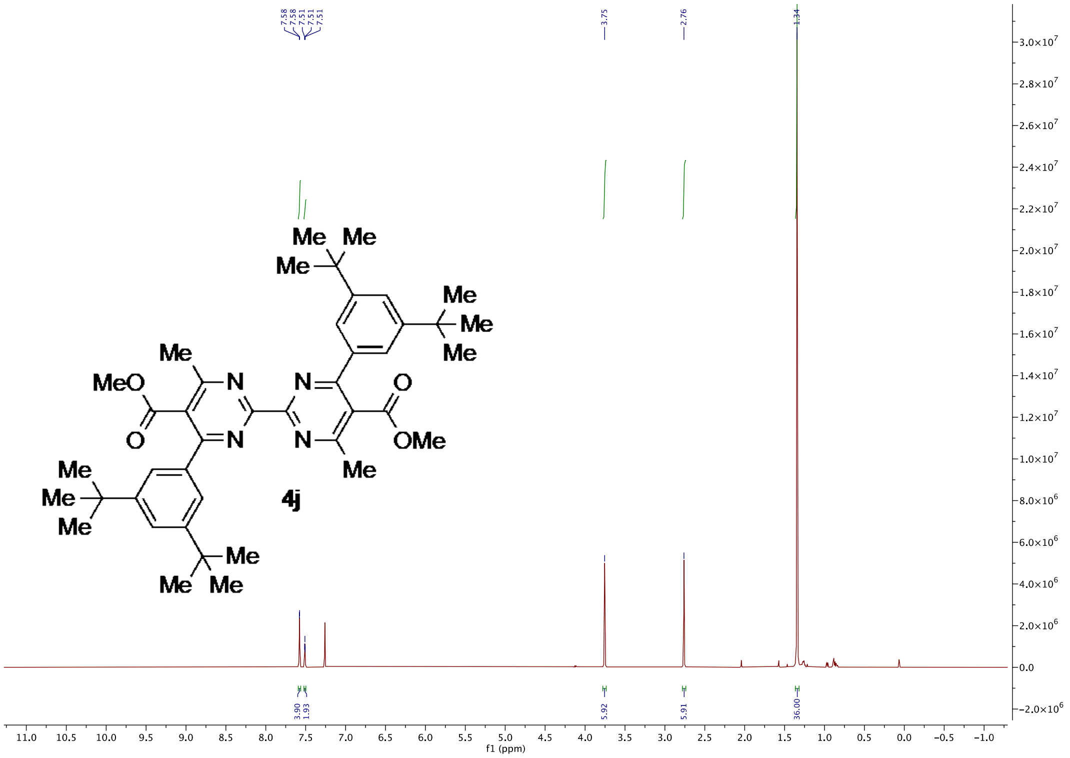
7.58
7.58
7.51
7.51
7.51
3.75
2.76
1.34
3.90
1.93
5.92
5.91
36.00
Me
Me
Me
MeO
O
N
N
O
OMe
4j
f1 (ppm)

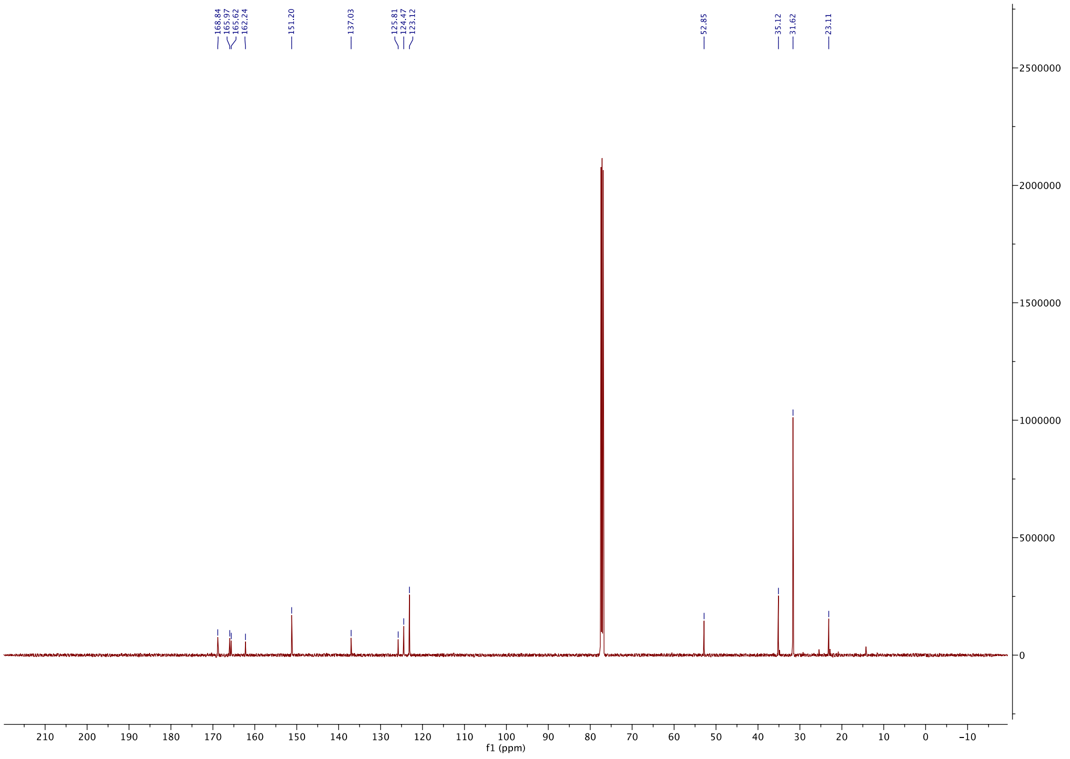
168.84
165.97
165.62
162.24
151.20
137.03
125.81
124.47
123.12
52.85
35.12
31.62
23.11
f1 (ppm)

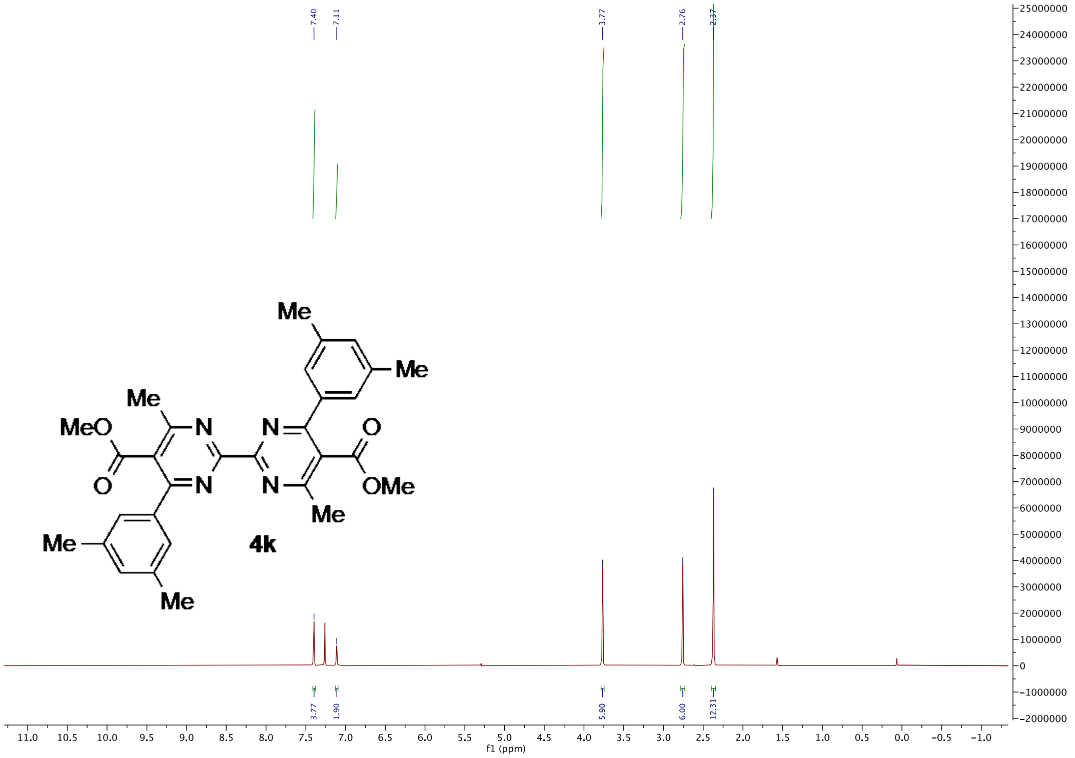
7.40
7.11
3.77
2.76
2.37
3.77
1.90
5.90
6.00
12.31
f1 (ppm)
Me
MeO
O
N
OMe
4k

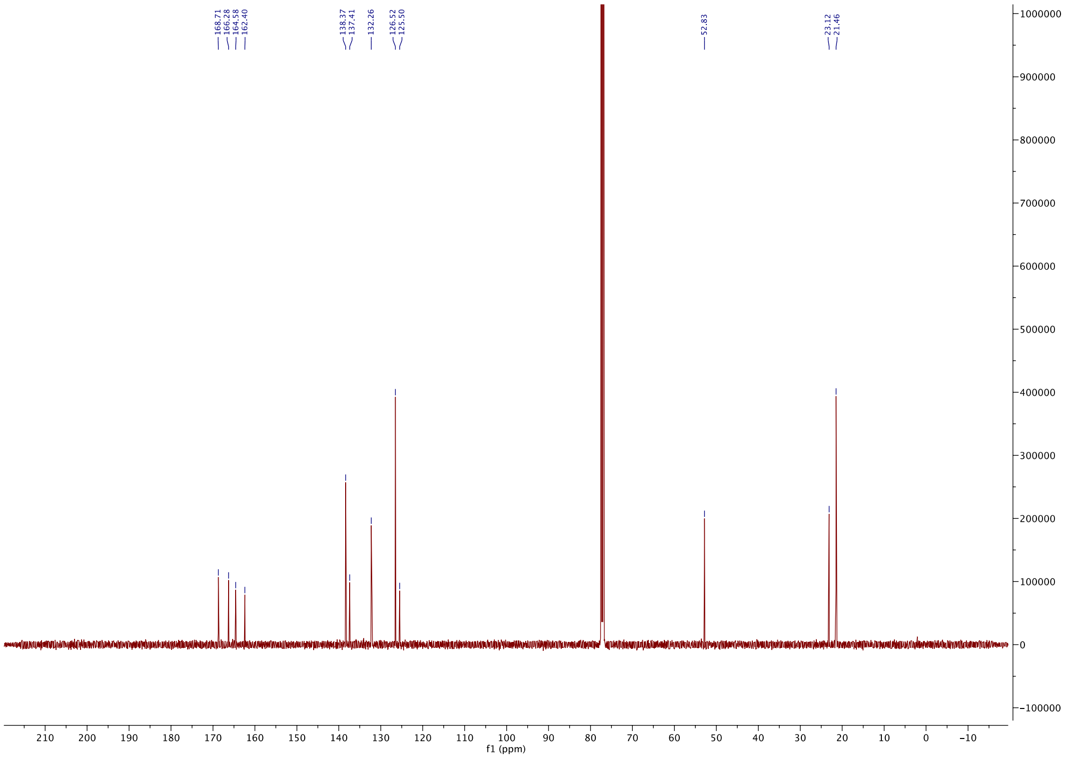
168.71
166.28
164.58
162.40
138.37
137.41
132.26
126.52
125.50
52.83
23.12
21.46
f1 (ppm)

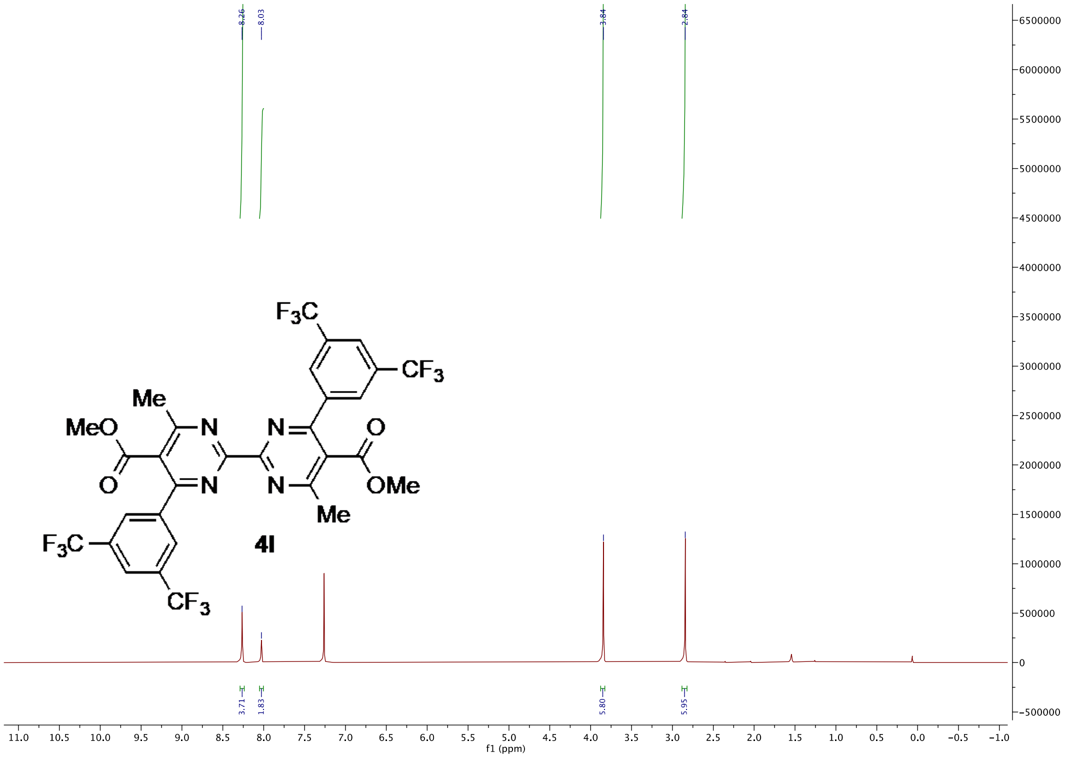
8.26
8.03
3.84
2.84
3.71
1.83
5.80
5.95
4l
f1 (ppm)

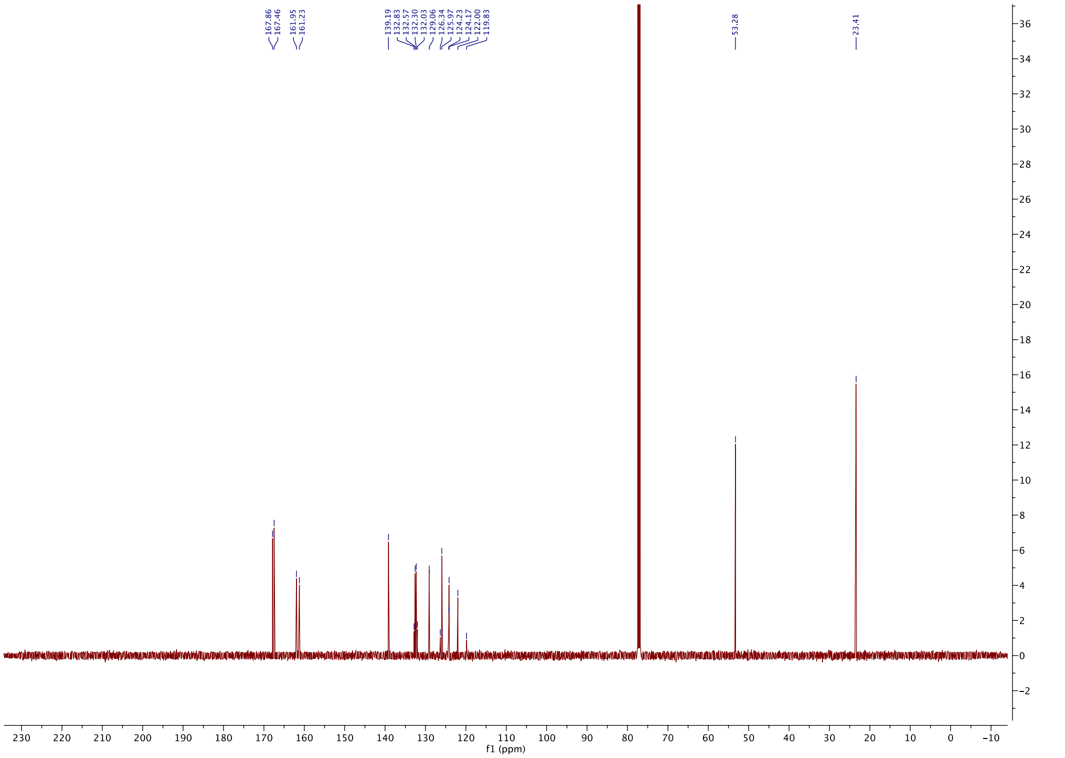
167.86
167.46
161.95
161.23
139.19
132.83
132.57
132.30
132.03
129.06
126.34
125.97
124.23
124.17
122.00
119.83
53.28
23.41
f1 (ppm)

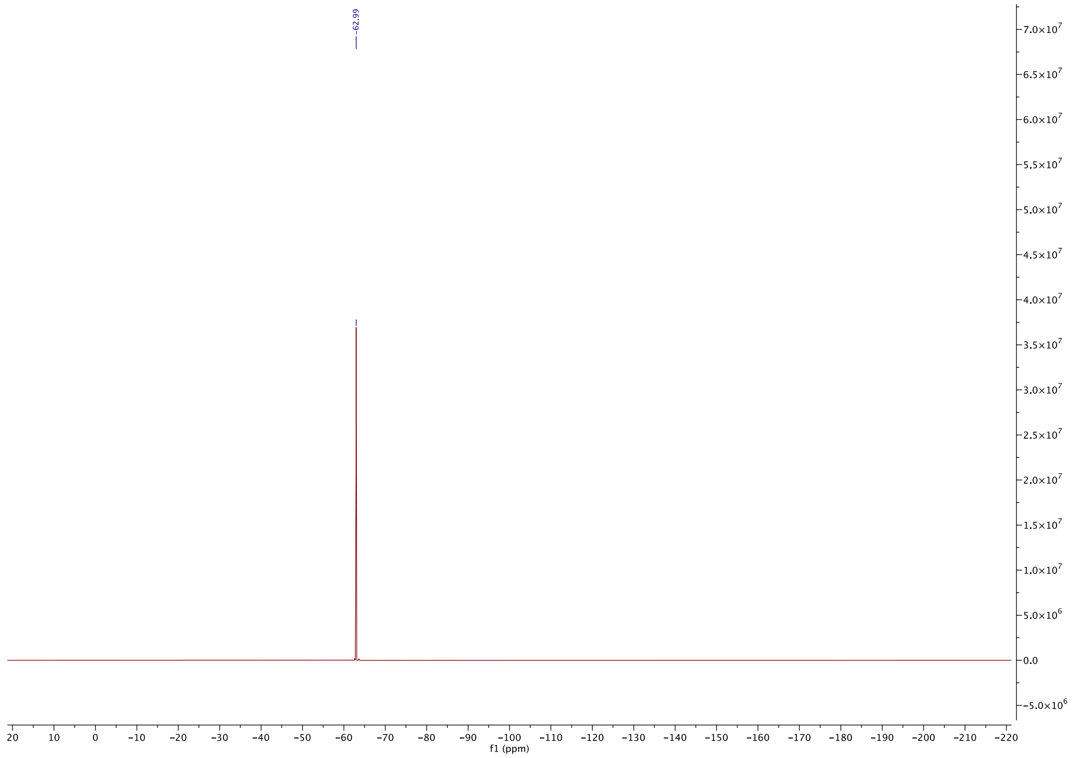
-62.99
7.0×10^7
6.5×10^7
6.0×10^7
5.5×10^7
5.0×10^7
4.5×10^7
4.0×10^7
3.5×10^7
3.0×10^7
2.5×10^7
2.0×10^7
1.5×10^7
1.0×10^7
5.0×10^6
0.0
-5.0×10^6
20 10 0 -10 -20 -30 -40 -50 -60 -70 -80 -90 -100 -110 -120 -130 -140 -150 -160 -170 -180 -190 -200 -210 -220
f1 (ppm)

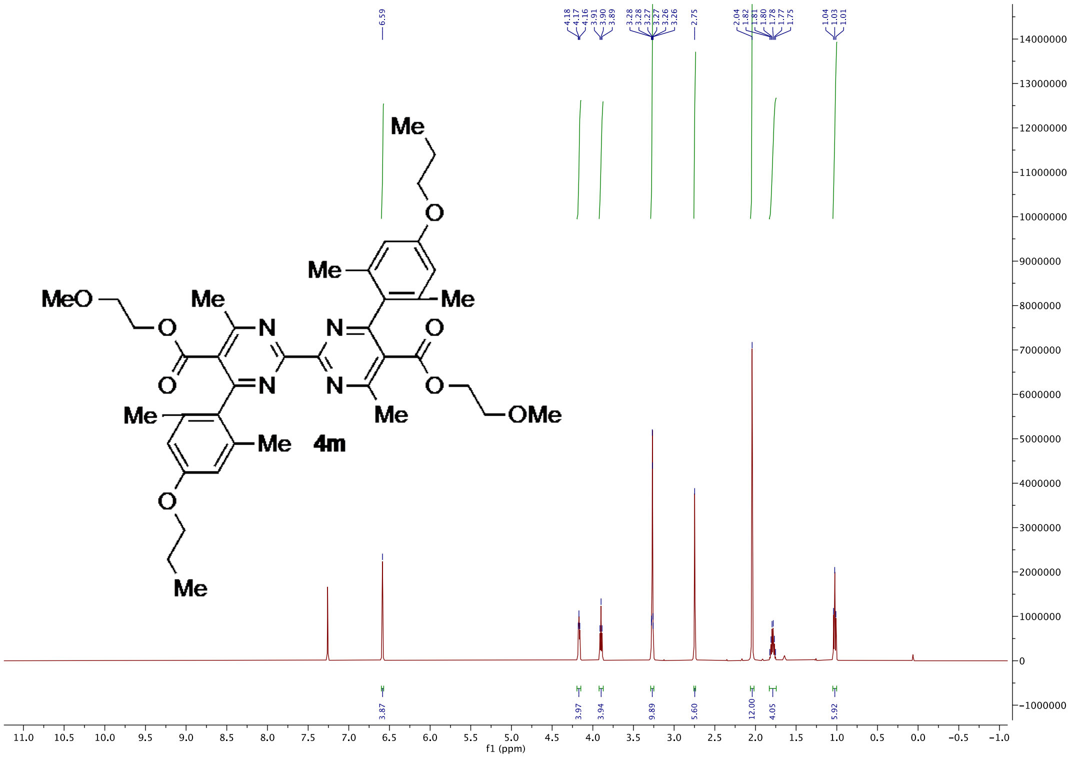
Me
O
Me
Me
MeO
Me
N
N
O
O
O
N
N
O
OMe
Me
Me
Me
4m
O
Me
6.59
4.18
4.17
4.16
3.91
3.90
3.89
3.28
3.28
3.27
3.27
3.26
3.26
2.75
2.04
1.82
1.81
1.80
1.78
1.77
1.75
1.04
1.03
1.01
3.87
3.97
3.94
9.89
5.60
12.00
4.05
5.92
f1 (ppm)

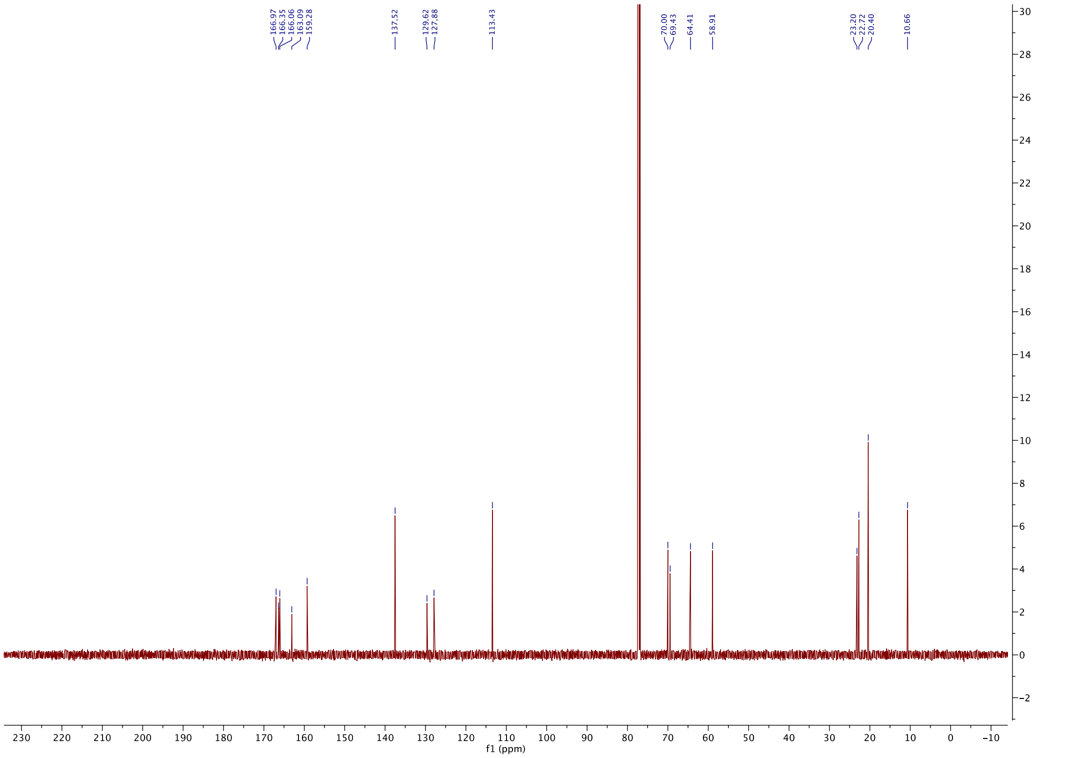
166.97
166.35
166.06
163.09
159.28
137.52
129.62
127.88
113.43
70.00
69.43
64.41
58.91
23.20
22.72
20.40
10.66
f1 (ppm)

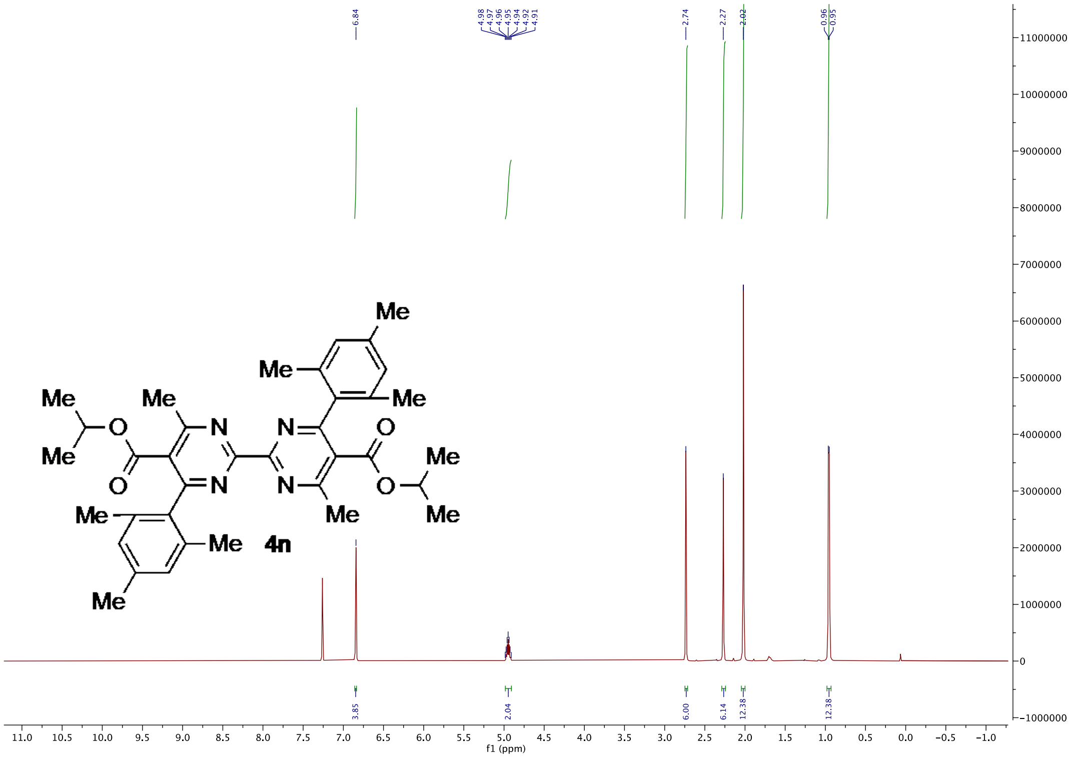

6.84
4.98 4.97 4.96 4.95 4.94 4.92 4.91
2.74
2.27
2.02
0.96 0.95
3.85
2.04
6.00
6.14
12.38
12.38
f1 (ppm)
4n

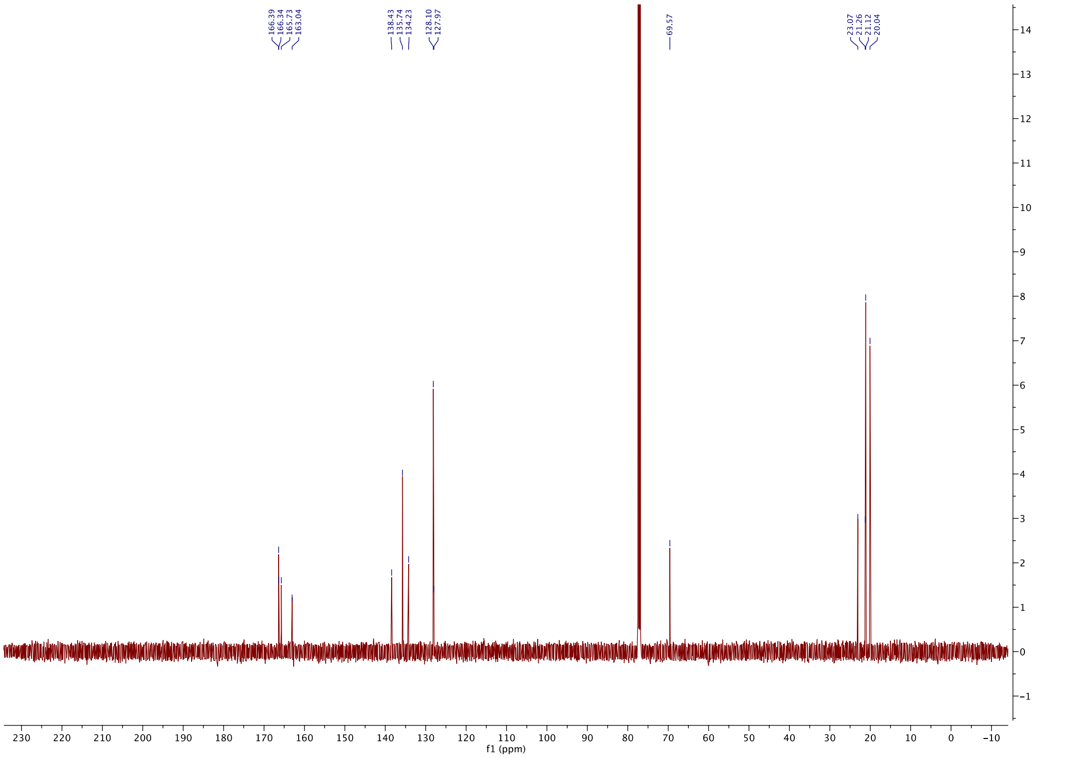

166.39 166.34 165.73 163.04
138.43 135.74 134.23
128.10 127.97
69.57
23.07 21.26 21.12 20.04
f1 (ppm)

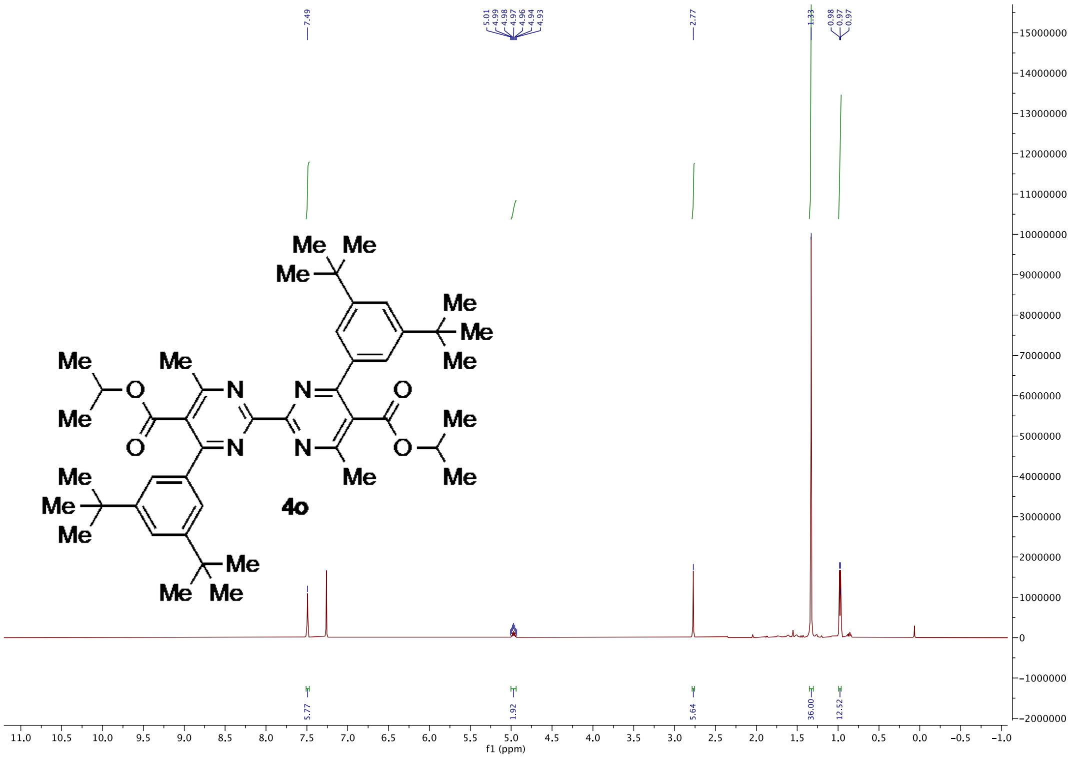
7.49
5.01
4.99
4.98
4.97
4.96
4.94
4.93
2.77
1.33
0.98
0.97
0.97
5.77
1.92
5.64
36.00
12.52
4o
f1 (ppm)

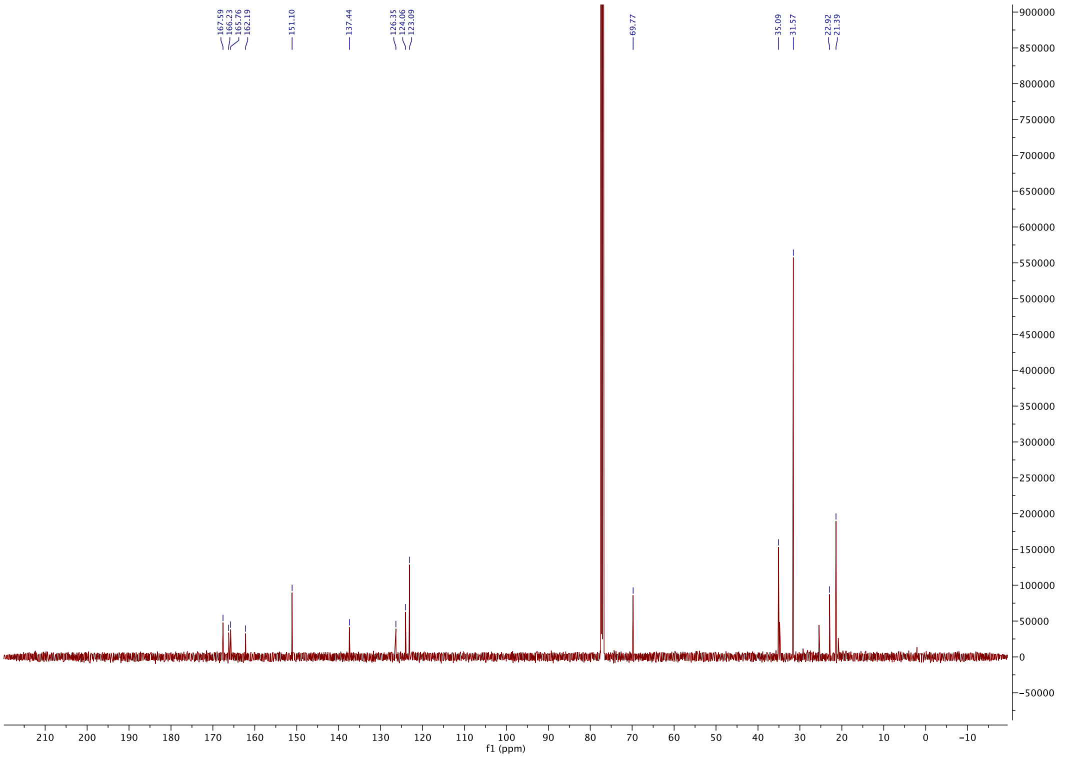
167.59
166.23
165.76
162.19
151.10
137.44
126.35
124.06
123.09
69.77
35.09
31.57
22.92
21.39
f1 (ppm)

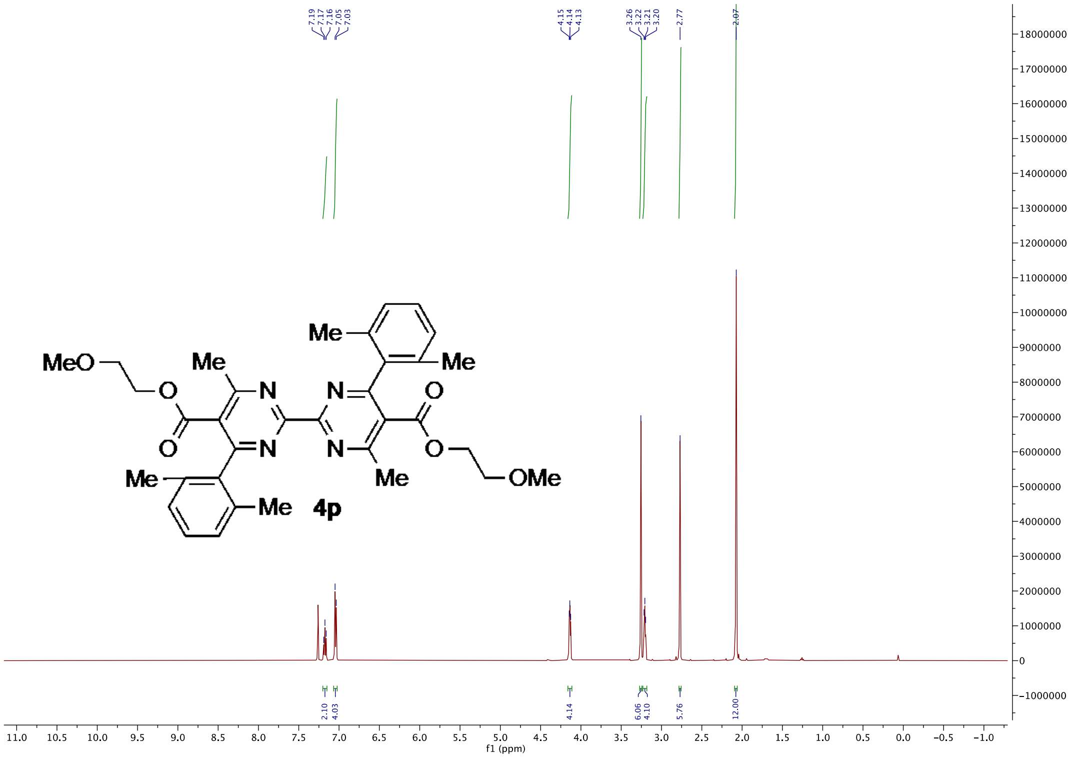
7.19
7.17
7.16
7.05
7.03
4.15
4.14
4.13
3.26
3.22
3.21
3.20
2.77
2.07
MeO
Me
N
N
O
O
N
N
Me
Me
Me
Me
O
O
OMe
Me
4p
2.10
4.03
4.14
6.06
4.10
5.76
12.00
f1 (ppm)

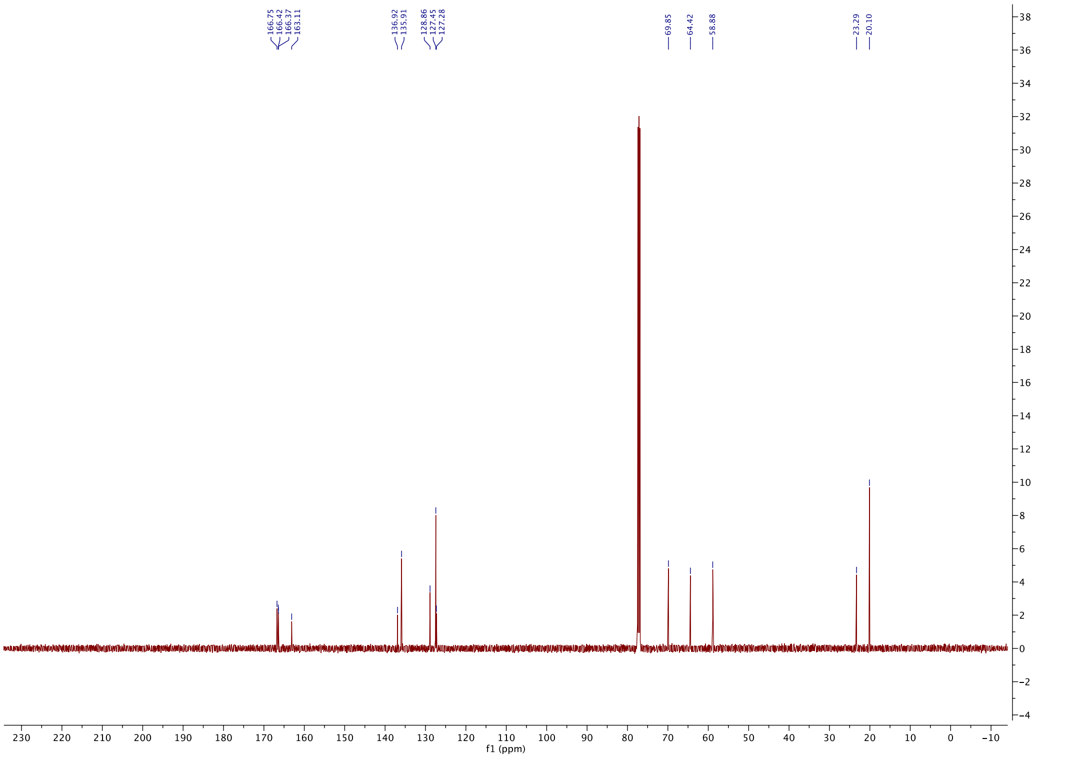
166.75
166.42
166.37
163.11
136.92
135.91
128.86
127.45
127.28
69.85
64.42
58.88
23.29
20.10
f1 (ppm)

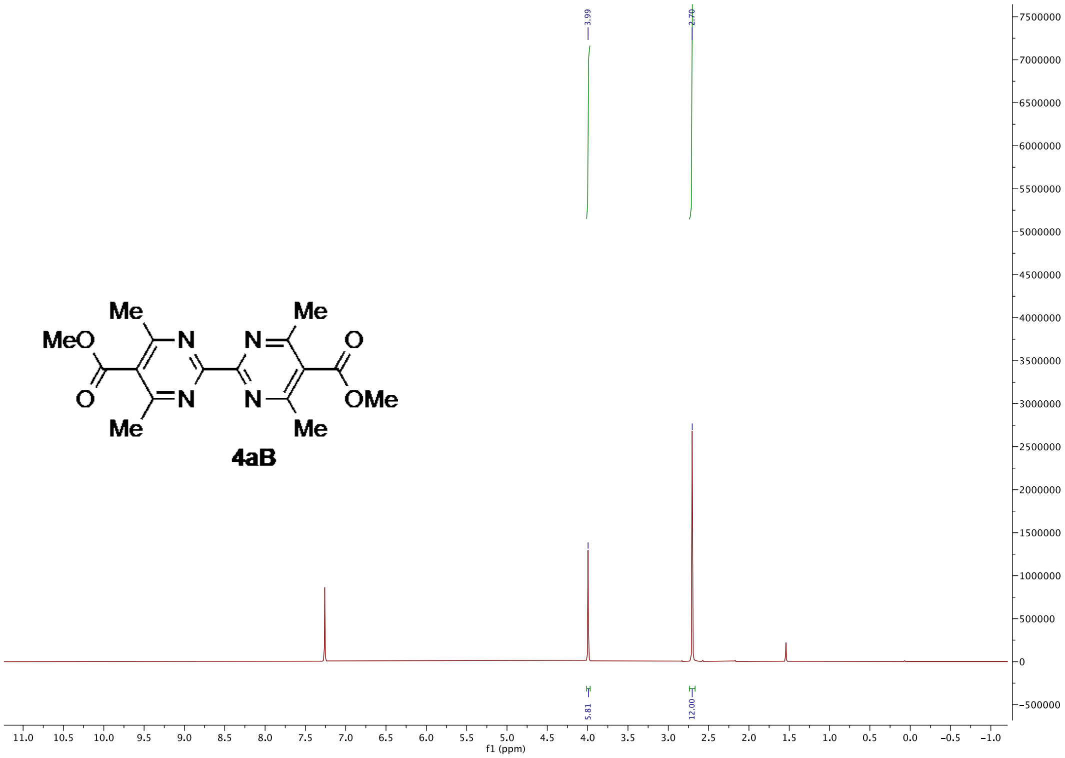

4aB

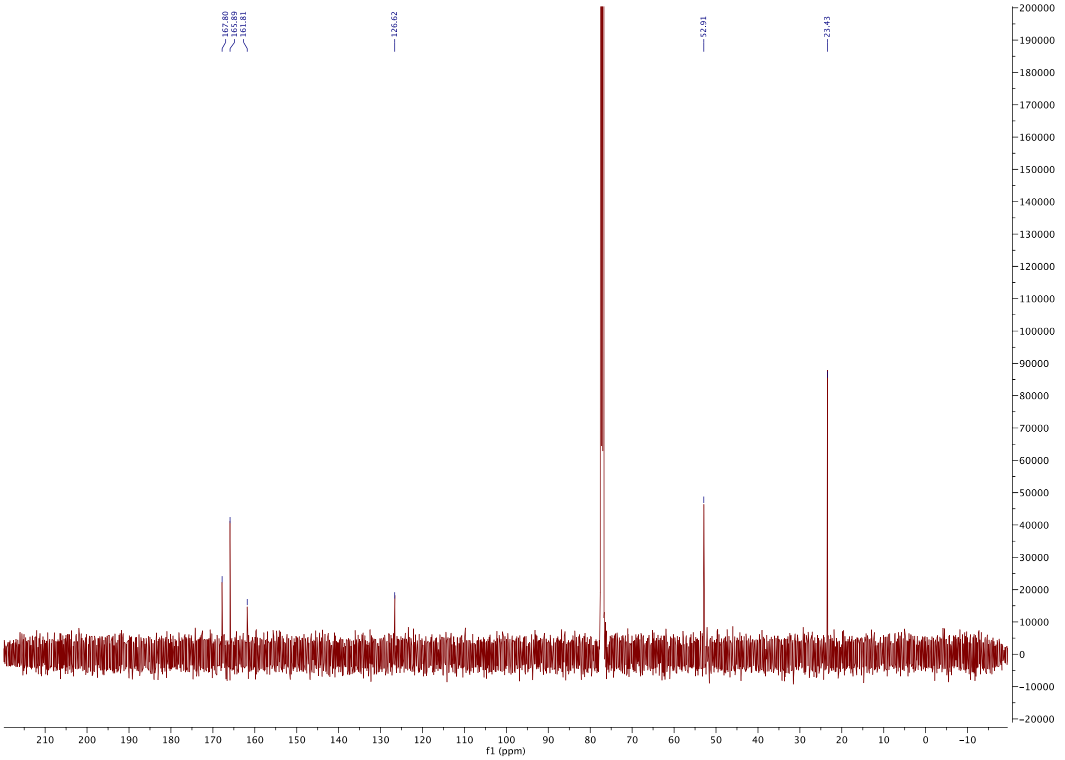

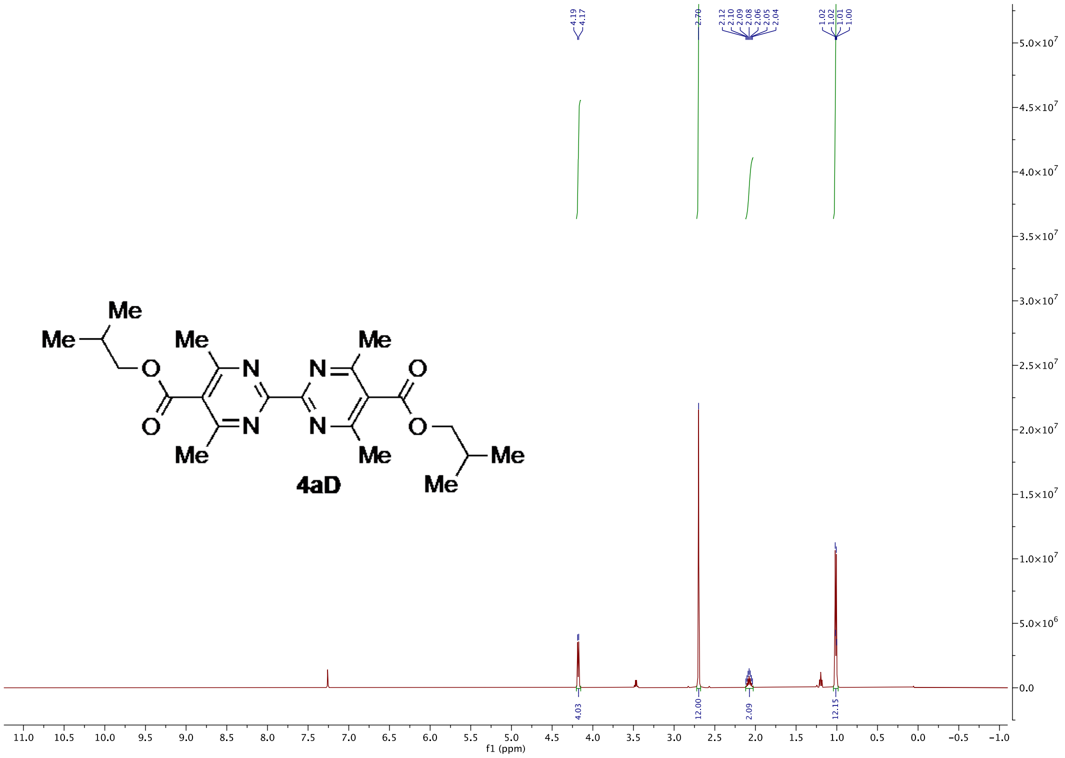
4.19
4.17
2.70
2.12
2.10
2.09
2.08
2.06
2.05
2.04
1.02
1.02
1.01
1.00
4.03
12.00
2.09
12.15
f1 (ppm)
4aD

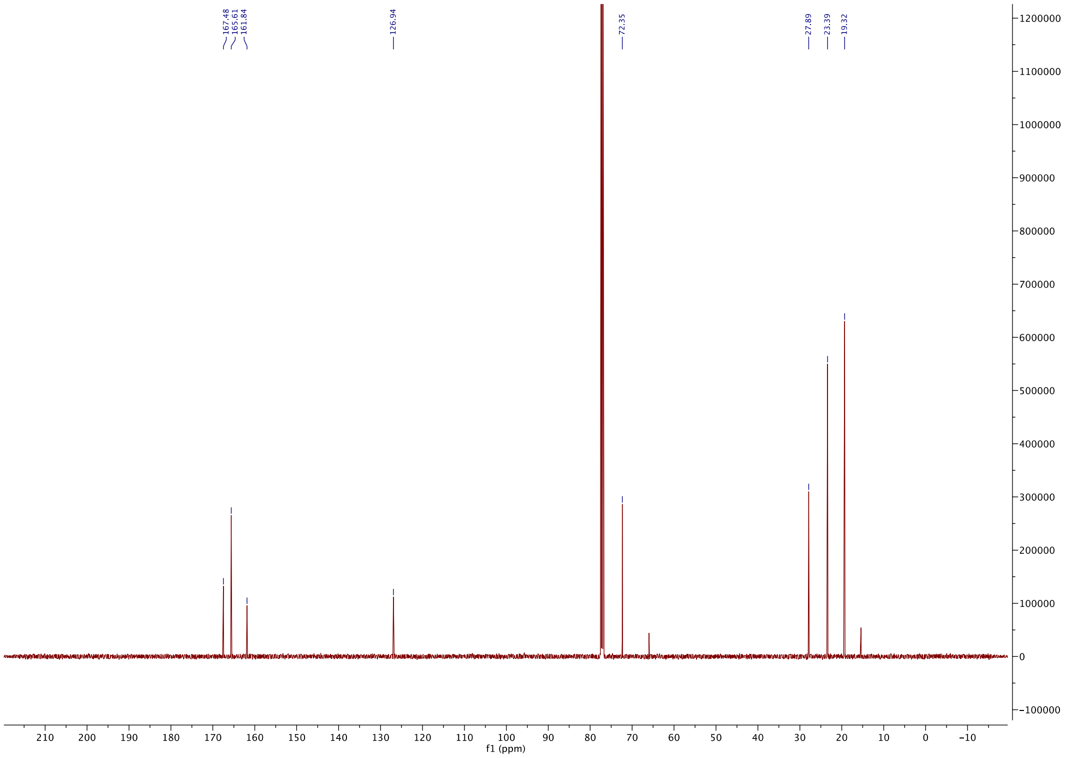
167.48
165.61
161.84
126.94
72.35
27.89
23.39
19.32
f1 (ppm)

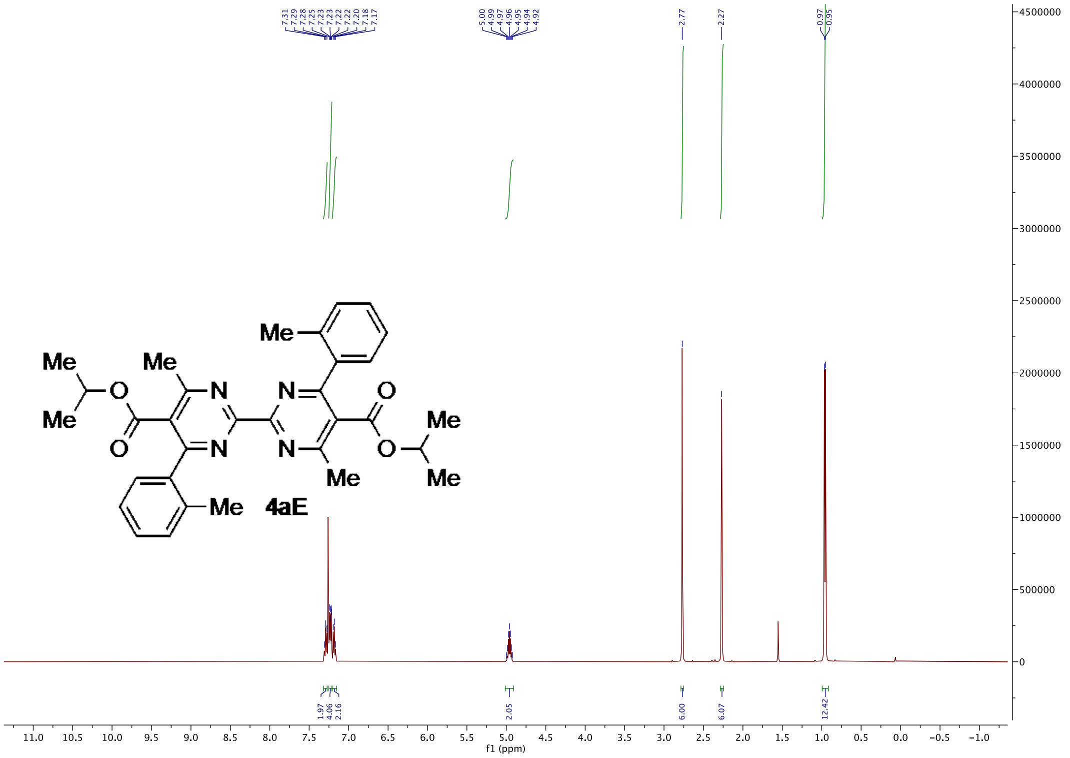
7.31 7.29 7.28 7.25 7.23 7.23 7.22 7.22 7.20 7.18 7.17
5.00 4.99 4.97 4.96 4.95 4.94 4.92
2.77
2.27
0.97 0.95
4aE
1.97 4.06 2.16
2.05
6.00
6.07
12.42
f1 (ppm)

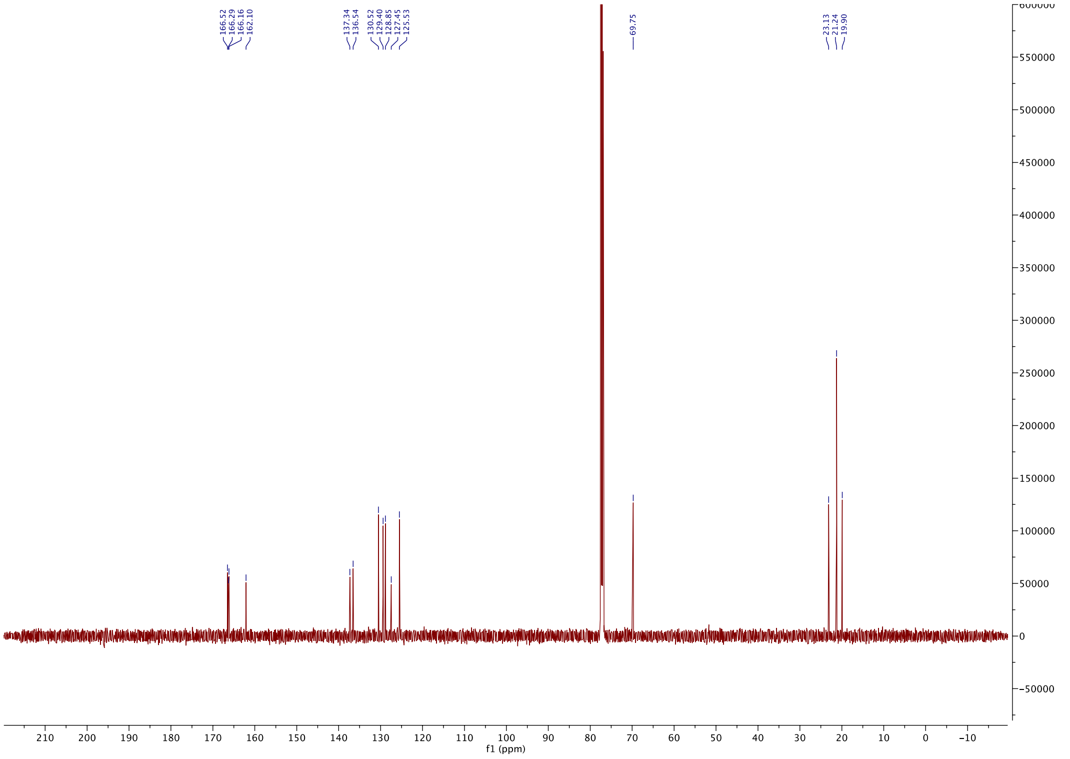
166.52 166.29 166.16 162.10
137.34 136.54 130.52 129.40 128.85 127.45 125.53
69.75
23.13 21.24 19.90
f1 (ppm)

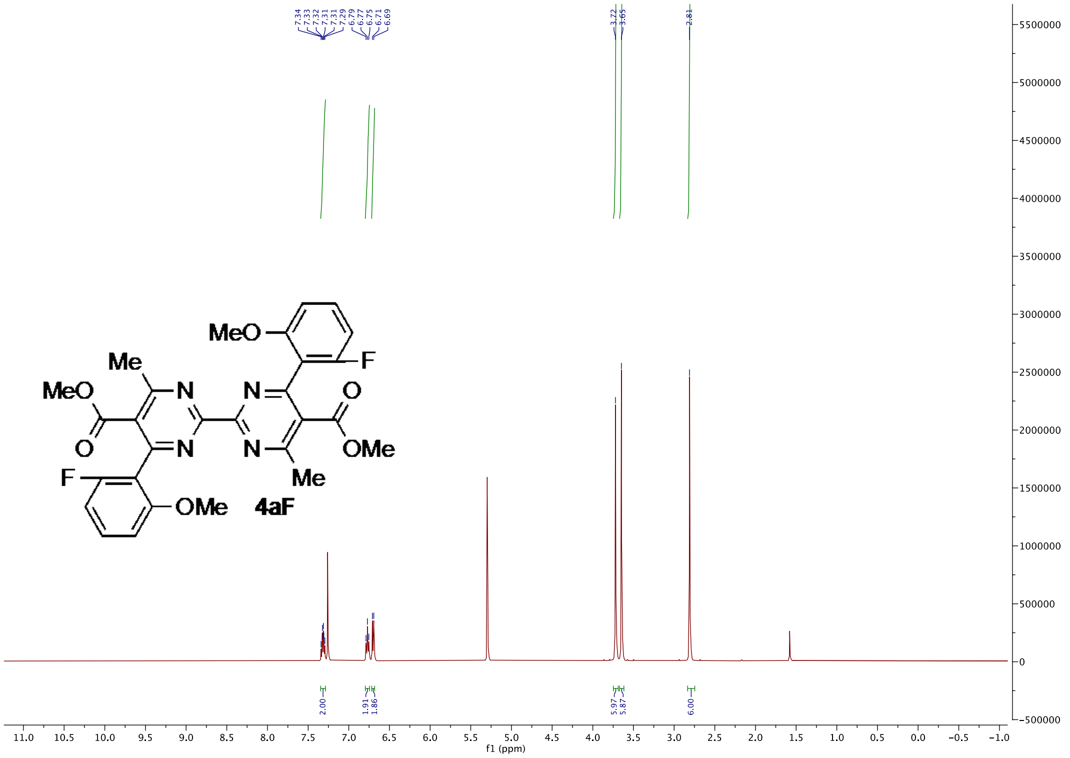
7.34
7.33
7.32
7.31
7.31
7.29
6.79
6.77
6.75
6.71
6.69
3.72
3.65
2.81
MeO
F
Me
MeO
N
N
O
O
O
N
N
OMe
F
Me
OMe
4aF
2.00
1.91
1.86
5.97
5.87
6.00
f1 (ppm)

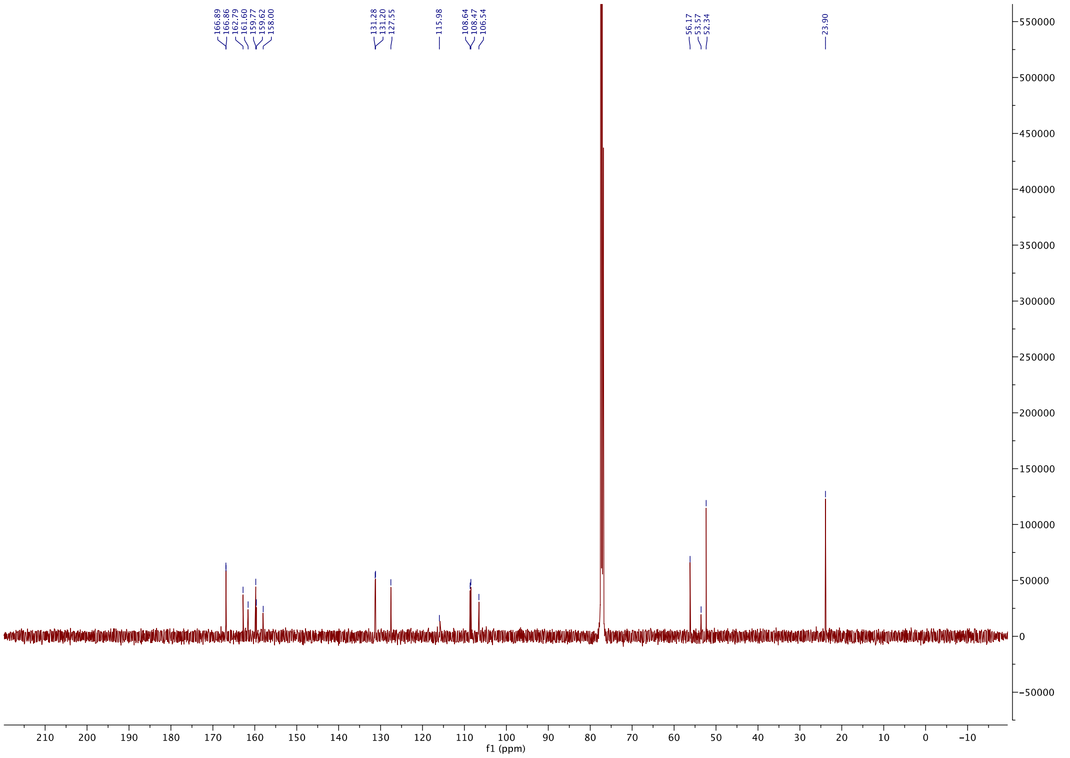
166.89
166.86
162.79
161.60
159.77
159.62
158.00
131.28
131.20
127.55
115.98
108.64
108.47
106.54
56.17
53.57
52.34
23.90
f1 (ppm)

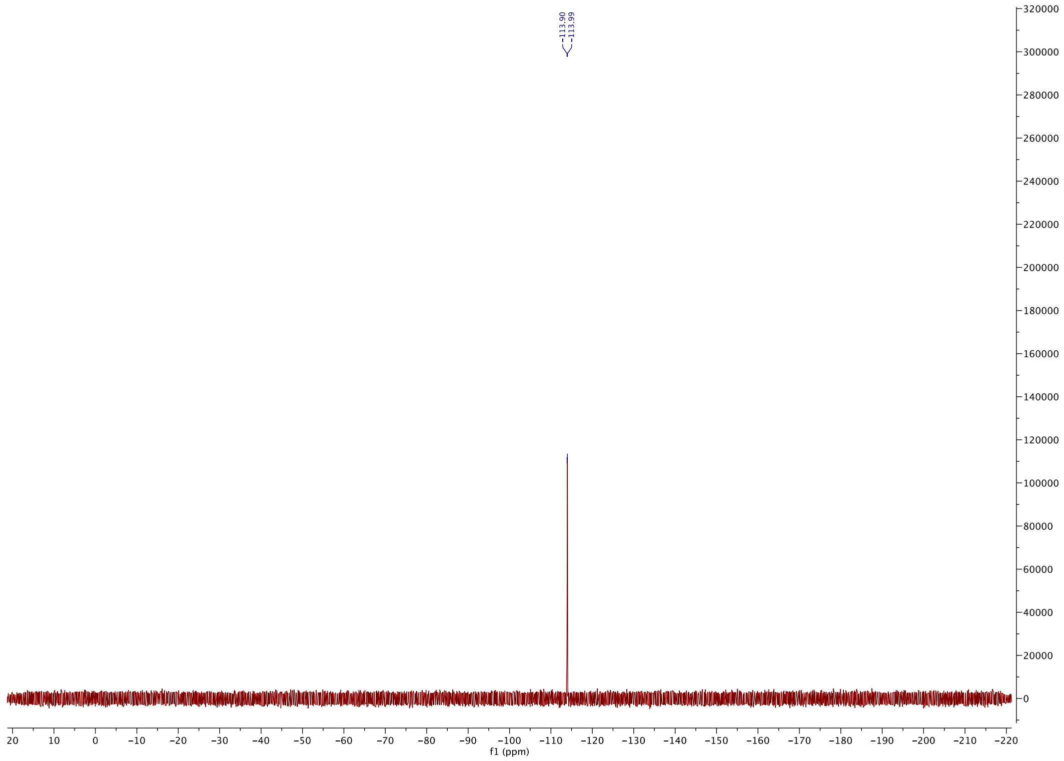

-113.90
-113.99
320000
300000
280000
260000
240000
220000
200000
180000
160000
140000
120000
100000
80000
60000
40000
20000
0
20 10 0 −10 −20 −30 −40 −50 −60 −70 −80 −90 −100 −110 −120 −130 −140 −150 −160 −170 −180 −190 −200 −210 −220
f1 (ppm)
